# Meta-Analysis of Polymyositis and Dermatomyositis Microarray Data Reveals Novel Genetic Biomarkers

**DOI:** 10.3390/genes10110864

**Published:** 2019-10-30

**Authors:** Jaeseung Song, Daeun Kim, Juyeon Hong, Go Woon Kim, Junghyun Jung, Sejin Park, Hee Jung Park, Jong Wha J. Joo, Wonhee Jang

**Affiliations:** 1 Department of Life Science, Dongguk University-Seoul, Seoul 04620, Korea; jaeseung6455@gmail.com (J.S.); as2211453@gmail.com (D.K.); hjyloling@gmail.com (J.H.); swordbreadjj@gmail.com (J.J.); 2 Department of Computer Science and Engineering, Dongguk University-Seoul, Seoul 04620, Korea; gowoone@gmail.com (G.W.K.); sejin8544@gmail.com (S.P.); 3 Western Seoul Center, Korea Basic Science Institute, Seoul 03759, Korea; hjpark8659@kbsi.re.kr

**Keywords:** polymyositis, dermatomyositis, meta-analysis, multiple-phenotype analysis

## Abstract

Polymyositis (PM) and dermatomyositis (DM) are both classified as idiopathic inflammatory myopathies. They share a few common characteristics such as inflammation and muscle weakness. Previous studies have indicated that these diseases present aspects of an auto-immune disorder; however, their exact pathogenesis is still unclear. In this study, three gene expression datasets (PM: 7, DM: 50, Control: 13) available in public databases were used to conduct meta-analysis. We then conducted expression quantitative trait loci analysis to detect the variant sites that may contribute to the pathogenesis of PM and DM. Six-hundred differentially expressed genes were identified in the meta-analysis (false discovery rate (FDR) < 0.01), among which 317 genes were up-regulated and 283 were down-regulated in the disease group compared with those in the healthy control group. The up-regulated genes were significantly enriched in interferon-signaling pathways in protein secretion, and/or in unfolded-protein response. We detected 10 single nucleotide polymorphisms (SNPs) which could potentially play key roles in driving the PM and DM. Along with previously reported genes, we identified 4 novel genes and 10 SNP-variant regions which could be used as candidates for potential drug targets or biomarkers for PM and DM.

## 1. Introduction

Idiopathic inflammatory myositis (IIM) is an extremely rare inflammatory myopathy, whose incidence was reported to be 7.98/million/year in literature based on meta-analysis using the data between 1966 and 2013 [1]. Polymyositis (PM) and dermatomyositis (DM) are the two most common types of IIM in developed countries, presenting muscle weakness and inflammation [2,3]. PM and DM are usually categorized together since the disease progression and muscle lesions are the same, irrespective of whether skin lesions are presented [4]. Serological features and clinical manifestations such as myositis-specific auto-antibodies were also reported for both PM and DM in clinical studies [5,6]. The two diseases exhibit features of an auto-immune disorder; however, their exact pathogenesis has not been clearly demonstrated yet.

IIM comprises subtypes other than PM and DM; there are also juvenile dermatomyositis (JDM) and inclusion body myositis (IBM) [7], which are characterized separately from PM and DM on the basis of several clinical features. For example, even though JDM shares commonalities with adult DM, recent studies reported that JDM is associated with diseases which are not associated with PM or DM [8]. IBM is known to have a slow onset and have very distinctive asymmetric patterns of muscle weakness, particularly in the quadriceps [9].

Some evidence indicates that PM and DM are strongly associated with severe complications such as numerous cancers, cardiac, or vascular diseases. Previous population-based cohort studies and observational studies indicated that the overall risk of malignant disease is increased among patients with PM or DM [10,11]. In addition, venous thromboembolism and coronary artery disease (CAD) can appear as complications of PM and DM with an 11.1-fold and 2.24-fold increased risk, respectively, compared with those in non-IIM patients [12,13,14]. Therefore, it is important to understand the biological mechanisms of PM and DM that can potentially result in detrimental complications.

Previous studies demonstrated that there is a relationship between the muscle lesion and auto-antibodies and muscle-infiltrating immune cells [15]. There are also several reports on the correlations between the two diseases and several genes involved in immune system and/or inflammatory responses such as *nuclear factor kappa B* (*NF-κB*), *tumor necrosis factor a* (*TNF-a*), *interleukin 1 a* (*IL-1a*), *interleukin 22* (*IL-22*), *toll-like receptor 2* (*TLR-2*), *toll-like receptor 4* (*TLR-4*), *toll-like receptor 9* (*TLR-9*), *interferon alpha* (*IFNA*), *interferon gamma* (*IFNG*), and *retinoic acid inducible gene 1* (*RIG-1*) [16,17,18,19,20].

Recently, the importance of genetic variants in gene expression has been emphasized [21]. Genetic variants directly act as expression quantitative trait loci (eQTL) regions affecting adjacent genes are named eGene of the *cis*-eQTL [22]. Studies demonstrated that some *cis*-eQTL of transcription regulatory regions can also indirectly alter the expression levels of genes that lie far from the variant regions or even genes in other chromosomes [23]. These eQTL variants which can regulate the activities of genes over greater distances are called *trans*-eQTL. Therefore, examining both *cis*- and *trans*-effects of the genetic variant regions can expand our understanding of transcriptomic data.

This study is the first meta-analysis to combine multiple microarray datasets from different studies on the two diseases. Our study includes two different tissue sources, skin for DM and muscle for both PM and DM. Thus, we were able to investigate robust genetic expression patterns and detect single nucleotide polymorphic (SNP) variants that covered a broad scale. By combining a meta-analytical and cross-tissue approach, we identified genetic markers which may be potential candidates for new druggable targets.

## 2. Materials and Methods

### 2.1. Data Collection

Raw datasets were collected from EBI-ArrayExpress (E-MTAB-2141: https://www.ebi.ac.uk/arrayexpress/experiments/E-MTAB-2141/; E-MEXP-2681: https://www.ebi.ac.uk/arrayexpress/experiments/E-MEXP-2681/; E-GEOD-46239: https://www.ebi.ac.uk/arrayexpress/experiments/E-GEOD-46239/), which contains data on PM and DM disease groups and healthy control groups. The selected datasets were E-MTAB-2141, E-MEXP-2681, and E-GEOD-46239. Each selected dataset included several disease subtypes; E-MTAB-2141 (PM:3, IBM:6, Control:4), E-MEXP-2681 (PM:4, DM:2, JDM:4, Control:5), and E-GEOD-46239 (PM:48, Control:4). To maintain consistency throughout the study, sample series for IBM or JDM were excluded.

### 2.2. Pre-Processing and Meta-Analysis

Pre-processing of datasets were performed using Oligo Bioconductor package, and the robust multiarray average (RMA) normalization method was used for normalizing each dataset separately [24,25,26,27]. Meta-analysis was carried out by GeneMeta Bioconductor package proposed by Choi et al., in order to reduce the variation across individual datasets. False discovery rate (FDR) and z-scores were computed to identify differentially expressed genes (DEGs) [28]. All steps above were conducted in R (version 3.4.3) following Jung et al. with slight modifications [29].

### 2.3. Gene Set Enrichment Analysis

Gene set enrichment analysis (GSEA) was performed using the pre-ranked method in GSEA Java implementation [30]. The gene list, arranged by z-scores in descending order, were used as pre-ranked gene sets and were converted to the ranked list file format following Jung et al. [31]. Hallmark genes from molecular signatures database (MsigDB, http://software.broadinstitute.org/ gsea/msigdb) were used as gene sets [32].

### 2.4. Network Visualization and Analysis

Core enrichment genes from two GSEA hallmark gene sets, protein secretion and unfolded protein response, were used as the nodes of network. Subsequently, search tool for the retrieval of interacting genes (STRING, https://string-db.org/) was applied for adding edges between the nodes [33]. The confidence level of edges was adjusted to 0.9 and the nodes which were not connected to other nodes were deleted. Using the results from STRING and meta-analysis, a protein-protein interaction (PPI) network was constructed by Cytoscape (version 3.7.0) [34]. Network analysis and subnetwork clustering were carried out by using NetworkAnalyzer Cytoscape tool [35]. The annotation files for the transcription factors were retrieved from transcriptional regulatory relationships unraveled by sentence-based text mining (TRRUST, version 2) database (https://www.grnpedia.org/trrust/) [36].

2.5. cis- and trans-eQTL Region Analysis

Generalized analysis of molecular variance for mixed-model analysis (GAMMA), which can determine the association of multiple phenotypes or expression data with multiple genotypes, was applied to the up-regulated DEGs to analyze eQTL [37]. The analysis was permuted up to 10^6^ times. Muscle data, sun-exposed skin, and not-exposed skin data in the genotype-tissue expression (GTEx, version 6, https://gtexportal.org) project were retrieved for the analysis [38]. A distance of ±1 Mb was used as the margin to identify *cis* activity between SNPs and eGenes. Database for annotation, visualization and integrated discovery (DAVID, http://david.ncif.org) was used to cluster the resulting eGenes from GAMMA [39]. Analysis and interpretation were performed following Jung et al. [40].

## 3. Results

### 3.1. Data Collection and Identifying Differentially Expressed Genes by Meta-Analysis

A total of three microarray datasets including those of PM/DM and healthy control samples were collected from EBI-ArrayExpress (Table 1). We then performed a meta-analysis by using the R package GeneMeta and DEGs were detected by comparing the differential expression levels between the merged (PM and DM) disease group and the control group. The results identified 600 genes as DEGs (FDR < 0.01; up-regulated: z-score > 0; down-regulated: z-score < 0) (Supplementary Table S1). Among these 600 genes, 317 genes were up-regulated and 283 were down-regulated in the merged (PM/DM) disease group compared with that in the control group.

**Figure 1:**
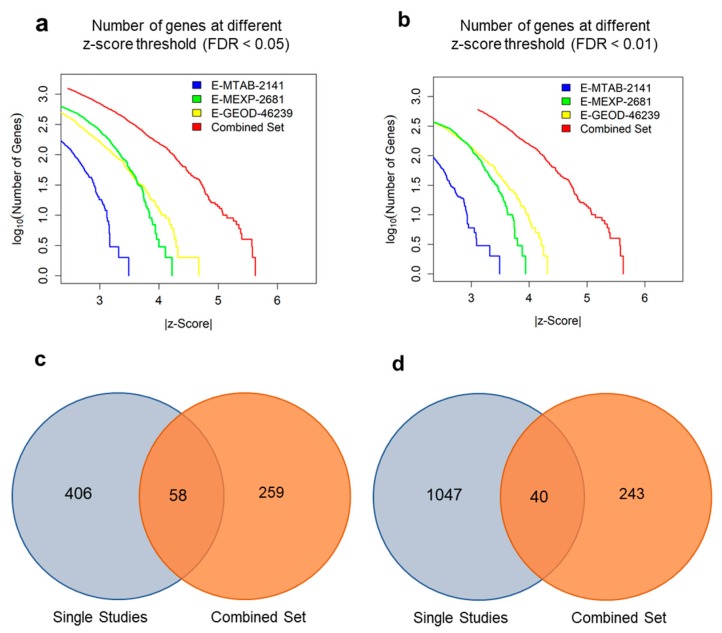
Identification of the DEGs by meta-analysis and comparison with the single study. (**a**) The number of DEGs in the log_10_ scale and their z-scores at false discovery rate (FDR) < 0.05 and (**b**) FDR < 0.01 were consistently increased in combined dataset. (**c**) A Venn-diagram of up-regulated DEGs. DEGs from single studies were the genes which appeared to be significant in at least one dataset (FDR < 0.01). (**d**) A Venn-diagram of down-regulated DEGs. All statistics for single studies and combined set were computed by GeneMeta R package.

Comparing the DEGs obtained from single studies and our merged study revealed that the meta-analysis could detect robust expression signatures which could not be detected in the single studies (Figure 1a,b). The difference between the results from the meta-analysis and single studies indicated that the meta-analysis successfully increased statistical power and sorted out false positive signatures (Figure 1c and d).

We also found that the majority of DEGs obtained from single studies were from one dataset, E-MEXP-2681. Among up-regulated DEGs, we were able to detect the genes associated with PM and/or DM [16,17]. For example, RIG-1 (z-score: 3.44, FDR: 0.00) and TLR2 (z-score: 3.31, FDR: 0.00) were significantly up-regulated in the merged-disease group. We examined the top 10 up-regulated DEGs and the data showed that 6 out of 10 genes were related to inflammation or immune response (Table 2) which were bone marrow stromal cell antigen 2 (BST2), alpha-2-macroglobulin (A2M), platelet and endothelial cell adhesion molecule 1 (PECAM1), insulin like growth factor 1 (IGF1), serpin family G member 1 (SERPING1), and CD163 molecule (CD163) [41,42,43,44,45,46]. By detecting the genes which are related with inflammation and/or immunity, we were able to validate that our results well conformed with previous studies.

### 3.2. Identifying Enriched Biological Pathways Using GSEA

In order to examine the biological effects of the gene expression profiles of PM/DM, we performed GSEA, which weighs each gene based on rank in the ordered gene set. This clustering method allows the interpretation of the global gene-expression pattern without any loss in information. Entire 10,032 genes from the meta-analysis were ranked with the z-scores obtained from GeneMeta.

**Figure 2:**
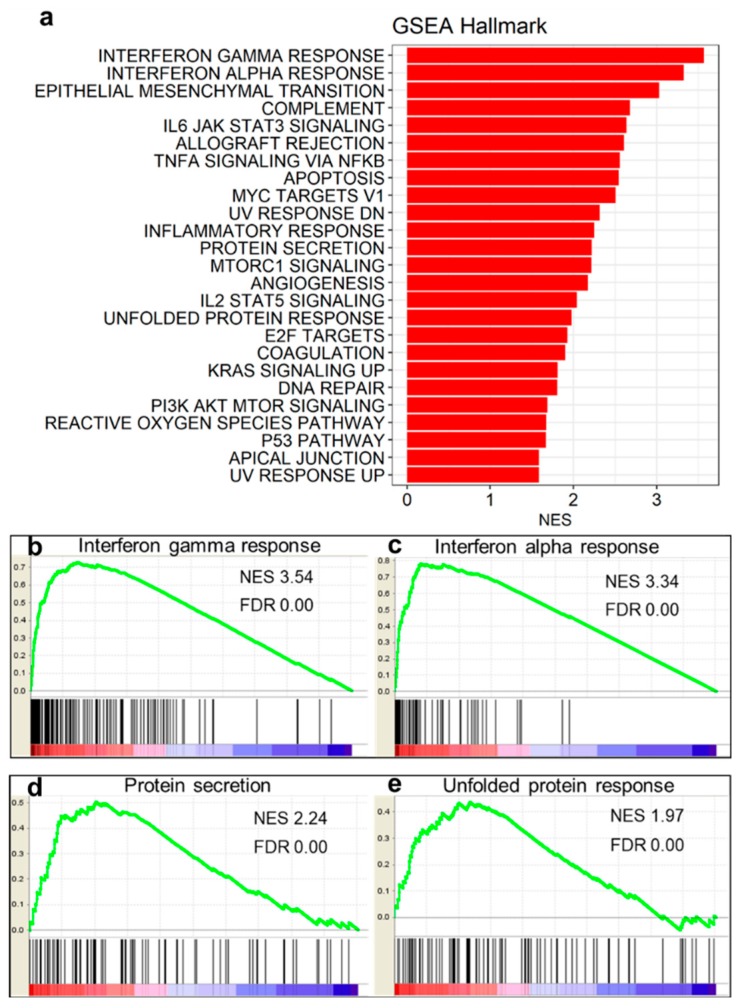
Gene set enrichment analysis results with hallmark gene sets. Whole genes ordered by the z-scores from the meta-analysis were used as ranked genes for analysis. (**a**) Top positively enriched biological pathways from gene set enrichment analysis (GSEA) hallmark gene sets. Normalized enrichment score (NES) is a relative value of enrichment which indicates how positive or negative the enrichment of gene set is, compared to other gene sets. GSEA plots of gene sets of intereset are displayed. (**b**) Interferon gamma response, (**c**) interferon alpha response, (**d**) protein secretion, and (**e**) unfolded protein response gene sets were positively enriched.

The ranked gene sets were tested on the hallmark gene sets, which provides the clusters of genes in broad categories. By conducting GSEA with the hallmark gene sets, we successfully identified 25 positively enriched gene sets with FDR < 0.01 (Figure 2a). The majority of them were enriched in the biological pathways which are directly associated with inflammation or immunity, such as the interferon alpha signaling pathway or immune response, respectively [48]. However, we obtained only 3 significantly down-regulated gene sets (FDR < 0.01), which were “KRAS signaling down”, “estrogen response early”, and “estrogen response late”. Especially, negative enrichment of “KRAS signaling down” implies that KRAS signaling pathway was up-regulated. The result agreed with our findings that “KRAS signaling up” gene set was significantly positively enriched. This suggests that only two gene sets were significant in down-regulated gene sets, thus were removed from this study.

The top two positively enriched gene sets were those of interferon gamma response (Figure 2b, FDR: 0.00 and normalized enrichment score, NES: 3.54) and interferon alpha response (Figure 2c, FDR: 0.00, NES: 3.34). These pathways are representatives of auto-immune inflammatory diseases [49]. From our analysis, we also identified 2 gene sets which were not previously mentioned in single study: protein secretion (Figure 2d, FDR: 0.00, NES: 2.24) and unfolded protein response (Figure 2e, FDR: 0.00, NES: 1.97). Grootjans et al. suggested that endoplasmic reticulum (ER) stress is associated with unfolded protein response, which may be related to inflammatory responses and auto-immune diseases [50]. In addition, a recent review by Manole et al. suggested that there may be a possible relationship between unfolded protein response and IIM [51]. In this study, we newly identified that the ER stress was related to unfolded protein response in PM and DM. Together, our GSEA plot indicated that the merged-disease group not only showed expression patterns which can be regarded as common characteristics of auto-immune diseases but also presented abnormally up-regulated pathways involved in ER stress (Figure 2).

### 3.3. Network Construction of Protein Secretion and Unfolded Protein Response Gene Sets

**Figure 3:**
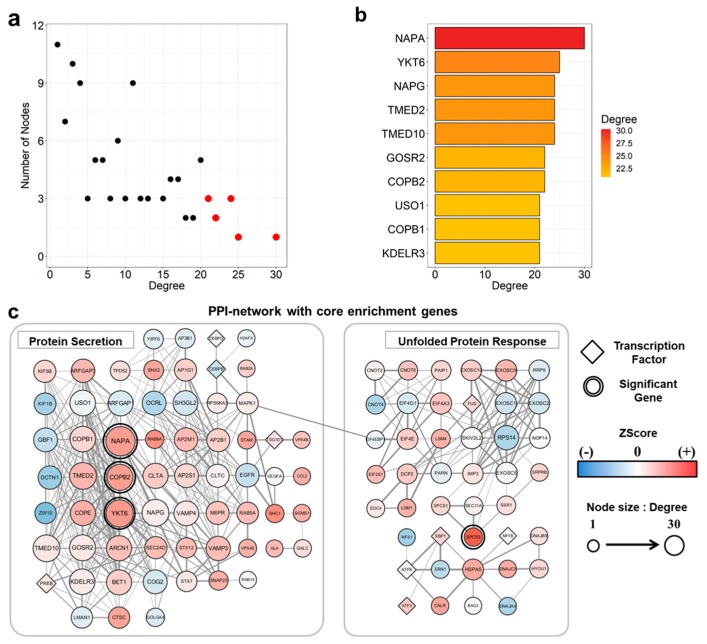
A protein-protein interaction (PPI)-network and its information obtained from protein secretion and unfolded protein response gene sets. (**a**) A Scatter plot showing the degree distribution. The nodes with the top 10 highest degrees were annotated with red dots. (**b**) The profiles and the degrees of the top 10 nodes. The color of the bar indicates the degree of the genes in PPI-network. (**c**) The PPI-network of protein secretion and unfolded protein responses and their link. The colors of nodes indicate up- or down-regulated genes. Red nodes indicate up-regulation and blue nodes indicate down-regulation. The sizes of the nodes are proportional to the degrees of the nodes. The identified novel genes were marked with double circles.

As an extension of functional annotation and the GSEA study, we constructed a gene network using 150 core enrichment genes of protein secretion and unfolded protein response gene sets, which were rarely highlighted in previous studies (Figure 3 and Supplementary Table S2). Among the total 150 genes retrieved from STRING, only 107 genes had a corresponding probe in the merged PM/DM data and were incorporated into the final network. We observed that the two separate gene sets (protein secretion and unfolded protein response) were linked to each other via interaction of MAPK1 and EIF4EBP1.

Next, we narrowed down the genes by two steps. First, we selected the genes which were both included in the up-regulated DEGs and the PPI network. Then, the genes listed as the top 10 genes with the most positive fold-changes (Table 2) or those that had the highest degrees on the network among the 107 genes were selected (Figure 3a and b), because both characteristics are equally important in revealing the pathogenesis of PM and DM. Among the genes that were selected, we found 3 genes that were not mentioned in previous studies, thus seemed to be novel. The genes were YKT6 v-SNARE homolog (YKT6, z-score: 3.48, FDR: 0.00, Degree: 29), NSF attachment protein alpha (NAPA, z-score: 3.38, FDR: 0.00, Degree: 34), and coatomer protein complex subunit beta-2 (COPB2, z-score: 3.33, FDR: 0.01, Degree: 24). In addition to these genes, we selected signal peptidase complex subunit 3 (SPCS3, z-score: 5.58, FDR: 0.00, Degree: 4). Even though it only had 4 degrees in the pathway, SPCS3 showed a 5.58 fold-change in z-score, which were the second highest value among those of up-regulated DEGs. These 4 genes were marked with double circles in the PPI-network (Figure 3c). All novel genes we proposed were both enriched in either protein secretion or unfolded protein response and were statistically significantly up-regulated (FDR < 0.01). These significant genes could be suggested as novel genes because they were not mentioned as causal genes for PM or DM in previous studies.

### 3.4. Identifying Potential cis- or trans-eQTL Regions

In addition to the gene expression profiles, the role of genetic variants such as eQTL loci in the pathogenesis of various diseases has been recently emphasized [52,53]. In order to determine the eQTL regions that could potentially affect the pathogenesis of the two diseases, we performed GAMMA, which is a powerful multiple-phenotype regression analysis to correct population structure in multi-variate analysis with high accuracy in detecting variants that reduce false positives [37]. GAMMA allows inference of the potential genetic variants from the gene expression data or phenotype information. By conducting an analysis against multiple tissues, we were able to identify SNPs that could specifically contribute to the pathogenesis of PM and DM.

Because our target diseases usually present symptoms in muscle and/or skin, we used the data of muscle tissue, sun-exposed skin tissue, and not-sun-exposed skin tissue from GTEx version 6. We then performed GAMMA on the 317 up-regulated DEGs and obtained 170 potential regulatory SNPs for 42 eGenes in muscle tissue (Supplementary Table S3), 112 SNPs for 40 eGenes in sun-exposed skin tissue (Supplementary Table S4), and 154 SNPs for 27 eGenes in not-sun-exposed skin tissue (Supplementary Table S5). Because the analyses were permuted to detect 1.0 × 10^−5^ in maximum significance, we determined the significance as *p* < 1.0 × 10^−4^ and *p* < 5.0 × 10^−4^ for GTEx and GAMMA, respectively. The p-value of GTEx indicates the association between detected SNPs and eGenes, whereas that of GAMMA indicates the significance of variant detection. Together, we suggest that the identified SNPs may be possible regulatory eQTLs affecting the expression of the up-regulated DEGs.

In order to clarify the causal relationship between the eGenes from each tissue and DEGs, we clustered eGenes using the DAVID functional clustering method. Functional category which involved most large number of gene were selected from each tissue. Genes and their eQTL loci from selected functions were presented (Table 3 and Table 4). As a result, we obtained at least one significant cis-eQTL site each from muscle and sun-exposed tissues, but none from the not-exposed tissue. The enriched term was “regulation of transcription, DNA-templated” from the muscle tissue, which contained 7 variants for 4 genes (Table 3). Three SNPs, rs587638658, rs115256213, and rs12925855, were located on chromosome 1, 6, and 16, respectively. The other 4 variants, rs61916118, rs59992343, rs11221871, and rs11221861, which were associated with NFRκB, were all located on chromosome 11. From the sun-exposed skin tissue, 3 variants for 7 genes were clustered to “immunoglobulin-like domain” (Table 4). Two variants, rs9269294 and rs75364579, were located on chromosome 6, showing significant association with various human leukocyte antigen (HLA) alleles and one variant, rs397600, on chromosome 19 was associated with the eGene LILRB2.

We subsequently examined the biological meanings of the detected eGenes in muscle tissues (Table 3). The variants detected from the muscle tissues could be interpreted as trans-eQTL regulatory regions for PM and DM. For example, NFRκB co-expresses with NF-κB in various inflammatory or auto-immune symptoms [54,55]. Therefore, it seems possible to stipulate that the variants for NFRκB might act as the trans-eQTL region for PM and DM [56]. The protein encoded by ATF7IP2, another eGene from the muscle tissue, is known to play a role in protein folding and act as the transcriptional regulator [57]. Therefore, the genetic variant for ATF7IP2 may abnormally regulate unfolded protein response in patients with PM/DM. The protein encoded by ZFP57, a previously reported potential repressor of unfolded protein response, was also detected as an eGene for the variant rs115256213 [58]. Even though the function of ZNF697 is not clearly identified, numerous studies have suggested that it may play a key role in various biological processes such as DNA recognition, RNA packaging, transcriptional activities, apoptotic regulation, protein folding process, and lipid binding, similar to other zinc finger protein families [59].

The detected eGenes from sun-exposed skin tissue were emphasized in previous studies and could be interpreted as potential cis-eQTL candidates (Table 4). HLA alleles play an important role in auto-immune diseases and the HLA families detected as eGenes from sun-exposed skin tissues were HLA-DQA1, HLA-DRB1, and HLA-DQB1. These alleles are reported as genetic risk factors associated with myositis phenotypes such as PM and DM [60]. However, the SNPs resulted from our study were not reported before [60]. Similarly, HLA-DQA1 was reported to have a positive association with DM [61]. Another eGene from sun-exposed skin tissue was LILRB2, which is genetically associated with several auto-immune conditions. Previous studies showed that LILR families bind to HLA class 1 and our analysis also showed that LILRB2 may bind to HLA-B [62]. Therefore, altered LILRB2 could be a contributing factor to auto-immune disease. Additionally, the advanced glycosylation end product-specific receptor (AGER) gene encodes the receptor for advanced glycation end products protein, which is a multi-ligand receptor involved in inflammation.

## 4. Discussion

Meta-analysis provides reliable results by combining multiple studies [28,29]. By using meta-analysis, we were able to attain more precisely pooled results from multiple individual studies compared with that from single studies (Figure 1). The data we used for this study originally contain other subtypes such as IBM or JDM. However, they were excluded for analyses due to following reasons. Although IBM and PM have similar morphological appearance, there are critical differences between IBM and PM/DM with respect to clinical features [63]. PM/DM patients are usually treated with anti-inflammatory or immunosuppresive therapies, whereas those with IBM do not respond to such treatments. The weakness patterns observed in IBM also differ from those observed in PM/DM [7,63]. In addition, there are some differences between JDM and PM/DM. The major difference between JDM and PM/DM is the age of onset. JDM usually manifests around the age of 6, whereas general onset of PM/DM is around the age of 20 [8,64]. Since the source of samples for JDM are much younger in age than those for PM and DM, the overall gene expression level may be different due to the variance between children and adults, not just by the presence of the disease itself. Because we believed that including the data on IBM or JDM may distort the genuine disease signatures, we only used the two most representative subtypes of IIM, PM and DM [65].

Our results showed that PM and DM were closely related to inflammatory responses or immune mechanisms such as interferon signaling responses which correspond with previous studies (Figure 2a) [15,16,17,18,19,20]. The GSEA results also showed that interferon gamma and interferon alpha gene sets were enriched, which also supports the previous findings (Figure 2b–e).

Although PM and DM are inflammatory myopathies, we sought to reveal non-inflammatory contributions of these diseases by using meta-analysis. As a result of meta-analysis, we identified that genes involved in protein secretion and unfolded protein response pathways are associated with PM/DM (Figure 3). Notably, *thymosin beta 10* (*TMSB10*, z-score: 5.62, FDR: 0.00, Entrez ID: 9168) showed the largest difference in expression levels among statistically significant up-regulated DEGs. There is ample evidence suggesting that *TMSB10* is one of the key regulators of malignancy and metastasis in various types of cancers and PM and DM are well-known for its association with malignancy [66,67]. Together, our results suggest that the acquired malignancy in PM and DM may be the result of the abnormal expression of *TMSB10*.

Previous studies suggested that the pathogenesis of PM and DM is influenced by reactive oxygen species (ROS) and mitochondrial damage [68]. It has been suggested that there is a correlation between interferon-induced ROS, unfolded protein response, ER stress, and mitochondrial activity in inflammatory diseases [69]. *Heat shock protein family A* (*Hsp70*) *member 5* (*HSPA5*, z-score: 2.94, FDR: 0.02, Entrez ID: 3309), which is included in our gene network, is known to play a role in ER stress (Figure 3c) [70]. In addition, a previous study detected that HSPA5 level were increased in the patients with PM or DM, which participates in ER stress [71]. Because HSPA5 was included in our PPI-network using genes from protein secretion and unfolded protein response and was up-regulated marginally significantly (FDR < 0.05), the data support our hypothesis that the two pathways may contribute to pathogenesis of PM and DM. Previous studies mentioned that protein metabolism pathways may indirectly be involved with pathogenesis of PM and DM [50,51]. The three newly identified genes in this study, *YKT6*, *NAPA*, and *COPB2*, encode proteins that are involved in the transportation from ER to Golgi structure, vesicle docking, and Golgi budding, respectively [72,73,74]. Another novel gene, *SPCS3*, encodes a component of microsomal signal peptidase [75]. These genes are all involved in the pathways of protein secretion or unfolded protein response. Together, we suggest that PM and DM are highly related with protein secretion and unfolded protein response pathways.

All eGenes in Table 3 and Table 4 have been previously mentioned to have a positive correlation with malignancy or some other severe complication of PM and DM [75,76,77,78,79,80,81,82,83,84,85]. NF-κB signaling and zinc finger proteins are known for their activity in key mechanisms of cancers such as migration, autophagy, apoptosis, cytokine processing, and metastasis [76,77,78,79]. Unfolded protein response, in which one of the eGenes *ATF7IP2* was involved, is also reported to contribute to tumor progression and carcinogenesis [80]. LILRB2 was proposed as a key player in the signaling pathway of lung cancer development [81]. HLA families are reported to have an association with various cancer types [82]. In addition, Liu et al. demonstrated that HLA families might play a protective role against CAD, which is one of the major complications of PM and DM [83]. Another eGene *AGER* is expressed primarily in the lung and has polymorphisms that may potentially increase the risk of lung cancer [84]. Detecting *AGER* as eGene agrees with the previous finding of the association between lung cancer and PM/DM [85]. Collectively, our analysis successfully captured several known genes implicated in complications of PM and DM patients, which may validate the soundness of our study.

This study, however, has some limitations. First, because our results were only derived from in silico analysis, they might not be ensured under physiological conditions. Further in vivo or in vitro studies are necessary to validate the actual underlying biological mechanisms of inflammatory myopathies. Second, down-regulated DEGs were excluded after examining the functional annotation using GSEA due to their ambiguous annotations. Third, because we could not retrieve enough data for each tissue sources, the eQTL analyses were performed with integrated result. Therefore, this result may need to be reconfirmed by further analysis conducted using separate tissue sources in large sample size. Despite of these limitations, our study successfully identified potential genetic markers of PM and DM using meta-analysis. These genetic signatures, if confirmed in a larger independent data set or through functional studies, could be applied to the development of targeted therapy or genetic diagnosis.

## Figures and Tables

**Table 1:** Information on the datasets included in this study.

ArrayExpress ID	PMID	Source	Platform	Organism	PM	DM	Control	Total
**E-MTAB-2141**	24462217 [47]	Muscle Biopsy	Affymetrix GeneChip Human Exon 1.0 ST Array version 2	Human	3	-	4	7
**E-MEXP-2681**	-	Muscle Biopsy	Affymetrix GeneChip Human Genome HG-U133A	Human	4	2	5	11
**E-GEOD-46239**	-	Skin Biopsy	Affymetrix GeneChip Human Genome U133 Plus 2.0	Human	-	48	4	52
Total	-	-	-	-	7	50	13	70

**Table 2:** Top 10 up-regulated differentially expressed genes (DEGs) between the merged-disease group and the control group.

Gene Symbol	Full Name	Entrez ID	z-Score	FDR
***TMSB10***	*thymosin beta 10*	9168	5.62	0.00
***SPCS3***	*signal peptidase complex subunit 3*	60559	5.58	0.00
***BST2***	*bone marrow stromal cell antigen 2*	684	5.31	0.00
***A2M***	*alpha-2-macroglobulin*	2	5.14	0.00
***PECAM1***	*platelet and endothelial cell adhesion molecule 1*	5175	5.07	0.00
***IGF1***	*insulin like growth factor 1*	3479	5.07	0.00
***SERPING1***	*serpin family G member 1*	710	4.95	0.00
***SLC25A24***	*solute carrier family 25 member 24*	29957	4.84	0.00
***CD163***	*CD163 molecule*	9332	4.84	0.00
***TM4SF1***	*transmembrane 4 L six family member 1*	4071	4.83	0.00

**Table 3:** Single nucleotide polymorphisms (SNPs) and eGenes significantly detected from muscle tissue, genotype-tissue expression (GTEx) version 6.

Refsnp ID	GAMMA *p* Value	GTEX *p* Value	ENSEMBL ID	Gene Symbol	Description
**rs61916118**	9.00 × 10^−5^	2.06 × 10^−6^	ENSG00000170322	*NFRκB*	*nuclear factor related to kappaB binding protein*
**rs59992343**	9.00 × 10^−5^	3.60 × 10^−6^			
**rs11221871**	1.01 × 10^−4^	1.42 × 10^−6^			
**rs11221861**	10.9 × 10^−4^	3.88 × 10^−6^			
**rs115256213**	2.10 × 10^−4^	5.00 × 10^−8^	ENSG00000204644	*ZFP57*	*ZFP57 zinc finger protein*
**rs12925855**	2.40 × 10^−4^	6.10 × 10^−5^	ENSG00000166669	*ATF7IP2*	*activating transcription factor 7 interacting protein 2*
**rs587638658**	3.10 × 10^−4^	9.95 × 10^−5^	ENSG00000143067	*ZNF697*	*zinc finger protein 697*

**Table 4:** SNPs and eGenes significantly detected from sun-exposed skin tissue detected in GTEx version 6.

Refsnp ID	GAMMA *p* Value	GTEX *p* Value	ENSEMBL ID	Gene Symbol	Description
**rs397600**	6.20 × 10^−5^	5.47 × 10^−7^	ENSG00000131042	*LILRB2*	*Leukocyte immunoglobulin-like receptor, subfamily B (with TM and ITIM domains), member 2*
**rs9269294**	2.50 × 10^−4^	3.77 × 10^−6^	ENSG00000204305	*AGER*	*Advanced glycosylation end product-specific receptor*
		4.13 × 10^−14^	ENSG00000196735	*HLA-DQA1*	*Major histocompatibility complex, class II, DQ alpha 1*
		3.53 × 10^−11^	ENSG00000223534	*HLA-DQB1-AS1*	*HLA-DQB1 antisense RNA 1*
		1.35 × 10^−12^	ENSG00000179344	*HLA-DQB1*	*Major histocompatibility complex, class II, DQ beta 1*
		1.54 × 10^−22^	ENSG00000196126	*HLA-DRB1*	*Major histocompatibility complex, class II, DR beta 1*
**rs75364579**	3.30 × 10^−4^	2.82 × 10^−5^	ENSG00000179344	*HLA-DQB1*	*Major histocompatibility complex, class II, DQ beta 1*

## Supplementary Materials

The following are available online at https://www.mdpi.com/2073-4425/10/11/864/s1, Table S1: Total list of 600 DEGs and their statistics, Table S2: Table used for network construction with tsv file format, Table S3: Detected SNPs and eGenes in muscle tissue, Table S4: Detected SNPs and eGenes in sun-exposed skin tissue, Table S5: Detected SNPs and eGenes in not-sun-exposed skin tissue.
