# Supplementary material for: Meta-Analysis of Polymyositis and Dermatomyositis Microarray Data Reveals Novel Genetic Biomarkers
Source: Genes (Basel). 2019 Oct 30;10(11):864. doi: 10.3390/genes10110864 (PMC6895911; doi:10.3390/genes10110864)
Supplement: Supplementary file 1 [file genes-10-00864-s001.zip › Supplementary_files/Supplementary Table 1.pdf]

Supplementary Table 1. Total list of 600 DEGs and their statistics

Entrez ID	zSco_Ex_1	FDR_Ex_1	zSco_Ex_2	FDR_Ex_2	zSco_Ex_3	FDR_Ex_3	zSco	FDR	MUvals	MUsds	Qvals	df	Qpvalues	Chisq
10010	1.16	0.32	2.47	0.03	1.83	0.23	3.14	0.01	1.18	0.38	0.92	2	0.63	0
1E+08	-1.29	0.27	-2.69	0.02	-2.25	0.14	-3.61	0	-1.38	0.38	0.96	2	0.62	0
10019	1.16	0.32	3.22	0.01	2.53	0.1	3.42	0	1.58	0.46	2.59	2	0.27	0
10026	0.97	0.44	1.45	0.2	3.17	0.05	3.42	0	1.26	0.37	1.43	2	0.49	0
1003	2.59	0.11	2.35	0.04	3.38	0.04	4.78	0	1.92	0.4	0.78	2	0.68	0
10045	-1.24	0.29	-2.45	0.03	-2.65	0.09	-3.76	0	-1.42	0.38	0.46	2	0.8	0
10089	-1.68	0.18	-2.99	0.01	-1.86	0.22	-3.55	0	-1.43	0.4	2.08	2	0.35	0
10135	1.36	0.25	2.31	0.04	1.94	0.2	3.24	0.01	1.21	0.37	0.46	2	0.8	0
10161	1.85	0.16	2.53	0.03	2.56	0.1	4.02	0	1.55	0.39	0.25	2	0.88	0
10184	2.4	0.12	2.3	0.04	2.77	0.08	4.26	0	1.67	0.39	0.63	2	0.73	0
10186	2.04	0.14	3.28	0.01	2.44	0.11	4.14	0	1.78	0.43	2.17	2	0.34	0
10211	0.99	0.42	1.51	0.18	2.94	0.06	3.31	0.01	1.22	0.37	0.94	2	0.63	0
10269	1.46	0.22	3.08	0.01	2.16	0.16	3.75	0	1.48	0.39	2.03	2	0.36	0
10278	-1.25	0.29	-2.09	0.06	-2.62	0.09	-3.55	0	-1.33	0.37	0.19	2	0.91	0
10279	-1.88	0.15	-3.01	0.01	-2.66	0.09	-4.32	0	-1.72	0.4	0.98	2	0.61	0
10346	1.98	0.14	3.88	0	3.02	0.06	3.26	0.01	2.33	0.72	4.56	2	0.1	0
10393	0.73	0.63	2.91	0.01	3.42	0.04	3.52	0	1.63	0.46	2.71	2	0.26	0
10403	1.36	0.25	1.31	0.25	2.96	0.06	3.38	0	1.24	0.37	0.91	2	0.63	0
10420	-2.21	0.13	-2.72	0.02	-2.82	0.07	-4.45	0	-1.77	0.4	0.44	2	0.8	0
10434	0.82	0.56	2.55	0.03	1.98	0.2	3.12	0.01	1.17	0.38	1.36	2	0.51	0
10497	-1.29	0.27	-1.3	0.25	-2.91	0.07	-3.31	0.01	-1.22	0.37	0.89	2	0.64	0
10512	1.32	0.26	1.63	0.15	3.31	0.04	3.78	0	1.41	0.37	1.03	2	0.6	0
10561	1.48	0.22	1.93	0.09	3.42	0.04	4.11	0	1.55	0.38	0.68	2	0.71	0
10652	1.8	0.16	2.26	0.05	2.05	0.18	3.48	0	1.32	0.38	0.39	2	0.82	0
10671	0.84	0.54	2.73	0.02	2.07	0.17	3.27	0.01	1.24	0.38	1.74	2	0.42	0
1072	2.13	0.14	2.8	0.02	1.74	0.26	3.57	0	1.45	0.41	2.1	2	0.35	0
10791	2.38	0.12	2.92	0.01	2.81	0.07	4.6	0	1.86	0.4	0.91	2	0.63	0
10797	1.78	0.16	2.26	0.05	2.41	0.11	3.74	0	1.42	0.38	0.12	2	0.94	0
10848	-1.32	0.26	-2.73	0.02	-1.97	0.2	-3.44	0	-1.31	0.38	1.29	2	0.53	0
10964	1.37	0.24	2.65	0.02	3.72	0.03	4.66	0	1.82	0.39	0.96	2	0.62	0
10990	2.25	0.13	2.26	0.05	1.88	0.22	3.53	0	1.36	0.38	1.24	2	0.54	0
1101	-1.72	0.17	-1.8	0.11	-2.49	0.1	-3.51	0	-1.31	0.37	0.08	2	0.96	0
11010	2.41	0.12	1.83	0.1	1.73	0.26	3.22	0.01	1.23	0.38	1.75	2	0.42	0
11021	2.56	0.12	1.42	0.2	2.13	0.16	3.14	0.01	1.27	0.4	2.19	2	0.33	0
11067	1.61	0.19	2.3	0.04	2.17	0.15	3.51	0	1.33	0.38	0.26	2	0.88	0
11076	-2.05	0.14	-2.89	0.01	-2.81	0.07	-4.46	0	-1.78	0.4	0.57	2	0.75	0
11083	-1.55	0.2	-2.84	0.02	-3.52	0.03	-4.71	0	-1.86	0.4	0.63	2	0.73	0
11096	1.03	0.4	1.45	0.2	3.01	0.06	3.35	0.01	1.23	0.37	1.06	2	0.59	0
11119	2.28	0.12	1.44	0.2	1.95	0.2	3.11	0.01	1.17	0.38	1.38	2	0.5	0
11189	-1.62	0.19	-2.65	0.02	-1.51	0.34	-3.21	0.01	-1.22	0.38	1.66	2	0.44	0
11190	-1.05	0.39	-3.07	0.01	-3.19	0.05	-4.2	0	-1.69	0.4	2.09	2	0.35	0
112464	0.74	0.62	2.13	0.06	2.71	0.08	3.38	0	1.26	0.37	0.99	2	0.61	0
11319	0.95	0.46	2.75	0.02	1.82	0.23	3.15	0.01	1.19	0.38	1.83	2	0.4	0
11326	2.9	0.1	2.69	0.02	2.53	0.1	3.73	0	1.9	0.51	2.74	2	0.25	0
11340	0.65	0.69	2.63	0.02	2.65	0.09	3.55	0	1.34	0.38	1.83	2	0.4	0
11341	-0.91	0.49	-2.54	0.03	-1.92	0.21	-3.12	0.01	-1.17	0.38	1.26	2	0.53	0
114625	-0.79	0.58	-2.68	0.02	-3.45	0.04	-4	0	-1.61	0.4	2.15	2	0.34	0
114818	-1.61	0.19	-1.77	0.11	-2.85	0.07	-3.69	0	-1.38	0.37	0.21	2	0.9	0
114908	2.02	0.14	2.48	0.03	2.98	0.06	4.37	0	1.71	0.39	0.05	2	0.97	0
1230	2.58	0.11	1.27	0.26	3.09	0.05	3.28	0.01	1.52	0.47	2.72	2	0.26	0
1266	2.22	0.13	1.87	0.1	3.57	0.03	4.5	0	1.75	0.39	0.93	2	0.63	0
1267	1.09	0.36	2.71	0.02	2.09	0.17	3.4	0	1.29	0.38	1.33	2	0.51	0
1272	-0.69	0.66	-2.77	0.02	-2.2	0.15	-3.23	0.01	-1.25	0.39	2.08	2	0.35	0
1282	1.62	0.19	2.64	0.02	2.71	0.08	4.08	0	1.58	0.39	0.34	2	0.84	0
1284	1.13	0.34	2.31	0.04	3	0.06	3.87	0	1.46	0.38	0.62	2	0.73	0
1293	2.55	0.12	2.5	0.03	2.44	0.11	4.16	0	1.65	0.4	1.38	2	0.5	0
1306	1.92	0.15	2.02	0.07	3.2	0.05	4.22	0	1.62	0.38	0.22	2	0.89	0
131408	-1.57	0.2	-1.67	0.14	-2.25	0.14	-3.2	0.01	-1.19	0.37	0.05	2	0.97	0
1381	-0.82	0.56	-2.22	0.05	-3.01	0.06	-3.68	0	-1.38	0.37	1.18	2	0.56	0
140578	-1.1	0.35	-2.28	0.04	-1.96	0.2	-3.12	0.01	-1.16	0.37	0.54	2	0.76	0
1428	-1.53	0.21	-1.27	0.26	-3.02	0.06	-3.47	0	-1.28	0.37	1.03	2	0.6	0
145389	2.2	0.13	1.63	0.14	2.31	0.13	3.47	0	1.31	0.38	0.84	2	0.66	0
1462	2.52	0.12	1.39	0.22	2.44	0.11	3.46	0	1.33	0.38	2.03	2	0.36	0
1471	2.03	0.14	3.01	0.01	1.69	0.28	3.19	0.01	1.53	0.48	2.7	2	0.26	0
1490	1.18	0.31	2.74	0.02	2.59	0.09	3.82	0	1.47	0.38	1.04	2	0.6	0
1499	1.34	0.25	2.78	0.02	1.82	0.23	3.36	0.01	1.29	0.38	1.56	2	0.46	0
1519	2.33	0.12	2.37	0.04	1.61	0.3	3.41	0	1.32	0.39	2	2	0.37	0
1536	2.87	0.1	1.8	0.11	3.18	0.05	3.65	0	1.77	0.49	2.72	2	0.26	0
1571	-1.11	0.35	-3.11	0.01	-2.9	0.07	-4.06	0	-1.63	0.4	2.08	2	0.35	0

1601	2.12	0.14	2.74	0.02	2.15	0.16	3.92	0	1.54	0.39	1.22	2	0.54	0
1634	1.57	0.2	2.34	0.04	1.85	0.23	3.28	0.01	1.24	0.38	0.58	2	0.75	0
1644	-1.96	0.15	-1.42	0.21	-2.14	0.16	-3.14	0.01	-1.16	0.37	0.61	2	0.74	0
1804	-1.59	0.19	-3.25	0.01	-4.17	0.02	-5.38	0	-2.22	0.41	1.53	2	0.47	0
1806	1.82	0.16	1.55	0.17	2.35	0.12	3.31	0.01	1.23	0.37	0.31	2	0.86	0
1896	-1.02	0.41	-2.94	0.01	-2.59	0.09	-3.82	0	-1.48	0.39	1.81	2	0.4	0
1901	1.77	0.17	1.02	0.37	3.41	0.04	3.35	0.01	1.36	0.41	2.33	2	0.31	0
1952	-2.58	0.11	-3.18	0.01	-2.75	0.08	-4.73	0	-1.97	0.42	1.93	2	0.38	0
1954	-1.67	0.18	-1.22	0.28	-3.54	0.03	-3.8	0	-1.44	0.38	2.04	2	0.36	0
196	2.04	0.14	3.1	0.01	2.02	0.18	3.68	0	1.61	0.44	2.3	2	0.32	0
199	1.73	0.17	1.9	0.09	1.99	0.19	3.22	0.01	1.2	0.37	0.19	2	0.91	0
2	1.92	0.15	3.4	0.01	3.54	0.03	5.15	0	2.14	0.41	1.25	2	0.54	0
2004	2.43	0.12	3.02	0.01	2.69	0.08	4.57	0	1.86	0.41	1.35	2	0.51	0
200424	-1.03	0.4	-2.77	0.02	-2.57	0.1	-3.74	0	-1.43	0.38	1.35	2	0.51	0
201134	1.71	0.17	1.48	0.19	3.49	0.03	3.99	0	1.5	0.38	1.38	2	0.5	0
202018	-1.07	0.38	-2.03	0.07	-3.56	0.03	-4.06	0	-1.53	0.38	1.43	2	0.49	0
2037	1.76	0.17	2.42	0.03	1.98	0.2	3.5	0	1.33	0.38	0.62	2	0.73	0
2040	1.96	0.15	2.63	0.02	2.16	0.16	3.82	0	1.48	0.39	0.85	2	0.66	0
2047	-1.62	0.19	-2.94	0.01	-2.38	0.12	-3.97	0	-1.55	0.39	1.2	2	0.55	0
2049	-1.32	0.26	-3.09	0.01	-3.09	0.05	-4.39	0	-1.74	0.4	1.53	2	0.46	0
205	-1.94	0.15	-2.62	0.02	-2.67	0.09	-4.18	0	-1.63	0.39	0.3	2	0.86	0
2050	-1.2	0.31	-1.95	0.08	-2.43	0.11	-3.31	0.01	-1.23	0.37	0.14	2	0.93	0
2059	1.6	0.19	1.88	0.09	2.62	0.09	3.6	0	1.35	0.37	0.04	2	0.98	0
2079	1.12	0.34	2.68	0.02	2.23	0.14	3.5	0	1.33	0.38	1.13	2	0.57	0
2150	-2.12	0.14	-2.8	0.02	-1.83	0.23	-3.71	0	-1.46	0.39	1.91	2	0.39	0
2176	-1.06	0.38	-2.92	0.01	-2.84	0.07	-4.02	0	-1.56	0.39	1.63	2	0.44	0
218	-1.17	0.32	-1.09	0.34	-3.15	0.05	-3.28	0.01	-1.2	0.37	1.72	2	0.42	0
2189	-0.88	0.51	-1.66	0.14	-2.63	0.09	-3.13	0.01	-1.15	0.37	0.59	2	0.74	0
2196	-1.64	0.18	-3.32	0.01	-2.06	0.18	-3.16	0.01	-1.64	0.52	3.05	2	0.22	0
2202	2.16	0.13	2.69	0.02	1.73	0.26	3.61	0	1.41	0.39	1.92	2	0.38	0
2207	2.86	0.1	2.82	0.02	2.17	0.15	3.26	0.01	1.9	0.58	3.44	2	0.18	0
2261	-1.74	0.17	-2.83	0.02	-2.02	0.18	-3.71	0	-1.44	0.39	1.35	2	0.51	0
22862	1.62	0.19	3.11	0.01	1.84	0.23	3.23	0.01	1.48	0.46	2.54	2	0.28	0
22877	-2.9	0.1	-2.56	0.02	-3.23	0.05	-4.86	0	-2	0.41	1.76	2	0.41	0
2288	-1.49	0.21	-1.75	0.12	-2.36	0.12	-3.28	0.01	-1.22	0.37	0.02	2	0.99	0
22893	-1.02	0.4	-2.57	0.02	-2.2	0.15	-3.39	0	-1.28	0.38	1.02	2	0.6	0
22916	1.69	0.18	1.56	0.16	2.87	0.07	3.62	0	1.35	0.37	0.45	2	0.8	0
22918	2.35	0.12	2.27	0.04	2.1	0.17	3.73	0	1.45	0.39	1.18	2	0.55	0
22919	1.34	0.25	2.55	0.03	2.52	0.1	3.76	0	1.43	0.38	0.51	2	0.78	0
22938	0.9	0.49	2.64	0.02	2.02	0.19	3.23	0.01	1.22	0.38	1.43	2	0.49	0
2299	-1.71	0.17	-2.56	0.02	-1.57	0.32	-3.25	0.01	-1.24	0.38	1.4	2	0.5	0
23071	2.18	0.13	2.13	0.06	1.46	0.36	3.13	0.01	1.19	0.38	1.63	2	0.44	0
23086	-1.13	0.34	-2.97	0.01	-1.88	0.22	-3.15	0.01	-1.32	0.42	2.29	2	0.32	0
23108	-0.88	0.51	-1.95	0.08	-2.38	0.12	-3.13	0.01	-1.15	0.37	0.45	2	0.8	0
23129	2	0.14	3.74	0	2.6	0.09	3.18	0.01	2.13	0.67	4.26	2	0.12	0
23166	2.32	0.12	2.47	0.03	2.04	0.18	3.78	0	1.47	0.39	1.33	2	0.51	0
23179	2.67	0.11	2.86	0.01	2.14	0.16	3.57	0	1.78	0.5	2.73	2	0.26	0
23180	2.12	0.14	2.61	0.02	2.15	0.16	3.86	0	1.51	0.39	1	2	0.61	0
23239	-1.56	0.2	-1.69	0.13	-2.35	0.12	-3.28	0.01	-1.22	0.37	0.05	2	0.97	0
23242	-1.8	0.16	-1.7	0.13	-2.4	0.12	-3.42	0	-1.28	0.37	0.18	2	0.91	0
23258	2.07	0.14	3.08	0.01	2.19	0.15	4.08	0	1.63	0.4	1.93	2	0.38	0
23263	-1	0.42	-2.77	0.02	-2.88	0.07	-3.95	0	-1.52	0.38	1.4	2	0.5	0
23286	-1.33	0.26	-3.4	0.01	-3.99	0.02	-4.35	0	-2.11	0.49	2.64	2	0.27	0
2332	2.07	0.14	2.22	0.05	1.65	0.29	3.28	0.01	1.25	0.38	1.17	2	0.56	0
23325	1.32	0.26	1.85	0.1	2.41	0.11	3.31	0.01	1.22	0.37	0.05	2	0.98	0
2335	1.84	0.16	2.11	0.06	1.89	0.22	3.31	0.01	1.25	0.38	0.47	2	0.79	0
23381	-1.84	0.16	-1.57	0.16	-2.4	0.12	-3.36	0.01	-1.25	0.37	0.31	2	0.85	0
23452	2.19	0.13	1.9	0.09	2.26	0.14	3.58	0	1.36	0.38	0.66	2	0.72	0
23471	1.7	0.17	2.77	0.02	1.61	0.3	3.37	0	1.3	0.39	1.85	2	0.4	0
23510	-1.84	0.16	-2.81	0.02	-1.89	0.22	-3.65	0	-1.42	0.39	1.54	2	0.46	0
23516	1.89	0.15	2.14	0.06	1.62	0.3	3.15	0.01	1.19	0.38	0.85	2	0.65	0
23545	-0.63	0.7	-2.75	0.02	-2.82	0.07	-3.5	0	-1.41	0.4	2.19	2	0.33	0
23586	1.4	0.24	1.92	0.09	2.49	0.1	3.44	0	1.28	0.37	0.03	2	0.98	0
23601	-1.21	0.3	-2.5	0.03	-2.52	0.1	-3.67	0	-1.39	0.38	0.57	2	0.75	0
23643	3.32	0.11	3.15	0.01	3.23	0.05	3.36	0	2.7	0.8	4.79	2	0.09	0
23760	2.2	0.13	3.11	0.01	1.77	0.25	3.15	0.01	1.66	0.53	3.09	2	0.21	0
24138	1.93	0.15	2.83	0.02	3.33	0.04	4.76	0	1.9	0.4	0.16	2	0.92	0
246	-1.21	0.3	-3.23	0.01	-2.46	0.11	-3.4	0	-1.57	0.46	2.6	2	0.27	0
2537	1.47	0.22	2.06	0.07	4.02	0.02	4.59	0	1.76	0.38	1.57	2	0.46	0
25758	-1.49	0.21	-2.63	0.02	-3.12	0.05	-4.3	0	-1.67	0.39	0.4	2	0.82	0
25778	-1.73	0.17	-1.32	0.24	-4.04	0.02	-3.37	0	-1.58	0.47	2.95	2	0.23	0

25801	1.94	0.15	1.74	0.12	2.61	0.09	3.65	0	1.37	0.38	0.29	2	0.86	0
25833	-1.25	0.28	-3.35	0.01	-2.75	0.08	-3.5	0	-1.71	0.49	2.79	2	0.25	0
259173	-1.95	0.15	-3.11	0.01	-3.06	0.06	-4.69	0	-1.89	0.4	0.78	2	0.68	0
25936	1.3	0.27	3.46	0.01	3.09	0.05	3.6	0	1.86	0.52	2.99	2	0.22	0
25937	2.15	0.13	2.56	0.02	2.25	0.14	3.92	0	1.53	0.39	0.84	2	0.66	0
25939	2.14	0.13	2.34	0.04	3	0.06	4.35	0	1.7	0.39	0.12	2	0.94	0
25970	-1.8	0.16	-2.86	0.01	-3.82	0.02	-5.06	0	-2.03	0.4	0.36	2	0.83	0
26001	-0.44	0.83	-2.26	0.04	-2.67	0.09	-3.27	0.01	-1.22	0.37	1.72	2	0.42	0
26013	-1.07	0.37	-3.3	0.01	-3.39	0.04	-3.71	0	-1.82	0.49	2.83	2	0.24	0
26015	-2.03	0.14	-2.55	0.03	-2.78	0.08	-4.27	0	-1.67	0.39	0.19	2	0.91	0
26017	1.93	0.15	2.41	0.03	2.04	0.18	3.61	0	1.38	0.38	0.67	2	0.72	0
26048	-1.22	0.3	-3.28	0.01	-4.32	0.02	-4.17	0	-2.13	0.51	2.96	2	0.23	0
26088	-0.99	0.43	-3.01	0.01	-4.21	0.02	-3.8	0	-1.93	0.51	3.1	2	0.21	0
26205	-1.52	0.21	-2.05	0.07	-2.35	0.12	-3.47	0	-1.3	0.37	0.02	2	0.99	0
26330	-1.39	0.24	-2.95	0.01	-2.63	0.09	-4.04	0	-1.58	0.39	1.21	2	0.54	0
2643	1.69	0.18	2.65	0.02	1.61	0.3	3.31	0.01	1.27	0.38	1.54	2	0.46	0
26520	0.78	0.59	2.42	0.03	2.32	0.13	3.28	0.01	1.23	0.37	1.1	2	0.58	0
26750	1.4	0.24	2.51	0.03	1.94	0.2	3.36	0.01	1.27	0.38	0.78	2	0.68	0
2694	-1.45	0.22	-2.96	0.01	-2.63	0.09	-4.08	0	-1.59	0.39	1.17	2	0.56	0
2697	1.45	0.22	3.04	0.01	2.1	0.17	3.72	0	1.45	0.39	1.96	2	0.38	0
27005	-2.75	0.11	-1.69	0.13	-3.02	0.06	-3.77	0	-1.65	0.44	2.36	2	0.31	0
27165	-1.45	0.22	-1.93	0.08	-2.88	0.07	-3.73	0	-1.4	0.37	0.18	2	0.92	0
27232	-2	0.14	-2.49	0.03	-1.64	0.29	-3.38	0	-1.3	0.38	1.44	2	0.49	0
27242	2.16	0.13	1.7	0.13	2.51	0.1	3.64	0	1.38	0.38	0.65	2	0.72	0
27292	0.86	0.52	1.88	0.09	2.78	0.08	3.36	0.01	1.24	0.37	0.75	2	0.69	0
2774	-2.2	0.13	-2.45	0.03	-3.61	0.03	-4.88	0	-1.94	0.4	0.12	2	0.94	0
2780	-2.1	0.14	-2.32	0.04	-2.08	0.17	-3.66	0	-1.4	0.38	0.75	2	0.69	0
2799	2.19	0.13	2.41	0.03	2.68	0.09	4.18	0	1.63	0.39	0.34	2	0.85	0
283459	-1.03	0.4	-1.57	0.16	-2.92	0.07	-3.35	0.01	-1.23	0.37	0.8	2	0.67	0
283638	-1.22	0.3	-3.21	0.01	-2.51	0.1	-3.56	0	-1.58	0.44	2.42	2	0.3	0
28965	-2.2	0.13	-2.2	0.05	-2.2	0.15	-3.71	0	-1.43	0.38	0.71	2	0.7	0
28977	0.94	0.46	2.15	0.06	2.93	0.06	3.64	0	1.36	0.37	0.81	2	0.67	0
2899	-1.6	0.19	-2.89	0.01	-1.55	0.32	-3.12	0.01	-1.31	0.42	2.29	2	0.32	0
2903	-1.79	0.16	-3.02	0.01	-2.14	0.16	-3.9	0	-1.54	0.39	1.73	2	0.42	0
29080	1.15	0.33	2.77	0.02	3.22	0.05	4.26	0	1.65	0.39	1.18	2	0.55	0
29091	-1.77	0.16	-2.28	0.04	-2.14	0.16	-3.55	0	-1.35	0.38	0.31	2	0.85	0
2959	1	0.42	2.78	0.02	2.33	0.13	3.56	0	1.36	0.38	1.51	2	0.47	0
2974	-1.41	0.23	-2.58	0.02	-1.75	0.26	-3.25	0.01	-1.23	0.38	1.13	2	0.57	0
29880	1.87	0.16	2.75	0.02	2.25	0.14	3.9	0	1.52	0.39	0.9	2	0.64	0
29887	2.55	0.12	1.88	0.09	2.55	0.1	3.88	0	1.5	0.39	1.46	2	0.48	0
2990	2.19	0.13	2.99	0.01	2.62	0.09	4.42	0	1.78	0.4	1.09	2	0.58	0
29915	0.9	0.49	2.36	0.04	2.28	0.13	3.28	0.01	1.23	0.37	0.8	2	0.67	0
29957	2.71	0.11	2.96	0.01	2.97	0.06	4.84	0	2	0.41	1.45	2	0.48	0
3001	1.93	0.15	2.44	0.03	1.78	0.24	3.44	0	1.32	0.38	1.05	2	0.59	0
3002	0.87	0.52	2.21	0.05	2.17	0.15	3.12	0.01	1.16	0.37	0.64	2	0.73	0
301	1.74	0.17	3.02	0.01	1.91	0.21	3.62	0	1.46	0.4	2.09	2	0.35	0
3010	-2.58	0.11	-2.73	0.02	-2.15	0.16	-3.89	0	-1.66	0.43	2.18	2	0.34	0
3059	1.85	0.16	1.95	0.08	1.91	0.21	3.24	0.01	1.22	0.37	0.38	2	0.83	0
308	1.84	0.16	3	0.01	2.45	0.11	4.14	0	1.64	0.4	1.22	2	0.54	0
30844	1.49	0.21	3.32	0.01	2.99	0.06	4.46	0	1.81	0.4	2.02	2	0.36	0
3091	2.23	0.13	2.26	0.04	1.52	0.33	3.26	0.01	1.25	0.38	1.77	2	0.41	0
3133	2.11	0.14	3.5	0.01	2.86	0.07	4.32	0	2.02	0.47	2.37	2	0.31	0
3169	-1.29	0.27	-2.07	0.07	-2.06	0.18	-3.17	0.01	-1.18	0.37	0.15	2	0.93	0
3200	-2.16	0.13	-2.01	0.07	-1.72	0.26	-3.25	0.01	-1.23	0.38	1.13	2	0.57	0
3294	-1.81	0.16	-2.46	0.03	-1.74	0.26	-3.37	0	-1.28	0.38	1.01	2	0.6	0
3352	-1.72	0.17	-2.39	0.03	-2.96	0.06	-4.18	0	-1.61	0.38	0.03	2	0.98	0
3371	2.56	0.11	1.63	0.15	2.28	0.14	3.54	0	1.35	0.38	1.9	2	0.39	0
3383	2.35	0.12	2.32	0.04	2.39	0.12	3.97	0	1.55	0.39	0.85	2	0.65	0
3384	2.52	0.12	1.53	0.17	2.94	0.06	3.93	0	1.51	0.38	1.85	2	0.4	0
3420	-1.93	0.15	-1.54	0.17	-2.4	0.12	-3.38	0	-1.26	0.37	0.46	2	0.8	0
3429	1.34	0.25	3.28	0.01	3.24	0.05	4.46	0	1.84	0.41	2.08	2	0.35	0
3431	2.25	0.13	3.61	0.01	2.77	0.08	3.79	0	2.16	0.57	3.13	2	0.21	0
3434	1.06	0.38	3.29	0.01	3.41	0.04	3.72	0	1.82	0.49	2.82	2	0.24	0
3437	1.46	0.22	2.39	0.03	3.36	0.04	4.33	0	1.66	0.38	0.41	2	0.82	0
3459	1.54	0.2	2.72	0.02	1.92	0.21	3.5	0	1.34	0.38	1.22	2	0.54	0
3479	2.64	0.11	3.19	0.01	3.15	0.05	5.07	0	2.13	0.42	1.36	2	0.51	0
3483	-1.43	0.23	-2.71	0.02	-1.7	0.27	-3.29	0.01	-1.25	0.38	1.51	2	0.47	0
3487	2.57	0.11	2.72	0.02	2.21	0.15	4.09	0	1.65	0.4	2.02	2	0.36	0
3490	1.59	0.19	3.29	0.01	2.69	0.08	4.27	0	1.73	0.4	2.03	2	0.36	0
3491	1.11	0.35	2.68	0.02	2.71	0.08	3.84	0	1.47	0.38	1	2	0.61	0
357	-1.13	0.34	-2.56	0.02	-1.76	0.25	-3.12	0.01	-1.18	0.38	1.21	2	0.55	0

3587	2.46	0.12	2.25	0.05	1.73	0.26	3.44	0	1.35	0.39	2.04	2	0.36	0
3613	-2.2	0.13	-1.15	0.31	-2.77	0.08	-3.49	0	-1.31	0.38	1.67	2	0.43	0
3617	-2.12	0.14	-1.86	0.1	-2.34	0.12	-3.6	0	-1.36	0.38	0.51	2	0.77	0
3620	1.98	0.14	2.2	0.05	2.06	0.18	3.53	0	1.34	0.38	0.52	2	0.77	0
3627	3.09	0.1	3.03	0.01	2.64	0.09	3.36	0.01	2.26	0.67	3.98	2	0.14	0
3643	-1.95	0.15	-2.29	0.04	-3.74	0.03	-4.77	0	-1.87	0.39	0.34	2	0.84	0
3670	-1.29	0.27	-3.01	0.01	-2.26	0.14	-3.74	0	-1.45	0.39	1.82	2	0.4	0
3671	2.48	0.12	2.41	0.03	1.99	0.19	3.75	0	1.47	0.39	1.79	2	0.41	0
3689	2.35	0.12	2.98	0.01	1.98	0.2	3.6	0	1.67	0.46	2.48	2	0.29	0
3737	-1.75	0.17	-2.7	0.02	-3.44	0.04	-4.69	0	-1.85	0.39	0.2	2	0.91	0
373863	-2.1	0.14	-3.62	0.01	-2.54	0.1	-3.39	0	-2.07	0.61	3.64	2	0.16	0
3769	-2.01	0.14	-2.47	0.03	-3.47	0.04	-4.71	0	-1.85	0.39	0.03	2	0.98	0
377	2.91	0.1	2.79	0.02	2.71	0.08	4.04	0	1.97	0.49	2.53	2	0.28	0
378	1.18	0.32	2.66	0.02	3.31	0.04	4.29	0	1.66	0.39	1.03	2	0.6	0
3781	-2.35	0.12	-1.96	0.08	-1.61	0.3	-3.2	0.01	-1.22	0.38	1.74	2	0.42	0
3801	-1.17	0.32	-2.52	0.03	-2.55	0.1	-3.69	0	-1.4	0.38	0.66	2	0.72	0
3816	-1.37	0.24	-2.76	0.02	-2.32	0.13	-3.72	0	-1.43	0.38	0.98	2	0.61	0
388650	1.83	0.16	3.01	0.01	2.16	0.16	3.92	0	1.55	0.39	1.67	2	0.43	0
390	1.95	0.15	2.15	0.06	2.09	0.17	3.52	0	1.33	0.38	0.43	2	0.81	0
3915	1.61	0.19	3.01	0.01	2.18	0.15	3.84	0	1.5	0.39	1.68	2	0.43	0
3954	-2.05	0.14	-1.96	0.08	-1.78	0.25	-3.23	0.01	-1.22	0.38	0.8	2	0.67	0
3956	2.27	0.13	2.25	0.05	2.15	0.16	3.73	0	1.44	0.39	0.93	2	0.63	0
397	2.62	0.11	3.49	0.01	2.71	0.08	3.83	0	2.23	0.58	3.17	2	0.21	0
3973	-1.68	0.18	-2.89	0.01	-1.73	0.26	-3.49	0	-1.35	0.39	1.97	2	0.37	0
3976	-1.36	0.25	-3.02	0.01	-3.28	0.04	-4.53	0	-1.79	0.4	1.24	2	0.54	0
3993	-1	0.42	-1.96	0.08	-2.78	0.08	-3.47	0	-1.29	0.37	0.55	2	0.76	0
399664	-1.12	0.34	-1.41	0.21	-2.82	0.07	-3.24	0.01	-1.19	0.37	0.73	2	0.69	0
4060	2.4	0.12	2.93	0.01	2.32	0.13	4.24	0	1.71	0.4	1.76	2	0.42	0
4067	2.93	0.11	2.75	0.02	2.28	0.14	3.29	0.01	1.93	0.59	3.42	2	0.18	0
4071	1.53	0.21	2.17	0.05	4.25	0.02	4.83	0	1.88	0.39	1.78	2	0.41	0
4117	-1.3	0.27	-3.03	0.01	-3.03	0.06	-4.32	0	-1.7	0.39	1.43	2	0.49	0
4121	2.23	0.13	1.89	0.09	1.82	0.23	3.28	0.01	1.24	0.38	1.13	2	0.57	0
4122	-1.85	0.16	-1.04	0.36	-3.21	0.05	-3.59	0	-1.34	0.37	1.93	2	0.38	0
4161	-1.4	0.24	-3.1	0.01	-2.73	0.08	-4.17	0	-1.65	0.39	1.59	2	0.45	0
4162	2.09	0.14	2.42	0.03	1.88	0.22	3.56	0	1.37	0.38	1.1	2	0.58	0
4175	2.01	0.14	3.13	0.01	2.15	0.16	3.91	0	1.63	0.42	2.13	2	0.34	0
4191	-1.57	0.2	-1.06	0.35	-3.06	0.06	-3.39	0	-1.25	0.37	1.5	2	0.47	0
4218	2.52	0.12	3.18	0.01	2.84	0.07	4.79	0	1.98	0.41	1.62	2	0.45	0
4222	1.42	0.23	1.84	0.1	2.27	0.14	3.25	0.01	1.2	0.37	0	2	1	0
4247	1.29	0.27	2.82	0.02	1.66	0.28	3.23	0.01	1.23	0.38	1.92	2	0.38	0
4253	-1.9	0.15	-3.13	0.01	-2.51	0.1	-4.26	0	-1.7	0.4	1.5	2	0.47	0
4283	2.88	0.1	2.29	0.04	2.2	0.15	3.21	0.01	1.68	0.53	3.03	2	0.22	0
4303	-2.34	0.12	-2.19	0.05	-2.08	0.17	-3.66	0	-1.41	0.39	1.15	2	0.56	0
4313	2.17	0.13	1.62	0.15	2.03	0.18	3.24	0.01	1.22	0.38	0.89	2	0.64	0
4332	2.25	0.13	2.65	0.02	1.89	0.22	3.73	0	1.46	0.39	1.72	2	0.42	0
4340	-1.48	0.21	-2.8	0.02	-2.22	0.14	-3.72	0	-1.43	0.39	1.08	2	0.58	0
4478	2.13	0.14	3.03	0.01	2.73	0.08	4.49	0	1.81	0.4	0.97	2	0.62	0
4499	1.75	0.17	2.74	0.02	1.97	0.2	3.64	0	1.41	0.39	1.22	2	0.54	0
4582	-1.25	0.29	-3.18	0.01	-2.23	0.14	-3.3	0.01	-1.5	0.46	2.56	2	0.28	0
4595	-1.8	0.16	-2.69	0.02	-2.51	0.1	-4.03	0	-1.57	0.39	0.5	2	0.78	0
4599	0.79	0.58	2.42	0.03	3.53	0.03	4.09	0	1.57	0.38	2.02	2	0.36	0
4602	-1.15	0.33	-3.02	0.01	-3.78	0.03	-4.76	0	-1.89	0.4	2.01	2	0.37	0
4610	-1.73	0.17	-2.54	0.03	-1.71	0.27	-3.35	0.01	-1.28	0.38	1.15	2	0.56	0
4615	2.43	0.12	2.65	0.02	2.4	0.12	4.17	0	1.66	0.4	1.24	2	0.54	0
474344	0.97	0.44	2.44	0.03	3.42	0.04	4.14	0	1.58	0.38	1.42	2	0.49	0
4756	-0.56	0.75	-2.74	0.02	-3.19	0.05	-3.3	0.01	-1.48	0.45	2.64	2	0.27	0
4837	3.49	0.1	3.22	0.01	4	0.02	3.43	0	3.15	0.92	5.42	2	0.07	0
488	-1.32	0.26	-1.2	0.29	-2.95	0.06	-3.28	0.01	-1.2	0.37	1.09	2	0.58	0
4899	-2.92	0.1	-2.87	0.01	-3.51	0.03	-5.26	0	-2.22	0.42	1.47	2	0.48	0
4907	1.52	0.21	1.66	0.14	3.22	0.05	3.83	0	1.43	0.37	0.71	2	0.7	0
4935	-1.49	0.21	-2.26	0.05	-2.5	0.1	-3.68	0	-1.39	0.38	0.1	2	0.95	0
5007	-1.56	0.2	-1.83	0.1	-3.73	0.03	-4.3	0	-1.63	0.38	1.23	2	0.54	0
50489	-1.71	0.17	-2.72	0.02	-2.42	0.11	-3.94	0	-1.53	0.39	0.63	2	0.73	0
50628	-0.67	0.68	-2.74	0.02	-2.26	0.14	-3.29	0.01	-1.26	0.38	2.03	2	0.36	0
50650	2.13	0.14	2.74	0.02	2.59	0.09	4.26	0	1.68	0.4	0.63	2	0.73	0
5071	-1.48	0.22	-1.5	0.18	-2.34	0.13	-3.13	0.01	-1.15	0.37	0.13	2	0.94	0
5077	-1.5	0.21	-2.63	0.02	-2.3	0.13	-3.72	0	-1.42	0.38	0.64	2	0.72	0
5080	-1.38	0.24	-2.28	0.04	-1.97	0.2	-3.25	0.01	-1.22	0.37	0.4	2	0.82	0
50805	-1.59	0.19	-1.56	0.16	-2.31	0.13	-3.2	0.01	-1.18	0.37	0.13	2	0.94	0
50810	2.47	0.12	1.19	0.29	3.3	0.04	3.29	0.01	1.54	0.47	2.77	2	0.25	0
51026	1.93	0.15	2.21	0.05	2.72	0.08	3.99	0	1.53	0.38	0.06	2	0.97	0

51056	1.44	0.23	3.75	0	3.15	0.05	3.16	0.01	2.08	0.66	4.32	2	0.12	0
51090	-1.17	0.32	-2.57	0.02	-2.03	0.18	-3.33	0.01	-1.26	0.38	0.96	2	0.62	0
5111	2.33	0.12	3.24	0.01	2.4	0.12	4.06	0	1.85	0.46	2.34	2	0.31	0
51177	1.88	0.15	3.08	0.01	2.03	0.18	3.76	0	1.55	0.41	2.12	2	0.35	0
51186	2.41	0.12	2.81	0.02	3.1	0.05	4.78	0	1.93	0.4	0.48	2	0.79	0
51195	-1.37	0.24	-1.41	0.21	-3.03	0.06	-3.49	0	-1.29	0.37	0.87	2	0.65	0
51206	-1.53	0.2	-2.73	0.02	-2	0.19	-3.56	0	-1.37	0.38	1.14	2	0.57	0
5126	-1.4	0.24	-3.19	0.01	-2.93	0.06	-4.35	0	-1.73	0.4	1.77	2	0.41	0
51279	2.03	0.14	1.27	0.26	2.66	0.09	3.43	0	1.28	0.37	1.06	2	0.59	0
51289	-1.26	0.28	-2.1	0.06	-2.09	0.17	-3.2	0.01	-1.19	0.37	0.18	2	0.91	0
51296	1.1	0.35	1.88	0.09	3.11	0.05	3.7	0	1.38	0.37	0.78	2	0.68	0
51338	1.56	0.2	2.75	0.02	2.74	0.08	4.12	0	1.6	0.39	0.53	2	0.77	0
51341	-1.34	0.25	-1.67	0.14	-2.59	0.09	-3.34	0.01	-1.23	0.37	0.17	2	0.92	0
51371	1.2	0.31	3.12	0.01	3.03	0.06	4.29	0	1.69	0.39	1.88	2	0.39	0
51388	2.04	0.14	1.17	0.3	2.51	0.1	3.27	0.01	1.22	0.37	1.15	2	0.56	0
51510	2.14	0.13	3.12	0.01	2.64	0.09	4.46	0	1.8	0.4	1.36	2	0.51	0
51569	2.47	0.12	3.21	0.01	2.05	0.18	3.3	0.01	1.88	0.57	3.33	2	0.19	0
51616	-1.44	0.23	-2.69	0.02	-1.81	0.24	-3.37	0	-1.28	0.38	1.31	2	0.52	0
51643	1.92	0.15	3.05	0.01	2.79	0.08	4.45	0	1.78	0.4	0.92	2	0.63	0
51651	1.17	0.32	2.97	0.01	2.88	0.07	4.11	0	1.6	0.39	1.52	2	0.47	0
5168	2.1	0.14	2.44	0.03	1.91	0.21	3.6	0	1.39	0.39	1.1	2	0.58	0
5175	1.77	0.17	3	0.01	3.77	0.03	5.08	0	2.05	0.4	0.52	2	0.77	0
5176	2.46	0.12	1.44	0.2	2.08	0.17	3.26	0.01	1.23	0.38	1.87	2	0.39	0
51760	-1.23	0.3	-1.72	0.12	-3.3	0.04	-3.78	0	-1.41	0.37	1.01	2	0.6	0
53	2.08	0.14	1.28	0.26	2.44	0.11	3.3	0.01	1.23	0.37	1.03	2	0.6	0
53335	-1.13	0.34	-3.43	0.01	-3.26	0.04	-3.47	0	-1.85	0.53	3.2	2	0.2	0
5352	1.91	0.15	1.02	0.37	2.8	0.07	3.33	0.01	1.23	0.37	1.48	2	0.48	0
5359	1.7	0.17	3.53	0.01	3.14	0.05	4.17	0	2	0.48	2.52	2	0.28	0
5360	2.61	0.11	3.16	0.01	2.74	0.08	4.72	0	1.96	0.42	1.98	2	0.37	0
53615	-1.76	0.17	-2.32	0.04	-1.67	0.28	-3.22	0.01	-1.22	0.38	0.86	2	0.65	0
5362	-0.76	0.61	-2.6	0.02	-2.85	0.07	-3.72	0	-1.41	0.38	1.61	2	0.45	0
53637	-1.67	0.18	-1.85	0.1	-2.07	0.18	-3.22	0.01	-1.2	0.37	0.1	2	0.95	0
53832	-1.3	0.27	-1.94	0.08	-2.17	0.15	-3.18	0.01	-1.18	0.37	0.05	2	0.98	0
53904	-1.8	0.16	-2.15	0.06	-1.76	0.25	-3.22	0.01	-1.21	0.38	0.59	2	0.74	0
540	-1.78	0.16	-3	0.01	-2.12	0.16	-3.87	0	-1.52	0.39	1.69	2	0.43	0
5426	-1.04	0.39	-1.84	0.1	-2.46	0.11	-3.2	0.01	-1.18	0.37	0.27	2	0.88	0
54361	-1.53	0.2	-1.76	0.12	-3.64	0.03	-4.19	0	-1.58	0.38	1.21	2	0.55	0
5440	0.72	0.64	2.78	0.02	3.85	0.02	3.42	0	1.69	0.49	3.09	2	0.21	0
54566	-1.24	0.29	-3.09	0.01	-3.01	0.06	-4.29	0	-1.69	0.39	1.7	2	0.43	0
5465	-1.84	0.16	-1.57	0.16	-3.1	0.05	-3.85	0	-1.45	0.38	0.71	2	0.7	0
547	-1.33	0.26	-3.3	0.01	-2.34	0.13	-3.27	0.01	-1.61	0.49	2.84	2	0.24	0
54784	-1.75	0.17	-1.81	0.11	-2.55	0.1	-3.57	0	-1.34	0.37	0.1	2	0.95	0
5479	2.57	0.11	3.74	0	3.28	0.04	4.23	0	2.51	0.59	3.06	2	0.22	0
54845	-2.04	0.14	-2.47	0.03	-2.08	0.17	-3.72	0	-1.43	0.39	0.81	2	0.67	0
54894	-1.29	0.27	-2.48	0.03	-2.49	0.1	-3.68	0	-1.4	0.38	0.46	2	0.79	0
54897	-2.57	0.11	-1.34	0.23	-2.43	0.11	-3.21	0.01	-1.34	0.42	2.3	2	0.32	0
54951	1.65	0.18	2.22	0.05	2.63	0.09	3.81	0	1.45	0.38	0.02	2	0.99	0
54961	-0.97	0.44	-2.81	0.02	-2.43	0.11	-3.62	0	-1.39	0.38	1.59	2	0.45	0
54964	-1.85	0.16	-2.09	0.06	-2.6	0.09	-3.81	0	-1.45	0.38	0.05	2	0.98	0
55012	2	0.14	2.81	0.02	1.82	0.23	3.66	0	1.43	0.39	1.8	2	0.41	0
55051	-1.26	0.28	-1.64	0.14	-2.65	0.09	-3.33	0.01	-1.23	0.37	0.27	2	0.87	0
55057	-1.54	0.2	-2.08	0.06	-3.6	0.03	-4.35	0	-1.66	0.38	0.7	2	0.7	0
55080	2.1	0.14	1.68	0.13	2.24	0.14	3.41	0	1.28	0.38	0.62	2	0.73	0
55090	-1.82	0.16	-2.01	0.07	-2.08	0.17	-3.38	0	-1.27	0.38	0.24	2	0.89	0
55095	-0.83	0.55	-2.64	0.02	-1.94	0.21	-3.13	0.01	-1.18	0.38	1.59	2	0.45	0
55117	-1.45	0.22	-3.03	0.01	-2.57	0.09	-4.06	0	-1.59	0.39	1.41	2	0.49	0
55227	-1.23	0.29	-2.32	0.04	-2.28	0.14	-3.42	0	-1.28	0.38	0.36	2	0.83	0
55233	1.83	0.16	2	0.07	3.53	0.03	4.39	0	1.68	0.38	0.52	2	0.77	0
55283	-0.77	0.6	-2.86	0.01	-2.57	0.09	-3.51	0	-1.4	0.4	2.13	2	0.35	0
55303	2.11	0.14	2.5	0.03	3.43	0.04	4.74	0	1.87	0.4	0.02	2	0.99	0
55323	1.82	0.16	1.78	0.11	2.11	0.17	3.27	0.01	1.22	0.37	0.22	2	0.9	0
55367	-1.25	0.28	-2.82	0.02	-1.66	0.29	-3.21	0.01	-1.23	0.38	1.96	2	0.37	0
55374	-0.84	0.54	-1.89	0.09	-2.76	0.08	-3.34	0.01	-1.23	0.37	0.77	2	0.68	0
5547	2.5	0.12	2.44	0.03	2.59	0.09	4.23	0	1.67	0.4	1.02	2	0.6	0
5552	2.73	0.11	3.46	0.01	2.55	0.1	3.5	0	2.27	0.65	3.77	2	0.15	0
55601	1.98	0.14	2.44	0.03	2.79	0.08	4.19	0	1.63	0.39	0.1	2	0.95	0
55616	-0.9	0.5	-2.62	0.02	-2.68	0.09	-3.68	0	-1.4	0.38	1.28	2	0.53	0
55670	-0.3	0.9	-2.15	0.06	-2.66	0.09	-3.14	0.01	-1.16	0.37	1.93	2	0.38	0
55748	2.4	0.12	2.17	0.05	2.21	0.15	3.77	0	1.46	0.39	1.14	2	0.57	0
55765	-1.71	0.17	-3.4	0.01	-3.51	0.03	-5.02	0	-2.07	0.41	1.62	2	0.44	0
55790	1.58	0.2	2.92	0.01	2.1	0.17	3.73	0	1.45	0.39	1.5	2	0.47	0

55803	2.65	0.11	2.84	0.02	2.35	0.12	4.05	0	1.77	0.44	2.22	2	0.33	0
55806	-1.34	0.25	-2.92	0.01	-2.24	0.14	-3.72	0	-1.44	0.39	1.53	2	0.47	0
55830	1.52	0.21	2.75	0.02	1.48	0.35	3.18	0.01	1.22	0.38	1.96	2	0.37	0
5610	1.12	0.34	1.82	0.1	3.08	0.05	3.65	0	1.36	0.37	0.75	2	0.69	0
56165	-2	0.14	-2.33	0.04	-2.54	0.1	-3.96	0	-1.52	0.39	0.2	2	0.9	0
5641	2.62	0.11	3.59	0.01	3.18	0.05	4.54	0	2.37	0.52	2.57	2	0.28	0
5682	0.99	0.43	2.31	0.04	2.62	0.09	3.54	0	1.33	0.38	0.66	2	0.72	0
56944	2.11	0.14	2.01	0.07	1.81	0.24	3.3	0.01	1.25	0.38	0.9	2	0.64	0
56957	-3.08	0.1	-2.2	0.05	-3.29	0.04	-3.67	0	-2.02	0.55	3.11	2	0.21	0
56980	-2.31	0.12	-1.51	0.18	-3.03	0.06	-3.93	0	-1.5	0.38	1.36	2	0.51	0
56998	-2.12	0.14	-1.99	0.08	-1.93	0.21	-3.38	0	-1.28	0.38	0.78	2	0.68	0
57102	1.33	0.26	1.79	0.11	2.28	0.13	3.19	0.01	1.18	0.37	0.02	2	0.99	0
57122	1.86	0.16	2.06	0.07	3.62	0.03	4.5	0	1.74	0.39	0.53	2	0.77	0
57144	-1.34	0.25	-2.51	0.03	-1.98	0.2	-3.35	0.01	-1.27	0.38	0.77	2	0.68	0
5716	1.09	0.36	2.39	0.04	2.68	0.08	3.67	0	1.38	0.38	0.61	2	0.74	0
5725	2.07	0.14	3.23	0.01	1.89	0.21	3.2	0.01	1.69	0.53	3.09	2	0.21	0
5753	-1.63	0.19	-2.2	0.05	-2.61	0.09	-3.78	0	-1.43	0.38	0.02	2	0.99	0
57604	-2.46	0.12	-3.02	0.01	-2.25	0.14	-4.01	0	-1.76	0.44	2.23	2	0.33	0
57715	-1.44	0.23	-2.47	0.03	-1.79	0.24	-3.24	0.01	-1.22	0.38	0.84	2	0.66	0
57718	-1.37	0.24	-3.38	0.01	-2.37	0.12	-3.19	0.01	-1.66	0.52	3.1	2	0.21	0
57835	-1.5	0.21	-2.87	0.01	-3.19	0.05	-4.47	0	-1.76	0.39	0.7	2	0.71	0
5908	1.7	0.17	3.55	0.01	2.42	0.11	3.17	0.01	1.86	0.58	3.6	2	0.17	0
5909	-1.4	0.24	-2.21	0.05	-2.13	0.16	-3.35	0.01	-1.25	0.37	0.21	2	0.9	0
5920	2.57	0.11	3.71	0	2.75	0.08	3.47	0	2.41	0.69	4.11	2	0.13	0
59340	-1.69	0.18	-3.34	0.01	-2.37	0.12	-3.62	0	-1.71	0.47	2.58	2	0.28	0
59342	2.4	0.12	2.7	0.02	2.5	0.1	4.27	0	1.7	0.4	1.08	2	0.58	0
5965	1.81	0.16	2.39	0.04	2.53	0.1	3.9	0	1.5	0.38	0.14	2	0.93	0
5976	-0.54	0.76	-1.52	0.18	-2.98	0.06	-3.13	0.01	-1.14	0.37	1.73	2	0.42	0
598	2.27	0.13	1.19	0.29	3.41	0.04	3.52	0	1.51	0.43	2.45	2	0.29	0
60	1.31	0.27	2.86	0.01	2.16	0.15	3.62	0	1.4	0.39	1.44	2	0.49	0
6002	-1.33	0.26	-2.31	0.04	-2.38	0.12	-3.54	0	-1.33	0.38	0.26	2	0.88	0
6035	2.54	0.12	3.36	0.01	2.92	0.07	4.82	0	2.08	0.43	2.07	2	0.36	0
60485	1.13	0.34	2.85	0.02	2.18	0.15	3.54	0	1.36	0.38	1.59	2	0.45	0
60559	2.38	0.12	2.93	0.01	4.11	0.02	5.58	0	2.32	0.42	0.01	2	1	0
607	-2.25	0.13	-3.15	0.01	-2.33	0.13	-4.21	0	-1.74	0.41	2.06	2	0.36	0
6097	-2.43	0.12	-2.95	0.01	-2.5	0.1	-4.39	0	-1.78	0.4	1.54	2	0.46	0
6184	2.2	0.13	2.81	0.02	3.07	0.06	4.68	0	1.88	0.4	0.29	2	0.87	0
6239	-1.24	0.29	-2.1	0.06	-2.88	0.07	-3.73	0	-1.4	0.38	0.33	2	0.85	0
6257	-1.93	0.15	-3.18	0.01	-1.85	0.23	-3.2	0.01	-1.61	0.5	2.89	2	0.24	0
6297	-1.32	0.26	-3.18	0.01	-2.05	0.18	-3.16	0.01	-1.48	0.47	2.68	2	0.26	0
6303	2.35	0.12	3.19	0.01	2.65	0.09	4.58	0	1.88	0.41	1.73	2	0.42	0
6352	2.53	0.12	2.85	0.02	2.26	0.14	4.14	0	1.7	0.41	2.05	2	0.36	0
6355	2.22	0.13	2.67	0.02	2.9	0.07	4.49	0	1.78	0.4	0.32	2	0.85	0
6373	3.05	0.11	2.11	0.06	2.65	0.09	3.16	0.01	1.83	0.58	3.49	2	0.17	0
63827	-1.77	0.17	-3.56	0.01	-2.36	0.12	-3.14	0.01	-1.87	0.6	3.71	2	0.16	0
6387	2.31	0.12	3.32	0.01	2.52	0.1	4.13	0	1.92	0.46	2.37	2	0.31	0
63876	-1.4	0.24	-2.79	0.02	-2.17	0.15	-3.64	0	-1.4	0.38	1.16	2	0.56	0
63893	-1.76	0.17	-2.98	0.01	-3.05	0.06	-4.54	0	-1.81	0.4	0.65	2	0.72	0
63925	-1.1	0.35	-2.23	0.05	-2.93	0.06	-3.76	0	-1.42	0.38	0.58	2	0.75	0
6405	-0.81	0.57	-2.38	0.04	-2.91	0.07	-3.68	0	-1.39	0.38	1.22	2	0.54	0
64092	2.07	0.14	2.16	0.06	1.84	0.23	3.38	0	1.29	0.38	0.88	2	0.65	0
64123	1.39	0.24	3.59	0.01	3.84	0.02	3.9	0	2.16	0.55	3.22	2	0.2	0
64135	1.66	0.18	2.62	0.02	2.6	0.09	4.01	0	1.55	0.39	0.35	2	0.84	0
64284	-2.03	0.14	-2.51	0.03	-2.22	0.14	-3.83	0	-1.48	0.39	0.67	2	0.72	0
64343	1.06	0.38	1.96	0.08	2.28	0.13	3.15	0.01	1.16	0.37	0.21	2	0.9	0
64375	-2.48	0.12	-2.75	0.02	-1.87	0.22	-3.46	0	-1.59	0.46	2.49	2	0.29	0
64429	1.05	0.39	2.1	0.06	2.17	0.15	3.14	0.01	1.16	0.37	0.32	2	0.85	0
6464	0.98	0.43	2.57	0.02	3.11	0.05	4	0	1.53	0.38	1.21	2	0.55	0
64763	-1.53	0.2	-1.89	0.09	-2.6	0.09	-3.56	0	-1.33	0.37	0.03	2	0.98	0
64793	-1.36	0.25	-2.58	0.02	-2.04	0.18	-3.44	0	-1.3	0.38	0.84	2	0.66	0
64925	-1.85	0.16	-3.53	0.01	-2.95	0.06	-4.14	0	-1.99	0.48	2.51	2	0.29	0
6507	2.25	0.13	1.71	0.13	2.21	0.15	3.46	0	1.31	0.38	0.93	2	0.63	0
6509	-1.86	0.15	-2.16	0.05	-2.82	0.07	-4.01	0	-1.53	0.38	0.02	2	0.99	0
65095	-1.15	0.33	-1.53	0.17	-3.02	0.06	-3.45	0	-1.27	0.37	0.87	2	0.65	0
653483	-1.77	0.17	-2.52	0.03	-2.54	0.1	-3.96	0	-1.53	0.39	0.24	2	0.89	0
6545	-1.48	0.21	-3.05	0.01	-1.96	0.2	-3.46	0	-1.43	0.41	2.19	2	0.33	0
658	-2.45	0.12	-2.96	0.01	-2.98	0.06	-4.77	0	-1.95	0.41	0.84	2	0.66	0
65989	-1.71	0.17	-2.91	0.01	-2.36	0.12	-3.98	0	-1.56	0.39	1.12	2	0.57	0
66002	-1.51	0.21	-3.22	0.01	-2.68	0.08	-4.24	0	-1.69	0.4	1.89	2	0.39	0
6608	-2.02	0.14	-3.51	0.01	-3.09	0.05	-4.74	0	-2.04	0.43	2.1	2	0.35	0
6614	1.3	0.27	2.96	0.01	3.6	0.03	4.7	0	1.86	0.4	1.37	2	0.5	0

6624	2.74	0.11	1.65	0.14	2.61	0.09	3.42	0	1.52	0.44	2.45	2	0.29	0
6663	-0.97	0.44	-3.15	0.01	-3.63	0.03	-3.8	0	-1.82	0.48	2.76	2	0.25	0
6665	-1.48	0.21	-2.99	0.01	-1.84	0.23	-3.42	0	-1.38	0.4	2.12	2	0.35	0
6672	2.27	0.13	2.9	0.01	2.54	0.1	4.34	0	1.74	0.4	1.1	2	0.58	0
6695	0.74	0.62	2	0.08	2.49	0.1	3.16	0.01	1.17	0.37	0.74	2	0.69	0
6712	-1.2	0.31	-2.16	0.05	-2.45	0.11	-3.45	0	-1.29	0.37	0.24	2	0.89	0
6717	1.47	0.22	3.2	0.01	2.16	0.16	3.44	0	1.54	0.45	2.45	2	0.29	0
6737	2.15	0.13	2.29	0.04	2	0.19	3.6	0	1.38	0.38	0.9	2	0.64	0
6741	1.08	0.36	3.47	0.01	3.57	0.03	3.43	0	1.94	0.57	3.55	2	0.17	0
6770	-0.82	0.56	-2.37	0.04	-2.76	0.08	-3.58	0	-1.34	0.38	1.09	2	0.58	0
6772	2.09	0.14	2.83	0.02	2.83	0.07	4.46	0	1.78	0.4	0.47	2	0.79	0
6782	2.37	0.12	1.23	0.28	2.84	0.07	3.64	0	1.38	0.38	1.97	2	0.37	0
684	2.2	0.13	3.62	0.01	3.49	0.03	5.31	0	2.27	0.43	1.96	2	0.38	0
6868	1.98	0.14	1.44	0.2	3.03	0.06	3.77	0	1.42	0.38	0.97	2	0.62	0
6897	1.29	0.27	2.66	0.02	1.81	0.24	3.27	0.01	1.24	0.38	1.28	2	0.53	0
6925	1.79	0.16	3.02	0.01	1.72	0.27	3.24	0.01	1.46	0.45	2.48	2	0.29	0
6990	1.11	0.35	3.18	0.01	2.55	0.1	3.5	0	1.56	0.44	2.46	2	0.29	0
7018	-1.26	0.28	-2.73	0.02	-1.7	0.27	-3.21	0.01	-1.22	0.38	1.62	2	0.44	0
7020	-1.68	0.18	-3.55	0.01	-2.59	0.09	-3.36	0.01	-1.89	0.56	3.33	2	0.19	0
7021	-1.28	0.27	-2.99	0.01	-2.31	0.13	-3.77	0	-1.46	0.39	1.74	2	0.42	0
7026	0.53	0.78	2.67	0.02	2.7	0.08	3.29	0.01	1.33	0.41	2.24	2	0.33	0
7035	1.94	0.15	1.62	0.15	3.44	0.04	4.14	0	1.57	0.38	1.07	2	0.59	0
7048	1.8	0.16	3.47	0.01	2.44	0.11	3.46	0	1.84	0.53	3.05	2	0.22	0
7058	2.13	0.14	0.98	0.39	2.47	0.11	3.15	0.01	1.17	0.37	1.66	2	0.44	0
7091	1.97	0.15	2.27	0.04	1.71	0.27	3.31	0.01	1.26	0.38	0.98	2	0.61	0
7097	2.44	0.12	1.63	0.15	2	0.19	3.31	0.01	1.26	0.38	1.66	2	0.44	0
710	1.84	0.16	3.24	0.01	3.41	0.04	4.95	0	2.02	0.41	0.96	2	0.62	0
712	2.63	0.11	3.94	0	3.46	0.04	3.82	0	2.8	0.73	4.08	2	0.13	0
7130	2.47	0.12	1.14	0.31	3.26	0.04	3.18	0.01	1.52	0.48	2.88	2	0.24	0
715	2.26	0.13	3.75	0	3.29	0.04	4.18	0	2.37	0.57	2.98	2	0.23	0
717	1.73	0.17	2.19	0.05	3.08	0.05	4.15	0	1.59	0.38	0.06	2	0.97	0
7180	-1.93	0.15	-3.07	0.01	-1.86	0.22	-3.42	0	-1.54	0.45	2.45	2	0.29	0
7200	-2.2	0.13	-3.35	0.01	-3.94	0.02	-5.57	0	-2.36	0.42	0.53	2	0.77	0
7299	-1.87	0.15	-3.17	0.01	-1.96	0.2	-3.4	0	-1.6	0.47	2.59	2	0.27	0
7347	1.13	0.34	2.97	0.01	2.24	0.14	3.63	0	1.4	0.39	1.91	2	0.38	0
7358	2.01	0.14	2.16	0.06	1.79	0.24	3.32	0.01	1.26	0.38	0.83	2	0.66	0
7368	-1.8	0.16	-2.88	0.01	-2.05	0.18	-3.78	0	-1.47	0.39	1.45	2	0.48	0
7412	2.88	0.1	2.83	0.02	2.29	0.13	3.4	0	1.93	0.57	3.26	2	0.2	0
7433	-1.91	0.15	-3.3	0.01	-2.6	0.09	-4.39	0	-1.78	0.41	2	2	0.37	0
7444	0.92	0.48	2.41	0.03	2.02	0.19	3.13	0.01	1.17	0.37	0.92	2	0.63	0
7453	2.36	0.12	2.58	0.02	2.26	0.14	4.01	0	1.58	0.39	1.22	2	0.54	0
7456	2.87	0.1	2.82	0.02	2.13	0.16	3.2	0.01	1.9	0.59	3.53	2	0.17	0
7477	-1.05	0.39	-2.65	0.02	-2.42	0.11	-3.59	0	-1.37	0.38	1.08	2	0.58	0
7480	-1.33	0.26	-2.44	0.03	-2.33	0.13	-3.56	0	-1.35	0.38	0.43	2	0.81	0
7482	-1.37	0.24	-1.17	0.3	-2.72	0.08	-3.14	0.01	-1.15	0.37	0.8	2	0.67	0
7625	-1.1	0.35	-2.69	0.02	-2.75	0.08	-3.87	0	-1.48	0.38	1.04	2	0.6	0
7629	-2.72	0.11	-1.58	0.16	-2.82	0.07	-3.5	0	-1.56	0.44	2.46	2	0.29	0
765	-1.44	0.23	-2.66	0.02	-1.72	0.26	-3.28	0.01	-1.25	0.38	1.34	2	0.51	0
7691	-1.73	0.17	-2.23	0.05	-2.03	0.18	-3.43	0	-1.3	0.38	0.34	2	0.84	0
776	-1.47	0.22	-2.94	0.01	-1.81	0.24	-3.47	0	-1.34	0.39	2	2	0.37	0
7791	2.39	0.12	1.78	0.11	2.71	0.08	3.9	0	1.5	0.38	1.03	2	0.6	0
783	-2.13	0.14	-2.64	0.02	-2.49	0.1	-4.14	0	-1.62	0.39	0.61	2	0.74	0
79047	-1.02	0.41	-1.66	0.14	-2.62	0.09	-3.2	0.01	-1.18	0.37	0.43	2	0.81	0
79139	1.32	0.26	2.67	0.02	2.65	0.09	3.91	0	1.5	0.38	0.69	2	0.71	0
79155	0.54	0.77	2.2	0.05	2.85	0.07	3.41	0	1.27	0.37	1.6	2	0.45	0
79169	-2.09	0.14	-1.42	0.21	-2.14	0.16	-3.18	0.01	-1.19	0.37	0.85	2	0.65	0
79616	-1.78	0.16	-2.71	0.02	-1.55	0.32	-3.33	0.01	-1.28	0.38	1.85	2	0.4	0
79628	-1.49	0.21	-2.9	0.01	-2.42	0.11	-3.92	0	-1.52	0.39	1.15	2	0.56	0
79667	-1.63	0.19	-2.29	0.04	-2.29	0.13	-3.6	0	-1.37	0.38	0.18	2	0.92	0
79842	-1.02	0.41	-1.19	0.29	-3.16	0.05	-3.28	0.01	-1.2	0.37	1.71	2	0.43	0
79868	2.53	0.12	2.22	0.05	1.98	0.2	3.66	0	1.43	0.39	1.87	2	0.39	0
79874	-1.79	0.16	-2.27	0.04	-2.55	0.1	-3.84	0	-1.47	0.38	0.06	2	0.97	0
79875	-1.87	0.15	-2.66	0.02	-1.65	0.29	-3.42	0	-1.32	0.39	1.62	2	0.44	0
79879	-0.37	0.87	-2.05	0.07	-2.89	0.07	-3.28	0.01	-1.21	0.37	1.95	2	0.38	0
79885	-2.93	0.11	-3.8	0	-2.8	0.07	-3.15	0.01	-2.79	0.89	5.66	2	0.06	0
79943	-1.52	0.21	-3.36	0.01	-2.4	0.12	-3.43	0	-1.69	0.49	2.8	2	0.25	0
79962	-1.22	0.3	-2.2	0.05	-3.13	0.05	-3.94	0	-1.49	0.38	0.55	2	0.76	0
79977	-1.64	0.18	-1.97	0.08	-1.9	0.21	-3.16	0.01	-1.18	0.37	0.21	2	0.9	0
79979	-2.22	0.13	-2.43	0.03	-3.78	0.03	-4.99	0	-1.99	0.4	0.22	2	0.9	0
800	1.93	0.15	3.19	0.01	1.86	0.22	3.21	0.01	1.61	0.5	2.89	2	0.24	0
80013	1.84	0.16	1.71	0.13	2.43	0.11	3.46	0	1.29	0.37	0.21	2	0.9	0

80071	-0.55	0.76	-1.93	0.08	-2.96	0.06	-3.35	0.01	-1.24	0.37	1.58	2	0.45	0
80094	-0.99	0.43	-3.47	0.01	-3.78	0.03	-3.3	0.01	-1.97	0.6	3.94	2	0.14	0
80095	-2.06	0.14	-1.94	0.08	-2.15	0.16	-3.48	0	-1.32	0.38	0.48	2	0.79	0
80131	-1.02	0.41	-2.96	0.01	-2.64	0.09	-3.86	0	-1.5	0.39	1.87	2	0.39	0
80219	1.39	0.24	2.69	0.02	1.99	0.19	3.46	0	1.32	0.38	1.1	2	0.58	0
8045	-1.86	0.16	-3	0.01	-1.96	0.2	-3.78	0	-1.49	0.39	1.99	2	0.37	0
8076	1.39	0.24	2.29	0.04	1.91	0.21	3.23	0.01	1.21	0.37	0.45	2	0.8	0
80833	1.76	0.17	2.37	0.04	3.03	0.06	4.23	0	1.63	0.39	0.02	2	0.99	0
8085	-1.91	0.15	-2.76	0.02	-2.75	0.08	-4.29	0	-1.69	0.39	0.4	2	0.82	0
8099	2.28	0.13	2.8	0.02	2.35	0.12	4.16	0	1.66	0.4	1.26	2	0.53	0
81029	-1.01	0.41	-2.3	0.04	-2.17	0.15	-3.23	0.01	-1.21	0.37	0.58	2	0.75	0
81542	1.89	0.15	1.61	0.15	3.06	0.06	3.86	0	1.45	0.38	0.64	2	0.73	0
81553	2.53	0.12	1.68	0.13	2.9	0.07	4	0	1.55	0.39	1.57	2	0.46	0
81606	1.13	0.34	3.05	0.01	3.11	0.05	4.29	0	1.69	0.39	1.8	2	0.41	0
81614	1.7	0.17	1.87	0.09	2.48	0.11	3.53	0	1.32	0.37	0.04	2	0.98	0
81628	-1.24	0.29	-1.35	0.23	-3.15	0.05	-3.47	0	-1.28	0.37	1.24	2	0.54	0
81693	-1.57	0.2	-2.51	0.03	-2.05	0.18	-3.51	0	-1.33	0.38	0.63	2	0.73	0
818	-2.72	0.11	-1.48	0.19	-3.34	0.04	-3.45	0	-1.69	0.49	2.88	2	0.24	0
81853	0.95	0.46	2.52	0.03	2.03	0.18	3.2	0.01	1.2	0.38	1.09	2	0.58	0
81894	1.82	0.16	2.86	0.01	2.03	0.18	3.77	0	1.47	0.39	1.45	2	0.48	0
824	2.23	0.13	2.15	0.06	1.82	0.24	3.41	0	1.31	0.38	1.21	2	0.55	0
826	1.67	0.18	3.12	0.01	1.98	0.2	3.48	0	1.51	0.43	2.33	2	0.31	0
8291	1.06	0.38	2.23	0.05	3.2	0.05	3.93	0	1.48	0.38	0.89	2	0.64	0
83447	-1.99	0.14	-2.77	0.02	-1.8	0.24	-3.62	0	-1.41	0.39	1.72	2	0.42	0
83637	-0.84	0.54	-2.63	0.02	-3.9	0.02	-3.79	0	-1.71	0.45	2.62	2	0.27	0
840	1.46	0.22	2.69	0.02	2.57	0.09	3.92	0	1.51	0.39	0.6	2	0.74	0
8408	-2.24	0.13	-1.18	0.3	-2.32	0.13	-3.2	0.01	-1.2	0.37	1.51	2	0.47	0
84302	-2.03	0.14	-3.46	0.01	-2.37	0.12	-3.49	0	-1.89	0.54	3.1	2	0.21	0
8471	-2.82	0.1	-1.61	0.15	-3.21	0.05	-3.44	0	-1.72	0.5	2.91	2	0.23	0
84719	-0.79	0.58	-2.78	0.02	-3.67	0.03	-3.75	0	-1.68	0.45	2.57	2	0.28	0
84722	-1.41	0.23	-2.29	0.04	-2.41	0.12	-3.58	0	-1.35	0.38	0.18	2	0.91	0
8492	-1.71	0.17	-2.98	0.01	-2.12	0.16	-3.82	0	-1.5	0.39	1.63	2	0.44	0
8503	2.93	0.11	2.21	0.05	2.9	0.07	3.89	0	1.83	0.47	2.5	2	0.29	0
8519	1.48	0.22	3.73	0	3.94	0.02	3.76	0	2.29	0.61	3.67	2	0.16	0
8541	-1.17	0.32	-2.91	0.01	-2.12	0.16	-3.54	0	-1.36	0.38	1.78	2	0.41	0
8542	1.73	0.17	2.08	0.06	2.17	0.15	3.44	0	1.3	0.38	0.15	2	0.93	0
8565	2.45	0.12	1.98	0.08	1.99	0.19	3.51	0	1.35	0.38	1.55	2	0.46	0
857	1.39	0.24	3.18	0.01	2.62	0.09	4.12	0	1.63	0.4	1.97	2	0.37	0
858	1.57	0.2	2.6	0.02	3.39	0.04	4.51	0	1.76	0.39	0.35	2	0.84	0
8605	1.47	0.22	2.83	0.02	1.8	0.24	3.43	0	1.32	0.38	1.7	2	0.43	0
8607	1.13	0.34	2.06	0.07	2.23	0.14	3.2	0.01	1.19	0.37	0.21	2	0.9	0
8624	0.53	0.78	1.8	0.11	3.03	0.06	3.31	0.01	1.22	0.37	1.74	2	0.42	0
8697	1.45	0.22	2.35	0.04	1.69	0.28	3.12	0.01	1.17	0.38	0.74	2	0.69	0
8704	-2.5	0.12	-2.18	0.05	-2.99	0.06	-4.38	0	-1.72	0.39	0.81	2	0.67	0
8743	1.99	0.14	2.87	0.01	2.73	0.08	4.36	0	1.73	0.4	0.6	2	0.74	0
8754	1.04	0.39	2.75	0.02	2.23	0.14	3.49	0	1.33	0.38	1.42	2	0.49	0
8775	0.81	0.57	2.53	0.03	2.37	0.12	3.38	0	1.27	0.38	1.26	2	0.53	0
8795	2.41	0.12	1.29	0.25	2.76	0.08	3.63	0	1.38	0.38	1.91	2	0.39	0
8813	0.85	0.54	2.47	0.03	3.27	0.04	4	0	1.52	0.38	1.56	2	0.46	0
883	-1.31	0.26	-2.96	0.01	-2.87	0.07	-4.18	0	-1.63	0.39	1.24	2	0.54	0
8910	2.38	0.12	3.23	0.01	1.96	0.2	3.21	0.01	1.84	0.57	3.4	2	0.18	0
8934	1.83	0.16	2.89	0.01	1.91	0.21	3.69	0	1.44	0.39	1.73	2	0.42	0
894	2.55	0.12	1.85	0.1	2.27	0.14	3.67	0	1.41	0.39	1.66	2	0.44	0
90	0.99	0.43	2.84	0.02	2.55	0.1	3.74	0	1.43	0.38	1.6	2	0.45	0
9013	-0.96	0.45	-1.97	0.08	-2.56	0.1	-3.3	0.01	-1.22	0.37	0.44	2	0.8	0
902	0.84	0.54	1.95	0.08	2.9	0.07	3.46	0	1.28	0.37	0.93	2	0.63	0
9039	0.98	0.44	3.27	0.01	2.98	0.06	3.35	0.01	1.68	0.5	2.98	2	0.23	0
9056	2.95	0.11	2.2	0.05	2.87	0.07	3.76	0	1.83	0.49	2.64	2	0.27	0
909	-2.71	0.11	-3.04	0.01	-2.57	0.09	-4.19	0	-1.95	0.46	2.35	2	0.31	0
910	-1.65	0.18	-3.57	0.01	-2.92	0.07	-3.65	0	-1.96	0.54	3.06	2	0.22	0
9111	2.69	0.11	3.43	0.01	2.44	0.11	3.43	0	2.21	0.64	3.78	2	0.15	0
913	-1.24	0.29	-1.35	0.23	-3.43	0.04	-3.65	0	-1.35	0.37	1.77	2	0.41	0
9145	-1.74	0.17	-1.59	0.16	-2.88	0.07	-3.66	0	-1.37	0.37	0.43	2	0.8	0
9153	-1.27	0.28	-2.72	0.02	-2.27	0.14	-3.62	0	-1.38	0.38	1.02	2	0.6	0
91543	1.3	0.27	2.08	0.06	3.65	0.03	4.27	0	1.62	0.38	1.13	2	0.57	0
9168	2.64	0.11	3.63	0.01	3.66	0.03	5.63	0	2.48	0.44	1.81	2	0.4	0
9247	-1.91	0.15	-2.47	0.03	-1.95	0.2	-3.56	0	-1.37	0.38	0.83	2	0.66	0
9276	1.47	0.22	2.61	0.02	1.8	0.24	3.33	0.01	1.27	0.38	1.12	2	0.57	0
93210	-0.62	0.71	-2.39	0.03	-3.39	0.04	-3.64	0	-1.47	0.4	2.24	2	0.33	0
9332	2.77	0.1	2.94	0.01	2.96	0.06	4.84	0	2.01	0.41	1.69	2	0.43	0
9340	-1.42	0.23	-2.78	0.02	-1.52	0.33	-3.18	0.01	-1.21	0.38	1.99	2	0.37	0

9366	-1.57	0.2	-2.75	0.02	-1.75	0.25	-3.4	0	-1.31	0.38	1.52	2	0.47	0
9367	2.03	0.14	3.23	0.01	2.24	0.14	3.84	0	1.72	0.45	2.33	2	0.31	0
9369	-0.93	0.47	-3.03	0.01	-3.83	0.02	-3.83	0	-1.83	0.48	2.78	2	0.25	0
9372	-0.8	0.57	-3.02	0.01	-3.39	0.04	-3.56	0	-1.67	0.47	2.73	2	0.26	0
9403	1.53	0.21	2.56	0.02	3.49	0.03	4.54	0	1.77	0.39	0.45	2	0.8	0
9410	1.11	0.35	2.38	0.04	2.62	0.09	3.63	0	1.37	0.38	0.54	2	0.76	0
9445	1.41	0.24	3.17	0.01	2.87	0.07	4.31	0	1.71	0.4	1.72	2	0.42	0
9446	2.3	0.12	3.33	0.01	2.58	0.09	4.25	0	1.92	0.45	2.27	2	0.32	0
9473	1.93	0.15	2.65	0.02	2.79	0.08	4.27	0	1.67	0.39	0.23	2	0.89	0
9474	0.73	0.62	2.31	0.04	2.73	0.08	3.49	0	1.31	0.37	1.17	2	0.56	0
9510	2.21	0.13	1.03	0.36	3.43	0.04	3.18	0.01	1.47	0.46	2.82	2	0.24	0
9532	0.83	0.55	2.65	0.02	2.36	0.12	3.44	0	1.3	0.38	1.47	2	0.48	0
9535	2.51	0.12	3.13	0.01	2.5	0.1	4.34	0	1.87	0.43	2.14	2	0.34	0
9541	2	0.14	2.67	0.02	3.24	0.05	4.64	0	1.84	0.4	0.05	2	0.98	0
9542	-1.85	0.16	-1.19	0.29	-2.72	0.08	-3.36	0.01	-1.24	0.37	0.99	2	0.61	0
9597	-1.82	0.16	-3.16	0.01	-2.82	0.07	-4.48	0	-1.8	0.4	1.25	2	0.54	0
9648	1.61	0.19	2.63	0.02	1.85	0.23	3.44	0	1.31	0.38	1.1	2	0.58	0
9651	-1.53	0.21	-0.97	0.4	-2.78	0.08	-3.12	0.01	-1.14	0.37	1.25	2	0.53	0
9672	2.07	0.14	1.89	0.09	2.09	0.17	3.42	0	1.29	0.38	0.55	2	0.76	0
9687	-1.84	0.16	-2.69	0.02	-1.72	0.26	-3.47	0	-1.34	0.39	1.53	2	0.47	0
9688	2.1	0.14	2.75	0.02	1.74	0.26	3.62	0	1.41	0.39	1.93	2	0.38	0
9757	-1.14	0.34	-2.19	0.05	-2.03	0.18	-3.14	0.01	-1.17	0.37	0.37	2	0.83	0
9796	-1.5	0.21	-2.27	0.04	-2.37	0.12	-3.59	0	-1.35	0.38	0.14	2	0.93	0
9825	-2.37	0.12	-2.95	0.01	-3.85	0.02	-5.4	0	-2.23	0.41	0.03	2	0.98	0
9836	-1.56	0.2	-1.13	0.32	-2.62	0.09	-3.13	0.01	-1.15	0.37	0.78	2	0.68	0
9839	1.67	0.18	2.29	0.04	1.87	0.22	3.32	0.01	1.25	0.38	0.52	2	0.77	0
9848	-2.16	0.13	-2.41	0.03	-1.69	0.27	-3.44	0	-1.32	0.39	1.49	2	0.47	0
9901	-1.08	0.37	-2.13	0.06	-2.64	0.09	-3.5	0	-1.31	0.37	0.4	2	0.82	0
9905	-1.02	0.4	-1.32	0.24	-3.2	0.05	-3.38	0	-1.24	0.37	1.58	2	0.45	0
9914	-1.3	0.27	-3.2	0.01	-2.18	0.15	-3.27	0.01	-1.51	0.46	2.61	2	0.27	0
9915	-2.62	0.11	-2.58	0.02	-3.95	0.02	-5.35	0	-2.2	0.41	0.52	2	0.77	0
9925	-0.67	0.67	-1.74	0.12	-2.72	0.08	-3.14	0.01	-1.15	0.37	1	2	0.61	0
994	1.27	0.28	1.69	0.13	2.54	0.1	3.28	0.01	1.21	0.37	0.17	2	0.92	0
9980	-1.7	0.17	-2.25	0.05	-2.57	0.09	-3.81	0	-1.45	0.38	0.04	2	0.98	0
