# Supplementary material for: Meta-Analysis of Polymyositis and Dermatomyositis Microarray Data Reveals Novel Genetic Biomarkers
Source: Genes (Basel). 2019 Oct 30;10(11):864. doi: 10.3390/genes10110864 (PMC6895911; doi:10.3390/genes10110864)
Supplement: Supplementary file 1 [file genes-10-00864-s001.zip › Supplementary_files/Supplementary Table 2.pdf]

Supplementary Table 2. Table used for network construction with tsv file format.

Node1	Node2	Node1_string_internal_id	Node2_string_internal_id	Node1_external_id	Node2_external_id	Neighborhood_on_chromosome	Gene_fusion	Phylogenetic_cooccurrence	Homology	Coexpression	Experimentally_determined_interaction	Database_annotated	Automated_textmining	Combined_score
NFYA	NFYB	1852963	1843547	9606.ENSP00000345702	9606.ENSP00000240055	0	0	0	0	0.096	0.988	0.9	0.957	0.999
EXOSC2	EXOSC4	1855666	1849789	9606.ENSP00000361433	9606.ENSP00000315476	0.309	0	0.318	0	0.464	0.997	0.9	0.804	0.999
EXOSC10	EXOSC4	1856580	1849789	9606.ENSP00000366135	9606.ENSP00000315476	0	0	0	0	0.085	0.989	0.941	0.82	0.999
IMP3	RPS14	1850884	1849373	9606.ENSP00000326981	9606.ENSP00000311028	0.309	0	0	0	0.952	0.773	0.924	0.254	0.999
LSM4	LSM1	1864161	1849319	9606.ENSP00000469468	9606.ENSP00000310596	0	0	0	0	0.101	0.987	0.9	0.902	0.999
COPB2	COPE	1851147	1845219	9606.ENSP00000329419	9606.ENSP00000262812	0	0	0	0	0.124	0.958	0.9	0.875	0.999
TMED10	TMED2	1848494	1845096	9606.ENSP00000303145	9606.ENSP00000262225	0	0	0	0.701	0.554	0.989	0.9	0.883	0.999
AP2B1	AP2M1	1849703	1847379	9606.ENSP00000314414	9606.ENSP00000292807	0	0	0	0	0.221	0.997	0.9	0.535	0.999
EXOSC10	EXOSC5	1856580	1842821	9606.ENSP00000366135	9606.ENSP00000221233	0	0	0	0	0.093	0.987	0.941	0.837	0.999
EXOSC2	EXOSC1	1855666	1855369	9606.ENSP00000361433	9606.ENSP00000359939	0	0	0	0	0.35	0.997	0.9	0.846	0.999
NOP56	RRP9	1857348	1843325	9606.ENSP00000370589	9606.ENSP00000232888	0	0	0	0	0.872	0.672	0.9	0.82	0.999
EXOSC9	EXOSC2	1857072	1855666	9606.ENSP00000368984	9606.ENSP00000361433	0.309	0	0.232	0	0.464	0.997	0.9	0.758	0.999
YKT6	BET1	1842981	1842924	9606.ENSP00000223369	9606.ENSP00000222547	0	0	0	0	0.128	0.914	0.9	0.894	0.999
COPB2	ARCN1	1851147	1845462	9606.ENSP00000329419	9606.ENSP00000264028	0	0	0	0	0.416	0.959	0.9	0.64	0.999
DKC1	NHP2	1855055	1846399	9606.ENSP00000358563	9606.ENSP00000274606	0.309	0	0	0	0.952	0.931	0.9	0.952	0.999
EXOSC10	EXOSC1	1856580	1855369	9606.ENSP00000366135	9606.ENSP00000359939	0	0	0	0	0.084	0.989	0.9	0.73	0.999
ATP6V0D1	ATP6V1B1	1847269	1843400	9606.ENSP00000290949	9606.ENSP00000234396	0.309	0	0	0	0.67	0.745	0.924	0.843	0.999
EXOSC10	EXOSC2	1856580	1855666	9606.ENSP00000366135	9606.ENSP00000361433	0	0	0	0	0.124	0.989	0.9	0.688	0.999
EXOSC2	EXOSC5	1855666	1842821	9606.ENSP00000361433	9606.ENSP00000221233	0.309	0	0.356	0	0.729	0.989	0.9	0.846	0.999
EIF4E	EIF4EBP1	1861659	1852358	9606.ENSP00000425561	9606.ENSP00000340691	0	0	0	0	0	0.99	0.9	0.976	0.999
EXOSC4	EXOSC5	1849789	1842821	9606.ENSP00000315476	9606.ENSP00000221233	0	0	0.503	0.741	0.316	0.987	0.9	0.93	0.999
GOSR2	BET1	1843032	1842924	9606.ENSP00000225567	9606.ENSP00000222547	0	0	0	0	0.216	0.862	0.9	0.941	0.999
EIF4G1	EIF4A2	1852064	1850828	9606.ENSP00000338020	9606.ENSP00000326381	0	0	0	0	0.074	0.709	0.9	0.976	0.999
EXOSC1	EXOSC4	1855369	1849789	9606.ENSP00000359939	9606.ENSP00000315476	0	0	0	0	0.285	0.997	0.9	0.93	0.999
EXOSC9	EXOSC1	1857072	1855369	9606.ENSP00000368984	9606.ENSP00000359939	0	0	0	0	0.209	0.997	0.9	0.836	0.999
ARCN1	COPB1	1845462	1843983	9606.ENSP00000264028	9606.ENSP00000249923	0	0	0	0	0.688	0.959	0.9	0.671	0.999
NOP56	NOP14	1857348	1849808	9606.ENSP00000370589	9606.ENSP00000315674	0	0	0	0	0.951	0.672	0.9	0.466	0.999
EXOSC1	EXOSC5	1855369	1842821	9606.ENSP00000359939	9606.ENSP00000221233	0	0	0	0	0.374	0.987	0.9	0.932	0.999
EIF4E	EIF4G1	1861659	1852064	9606.ENSP00000425561	9606.ENSP00000338020	0	0	0	0	0.091	0.969	0.9	0.981	0.999
EXOSC9	EXOSC5	1857072	1842821	9606.ENSP00000368984	9606.ENSP00000221233	0.309	0	0.46	0	0.263	0.987	0.9	0.92	0.999
EXOSC9	EXOSC10	1857072	1856580	9606.ENSP00000368984	9606.ENSP00000366135	0	0	0	0	0.15	0.987	0.9	0.951	0.999
NOP56	DKC1	1857348	1855055	9606.ENSP00000370589	9606.ENSP00000358563	0.163	0	0	0	0.954	0.885	0	0.823	0.999
EXOSC9	EXOSC4	1857072	1849789	9606.ENSP00000368984	9606.ENSP00000315476	0.309	0	0.422	0	0.351	0.997	0.9	0.847	0.999
COPB2	COPB1	1851147	1843983	9606.ENSP00000329419	9606.ENSP00000249923	0	0	0	0	0.826	0.959	0.9	0.755	0.999
HSPA5	HSP90B1	1850618	1848102	9606.ENSP00000324173	9606.ENSP00000299767	0	0	0	0	0.965	0.699	0.36	0.945	0.999
EIF4G1	EIF4A1	1852064	1847443	9606.ENSP00000338020	9606.ENSP00000293831	0	0	0	0	0.1	0.887	0.9	0.976	0.999
ATP6V1H	ATP6V1B1	1853860	1843400	9606.ENSP00000352522	9606.ENSP00000234396	0	0	0	0	0.464	0.932	0.9	0.85	0.999
IMP3	RRP9	1850884	1843325	9606.ENSP00000326981	9606.ENSP00000232888	0	0	0	0	0.558	0.931	0.9	0.521	0.998
AP2M1	AP2S1	1847379	1845323	9606.ENSP00000292807	9606.ENSP00000263270	0	0	0	0	0.466	0.938	0.9	0.496	0.998
USO1	YKT6	1862154	1842981	9606.ENSP00000440586	9606.ENSP00000223369	0	0	0	0	0.053	0.965	0.9	0.625	0.998
COPE	COPB1	1845219	1843983	9606.ENSP00000262812	9606.ENSP00000249923	0	0	0	0	0.27	0.958	0.9	0.442	0.998
ATP6V1H	ATP6V0D1	1853860	1847269	9606.ENSP00000352522	9606.ENSP00000290949	0	0	0	0	0.294	0.926	0.9	0.824	0.998
SRPRB	SRPR	1861356	1850982	9606.ENSP00000418401	9606.ENSP00000328023	0	0	0	0	0.122	0.984	0.9	0.333	0.998
CLTC	CLTA	1846064	1843621	9606.ENSP00000269122	9606.ENSP00000242285	0	0	0	0	0.054	0.885	0.9	0.839	0.998
CALR	HSP90B1	1850288	1848102	9606.ENSP00000320866	9606.ENSP00000299767	0	0	0	0	0.734	0.784	0.9	0.788	0.998
AP3S1	AP3B1	1850729	1844363	9606.ENSP00000325369	9606.ENSP00000255194	0	0	0	0	0.055	0.906	0.9	0.827	0.998
SNAP23	VAMP3	1843971	1842224	9606.ENSP00000249647	9606.ENSP00000054666	0	0	0	0	0.118	0.781	0.9	0.885	0.997
NOP56	NHP2	1857348	1846399	9606.ENSP00000370589	9606.ENSP00000274606	0	0	0	0	0.956	0.633	0	0.845	0.997
EIF4E	EIF4A1	1861659	1847443	9606.ENSP00000425561	9606.ENSP00000293831	0	0	0	0	0.124	0.569	0.9	0.944	0.997
AP2B1	AP2S1	1849703	1845323	9606.ENSP00000314414	9606.ENSP00000263270	0	0	0	0	0.055	0.955	0.9	0.492	0.997
ARCN1	COPE	1845462	1845219	9606.ENSP00000264028	9606.ENSP00000262812	0	0	0	0	0.12	0.958	0.9	0.311	0.997
EXOSC10	SKIV2L2	1856580	1843261	9606.ENSP00000366135	9606.ENSP00000230640	0	0	0	0	0.29	0.8	0.9	0.837	0.997
EIF4E	EIF4A2	1861659	1850828	9606.ENSP00000425561	9606.ENSP00000326381	0	0	0	0	0.124	0.423	0.9	0.947	0.997
NAPG	NAPA	1850661	1845337	9606.ENSP00000324628	9606.ENSP00000263354	0	0	0	0	0.087	0.85	0.9	0.814	0.997
ARFGAP1	ARF1	1849722	1846224	9606.ENSP00000314615	9606.ENSP00000272102	0	0	0	0	0.063	0.808	0.9	0.873	0.997
CNOT4	CNOT2	1862294	1843182	9606.ENSP00000445508	9606.ENSP00000229195	0	0	0	0	0.182	0.717	0.9	0.902	0.997
GBF1	ARF1	1855151	1846224	9606.ENSP00000359000	9606.ENSP00000272102	0	0	0	0	0.07	0.853	0.9	0.744	0.996
SHC1	EGFR	1860266	1846445	9606.ENSP00000401303	9606.ENSP00000275493	0	0	0	0	0	0.585	0.9	0.917	0.996
STX16	NAPA	1855414	1845337	9606.ENSP00000360183	9606.ENSP00000263354	0	0	0	0	0.073	0.926	0.9	0.57	0.996
ERN1	HSPA5	1860279	1850618	9606.ENSP00000401445	9606.ENSP00000324173	0	0	0	0	0.05	0.445	0.9	0.937	0.996
VAMP7	SNAP23	1845189	1843971	9606.ENSP00000262640	9606.ENSP00000249647	0	0	0	0	0.16	0.692	0.9	0.866	0.996
ATF6	HSPA5	1854690	1850618	9606.ENSP00000356919	9606.ENSP00000324173	0	0	0	0	0.049	0.407	0.9	0.937	0.995
SPCS3	SEC11A	1861710	1846006	9606.ENSP00000427463	9606.ENSP00000268220	0	0	0	0	0.794	0.258	0.9	0.743	0.995
STX16	VAMP4	1855414	1843436	9606.ENSP00000360183	9606.ENSP00000236192	0	0	0	0	0.073	0.672	0.9	0.858	0.995
GOSR2	YKT6	1843032	1842981	9606.ENSP00000225567	9606.ENSP00000223369	0	0	0	0	0.115	0.86	0.9	0.689	0.995
SEC31A	SEC24D	1853159	1846673	9606.ENSP00000347329	9606.ENSP00000280551	0	0	0	0	0.728	0.166	0.9	0.844	0.995
NOP14	RRP9	1849808	1843325	9606.ENSP00000315674	9606.ENSP00000232888	0	0	0	0	0.884	0.324	0.9	0.361	0.994
DCP2	EDC4	1857792	1853770	9606.ENSP00000373715	9606.ENSP00000351811	0	0	0	0	0.069	0.449	0.9	0.9	0.994
ERN1	XBP1	1860279	1842534	9606.ENSP00000401445	9606.ENSP00000216037	0	0	0	0	0.05	0	0.8	0.975	0.994
USO1	BET1	1862154	1842924	9606.ENSP00000440586	9606.ENSP00000222547	0	0	0	0	0.124	0.785	0.9	0.71	0.993
ATF3	ATF4	1852803	1851927	9606.ENSP00000344352	9606.ENSP00000336790	0	0	0	0	0.054	0.632	0.9	0.843	0.993
EIF4G1	EIF4A3	1852064	1846086	9606.ENSP00000338020	9606.ENSP00000269349	0	0	0	0	0.074	0.679	0.9	0.807	0.993
ARF1	ARFGAP3	1846224	1845314	9606.ENSP00000272102	9606.ENSP00000263245	0	0	0	0	0.065	0.496	0.9	0.882	0.993
ATF6	XBP1	1854690	1842534	9606.ENSP00000356919	9606.ENSP00000216037	0	0	0	0	0.047	0.655	0	0.98	0.993
STX12	SNAP23	1856019	1843971	9606.ENSP00000363054	9606.ENSP00000249647	0	0	0	0	0.055	0.869	0.9	0.449	0.992
STX12	VAMP7	1856019	1845189	9606.ENSP00000363054	9606.ENSP00000262640	0	0	0	0	0.086	0.838	0.9	0.573	0.992
EIF4EBP1	EIF4G1	1852358	1852064	9606.ENSP00000340691	9606.ENSP00000338020	0	0	0	0	0.047	0	0.9	0.929	0.992
CNOT4	CNOT6	1862294	1845032	9606.ENSP00000445508	9606.ENSP00000261951	0	0	0	0	0.11	0.82	0.9	0.563	0.992
NAPA	VAMP7	1845337	1845189	9606.ENSP00000263354	9606.ENSP00000262640	0	0	0	0	0.101	0.744	0.9	0.723	0.992
USO1	GOSR2	1862154	1843032	9606.ENSP00000440586	9606.ENSP00000225567	0	0	0	0	0.055	0.846	0.9	0.52	0.992
STX7	VAMP7	1854689	1845189	9606.ENSP00000356918	9606.ENSP00000262640	0	0	0	0	0.142	0.849	0.54	0.867	0.991
HYOU1	HSP90B1	1858903	1848102	9606.ENSP00000384144	9606.ENSP00000299767	0	0	0	0	0.922	0.421	0.36	0.733	0.991
DCP2	LSM1	1857792	1849319	9606.ENSP00000373715	9606.ENSP00000310596	0	0	0	0	0	0.392	0.9	0.863	0.99
HSP90B1	PDIA6	1848102	1846238	9606.ENSP00000299767	9606.ENSP00000272227	0	0	0	0	0.962	0.273	0.36	0.48	0.989
RPS14	EEF2	1849373	1849021	9606.ENSP00000311028	9606.ENSP00000307940	0.309	0	0	0	0.791	0.053	0.9	0.351	0.989
CNOT6	CNOT2	1845032	1843182	9606.ENSP00000261951	9606.ENSP00000229195	0	0	0	0	0.145	0.761	0.9	0.553	0.989
AP1G1	AP2B1	1858105	1849703	9606.ENSP00000377148	9606.ENSP00000314414	0	0	0	0.612	0.306	0.939	0.72	0.463	0.989
STX7	NAPA	1854689	1845337	9606.ENSP00000356918	9606.ENSP00000263354	0	0	0	0	0.06	0.89	0.54	0.785	0.988
HSPA5	CALR	1850618	1850288	9606.ENSP00000324173	9606.ENSP00000320866	0	0	0	0	0.307	0.064	0.9	0.844	0.988
STX7	YKT6	1854689	1842981	9606.ENSP00000356918	9606.ENSP00000223369	0	0	0	0	0.096	0.828	0.8	0.679	0.988
TSG101	VPS4B	1844091	1843485	9606.ENSP00000251968	9606.ENSP00000238497	0	0	0	0	0.122	0.495	0.9	0.743	0.987
VEGFA	EGFR	1855611	1846445	9606.ENSP00000361125	9606.ENSP00000275493	0	0	0	0	0	0	0.8	0.942	0.987
EIF2AK3	EIF2S1	1848934	1844427	9606.ENSP00000307235	9606.ENSP00000256383	0	0	0	0	0.052	0.36	0.9	0.824	0.987
ARFGAP1	ARFGAP3	1849722	1845314	9606.ENSP00000314615	9606.ENSP00000263245	0	0	0	0.688	0.099	0.833	0.9	0.695	0.987
AP1G1	ARF1	1858105	1846224	9606.ENSP00000377148	9606.ENSP00000272102	0	0	0	0	0.044	0.826	0.9	0.309	0.987
NAPA	YKT6	1845337	1842981	9606.ENSP00000263354	9606.ENSP00000223369	0	0	0	0	0.159	0.733	0.9	0.518	0.987
IMP3	NOP14	1850884	1849808	9606.ENSP00000326981	9606.ENSP00000315674	0	0	0	0	0.577	0.717	0.9	0.09	0.987
AP2B1	CLTC	1849703	1846064	9606.ENSP00000314414	9606.ENSP00000269122	0	0	0	0	0.125	0.714	0.9	0.515	0.986
IMP3	NHP2	1850884	1846399	9606.ENSP00000326981	9606.ENSP00000274606	0.082	0	0	0	0.957	0.673	0	0.101	0.986
EIF4E	EIF2S1	1861659	1844427	9606.ENSP00000425561	9606.ENSP00000256383	0	0	0	0	0.304	0.071	0.9	0.81	0.986
NAPA	GOSR2	1845337	1843032	9606.ENSP00000263354	9606.ENSP00000225567	0	0	0	0	0.072	0.781	0.9	0.357	0.985
STX12	VAMP3	1856019	1842224	9606.ENSP00000363054	9606.ENSP00000054666	0	0	0	0	0.1	0.78	0.9	0.358	0.985
STX16	VAMP3	1855414	1842224	9606.ENSP00000360183	9606.ENSP00000054666	0	0	0	0	0.073	0.672	0.9	0.592	0.985
NOP56	IMP3	1857348	1850884	9606.ENSP00000370589	9606.ENSP00000326981	0	0	0	0	0.478	0.672	0.9	0.189	0.984
SEC11A	SPCS1	1846006	1843331	9606.ENSP00000268220	9606.ENSP00000233025	0	0	0	0	0.498	0.257	0.9	0.641	0.984
EXOSC4	SKIV2L2	1849789	1843261	9606.ENSP00000315476	9606.ENSP00000230640	0	0	0	0	0.122	0.505	0.9	0.675	0.984
ATF4	EIF2AK3	1851927	1848934	9606.ENSP00000336790	9606.ENSP00000307235	0	0	0	0	0	0	0.9	0.854	0.984
ATF4	HSPA5	1851927	1850618	9606.ENSP00000336790	9606.ENSP00000324173	0	0	0	0	0.057	0	0.9	0.834	0.983
EXOSC1	SKIV2L2	1855369	1843261	9606.ENSP00000359939	9606.ENSP00000230640	0	0	0	0	0.102	0.531	0.9	0.659	0.983
AP1G1	AP3B1	1858105	1844363	9606.ENSP00000377148	9606.ENSP00000255194	0	0	0	0	0.319	0.524	0.9	0.517	0.982
VAMP7	VAMP3	1845189	1842224	9606.ENSP00000262640	9606.ENSP00000054666	0	0	0	0	0.073	0.286	0.9	0.77	0.982
SKIV2L2	EXOSC5	1843261	1842821	9606.ENSP00000230640	9606.ENSP00000221233	0	0	0	0	0.12	0.417	0.9	0.7	0.982
HYOU1	DNAJC3	1858903	1856557	9606.ENSP00000384144	9606.ENSP00000365991	0.309	0	0	0	0.764	0.592	0.274	0.684	0.982
SHC1	MAPK1	1860266	1842512	9606.ENSP00000401303	9606.ENSP00000215832	0	0	0	0	0.055	0.38	0.9	0.736	0.982
IGF2R	M6PR	1853441	1842110	9606.ENSP00000349437	9606.ENSP00000000412	0	0	0	0	0.051	0	0.9	0.823	0.981
EXOSC9	SKIV2L2	1857072	1843261	9606.ENSP00000368984	9606.ENSP00000230640	0	0	0	0	0.211	0.424	0.9	0.636	0.981
NOP56	NOLC1	1857348	1855156	9606.ENSP00000370589	9606.ENSP00000359024	0	0	0	0	0.673	0.851	0	0.637	0.981
PAIP1	EIF4A1	1848447	1847443	9606.ENSP00000302768	9606.ENSP00000293831	0	0	0	0	0.053	0.36	0.9	0.728	0.981
NAPA	BET1	1845337	1842924	9606.ENSP00000263354	9606.ENSP00000222547	0	0	0	0	0.068	0.644	0.9	0.447	0.979
NAPG	VAMP4	1850661	1843436	9606.ENSP00000324628	9606.ENSP00000236192	0	0	0	0	0.055	0.798	0.9	0	0.979
PARN	KHSRP	1859310	1858577	9606.ENSP00000387911	9606.ENSP00000381216	0	0	0	0	0.053	0.36	0.9	0.701	0.979
STX7	VAMP3	1854689	1842224	9606.ENSP00000356918	9606.ENSP00000054666	0	0	0	0	0.073	0.906	0.54	0.538	0.979
STX7	VAMP4	1854689	1843436	9606.ENSP00000356918	9606.ENSP00000236192	0	0	0	0	0.073	0.906	0.54	0.549	0.979
STX16	VPS45	1855414	1854956	9606.ENSP00000360183	9606.ENSP00000358126	0	0	0	0	0.093	0.926	0	0.71	0.979
EIF4E	EIF4A3	1861659	1846086	9606.ENSP00000425561	9606.ENSP00000269349	0	0	0	0	0.124	0.387	0.9	0.64	0.978
STX7	SNAP23	1854689	1843971	9606.ENSP00000356918	9606.ENSP00000249647	0	0	0	0	0.158	0.878	0.54	0.605	0.978
SPCS3	SPCS1	1861710	1843331	9606.ENSP00000427463	9606.ENSP00000233025	0	0	0	0	0.464	0.292	0.9	0.477	0.977
VPS45	RAB5A	1854956	1846291	9606.ENSP00000358126	9606.ENSP00000273047	0	0	0	0	0.095	0.409	0.9	0.626	0.977
ARF1	COPB1	1846224	1843983	9606.ENSP00000272102	9606.ENSP00000249923	0	0	0	0	0.098	0.529	0.9	0.528	0.977
NAPA	VAMP3	1845337	1842224	9606.ENSP00000263354	9606.ENSP00000054666	0	0	0	0	0.101	0.513	0.9	0.537	0.977
EIF4A2	EIF4A1	1850828	1847443	9606.ENSP00000326381	9606.ENSP00000293831	0	0	0.527	0.987	0	0.774	0.9	0.823	0.976
CEBPB	MAPK1	1848728	1842512	9606.ENSP00000305422	9606.ENSP00000215832	0	0	0	0	0	0.36	0.9	0.659	0.976
AP1G1	AP3S1	1858105	1850729	9606.ENSP00000377148	9606.ENSP00000325369	0	0	0	0	0.057	0.534	0.9	0.514	0.975
STX12	NAPA	1856019	1845337	9606.ENSP00000363054	9606.ENSP00000263354	0	0	0	0	0.06	0.89	0.54	0.528	0.974
RAB9A	M6PR	1861457	1842110	9606.ENSP00000420127	9606.ENSP00000000412	0	0	0	0	0.053	0	0.9	0.755	0.974
EIF4EBP1	EIF4A1	1852358	1847443	9606.ENSP00000340691	9606.ENSP00000293831	0	0	0	0	0.042	0.046	0.9	0.739	0.973
VAMP7	VAMP4	1845189	1843436	9606.ENSP00000262640	9606.ENSP00000236192	0	0	0	0	0.1	0.187	0.9	0.676	0.973
ATF4	XBP1	1851927	1842534	9606.ENSP00000336790	9606.ENSP00000216037	0	0	0	0	0.085	0	0.8	0.865	0.973
EXOSC2	SKIV2L2	1855666	1843261	9606.ENSP00000361433	9606.ENSP00000230640	0	0	0	0	0.252	0.226	0.9	0.601	0.973
COPB2	ARFGAP3	1851147	1845314	9606.ENSP00000329419	9606.ENSP00000263245	0	0	0	0	0.144	0.672	0.9	0.177	0.973
NAPA	VAMP4	1845337	1843436	9606.ENSP00000263354	9606.ENSP00000236192	0	0	0	0	0.101	0.513	0.9	0.464	0.973
ARFGAP3	COPB1	1845314	1843983	9606.ENSP00000263245	9606.ENSP00000249923	0	0	0	0	0.102	0.672	0.9	0.202	0.973
AP2M1	CLTC	1847379	1846064	9606.ENSP00000292807	9606.ENSP00000269122	0	0	0	0	0.101	0.446	0.9	0.537	0.973
EIF4G1	EIF2S1	1852064	1844427	9606.ENSP00000338020	9606.ENSP00000256383	0	0	0	0	0.135	0.094	0.9	0.686	0.972
ARF1	GOSR2	1846224	1843032	9606.ENSP00000272102	9606.ENSP00000225567	0	0	0	0	0.054	0.446	0.9	0.537	0.972
EIF4EBP1	EIF4A2	1852358	1850828	9606.ENSP00000340691	9606.ENSP00000326381	0	0	0	0	0.042	0.046	0.9	0.736	0.972
ARFGEF2	ARF1	1855574	1846224	9606.ENSP00000360985	9606.ENSP00000272102	0	0	0	0	0.047	0.296	0.9	0.632	0.972
ATF4	ASNS	1851927	1842325	9606.ENSP00000336790	9606.ENSP00000175506	0	0	0	0	0.097	0	0.9	0.717	0.972
ATF6	HSP90B1	1854690	1848102	9606.ENSP00000356919	9606.ENSP00000299767	0	0	0	0	0.047	0	0.9	0.734	0.972
SNAP23	VAMP4	1843971	1843436	9606.ENSP00000249647	9606.ENSP00000236192	0	0	0	0	0.061	0.672	0.8	0.608	0.972
ATF4	EIF2S1	1851927	1844427	9606.ENSP00000336790	9606.ENSP00000256383	0	0	0	0	0	0	0.9	0.731	0.971
ERN1	ATF6	1860279	1854690	9606.ENSP00000401445	9606.ENSP00000356919	0	0	0	0	0.099	0	0	0.968	0.971
STX16	YKT6	1855414	1842981	9606.ENSP00000360183	9606.ENSP00000223369	0	0	0	0	0.073	0.799	0.54	0.709	0.971
EIF4A2	PAIP1	1850828	1848447	9606.ENSP00000326381	9606.ENSP00000302768	0	0	0	0	0.053	0	0.9	0.727	0.971
SEC31A	LMAN1	1853159	1844034	9606.ENSP00000347329	9606.ENSP00000251047	0	0	0	0	0.113	0	0.9	0.699	0.971
LSM4	DCP2	1864161	1857792	9606.ENSP00000469468	9606.ENSP00000373715	0	0	0	0	0.06	0.07	0.9	0.701	0.97
VAMP7	YKT6	1845189	1842981	9606.ENSP00000262640	9606.ENSP00000223369	0	0	0	0	0.074	0.691	0.54	0.804	0.97
HSPA5	EIF2AK3	1850618	1848934	9606.ENSP00000324173	9606.ENSP00000307235	0	0	0	0	0.073	0.36	0.9	0.567	0.97
HSPA5	DNAJB9	1850618	1843958	9606.ENSP00000324173	9606.ENSP00000249356	0.309	0	0.46	0	0.459	0.528	0.274	0.654	0.97
ARF1	ARFGEF1	1846224	1845094	9606.ENSP00000272102	9606.ENSP00000262215	0	0	0	0	0.047	0.296	0.9	0.618	0.97
RPS6KA3	MAPK1	1857057	1842512	9606.ENSP00000368884	9606.ENSP00000215832	0	0	0	0.697	0.146	0.547	0.9	0.875	0.969
VAMP7	BET1	1845189	1842924	9606.ENSP00000262640	9606.ENSP00000222547	0	0	0	0	0.187	0.863	0.36	0.627	0.969
STX12	VAMP4	1856019	1843436	9606.ENSP00000363054	9606.ENSP00000236192	0	0	0	0	0.1	0.888	0.54	0.397	0.968
SEC24D	PREB	1846673	1844807	9606.ENSP00000280551	9606.ENSP00000260643	0	0	0	0	0.072	0.071	0.9	0.68	0.968
EXOSC2	IMP3	1855666	1850884	9606.ENSP00000361433	9606.ENSP00000326981	0	0	0	0	0.694	0	0.9	0	0.968
AP1G1	IGF2R	1858105	1853441	9606.ENSP00000377148	9606.ENSP00000349437	0	0	0	0	0.134	0.637	0.9	0.103	0.968
RAB9A	IGF2R	1861457	1853441	9606.ENSP00000420127	9606.ENSP00000349437	0	0	0	0	0.053	0	0.9	0.69	0.968
EXOSC10	NOP14	1856580	1849808	9606.ENSP00000366135	9606.ENSP00000315674	0	0	0	0	0.685	0	0.9	0.057	0.967
EXOSC2	NOP14	1855666	1849808	9606.ENSP00000361433	9606.ENSP00000315674	0	0	0	0	0.683	0	0.9	0	0.967
EIF4A3	SKIV2L2	1846086	1843261	9606.ENSP00000269349	9606.ENSP00000230640	0	0	0	0	0.126	0.449	0.9	0.41	0.967
TMED2	LMAN1	1845096	1844034	9606.ENSP00000262225	9606.ENSP00000251047	0	0	0	0	0.229	0.503	0.9	0.236	0.966
EGFR	MAPK1	1846445	1842512	9606.ENSP00000275493	9606.ENSP00000215832	0	0	0	0.632	0	0.503	0.9	0.932	0.965
ARF1	AP3B1	1846224	1844363	9606.ENSP00000272102	9606.ENSP00000255194	0	0	0	0	0.055	0.521	0.9	0.334	0.965
SEC31A	BET1	1853159	1842924	9606.ENSP00000347329	9606.ENSP00000222547	0	0	0	0	0	0.277	0.9	0.543	0.964
ATF6	CALR	1854690	1850288	9606.ENSP00000356919	9606.ENSP00000320866	0	0	0	0	0	0	0.9	0.656	0.964
EIF4EBP1	MAPK1	1852358	1842512	9606.ENSP00000340691	9606.ENSP00000215832	0	0	0	0	0	0.409	0.8	0.717	0.963
STAM	TSG101	1856699	1844091	9606.ENSP00000366746	9606.ENSP00000251968	0	0	0	0	0.052	0.098	0.9	0.625	0.963
AP1G1	CLTC	1858105	1846064	9606.ENSP00000377148	9606.ENSP00000269122	0	0	0	0	0.181	0.411	0.9	0.307	0.962
EIF4A1	EIF2S1	1847443	1844427	9606.ENSP00000293831	9606.ENSP00000256383	0	0	0	0	0.241	0.07	0.9	0.531	0.962
ARF1	COPE	1846224	1845219	9606.ENSP00000272102	9606.ENSP00000262812	0	0	0	0	0.176	0.415	0.9	0.318	0.962
EIF4A2	EIF2S1	1850828	1844427	9606.ENSP00000326381	9606.ENSP00000256383	0	0	0	0	0.241	0.07	0.9	0.531	0.962
RAB5A	VAMP7	1846291	1845189	9606.ENSP00000273047	9606.ENSP00000262640	0	0	0	0	0.213	0	0.9	0.548	0.961
ARF1	BET1	1846224	1842924	9606.ENSP00000272102	9606.ENSP00000222547	0	0	0	0	0.057	0.169	0.9	0.55	0.96
SEC24D	BET1	1846673	1842924	9606.ENSP00000280551	9606.ENSP00000222547	0	0	0	0	0.065	0.17	0.9	0.545	0.96
RPS14	RRP9	1849373	1843325	9606.ENSP00000311028	9606.ENSP00000232888	0	0	0	0	0.15	0.499	0.9	0.174	0.96
RAB9A	NAPA	1861457	1845337	9606.ENSP00000420127	9606.ENSP00000263354	0	0	0	0	0.122	0.17	0.9	0.508	0.959
IMP3	EXOSC5	1850884	1842821	9606.ENSP00000326981	9606.ENSP00000221233	0.055	0	0	0	0.605	0	0.9	0	0.959
NOP56	DDX10	1857348	1849698	9606.ENSP00000370589	9606.ENSP00000314348	0	0	0	0	0.849	0.702	0	0.177	0.959

NOP14	SKIV2L2	1849808	1843261	9606.ENSP00000315674	9606.ENSP00000230640	0	0	0	0	0.612	0	0.9	0	0.959
DNAJC3	PDIA6	1856557	1846238	9606.ENSP00000365991	9606.ENSP00000272227	0	0	0	0	0.844	0.586	0	0.414	0.958
GBF1	COPB1	1855151	1843983	9606.ENSP00000359000	9606.ENSP00000249923	0	0	0	0	0.353	0.383	0.9	0.086	0.958
OCRL	RAB5A	1855405	1846291	9606.ENSP00000360154	9606.ENSP00000273047	0	0	0	0	0.052	0.377	0.9	0.363	0.957
NOP56	EXOSC4	1857348	1849789	9606.ENSP00000370589	9606.ENSP00000315476	0	0	0	0	0.269	0.42	0.9	0.108	0.957
PARN	EIF4G1	1859310	1852064	9606.ENSP00000387911	9606.ENSP00000338020	0	0	0	0	0.053	0	0.9	0.574	0.956
ARF1	TMED2	1846224	1845096	9606.ENSP00000272102	9606.ENSP00000262225	0	0	0	0	0.18	0.325	0.9	0.299	0.956
TMED10	LMAN1	1848494	1844034	9606.ENSP00000303145	9606.ENSP00000251047	0	0	0	0	0.126	0.417	0.9	0.251	0.956
RAB5A	M6PR	1846291	1842110	9606.ENSP00000273047	9606.ENSP00000000412	0	0	0	0	0.053	0	0.9	0.574	0.956
PARN	DCP2	1859310	1857792	9606.ENSP00000387911	9606.ENSP00000373715	0	0	0	0	0.088	0	0.9	0.554	0.955
VPS45	STX7	1854956	1854689	9606.ENSP00000358126	9606.ENSP00000356918	0	0	0	0	0.11	0.884	0	0.597	0.955
VAMP4	YKT6	1843436	1842981	9606.ENSP00000236192	9606.ENSP00000223369	0	0	0	0	0.048	0.673	0.54	0.728	0.955
OCRL	AP2B1	1855405	1849703	9606.ENSP00000360154	9606.ENSP00000314414	0	0	0	0	0.066	0.444	0.9	0.233	0.954
USO1	GBF1	1862154	1855151	9606.ENSP00000440586	9606.ENSP00000359000	0	0	0	0	0.133	0.402	0.9	0.219	0.954
RPS6KA3	CEBPB	1857057	1848728	9606.ENSP00000368884	9606.ENSP00000305422	0	0	0	0	0	0	0.9	0.566	0.954
SEC31A	COPB2	1853159	1851147	9606.ENSP00000347329	9606.ENSP00000329419	0	0	0	0	0.388	0	0.9	0.316	0.954
USO1	ARF1	1862154	1846224	9606.ENSP00000440586	9606.ENSP00000272102	0	0	0	0	0.054	0.293	0.9	0.401	0.954
NOP56	SKIV2L2	1857348	1843261	9606.ENSP00000370589	9606.ENSP00000230640	0	0	0	0	0.36	0.095	0.9	0.284	0.953
ARFGAP3	BET1	1845314	1842924	9606.ENSP00000263245	9606.ENSP00000222547	0	0	0	0	0	0.263	0.9	0.416	0.953
SEC24D	TMED2	1846673	1845096	9606.ENSP00000280551	9606.ENSP00000262225	0	0	0	0	0.101	0.357	0.9	0.286	0.953
EIF4E	PAIP1	1861659	1848447	9606.ENSP00000425561	9606.ENSP00000302768	0	0	0	0	0.115	0	0.9	0.502	0.952
H2AFX	NPM1	1856252	1847808	9606.ENSP00000364310	9606.ENSP00000296930	0	0	0	0	0.053	0.36	0.9	0.304	0.952
ALDH18A1	CKS1B	1855437	1849378	9606.ENSP00000360268	9606.ENSP00000311083	0	0	0	0	0.059	0.952	0	0	0.952
NOP56	SDAD1	1857348	1853320	9606.ENSP00000370589	9606.ENSP00000348596	0	0	0	0	0.951	0.065	0	0	0.952
SDAD1	NOP14	1853320	1849808	9606.ENSP00000348596	9606.ENSP00000315674	0	0	0	0	0.952	0	0	0	0.952
SDAD1	RRP9	1853320	1843325	9606.ENSP00000348596	9606.ENSP00000232888	0	0	0	0	0.952	0	0	0	0.952
YKT6	VAMP3	1842981	1842224	9606.ENSP00000223369	9606.ENSP00000054666	0	0	0	0	0.048	0.673	0.54	0.701	0.951
LMAN1	BET1	1844034	1842924	9606.ENSP00000251047	9606.ENSP00000222547	0	0	0	0	0.053	0	0.9	0.53	0.951
COG2	COPB1	1854425	1843983	9606.ENSP00000355629	9606.ENSP00000249923	0	0	0	0	0.127	0	0.9	0.491	0.951
SEC24D	GOSR2	1846673	1843032	9606.ENSP00000280551	9606.ENSP00000225567	0	0	0	0	0.092	0.17	0.9	0.432	0.951
COPB1	GOSR2	1843983	1843032	9606.ENSP00000249923	9606.ENSP00000225567	0	0	0	0	0	0.095	0.9	0.508	0.951
IMP3	EEF2	1850884	1849021	9606.ENSP00000326981	9606.ENSP00000307940	0.309	0	0	0	0.913	0	0	0.255	0.951
EDC4	LSM1	1853770	1849319	9606.ENSP00000351811	9606.ENSP00000310596	0	0	0	0	0.053	0	0.9	0.527	0.951
LMAN1	CTSC	1844034	1843108	9606.ENSP00000251047	9606.ENSP00000227266	0	0	0	0	0.054	0	0.9	0.525	0.951
TMED10	ARF1	1848494	1846224	9606.ENSP00000303145	9606.ENSP00000272102	0	0	0	0	0.105	0.396	0.9	0.189	0.95
EGFR	RAB5A	1846445	1846291	9606.ENSP00000275493	9606.ENSP00000273047	0	0	0	0	0	0	0.9	0.515	0.949
USO1	COPB1	1862154	1843983	9606.ENSP00000440586	9606.ENSP00000249923	0	0	0	0	0.46	0.066	0.9	0.112	0.949
COPB2	GOSR2	1851147	1843032	9606.ENSP00000329419	9606.ENSP00000225567	0	0	0	0	0.053	0.292	0.9	0.336	0.949
RAB5A	VAMP3	1846291	1842224	9606.ENSP00000273047	9606.ENSP00000054666	0	0	0	0	0.099	0	0.9	0.477	0.948
COPB2	ARF1	1851147	1846224	9606.ENSP00000329419	9606.ENSP00000272102	0	0	0	0	0.073	0.178	0.9	0.4	0.948
COG2	GOSR2	1854425	1843032	9606.ENSP00000355629	9606.ENSP00000225567	0	0	0	0	0.051	0	0.9	0.493	0.947
IMP3	EXOSC4	1850884	1849789	9606.ENSP00000326981	9606.ENSP00000315476	0.055	0	0	0	0.491	0	0.9	0	0.947
NAPG	YKT6	1850661	1842981	9606.ENSP00000324628	9606.ENSP00000223369	0	0	0	0	0.052	0.486	0.9	0	0.947
STX16	VAMP7	1855414	1845189	9606.ENSP00000360183	9606.ENSP00000262640	0	0	0	0	0.124	0.521	0.72	0.609	0.947
NAPG	BET1	1850661	1842924	9606.ENSP00000324628	9606.ENSP00000222547	0	0	0	0	0	0.486	0.9	0.068	0.947
STX16	NAPG	1855414	1850661	9606.ENSP00000360183	9606.ENSP00000324628	0	0	0	0	0.095	0.47	0.9	0	0.947
RAB9A	STX16	1861457	1855414	9606.ENSP00000420127	9606.ENSP00000360183	0	0	0	0	0.072	0.07	0.9	0.461	0.947
SH3GL2	EGFR	1857254	1846445	9606.ENSP00000369981	9606.ENSP00000275493	0	0	0	0	0.053	0.36	0.9	0.221	0.946
HYOU1	HSPA5	1858903	1850618	9606.ENSP00000384144	9606.ENSP00000324173	0	0	0	0.726	0.846	0.34	0.36	0.889	0.946
SEC24D	LMAN1	1846673	1844034	9606.ENSP00000280551	9606.ENSP00000251047	0	0	0	0	0.163	0	0.9	0.41	0.946
STX16	SNAP23	1855414	1843971	9606.ENSP00000360183	9606.ENSP00000249647	0	0	0	0	0.15	0.602	0.72	0.505	0.946
RAB5A	CLTC	1846291	1846064	9606.ENSP00000273047	9606.ENSP00000269122	0	0	0	0	0.057	0.108	0.9	0.44	0.946
GBF1	COPB2	1855151	1851147	9606.ENSP00000359000	9606.ENSP00000329419	0	0	0	0	0.29	0.265	0.9	0.089	0.946
OCRL	CLTC	1855405	1846064	9606.ENSP00000360154	9606.ENSP00000269122	0	0	0	0	0.071	0.416	0.9	0.13	0.946
EXOSC1	IMP3	1855369	1850884	9606.ENSP00000359939	9606.ENSP00000326981	0	0	0	0	0.48	0	0.9	0	0.945
DCP2	EXOSC5	1857792	1842821	9606.ENSP00000373715	9606.ENSP00000221233	0	0	0	0	0.054	0	0.9	0.466	0.945
DNAJA4	HSPA9	1858237	1847834	9606.ENSP00000378324	9606.ENSP00000297185	0.309	0	0.336	0	0.4	0.592	0.274	0.453	0.945
IARS	TARS	1856347	1845694	9606.ENSP00000364794	9606.ENSP00000265112	0	0	0	0	0.814	0	0	0.718	0.945
IGF2R	RAB5A	1853441	1846291	9606.ENSP00000349437	9606.ENSP00000273047	0	0	0	0	0.072	0.048	0.9	0.445	0.944
EIF4A2	EIF4A3	1850828	1846086	9606.ENSP00000326381	9606.ENSP00000269349	0	0	0.527	0.973	0.089	0.417	0.9	0.731	0.944
HYOU1	DNAJB9	1858903	1843958	9606.ENSP00000384144	9606.ENSP00000249356	0.309	0	0	0	0.4	0.329	0.274	0.765	0.944
RRP9	SKIV2L2	1843325	1843261	9606.ENSP00000232888	9606.ENSP00000230640	0	0	0	0	0.359	0.096	0.9	0.148	0.944
COPB2	BET1	1851147	1842924	9606.ENSP00000329419	9606.ENSP00000222547	0	0	0	0	0.104	0.292	0.9	0.216	0.943
KHSRP	EXOSC2	1858577	1855666	9606.ENSP00000381216	9606.ENSP00000361433	0	0	0	0	0.095	0.36	0.9	0.144	0.943
SNX2	IGF2R	1857048	1853441	9606.ENSP00000368831	9606.ENSP00000349437	0	0	0	0	0.075	0.079	0.9	0.417	0.943
NOLC1	DKC1	1855156	1855055	9606.ENSP00000359024	9606.ENSP00000358563	0	0	0	0	0.5	0.567	0	0.757	0.943
RAB14	YWHAZ	1855997	1849183	9606.ENSP00000362946	9606.ENSP00000309503	0	0	0	0	0.103	0.184	0.9	0.313	0.943
ARF1	YKT6	1846224	1842981	9606.ENSP00000272102	9606.ENSP00000223369	0	0	0	0	0.086	0.119	0.9	0.385	0.943
SHC1	VEGFA	1860266	1855611	9606.ENSP00000401303	9606.ENSP00000361125	0	0	0	0	0	0	0.9	0.46	0.943
DNAJA4	HSPA5	1858237	1850618	9606.ENSP00000378324	9606.ENSP00000324173	0.309	0	0.389	0	0.4	0.592	0.274	0.391	0.943
AP2B1	CLTA	1849703	1843621	9606.ENSP00000314414	9606.ENSP00000242285	0	0	0	0	0.054	0.118	0.9	0.396	0.942
ARFGAP1	BET1	1849722	1842924	9606.ENSP00000314615	9606.ENSP00000222547	0	0	0	0	0	0.118	0.9	0.387	0.941
NOP14	RPS14	1849808	1849373	9606.ENSP00000315674	9606.ENSP00000311028	0	0	0	0	0.054	0.397	0.9	0.088	0.941
COPB2	TMED10	1851147	1848494	9606.ENSP00000329419	9606.ENSP00000303145	0	0	0	0	0.246	0.248	0.9	0.091	0.941
GBF1	ARFGAP1	1855151	1849722	9606.ENSP00000359000	9606.ENSP00000314615	0	0	0	0	0.174	0	0.9	0.341	0.94
RAB9A	VAMP3	1861457	1842224	9606.ENSP00000420127	9606.ENSP00000054666	0	0	0	0	0.113	0.121	0.9	0.324	0.94
GALC	GLA	1844883	1842708	9606.ENSP00000261304	9606.ENSP00000218516	0.064	0	0.222	0	0.05	0	0.9	0.272	0.94
MON2	DOPEY1	1858112	1842368	9606.ENSP00000377252	9606.ENSP00000195654	0	0	0	0	0.422	0.672	0	0.71	0.94
HSP90B1	HSPA9	1848102	1847834	9606.ENSP00000299767	9606.ENSP00000297185	0	0	0	0	0.415	0.613	0	0.756	0.94
NOP56	RPS14	1857348	1849373	9606.ENSP00000370589	9606.ENSP00000311028	0	0	0	0	0.087	0.133	0.9	0.334	0.94
AP1G1	AP2M1	1858105	1847379	9606.ENSP00000377148	9606.ENSP00000292807	0	0	0	0	0.159	0.678	0.72	0.295	0.939
USO1	SEC24D	1862154	1846673	9606.ENSP00000440586	9606.ENSP00000280551	0	0	0	0	0.157	0.071	0.9	0.315	0.939
ARF1	M6PR	1846224	1842110	9606.ENSP00000272102	9606.ENSP00000000412	0	0	0	0	0.065	0	0.9	0.408	0.939
ARCN1	YKT6	1845462	1842981	9606.ENSP00000264028	9606.ENSP00000223369	0	0	0	0	0.086	0.373	0.9	0.066	0.939
GBF1	ARCN1	1855151	1845462	9606.ENSP00000359000	9606.ENSP00000264028	0	0	0	0	0.35	0	0.9	0.131	0.938
ARFGAP1	TMED2	1849722	1845096	9606.ENSP00000314615	9606.ENSP00000262225	0	0	0	0	0.084	0.375	0.9	0.045	0.938
DNAJC3	HSPA5	1856557	1850618	9606.ENSP00000365991	9606.ENSP00000324173	0.309	0	0	0	0.4	0.527	0.274	0.635	0.938
SNX2	VAMP7	1857048	1845189	9606.ENSP00000368831	9606.ENSP00000262640	0	0	0	0	0.318	0.096	0.9	0.126	0.938
EXOSC2	RRP9	1855666	1843325	9606.ENSP00000361433	9606.ENSP00000232888	0	0	0	0	0.414	0	0.9	0	0.938
COPB2	SEC24D	1851147	1846673	9606.ENSP00000329419	9606.ENSP00000280551	0	0	0	0	0.292	0.065	0.9	0.169	0.937
OCRL	AP2M1	1855405	1847379	9606.ENSP00000360154	9606.ENSP00000292807	0	0	0	0	0.054	0.359	0.9	0.093	0.937
COPB2	ZW10	1851147	1842389	9606.ENSP00000329419	9606.ENSP00000200135	0	0	0	0	0.101	0.36	0.9	0	0.937
HERPUD1	ATF4	1860798	1851927	9606.ENSP00000409555	9606.ENSP00000336790	0	0	0	0	0.093	0	0.9	0.364	0.937
USO1	NAPA	1862154	1845337	9606.ENSP00000440586	9606.ENSP00000263354	0	0	0	0	0	0.079	0.9	0.369	0.936
NAPG	GOSR2	1850661	1843032	9606.ENSP00000324628	9606.ENSP00000225567	0	0	0	0	0	0.394	0.9	0	0.936
TMED10	COPB1	1848494	1843983	9606.ENSP00000303145	9606.ENSP00000249923	0	0	0	0	0.227	0.248	0.9	0	0.936
DCP2	EXOSC4	1857792	1849789	9606.ENSP00000373715	9606.ENSP00000315476	0	0	0	0	0.055	0	0.9	0.381	0.936
PREB	LMAN1	1844807	1844034	9606.ENSP00000260643	9606.ENSP00000251047	0	0	0	0	0.072	0	0.9	0.373	0.936
EXOSC9	NOP14	1857072	1849808	9606.ENSP00000368984	9606.ENSP00000315674	0	0	0	0	0.39	0	0.9	0	0.936
SHC1	YWHAZ	1860266	1849183	9606.ENSP00000401303	9606.ENSP00000309503	0	0	0	0	0	0.36	0.9	0.092	0.936
KDELR3	TMED10	1859221	1848494	9606.ENSP00000386918	9606.ENSP00000303145	0	0	0	0	0.27	0.137	0.9	0.105	0.936
PARN	EXOSC5	1859310	1842821	9606.ENSP00000387911	9606.ENSP00000221233	0	0	0	0	0.053	0	0.9	0.382	0.936
ARCN1	GOSR2	1845462	1843032	9606.ENSP00000264028	9606.ENSP00000225567	0	0	0	0	0.1	0.095	0.9	0.3	0.935
IGF2R	EGFR	1853441	1846445	9606.ENSP00000349437	9606.ENSP00000275493	0	0	0	0	0	0	0.9	0.38	0.935
RRP9	EXOSC5	1843325	1842821	9606.ENSP00000232888	9606.ENSP00000221233	0	0	0	0	0.325	0	0.9	0.114	0.935
EGFR	TSG101	1846445	1844091	9606.ENSP00000275493	9606.ENSP00000251968	0	0	0	0	0	0	0.9	0.38	0.935
OCRL	CLTA	1855405	1843621	9606.ENSP00000360154	9606.ENSP00000242285	0	0	0	0	0	0.364	0.9	0.068	0.935
OCRL	AP2S1	1855405	1845323	9606.ENSP00000360154	9606.ENSP00000263270	0	0	0	0	0.05	0.359	0.9	0.065	0.935
EIF4E	PARN	1861659	1859310	9606.ENSP00000425561	9606.ENSP00000387911	0	0	0	0	0	0	0.9	0.38	0.935
KHSRP	DCP2	1858577	1857792	9606.ENSP00000381216	9606.ENSP00000373715	0	0	0	0	0.058	0	0.9	0.368	0.935
USO1	SEC31A	1862154	1853159	9606.ENSP00000440586	9606.ENSP00000347329	0	0	0	0	0.096	0	0.9	0.333	0.934
STX7	GOSR2	1854689	1843032	9606.ENSP00000356918	9606.ENSP00000225567	0	0	0	0	0.073	0.805	0.54	0.298	0.934
NOP56	EXOSC2	1857348	1855666	9606.ENSP00000370589	9606.ENSP00000361433	0	0	0	0	0.318	0	0.9	0.115	0.934
NOP56	EXOSC10	1857348	1856580	9606.ENSP00000370589	9606.ENSP00000366135	0	0	0	0	0.24	0	0.9	0.201	0.934
TMED10	ARCN1	1848494	1845462	9606.ENSP00000303145	9606.ENSP00000264028	0	0	0	0	0.18	0.248	0.9	0.047	0.933
USO1	COPB2	1862154	1851147	9606.ENSP00000440586	9606.ENSP00000329419	0	0	0	0	0.269	0.071	0.9	0.132	0.933
HSPA5	PDIA6	1850618	1846238	9606.ENSP00000324173	9606.ENSP00000272227	0.048	0	0	0	0.376	0.554	0.54	0.539	0.933
RAB5A	VAMP4	1846291	1843436	9606.ENSP00000273047	9606.ENSP00000236192	0	0	0	0	0.1	0	0.9	0.318	0.933
YWHAZ	DDIT4	1849183	1848949	9606.ENSP00000309503	9606.ENSP00000307305	0	0	0	0	0	0.36	0.9	0	0.933
BAG3	HSPA5	1854947	1850618	9606.ENSP00000358081	9606.ENSP00000324173	0	0	0	0	0.091	0.17	0.9	0.225	0.933
COPB2	ARFGAP1	1851147	1849722	9606.ENSP00000329419	9606.ENSP00000314615	0	0	0	0	0.122	0.188	0.9	0.17	0.932
DCP2	EXOSC1	1857792	1855369	9606.ENSP00000373715	9606.ENSP00000359939	0	0	0	0	0.054	0.182	0.9	0.225	0.932
STX16	IGF2R	1855414	1853441	9606.ENSP00000360183	9606.ENSP00000349437	0	0	0	0	0.135	0	0.9	0.277	0.932
IGF2R	ARF1	1853441	1846224	9606.ENSP00000349437	9606.ENSP00000272102	0	0	0	0	0	0.071	0.9	0.33	0.932
ARFGAP1	COPB1	1849722	1843983	9606.ENSP00000314615	9606.ENSP00000249923	0	0	0	0	0.084	0.169	0.9	0.204	0.931
YIPF6	OCRL	1861304	1855405	9606.ENSP00000417573	9606.ENSP00000360154	0	0	0	0	0.346	0	0.9	0	0.931
VAMP3	M6PR	1842224	1842110	9606.ENSP00000054666	9606.ENSP00000000412	0	0	0	0	0.053	0	0.9	0.33	0.931
KDELR3	ARCN1	1859221	1845462	9606.ENSP00000386918	9606.ENSP00000264028	0	0	0	0	0.125	0	0.9	0.276	0.931
NOP56	EXOSC9	1857348	1857072	9606.ENSP00000370589	9606.ENSP00000368984	0	0	0	0	0.241	0.125	0.9	0.084	0.931
AP1G1	OCRL	1858105	1855405	9606.ENSP00000377148	9606.ENSP00000360154	0	0	0	0	0.099	0.278	0.9	0.065	0.931
EIF4G1	PAIP1	1852064	1848447	9606.ENSP00000338020	9606.ENSP00000302768	0	0	0	0.641	0.048	0	0.9	0.849	0.93
DCTN1	KIF5B	1854219	1848911	9606.ENSP00000354791	9606.ENSP00000307078	0	0	0	0	0.099	0.142	0.9	0.212	0.93
VAMP7	M6PR	1845189	1842110	9606.ENSP00000262640	9606.ENSP00000000412	0	0	0	0	0.075	0	0.9	0.304	0.93
HSPA5	XBP1	1850618	1842534	9606.ENSP00000324173	9606.ENSP00000216037	0	0	0	0	0.259	0	0	0.91	0.93
EIF4A2	RPS14	1850828	1849373	9606.ENSP00000326381	9606.ENSP00000311028	0	0	0	0	0.129	0.097	0.9	0.218	0.93
PARN	EXOSC4	1859310	1849789	9606.ENSP00000387911	9606.ENSP00000315476	0	0	0	0	0.047	0	0.9	0.332	0.93
ARF1	ARCN1	1846224	1845462	9606.ENSP00000272102	9606.ENSP00000264028	0	0	0	0	0.093	0.171	0.9	0.189	0.93
RPS14	EIF4A1	1849373	1847443	9606.ENSP00000311028	9606.ENSP00000293831	0	0	0	0	0.129	0.097	0.9	0.218	0.93
GBF1	ARFGAP3	1855151	1845314	9606.ENSP00000359000	9606.ENSP00000263245	0	0	0	0	0.125	0	0.9	0.264	0.93
STX16	M6PR	1855414	1842110	9606.ENSP00000360183	9606.ENSP00000000412	0	0	0	0	0	0	0.9	0.329	0.93
AP2M1	RAB5A	1847379	1846291	9606.ENSP00000292807	9606.ENSP00000273047	0	0	0	0	0.104	0	0.9	0.289	0.93
KDELR3	BET1	1859221	1842924	9606.ENSP00000386918	9606.ENSP00000222547	0	0	0	0	0.065	0	0.9	0.306	0.929
EGFR	M6PR	1846445	1842110	9606.ENSP00000275493	9606.ENSP00000000412	0	0	0	0	0	0	0.9	0.326	0.929
NOP56	EXOSC5	1857348	1842821	9606.ENSP00000370589	9606.ENSP00000221233	0	0	0	0	0.239	0.046	0.9	0.133	0.928
TMED10	COPE	1848494	1845219	9606.ENSP00000303145	9606.ENSP00000262812	0	0	0	0	0.126	0.248	0.9	0	0.928
NFYA	HSPA5	1852963	1850618	9606.ENSP00000345702	9606.ENSP00000324173	0	0	0	0	0.054	0	0.9	0.306	0.928
VAMP4	M6PR	1843436	1842110	9606.ENSP00000236192	9606.ENSP00000000412	0	0	0	0	0.052	0	0.9	0.305	0.928
NOP56	EXOSC1	1857348	1855369	9606.ENSP00000370589	9606.ENSP00000359939	0	0	0	0	0.31	0	0.9	0.048	0.928
AP2S1	MAPK1	1845323	1842512	9606.ENSP00000263270	9606.ENSP00000215832	0	0	0	0	0.051	0.292	0.9	0.057	0.928
RAB2A	MAPK1	1845192	1842512	9606.ENSP00000262646	9606.ENSP00000215832	0	0	0	0	0.053	0.18	0.9	0.192	0.928
CEBPB	CEBPG	1848728	1846879	9606.ENSP00000305422	9606.ENSP00000284000	0	0	0	0.805	0	0.628	0.8	0.345	0.927
USO1	TMED10	1862154	1848494	9606.ENSP00000440586	9606.ENSP00000303145	0	0	0	0	0.1	0	0.9	0.263	0.927
SPCS3	SSR1	1861710	1843739	9606.ENSP00000427463	9606.ENSP00000244763	0	0	0	0	0.239	0	0.9	0.124	0.927
STX16	GOLGA4	1855414	1853422	9606.ENSP00000360183	9606.ENSP00000349305	0	0	0	0	0.117	0	0.9	0.248	0.927
DNAJC3	HSPA9	1856557	1847834	9606.ENSP00000365991	9606.ENSP00000297185	0.309	0	0	0	0.4	0.718	0.274	0.271	0.927
YWHAZ	MAPK1	1849183	1842512	9606.ENSP00000309503	9606.ENSP00000215832	0	0	0	0	0.106	0.099	0.9	0.202	0.927
TMED2	PREB	1845096	1844807	9606.ENSP00000262225	9606.ENSP00000260643	0	0	0	0	0.054	0	0.9	0.299	0.927
SEC31A	TMED2	1853159	1845096	9606.ENSP00000347329	9606.ENSP00000262225	0	0	0	0	0	0.094	0.9	0.267	0.927
LSM4	EDC4	1864161	1853770	9606.ENSP00000469468	9606.ENSP00000351811	0	0	0	0	0	0	0.9	0.301	0.927
LMAN1	GOSR2	1844034	1843032	9606.ENSP00000251047	9606.ENSP00000225567	0	0	0	0	0.056	0	0.9	0.29	0.927
OCRL	ARF1	1855405	1846224	9606.ENSP00000360154	9606.ENSP00000272102	0	0	0	0	0.046	0.069	0.9	0.282	0.927
SH3GL2	OCRL	1857254	1855405	9606.ENSP00000369981	9606.ENSP00000360154	0	0	0	0	0.055	0.11	0.9	0.225	0.926
EXOSC9	RRP9	1857072	1843325	9606.ENSP00000368984	9606.ENSP00000232888	0	0	0	0	0.246	0	0.9	0.106	0.926
USO1	LMAN1	1862154	1844034	9606.ENSP00000440586	9606.ENSP00000251047	0	0	0	0	0.087	0	0.9	0.254	0.926
STX12	YKT6	1856019	1842981	9606.ENSP00000363054	9606.ENSP00000223369	0	0	0	0	0.096	0.721	0.54	0.439	0.926
DCP2	EXOSC9	1857792	1857072	9606.ENSP00000373715	9606.ENSP00000368984	0	0	0	0	0.059	0	0.9	0.277	0.926
USO1	ARFGAP3	1862154	1845314	9606.ENSP00000440586	9606.ENSP00000263245	0	0	0	0	0.154	0.118	0.9	0.126	0.926
ARF1	VAMP7	1846224	1845189	9606.ENSP00000272102	9606.ENSP00000262640	0	0	0	0	0.053	0.119	0.9	0.218	0.926
NOP14	EXOSC4	1849808	1849789	9606.ENSP00000315674	9606.ENSP00000315476	0	0	0	0	0.282	0	0.9	0	0.925
SEC31A	TMED10	1853159	1848494	9606.ENSP00000347329	9606.ENSP00000303145	0	0	0	0	0.147	0.094	0.9	0.148	0.925
TMED10	SEC24D	1848494	1846673	9606.ENSP00000303145	9606.ENSP00000280551	0	0	0	0	0.109	0.073	0.9	0.199	0.925
SH3GL2	CLTC	1857254	1846064	9606.ENSP00000369981	9606.ENSP00000269122	0	0	0	0	0	0	0.9	0.288	0.925
IGF2R	VAMP4	1853441	1843436	9606.ENSP00000349437	9606.ENSP00000236192	0	0	0	0	0.05	0	0.9	0.274	0.925
KIF5B	KIF1B	1848911	1845440	9606.ENSP00000307078	9606.ENSP00000263934	0	0	0	0.691	0.127	0	0.9	0.611	0.925
CLTC	AP2S1	1846064	1845323	9606.ENSP00000269122	9606.ENSP00000263270	0	0	0	0	0.053	0.12	0.9	0.21	0.925
GOLGA4	VAMP4	1853422	1843436	9606.ENSP00000349305	9606.ENSP00000236192	0	0	0	0	0.068	0	0.9	0.264	0.925
GBF1	GOSR2	1855151	1843032	9606.ENSP00000359000	9606.ENSP00000225567	0	0	0	0	0.089	0	0.9	0.235	0.924
NAPG	VAMP3	1850661	1842224	9606.ENSP00000324628	9606.ENSP00000054666	0	0	0	0	0.055	0.267	0.9	0	0.924
VAMP7	AP3B1	1845189	1844363	9606.ENSP00000262640	9606.ENSP00000255194	0	0	0	0	0.112	0.12	0.9	0.141	0.924
PREB	GOSR2	1844807	1843032	9606.ENSP00000260643	9606.ENSP00000225567	0	0	0	0	0.116	0.045	0.9	0.211	0.924
SRPRB	SSR1	1861356	1843739	9606.ENSP00000418401	9606.ENSP00000244763	0	0	0	0	0.172	0	0.9	0.166	0.924
ARFGAP3	YKT6	1845314	1842981	9606.ENSP00000263245	9606.ENSP00000223369	0	0	0	0	0.057	0.099	0.9	0.217	0.924
EXOSC1	RRP9	1855369	1843325	9606.ENSP00000359939	9606.ENSP00000232888	0	0	0	0	0.266	0	0.9	0.057	0.924
STAM	EGFR	1856699	1846445	9606.ENSP00000366746	9606.ENSP00000275493	0	0	0	0	0	0	0.9	0.272	0.924
AP2M1	EGFR	1847379	1846445	9606.ENSP00000292807	9606.ENSP00000275493	0	0	0	0	0	0.126	0.9	0.206	0.924
ARCN1	TMED2	1845462	1845096	9606.ENSP00000264028	9606.ENSP00000262225	0	0	0	0	0.238	0.069	0.9	0.067	0.924
ARFGAP1	YKT6	1849722	1842981	9606.ENSP00000314615	9606.ENSP00000223369	0	0	0	0	0.044	0.099	0.9	0.218	0.923
LMAN1	YKT6	1844034	1842981	9606.ENSP00000251047	9606.ENSP00000223369	0	0	0	0	0.06	0	0.9	0.256	0.923
SNAP23	YKT6	1843971	1842981	9606.ENSP00000249647	9606.ENSP00000223369	0	0	0	0	0.061	0.539	0.54	0.661	0.923
CNOT4	EIF4G1	1862294	1852064	9606.ENSP00000445508	9606.ENSP00000338020	0	0	0	0	0.223	0	0.9	0.09	0.923

RPS6KA3	ATF4	1857057	1851927	9606.ENSP00000368884	9606.ENSP00000336790	0	0	0	0	0	0	0.8	0.632	0.923
PARN	EIF4A2	1859310	1850828	9606.ENSP00000387911	9606.ENSP00000326381	0	0	0	0	0.055	0	0.9	0.258	0.923
BAG3	HSPA9	1854947	1847834	9606.ENSP00000358081	9606.ENSP00000297185	0	0	0	0	0	0.165	0.9	0.146	0.922
SEC31A	GOSR2	1853159	1843032	9606.ENSP00000347329	9606.ENSP00000225567	0	0	0	0	0.067	0	0.9	0.239	0.922
HSPA9	DNAJB9	1847834	1843958	9606.ENSP00000297185	9606.ENSP00000249356	0.309	0	0.445	0	0.4	0.329	0.274	0.437	0.922
AP2M1	CLTA	1847379	1843621	9606.ENSP00000292807	9606.ENSP00000242285	0	0	0	0	0.151	0.071	0.9	0.136	0.922
USO1	ARFGAP1	1862154	1849722	9606.ENSP00000440586	9606.ENSP00000314615	0	0	0	0	0.092	0.118	0.9	0.141	0.922
EXOSC4	RRP9	1849789	1843325	9606.ENSP00000315476	9606.ENSP00000232888	0	0	0	0	0.214	0	0.9	0.09	0.922
USO1	COG2	1862154	1854425	9606.ENSP00000440586	9606.ENSP00000355629	0	0	0	0	0.118	0	0.9	0.193	0.922
ARFGAP1	AP2B1	1849722	1849703	9606.ENSP00000314615	9606.ENSP00000314414	0	0	0	0	0.065	0.169	0.9	0.11	0.921
PARN	EIF4A1	1859310	1847443	9606.ENSP00000387911	9606.ENSP00000293831	0	0	0	0	0.055	0	0.9	0.231	0.921
RPS14	EIF4A3	1849373	1846086	9606.ENSP00000311028	9606.ENSP00000269349	0	0	0	0	0.129	0.097	0.9	0.125	0.921
EIF4G1	CNOT6	1852064	1845032	9606.ENSP00000338020	9606.ENSP00000261951	0	0	0	0	0.103	0	0.9	0.19	0.921
ATF4	CCL2	1851927	1843055	9606.ENSP00000336790	9606.ENSP00000225831	0	0	0	0	0	0	0.9	0.25	0.921
SRPR	SSR1	1850982	1843739	9606.ENSP00000328023	9606.ENSP00000244763	0	0	0	0	0.225	0	0.9	0.064	0.921
ERO1L	PDIA6	1858318	1846238	9606.ENSP00000379042	9606.ENSP00000272227	0	0	0	0	0.058	0.094	0.8	0.588	0.92
OCRL	AP3B1	1855405	1844363	9606.ENSP00000360154	9606.ENSP00000255194	0	0	0	0	0.074	0.169	0.9	0.09	0.92
STX7	NAPG	1854689	1850661	9606.ENSP00000356918	9606.ENSP00000324628	0	0	0	0	0.094	0.807	0.54	0.124	0.92
HSPA5	NFYB	1850618	1843547	9606.ENSP00000324173	9606.ENSP00000240055	0	0	0	0	0	0	0.9	0.235	0.92
EXOSC10	RRP9	1856580	1843325	9606.ENSP00000366135	9606.ENSP00000232888	0	0	0	0	0.222	0	0.9	0.062	0.92
SNX2	AP3B1	1857048	1844363	9606.ENSP00000368831	9606.ENSP00000255194	0	0	0	0	0.118	0.064	0.9	0.148	0.92
RAB14	EXOC2	1855997	1843254	9606.ENSP00000362946	9606.ENSP00000230449	0	0	0	0	0	0.17	0.9	0.12	0.92
ARFGAP1	CLTC	1849722	1846064	9606.ENSP00000314615	9606.ENSP00000269122	0	0	0	0	0.072	0.17	0.9	0.086	0.92
CLTC	VAMP7	1846064	1845189	9606.ENSP00000269122	9606.ENSP00000262640	0	0	0	0	0.053	0.067	0.9	0.194	0.919
EGFR	CLTC	1846445	1846064	9606.ENSP00000275493	9606.ENSP00000269122	0	0	0	0	0.05	0	0.9	0.217	0.919
KDELR3	ARF1	1859221	1846224	9606.ENSP00000386918	9606.ENSP00000272102	0	0	0	0	0.058	0.121	0.9	0.142	0.919
PARN	PAIP1	1859310	1848447	9606.ENSP00000387911	9606.ENSP00000302768	0	0	0	0	0	0	0.9	0.231	0.919
ARFGAP1	ARCN1	1849722	1845462	9606.ENSP00000314615	9606.ENSP00000264028	0	0	0	0	0.154	0.063	0.9	0.101	0.919
NOP14	EXOSC5	1849808	1842821	9606.ENSP00000315674	9606.ENSP00000221233	0	0	0	0	0.23	0	0.9	0	0.919
IMP3	SKIV2L2	1850884	1843261	9606.ENSP00000326981	9606.ENSP00000230640	0	0	0	0	0.18	0.096	0.9	0	0.919
KIF5B	COPB1	1848911	1843983	9606.ENSP00000307078	9606.ENSP00000249923	0	0	0	0	0.149	0.062	0.9	0.094	0.918
AP2B1	EGFR	1849703	1846445	9606.ENSP00000314414	9606.ENSP00000275493	0	0	0	0	0	0.126	0.9	0.142	0.918
CLTC	VAMP3	1846064	1842224	9606.ENSP00000269122	9606.ENSP00000054666	0	0	0	0	0.052	0.071	0.9	0.187	0.918
IGF2R	AP3B1	1853441	1844363	9606.ENSP00000349437	9606.ENSP00000255194	0	0	0	0	0.181	0	0.9	0.081	0.918
GBF1	BET1	1855151	1842924	9606.ENSP00000359000	9606.ENSP00000222547	0	0	0	0	0.053	0	0.9	0.21	0.918
COPB1	BET1	1843983	1842924	9606.ENSP00000249923	9606.ENSP00000222547	0	0	0	0	0.121	0.095	0.9	0.092	0.918
KDELR3	TMED2	1859221	1845096	9606.ENSP00000386918	9606.ENSP00000262225	0	0	0	0	0.123	0	0.9	0.141	0.918
KDELR3	GOSR2	1859221	1843032	9606.ENSP00000386918	9606.ENSP00000225567	0	0	0	0	0.125	0	0.9	0.142	0.918
SNX2	ARF1	1857048	1846224	9606.ENSP00000368831	9606.ENSP00000272102	0	0	0	0	0.053	0.053	0.9	0.199	0.918
USO1	TMED2	1862154	1845096	9606.ENSP00000440586	9606.ENSP00000262225	0	0	0	0	0.112	0	0.9	0.144	0.917
AP3S1	AP2M1	1850729	1847379	9606.ENSP00000325369	9606.ENSP00000292807	0	0	0	0	0.099	0.539	0.72	0.372	0.917
AP2B1	RAB5A	1849703	1846291	9606.ENSP00000314414	9606.ENSP00000273047	0	0	0	0	0.12	0	0.9	0.136	0.917
TMED2	BET1	1845096	1842924	9606.ENSP00000262225	9606.ENSP00000222547	0	0	0	0	0.112	0	0.9	0.147	0.917
KDELR3	COPB2	1859221	1851147	9606.ENSP00000386918	9606.ENSP00000329419	0	0	0	0	0.127	0	0.9	0.128	0.917
SEC31A	YKT6	1853159	1842981	9606.ENSP00000347329	9606.ENSP00000223369	0	0	0	0	0.049	0.068	0.9	0.182	0.917
ARFGAP1	COPE	1849722	1845219	9606.ENSP00000314615	9606.ENSP00000262812	0	0	0	0	0.062	0.094	0.9	0.146	0.917
IGF2R	VAMP3	1853441	1842224	9606.ENSP00000349437	9606.ENSP00000054666	0	0	0	0	0.048	0	0.9	0.203	0.917
GBF1	DCTN1	1855151	1854219	9606.ENSP00000359000	9606.ENSP00000354791	0	0	0	0	0.182	0	0.9	0.07	0.917
EIF4E	CNOT6	1861659	1845032	9606.ENSP00000425561	9606.ENSP00000261951	0	0	0	0	0.055	0	0.9	0.201	0.917
ARFGAP3	COPE	1845314	1845219	9606.ENSP00000263245	9606.ENSP00000262812	0	0	0	0	0.095	0.068	0.9	0.141	0.917
COPB2	TMED2	1851147	1845096	9606.ENSP00000329419	9606.ENSP00000262225	0	0	0	0	0.15	0.054	0.9	0.09	0.917
PARN	EXOSC9	1859310	1857072	9606.ENSP00000387911	9606.ENSP00000368984	0	0	0	0	0.051	0	0.9	0.205	0.917
TMED2	GOSR2	1845096	1843032	9606.ENSP00000262225	9606.ENSP00000225567	0	0	0	0	0.097	0	0.9	0.159	0.917
KDELR3	ARFGAP3	1859221	1845314	9606.ENSP00000386918	9606.ENSP00000263245	0	0	0	0	0.055	0	0.9	0.188	0.916
ARFGAP3	TMED2	1845314	1845096	9606.ENSP00000263245	9606.ENSP00000262225	0	0	0	0	0.072	0.175	0.9	0	0.916
USO1	ARCN1	1862154	1845462	9606.ENSP00000440586	9606.ENSP00000264028	0	0	0	0	0.131	0.094	0.9	0.064	0.916
KIF5B	COPE	1848911	1845219	9606.ENSP00000307078	9606.ENSP00000262812	0	0	0	0	0	0.176	0.9	0.067	0.916
VEGFA	CCL2	1855611	1843055	9606.ENSP00000361125	9606.ENSP00000225831	0	0	0	0	0	0	0	0.916	0.916
TMED10	BET1	1848494	1842924	9606.ENSP00000303145	9606.ENSP00000222547	0	0	0	0	0.125	0	0.9	0.119	0.916
KHSRP	EXOSC5	1858577	1842821	9606.ENSP00000381216	9606.ENSP00000221233	0	0	0	0	0.058	0	0.9	0.184	0.916
CLTA	VAMP4	1843621	1843436	9606.ENSP00000242285	9606.ENSP00000236192	0	0	0	0	0.096	0.053	0.9	0.14	0.916
ATF4	IGFBP1	1851927	1846447	9606.ENSP00000336790	9606.ENSP00000275525	0	0	0	0	0	0	0.9	0.202	0.916
OCRL	EGFR	1855405	1846445	9606.ENSP00000360154	9606.ENSP00000275493	0	0	0	0	0	0	0.9	0.199	0.916
IGF2R	VAMP7	1853441	1845189	9606.ENSP00000349437	9606.ENSP00000262640	0	0	0	0	0.074	0	0.9	0.168	0.916
AP2S1	CLTA	1845323	1843621	9606.ENSP00000263270	9606.ENSP00000242285	0	0	0	0	0.109	0.071	0.9	0.095	0.915
EIF4E	CNOT2	1861659	1843182	9606.ENSP00000425561	9606.ENSP00000229195	0	0	0	0	0.062	0	0.9	0.171	0.915
EGFR	VAMP3	1846445	1842224	9606.ENSP00000275493	9606.ENSP00000054666	0	0	0	0	0.042	0	0.9	0.19	0.915
RPS14	EIF2S1	1849373	1844427	9606.ENSP00000311028	9606.ENSP00000256383	0	0	0	0	0.124	0	0.9	0.108	0.915
CLTA	VAMP3	1843621	1842224	9606.ENSP00000242285	9606.ENSP00000054666	0	0	0	0	0.096	0.053	0.9	0.13	0.915
TMED10	ARFGAP3	1848494	1845314	9606.ENSP00000303145	9606.ENSP00000263245	0	0	0	0	0.055	0.175	0.9	0	0.915
KDELR3	GBF1	1859221	1855151	9606.ENSP00000386918	9606.ENSP00000359000	0	0	0	0	0.095	0.066	0.9	0.114	0.915
HSPA5	HSPA9	1850618	1847834	9606.ENSP00000324173	9606.ENSP00000297185	0	0	0.526	0.946	0.128	0.051	0.9	0.737	0.915
SEC24D	YKT6	1846673	1842981	9606.ENSP00000280551	9606.ENSP00000223369	0	0	0	0	0.072	0.113	0.9	0.093	0.915
YIPF6	ARF1	1861304	1846224	9606.ENSP00000417573	9606.ENSP00000272102	0	0	0	0	0.054	0.117	0.9	0.104	0.915
EIF4A1	EIF4A3	1847443	1846086	9606.ENSP00000293831	9606.ENSP00000269349	0	0	0.527	0.973	0.164	0	0.9	0.73	0.915
TMED2	COPB1	1845096	1843983	9606.ENSP00000262225	9606.ENSP00000249923	0	0	0	0	0.148	0	0.9	0.08	0.914
EGFR	CLTA	1846445	1843621	9606.ENSP00000275493	9606.ENSP00000242285	0	0	0	0	0	0.129	0.9	0.095	0.914
PAIP1	CNOT6	1848447	1845032	9606.ENSP00000302768	9606.ENSP00000261951	0	0	0	0	0.054	0	0.9	0.172	0.914
STX12	NAPG	1856019	1850661	9606.ENSP00000363054	9606.ENSP00000324628	0	0	0	0	0.075	0.807	0.54	0.078	0.914
COPB2	YKT6	1851147	1842981	9606.ENSP00000329419	9606.ENSP00000223369	0	0	0	0	0.053	0.141	0.9	0.068	0.914
OCRL	IGF2R	1855405	1853441	9606.ENSP00000360154	9606.ENSP00000349437	0	0	0	0	0.053	0	0.9	0.166	0.914
ARF1	CLTA	1846224	1843621	9606.ENSP00000272102	9606.ENSP00000242285	0	0	0	0	0.084	0.052	0.9	0.128	0.914
AP2M1	COPB1	1847379	1843983	9606.ENSP00000292807	9606.ENSP00000249923	0	0	0	0	0.168	0.596	0.72	0.198	0.914
CLTC	VAMP4	1846064	1843436	9606.ENSP00000269122	9606.ENSP00000236192	0	0	0	0	0.052	0.071	0.9	0.141	0.914
AP3S1	ARF1	1850729	1846224	9606.ENSP00000325369	9606.ENSP00000272102	0	0	0	0	0.055	0.145	0.9	0.065	0.914
KHSRP	EXOSC4	1858577	1849789	9606.ENSP00000381216	9606.ENSP00000315476	0	0	0	0	0.054	0	0.9	0.169	0.914
EIF4A1	CNOT6	1847443	1845032	9606.ENSP00000293831	9606.ENSP00000261951	0	0	0	0	0.056	0	0.9	0.163	0.914
SNX2	AP3S1	1857048	1850729	9606.ENSP00000368831	9606.ENSP00000325369	0	0	0	0	0.109	0.073	0.9	0.087	0.914
ARFGAP3	CLTA	1845314	1843621	9606.ENSP00000263245	9606.ENSP00000242285	0	0	0	0	0.091	0.119	0.9	0.053	0.914
CNOT4	EIF4E	1862294	1861659	9606.ENSP00000445508	9606.ENSP00000425561	0	0	0	0	0.053	0	0.9	0.166	0.914
KDELR3	YKT6	1859221	1842981	9606.ENSP00000386918	9606.ENSP00000223369	0	0	0	0	0.1	0	0.9	0.118	0.913
PARN	EXOSC2	1859310	1855666	9606.ENSP00000387911	9606.ENSP00000361433	0	0	0	0	0.054	0	0.9	0.162	0.913
AP3S1	VAMP7	1850729	1845189	9606.ENSP00000325369	9606.ENSP00000262640	0	0	0	0	0.146	0	0.9	0.066	0.913
KDELR3	ARFGAP1	1859221	1849722	9606.ENSP00000386918	9606.ENSP00000314615	0	0	0	0	0.06	0	0.9	0.148	0.913
AP2M1	M6PR	1847379	1842110	9606.ENSP00000292807	9606.ENSP00000000412	0	0	0	0	0	0	0.9	0.17	0.913
AP2B1	VAMP7	1849703	1845189	9606.ENSP00000314414	9606.ENSP00000262640	0	0	0	0	0.099	0.079	0.9	0.08	0.913
OCRL	M6PR	1855405	1842110	9606.ENSP00000360154	9606.ENSP00000000412	0	0	0	0	0	0	0.9	0.167	0.913
ARCN1	ARFGAP3	1845462	1845314	9606.ENSP00000264028	9606.ENSP00000263245	0	0	0	0	0.099	0.071	0.9	0.09	0.913
COPE	TMED2	1845219	1845096	9606.ENSP00000262812	9606.ENSP00000262225	0	0	0	0	0.172	0	0.9	0.043	0.913
COPE	GOSR2	1845219	1843032	9606.ENSP00000262812	9606.ENSP00000225567	0	0	0	0	0.093	0.095	0.9	0.073	0.913
ARFGAP1	TMED10	1849722	1848494	9606.ENSP00000314615	9606.ENSP00000303145	0	0	0	0	0.099	0.113	0.9	0.044	0.913
COPB1	ZW10	1843983	1842389	9606.ENSP00000249923	9606.ENSP00000200135	0	0	0	0	0.147	0	0.9	0.061	0.913
SNAP23	EXOC2	1843971	1843254	9606.ENSP00000249647	9606.ENSP00000230449	0	0	0	0	0	0	0.9	0.16	0.912
COPE	BET1	1845219	1842924	9606.ENSP00000262812	9606.ENSP00000222547	0	0	0	0	0.06	0.095	0.9	0.092	0.912
TMED10	PREB	1848494	1844807	9606.ENSP00000303145	9606.ENSP00000260643	0	0	0	0	0	0.07	0.9	0.135	0.912
RPS14	EXOSC5	1849373	1842821	9606.ENSP00000311028	9606.ENSP00000221233	0	0	0	0	0.134	0	0.9	0.066	0.912
AP1G1	SNX2	1858105	1857048	9606.ENSP00000377148	9606.ENSP00000368831	0	0	0	0	0.114	0	0.9	0.089	0.912
PARN	EXOSC1	1859310	1855369	9606.ENSP00000387911	9606.ENSP00000359939	0	0	0	0	0	0	0.9	0.163	0.912
RAB5A	CLTA	1846291	1843621	9606.ENSP00000273047	9606.ENSP00000242285	0	0	0	0	0.056	0.05	0.9	0.142	0.912
CLTC	MAPK1	1846064	1842512	9606.ENSP00000269122	9606.ENSP00000215832	0	0	0	0	0.054	0.102	0.9	0.096	0.912
EXOSC1	NOP14	1855369	1849808	9606.ENSP00000359939	9606.ENSP00000315674	0	0	0	0	0.152	0	0.9	0	0.911
AP1G1	AP2S1	1858105	1845323	9606.ENSP00000377148	9606.ENSP00000263270	0	0	0	0	0.057	0.673	0.72	0.091	0.911
SH3GL2	VAMP3	1857254	1842224	9606.ENSP00000369981	9606.ENSP00000054666	0	0	0	0	0.055	0	0.9	0.138	0.911
HYOU1	DNAJA4	1858903	1858237	9606.ENSP00000384144	9606.ENSP00000378324	0.309	0	0	0	0.4	0.592	0.274	0.387	0.911
VAMP4	VAMP3	1843436	1842224	9606.ENSP00000236192	9606.ENSP00000054666	0	0	0	0.889	0.105	0	0.9	0.515	0.911
ARCN1	BET1	1845462	1842924	9606.ENSP00000264028	9606.ENSP00000222547	0	0	0	0	0.054	0.095	0.9	0.092	0.911
SEC11A	SSR1	1846006	1843739	9606.ENSP00000268220	9606.ENSP00000244763	0	0	0	0	0.149	0	0.9	0.045	0.911
VPS45	COG2	1854956	1854425	9606.ENSP00000358126	9606.ENSP00000355629	0	0	0	0	0.14	0	0.9	0.051	0.911
LSM4	EIF4A3	1864161	1846086	9606.ENSP00000469468	9606.ENSP00000269349	0	0	0	0	0.065	0	0.9	0.128	0.911
KIF5B	ARF1	1848911	1846224	9606.ENSP00000307078	9606.ENSP00000272102	0	0	0	0	0.053	0.096	0.9	0.092	0.911
KDELR3	COPB1	1859221	1843983	9606.ENSP00000386918	9606.ENSP00000249923	0	0	0	0	0.129	0	0.9	0.065	0.911
AP2B1	VAMP4	1849703	1843436	9606.ENSP00000314414	9606.ENSP00000236192	0	0	0	0	0.08	0.079	0.9	0.08	0.911
EIF4A2	CNOT6	1850828	1845032	9606.ENSP00000326381	9606.ENSP00000261951	0	0	0	0	0.056	0	0.9	0.134	0.911
SH3GL2	ARF1	1857254	1846224	9606.ENSP00000369981	9606.ENSP00000272102	0	0	0	0	0.053	0.054	0.9	0.124	0.911
SH3GL2	AP2M1	1857254	1847379	9606.ENSP00000369981	9606.ENSP00000292807	0	0	0	0	0.053	0	0.9	0.141	0.911
TMED10	GOSR2	1848494	1843032	9606.ENSP00000303145	9606.ENSP00000225567	0	0	0	0	0.09	0.053	0.9	0.091	0.911
RPS14	SEC11A	1849373	1846006	9606.ENSP00000311028	9606.ENSP00000268220	0	0	0	0	0.096	0	0.9	0.097	0.911
EXOSC9	IMP3	1857072	1850884	9606.ENSP00000368984	9606.ENSP00000326981	0	0	0	0	0.154	0	0.9	0	0.911
VAMP7	CLTA	1845189	1843621	9606.ENSP00000262640	9606.ENSP00000242285	0	0	0	0	0.096	0.048	0.9	0.078	0.91
KDELR3	COPE	1859221	1845219	9606.ENSP00000386918	9606.ENSP00000262812	0	0	0	0	0.121	0	0.9	0.067	0.91
YIPF6	VAMP7	1861304	1845189	9606.ENSP00000417573	9606.ENSP00000262640	0	0	0	0	0.12	0	0.9	0.069	0.91
ARFGAP1	CLTA	1849722	1843621	9606.ENSP00000314615	9606.ENSP00000242285	0	0	0	0	0.057	0.119	0.9	0.046	0.91
SRPR	RPS14	1850982	1849373	9606.ENSP00000328023	9606.ENSP00000311028	0	0	0	0	0.06	0	0.9	0.121	0.91
PAIP1	EIF4A3	1848447	1846086	9606.ENSP00000302768	9606.ENSP00000269349	0	0	0	0	0.053	0	0.9	0.127	0.91
ARF1	KIF1B	1846224	1845440	9606.ENSP00000272102	9606.ENSP00000263934	0	0	0	0	0.043	0.096	0.9	0.092	0.91
AP1G1	VAMP7	1858105	1845189	9606.ENSP00000377148	9606.ENSP00000262640	0	0	0	0	0.117	0	0.9	0.064	0.91
EIF4A3	CNOT6	1846086	1845032	9606.ENSP00000269349	9606.ENSP00000261951	0	0	0	0	0.056	0	0.9	0.127	0.91
KHSRP	EXOSC9	1858577	1857072	9606.ENSP00000381216	9606.ENSP00000368984	0	0	0	0	0.044	0	0.9	0.142	0.91
AP2B1	VAMP3	1849703	1842224	9606.ENSP00000314414	9606.ENSP00000054666	0	0	0	0	0.054	0.079	0.9	0.08	0.909
COPB1	YKT6	1843983	1842981	9606.ENSP00000249923	9606.ENSP00000223369	0	0	0	0	0.057	0.079	0.9	0.083	0.909
EGFR	AP2S1	1846445	1845323	9606.ENSP00000275493	9606.ENSP00000263270	0	0	0	0	0	0.12	0.9	0.058	0.909
RAB14	SNAP23	1855997	1843971	9606.ENSP00000362946	9606.ENSP00000249647	0	0	0	0	0.054	0	0.9	0.116	0.909
AP2M1	VAMP3	1847379	1842224	9606.ENSP00000292807	9606.ENSP00000054666	0	0	0	0	0.064	0	0.9	0.112	0.909
AP1G1	CLTA	1858105	1843621	9606.ENSP00000377148	9606.ENSP00000242285	0	0	0	0	0.054	0.054	0.9	0.108	0.909
AP2B1	MAPK1	1849703	1842512	9606.ENSP00000314414	9606.ENSP00000215832	0	0	0	0	0.117	0.054	0.9	0	0.909
SH3GL2	STAM	1857254	1856699	9606.ENSP00000369981	9606.ENSP00000366746	0	0	0	0.635	0.116	0	0.9	0.083	0.909
KDELR3	NAPA	1859221	1845337	9606.ENSP00000386918	9606.ENSP00000263354	0	0	0	0	0.055	0	0.9	0.117	0.909
DCTN1	COPB2	1854219	1851147	9606.ENSP00000354791	9606.ENSP00000329419	0	0	0	0	0.052	0	0.9	0.118	0.909
GBF1	TMED10	1855151	1848494	9606.ENSP00000359000	9606.ENSP00000303145	0	0	0	0	0.125	0	0.9	0	0.908
EXOSC4	RPS14	1849789	1849373	9606.ENSP00000315476	9606.ENSP00000311028	0	0	0	0	0.096	0	0.9	0.066	0.908
COPB2	AP2S1	1851147	1845323	9606.ENSP00000329419	9606.ENSP00000263270	0	0	0	0	0.054	0.054	0.9	0.102	0.908
PREB	CTSC	1844807	1843108	9606.ENSP00000260643	9606.ENSP00000227266	0	0	0	0	0	0	0.9	0.122	0.908
SH3GL2	AP2B1	1857254	1849703	9606.ENSP00000369981	9606.ENSP00000314414	0	0	0	0	0.057	0	0.9	0.104	0.908
COG2	BET1	1854425	1842924	9606.ENSP00000355629	9606.ENSP00000222547	0	0	0	0	0.075	0	0.9	0.092	0.908
RAB5A	ARF1	1846291	1846224	9606.ENSP00000273047	9606.ENSP00000272102	0	0	0	0	0.054	0.786	0	0.582	0.908
NAPA	COPE	1845337	1845219	9606.ENSP00000263354	9606.ENSP00000262812	0	0	0	0	0.119	0	0.9	0	0.908
GBF1	COPE	1855151	1845219	9606.ENSP00000359000	9606.ENSP00000262812	0	0	0	0	0.06	0	0.9	0.103	0.908
AP2M1	MAPK1	1847379	1842512	9606.ENSP00000292807	9606.ENSP00000215832	0	0	0	0	0.066	0	0.9	0.099	0.908
SH3GL2	RAB5A	1857254	1846291	9606.ENSP00000369981	9606.ENSP00000273047	0	0	0	0	0.053	0	0.9	0.111	0.908
EIF4E	RPS14	1861659	1849373	9606.ENSP00000425561	9606.ENSP00000311028	0	0	0	0	0.097	0	0.9	0.069	0.908
COPB2	NAPA	1851147	1845337	9606.ENSP00000329419	9606.ENSP00000263354	0	0	0	0	0.053	0.066	0.9	0.085	0.908
IGF2R	AP2B1	1853441	1849703	9606.ENSP00000349437	9606.ENSP00000314414	0	0	0	0	0.073	0	0.9	0.093	0.908
PARN	EIF4A3	1859310	1846086	9606.ENSP00000387911	9606.ENSP00000269349	0	0	0	0	0.055	0	0.9	0.112	0.908
ARFGAP1	AP2M1	1849722	1847379	9606.ENSP00000314615	9606.ENSP00000292807	0	0	0	0	0.066	0.071	0.9	0.065	0.908
IGF2R	AP2M1	1853441	1847379	9606.ENSP00000349437	9606.ENSP00000292807	0	0	0	0	0	0	0.9	0.127	0.908
SH3GL2	RPS6KA3	1857254	1857057	9606.ENSP00000369981	9606.ENSP00000368884	0	0	0	0	0.053	0.088	0.9	0.069	0.908
AP2S1	VAMP7	1845323	1845189	9606.ENSP00000263270	9606.ENSP00000262640	0	0	0	0	0.098	0	0.9	0.066	0.908
STX12	GOSR2	1856019	1843032	9606.ENSP00000363054	9606.ENSP00000225567	0	0	0	0	0.073	0.768	0.54	0.184	0.908
EIF4G1	RPS14	1852064	1849373	9606.ENSP00000338020	9606.ENSP00000311028	0	0	0	0	0	0	0.9	0.122	0.908
YIPF6	AP3B1	1861304	1844363	9606.ENSP00000417573	9606.ENSP00000255194	0	0	0	0	0.123	0	0.9	0.044	0.908
YIPF6	SNX2	1861304	1857048	9606.ENSP00000417573	9606.ENSP00000368831	0	0	0	0	0.062	0.071	0.9	0.07	0.908
SEC24D	NAPA	1846673	1845337	9606.ENSP00000280551	9606.ENSP00000263354	0	0	0	0	0.054	0	0.9	0.101	0.907
CNOT4	EIF4A1	1862294	1847443	9606.ENSP00000445508	9606.ENSP00000293831	0	0	0	0	0.054	0.05	0.9	0.087	0.907
AP2M1	VAMP4	1847379	1843436	9606.ENSP00000292807	9606.ENSP00000236192	0	0	0	0	0.064	0	0.9	0.09	0.907
RPS6KA3	AP2B1	1857057	1849703	9606.ENSP00000368884	9606.ENSP00000314414	0	0	0	0	0.107	0	0.9	0	0.907
COG2	COPB2	1854425	1851147	9606.ENSP00000355629	9606.ENSP00000329419	0	0	0	0	0.116	0	0.9	0	0.907
SH3GL2	CLTA	1857254	1843621	9606.ENSP00000369981	9606.ENSP00000242285	0	0	0	0	0.055	0	0.9	0.095	0.907
RPS6KA3	CLTC	1857057	1846064	9606.ENSP00000368884	9606.ENSP00000269122	0	0	0	0	0.094	0	0.9	0.056	0.907
GBF1	YKT6	1855151	1842981	9606.ENSP00000359000	9606.ENSP00000223369	0	0	0	0	0.055	0.054	0.9	0.088	0.907
TMED10	YKT6	1848494	1842981	9606.ENSP00000303145	9606.ENSP00000223369	0	0	0	0	0.057	0.054	0.9	0.087	0.907
TMED2	YKT6	1845096	1842981	9606.ENSP00000262225	9606.ENSP00000223369	0	0	0	0	0.058	0.054	0.9	0.088	0.907
OCRL	VAMP3	1855405	1842224	9606.ENSP00000360154	9606.ENSP00000054666	0	0	0	0	0.093	0	0.9	0.066	0.907
SSR1	SPCS1	1843739	1843331	9606.ENSP00000244763	9606.ENSP00000233025	0	0	0	0	0.115	0	0.9	0	0.907
SNX2	NAPA	1857048	1845337	9606.ENSP00000368831	9606.ENSP00000263354	0	0	0	0	0.084	0	0.9	0.061	0.906
EIF4A2	CNOT2	1850828	1843182	9606.ENSP00000326381	9606.ENSP00000229195	0	0	0	0	0.065	0	0.9	0.08	0.906
AP2M1	VAMP7	1847379	1845189	9606.ENSP00000292807	9606.ENSP00000262640	0	0	0	0	0.064	0	0.9	0.083	0.906
DCTN1	ARF1	1854219	1846224	9606.ENSP00000354791	9606.ENSP00000272102	0	0	0	0	0.052	0.061	0.9	0.074	0.906
KIF1B	COPB1	1845440	1843983	9606.ENSP00000263934	9606.ENSP00000249923	0	0	0	0	0.053	0.062	0.9	0.068	0.906
PAIP1	CNOT2	1848447	1843182	9606.ENSP00000302768	9606.ENSP00000229195	0	0	0	0	0.1	0	0.9	0	0.906
GOLGA4	NAPG	1853422	1850661	9606.ENSP00000349305	9606.ENSP00000324628	0	0	0	0	0.107	0	0.9	0	0.906

EIF4G1	CNOT2	1852064	1843182	9606.ENSP00000338020	9606.ENSP00000229195	0	0	0	0	0.054	0	0.9	0.091	0.906
COPE	YKT6	1845219	1842981	9606.ENSP00000262812	9606.ENSP00000223369	0	0	0	0	0.054	0.07	0.9	0.065	0.906
EGFR	VAMP7	1846445	1845189	9606.ENSP00000275493	9606.ENSP00000262640	0	0	0	0	0	0	0.9	0.1	0.906
KIF5B	TMED2	1848911	1845096	9606.ENSP00000307078	9606.ENSP00000262225	0	0	0	0	0.099	0	0.9	0	0.906
EXOSC1	RPS14	1855369	1849373	9606.ENSP00000359939	9606.ENSP00000311028	0	0	0	0	0.099	0	0.9	0.046	0.906
COG2	YKT6	1854425	1842981	9606.ENSP00000355629	9606.ENSP00000223369	0	0	0	0	0.052	0	0.9	0.093	0.906
COG2	ARCN1	1854425	1845462	9606.ENSP00000355629	9606.ENSP00000264028	0	0	0	0	0.09	0	0.9	0	0.905
USO1	COPE	1862154	1845219	9606.ENSP00000440586	9606.ENSP00000262812	0	0	0	0	0.073	0	0.9	0.064	0.905
KIF5B	ZW10	1848911	1842389	9606.ENSP00000307078	9606.ENSP00000200135	0	0	0	0	0.054	0	0.9	0.081	0.905
SNX2	OCRL	1857048	1855405	9606.ENSP00000368831	9606.ENSP00000360154	0	0	0	0	0.055	0	0.9	0.076	0.905
OCRL	VAMP7	1855405	1845189	9606.ENSP00000360154	9606.ENSP00000262640	0	0	0	0	0.072	0	0.9	0.066	0.905
GBF1	KIF5B	1855151	1848911	9606.ENSP00000359000	9606.ENSP00000307078	0	0	0	0	0.096	0	0.9	0	0.905
OCRL	VAMP4	1855405	1843436	9606.ENSP00000360154	9606.ENSP00000236192	0	0	0	0	0.072	0	0.9	0.066	0.905
SH3GL2	VAMP4	1857254	1843436	9606.ENSP00000369981	9606.ENSP00000236192	0	0	0	0	0.055	0	0.9	0.082	0.905
RPS14	SPCS1	1849373	1843331	9606.ENSP00000311028	9606.ENSP00000233025	0	0	0	0	0.094	0	0.9	0	0.905
AP2S1	VAMP4	1845323	1843436	9606.ENSP00000263270	9606.ENSP00000236192	0	0	0	0	0.068	0	0.9	0.066	0.905
RPS14	SKIV2L2	1849373	1843261	9606.ENSP00000311028	9606.ENSP00000230640	0	0	0	0	0.054	0	0.9	0.081	0.905
EXOSC10	IMP3	1856580	1850884	9606.ENSP00000366135	9606.ENSP00000326981	0	0	0	0	0.074	0	0.9	0.063	0.905
CEBPB	HSP90B1	1848728	1848102	9606.ENSP00000305422	9606.ENSP00000299767	0	0	0	0	0	0	0.9	0.089	0.905
KIF5B	TMED10	1848911	1848494	9606.ENSP00000307078	9606.ENSP00000303145	0	0	0	0	0.088	0	0.9	0	0.905
AP2B1	M6PR	1849703	1842110	9606.ENSP00000314414	9606.ENSP00000000412	0	0	0	0	0	0	0.9	0.091	0.905
OCRL	NAPA	1855405	1845337	9606.ENSP00000360154	9606.ENSP00000263354	0	0	0	0	0.074	0.048	0.9	0.05	0.905
AP2S1	VAMP3	1845323	1842224	9606.ENSP00000263270	9606.ENSP00000054666	0	0	0	0	0.068	0	0.9	0.066	0.905
GBF1	TMED2	1855151	1845096	9606.ENSP00000359000	9606.ENSP00000262225	0	0	0	0	0	0.08	0.9	0.049	0.904
USO1	KDELR3	1862154	1859221	9606.ENSP00000440586	9606.ENSP00000386918	0	0	0	0	0.047	0	0.9	0.081	0.904
STX16	COG2	1855414	1854425	9606.ENSP00000360183	9606.ENSP00000355629	0	0	0	0	0.054	0	0.9	0.068	0.904
LSM4	FUS	1864161	1844273	9606.ENSP00000469468	9606.ENSP00000254108	0	0	0	0	0.082	0	0.9	0	0.904
AP3S1	NAPA	1850729	1845337	9606.ENSP00000325369	9606.ENSP00000263354	0	0	0	0	0.073	0	0.9	0.05	0.904
YIPF6	AP1G1	1861304	1858105	9606.ENSP00000417573	9606.ENSP00000377148	0	0	0	0	0.069	0	0.9	0.052	0.904
SH3GL2	AP2S1	1857254	1845323	9606.ENSP00000369981	9606.ENSP00000263270	0	0	0	0	0	0	0.9	0.081	0.904
CLTA	MAPK1	1843621	1842512	9606.ENSP00000242285	9606.ENSP00000215832	0	0	0	0	0	0.049	0.9	0.075	0.904
COG2	VAMP4	1854425	1843436	9606.ENSP00000355629	9606.ENSP00000236192	0	0	0	0	0.047	0	0.9	0.083	0.904
KIF1B	ZW10	1845440	1842389	9606.ENSP00000263934	9606.ENSP00000200135	0	0	0	0	0.054	0	0.9	0.07	0.904
EIF4A1	CNOT2	1847443	1843182	9606.ENSP00000293831	9606.ENSP00000229195	0	0	0	0	0.065	0	0.9	0.061	0.904
EIF4A3	FUS	1846086	1844273	9606.ENSP00000269349	9606.ENSP00000254108	0	0	0	0	0.054	0	0.9	0.074	0.904
COPB2	KIF5B	1851147	1848911	9606.ENSP00000329419	9606.ENSP00000307078	0	0	0	0	0.086	0	0.9	0	0.904
DCP2	EXOSC2	1857792	1855666	9606.ENSP00000373715	9606.ENSP00000361433	0	0	0	0	0.053	0	0.9	0.067	0.904
USO1	DCTN1	1862154	1854219	9606.ENSP00000440586	9606.ENSP00000354791	0	0	0	0	0.058	0	0.9	0.065	0.904
ATF3	XBP1	1852803	1842534	9606.ENSP00000344352	9606.ENSP00000216037	0	0	0	0	0.069	0	0.8	0.526	0.904
DCTN1	BET1	1854219	1842924	9606.ENSP00000354791	9606.ENSP00000222547	0	0	0	0	0	0	0.9	0.087	0.904
ARCN1	ZW10	1845462	1842389	9606.ENSP00000264028	9606.ENSP00000200135	0	0	0	0	0.087	0	0.9	0	0.904
RAB5A	AP2S1	1846291	1845323	9606.ENSP00000273047	9606.ENSP00000263270	0	0	0	0	0.058	0	0.9	0.066	0.904
EXOSC2	RPS14	1855666	1849373	9606.ENSP00000361433	9606.ENSP00000311028	0	0	0	0	0.085	0	0.9	0	0.904
OCRL	AP3S1	1855405	1850729	9606.ENSP00000360154	9606.ENSP00000325369	0	0	0	0	0.05	0	0.9	0.065	0.903
ARFGAP1	AP2S1	1849722	1845323	9606.ENSP00000314615	9606.ENSP00000263270	0	0	0	0	0.054	0.061	0.9	0	0.903
DNAJC3	EIF2AK3	1856557	1848934	9606.ENSP00000365991	9606.ENSP00000307235	0	0	0	0	0.049	0.36	0.8	0.297	0.903
CLTA	GOSR2	1843621	1843032	9606.ENSP00000242285	9606.ENSP00000225567	0	0	0	0	0.064	0	0.9	0.055	0.903
ARFGAP1	KIF5B	1849722	1848911	9606.ENSP00000314615	9606.ENSP00000307078	0	0	0	0	0.073	0	0.9	0.043	0.903
CNOT4	EIF4A3	1862294	1846086	9606.ENSP00000445508	9606.ENSP00000269349	0	0	0	0	0.054	0.05	0.9	0.05	0.903
NAPA	AP3B1	1845337	1844363	9606.ENSP00000263354	9606.ENSP00000255194	0	0	0	0	0.054	0	0.9	0.063	0.903
KHSRP	YWHAZ	1858577	1849183	9606.ENSP00000381216	9606.ENSP00000309503	0	0	0	0	0.073	0	0.9	0	0.903
COG2	GOLGA4	1854425	1853422	9606.ENSP00000355629	9606.ENSP00000349305	0	0	0	0	0.07	0	0.9	0	0.903
AP3S1	AP2S1	1850729	1845323	9606.ENSP00000325369	9606.ENSP00000263270	0	0	0	0.907	0.057	0	0.9	0.255	0.903
SH3GL2	VAMP7	1857254	1845189	9606.ENSP00000369981	9606.ENSP00000262640	0	0	0	0	0.053	0	0.9	0.061	0.903
IGF2R	NAPG	1853441	1850661	9606.ENSP00000349437	9606.ENSP00000324628	0	0	0	0	0.075	0	0.9	0	0.903
CNOT4	EIF4A2	1862294	1850828	9606.ENSP00000445508	9606.ENSP00000326381	0	0	0	0	0.054	0.05	0.9	0.056	0.903
DCTN1	GOSR2	1854219	1843032	9606.ENSP00000354791	9606.ENSP00000225567	0	0	0	0	0	0	0.9	0.074	0.903
NAPA	COPB1	1845337	1843983	9606.ENSP00000263354	9606.ENSP00000249923	0	0	0	0	0.053	0.042	0.9	0.063	0.903
AP2S1	M6PR	1845323	1842110	9606.ENSP00000263270	9606.ENSP00000000412	0	0	0	0	0	0	0.9	0.075	0.903
RPS6KA3	AP2M1	1857057	1847379	9606.ENSP00000368884	9606.ENSP00000292807	0	0	0	0	0.054	0.063	0.9	0	0.903
IGF2R	AP2S1	1853441	1845323	9606.ENSP00000349437	9606.ENSP00000263270	0	0	0	0	0	0	0.9	0.078	0.903
KIF1B	COPE	1845440	1845219	9606.ENSP00000263934	9606.ENSP00000262812	0	0	0	0	0	0	0.9	0.076	0.903
CNOT4	PAIP1	1862294	1848447	9606.ENSP00000445508	9606.ENSP00000302768	0	0	0	0	0.073	0	0.9	0	0.903
NAPA	TMED2	1845337	1845096	9606.ENSP00000263354	9606.ENSP00000262225	0	0	0	0	0.053	0	0.9	0.065	0.903
TMED10	CTSC	1848494	1843108	9606.ENSP00000303145	9606.ENSP00000227266	0	0	0	0	0.053	0	0.9	0.052	0.902
SH3GL2	MAPK1	1857254	1842512	9606.ENSP00000369981	9606.ENSP00000215832	0	0	0	0	0.053	0	0.9	0.054	0.902
KHSRP	EXOSC1	1858577	1855369	9606.ENSP00000381216	9606.ENSP00000359939	0	0	0	0	0	0	0.9	0.061	0.902
KIF5B	ARFGAP3	1848911	1845314	9606.ENSP00000307078	9606.ENSP00000263245	0	0	0	0	0.064	0	0.9	0	0.902
SGMS1	GALC	1854226	1844883	9606.ENSP00000354829	9606.ENSP00000261304	0	0	0	0	0	0	0.9	0.06	0.902
SH3GL2	SNX2	1857254	1857048	9606.ENSP00000369981	9606.ENSP00000368831	0	0	0	0	0	0	0.9	0.061	0.902
EGFR	VAMP4	1846445	1843436	9606.ENSP00000275493	9606.ENSP00000236192	0	0	0	0	0	0	0.9	0.061	0.902
GBF1	CLTA	1855151	1843621	9606.ENSP00000359000	9606.ENSP00000242285	0	0	0	0	0	0	0.9	0.06	0.902
AP1G1	M6PR	1858105	1842110	9606.ENSP00000377148	9606.ENSP00000000412	0	0	0	0	0	0	0.9	0.064	0.902
DCTN1	YKT6	1854219	1842981	9606.ENSP00000354791	9606.ENSP00000223369	0	0	0	0	0.047	0	0.9	0.054	0.902
GBF1	KIF1B	1855151	1845440	9606.ENSP00000359000	9606.ENSP00000263934	0	0	0	0	0.066	0	0.9	0	0.902
SEC31A	AP2S1	1853159	1845323	9606.ENSP00000347329	9606.ENSP00000263270	0	0	0	0	0.061	0	0.9	0.041	0.902
KIF5B	ARCN1	1848911	1845462	9606.ENSP00000307078	9606.ENSP00000264028	0	0	0	0	0.067	0	0.9	0	0.902
ARCN1	KIF1B	1845462	1845440	9606.ENSP00000264028	9606.ENSP00000263934	0	0	0	0	0.067	0	0.9	0	0.902
DCTN1	ARFGAP1	1854219	1849722	9606.ENSP00000354791	9606.ENSP00000314615	0	0	0	0	0.054	0	0.9	0.048	0.902
COG2	NAPG	1854425	1850661	9606.ENSP00000355629	9606.ENSP00000324628	0	0	0	0	0.064	0	0.9	0	0.902
YIPF6	AP3S1	1861304	1850729	9606.ENSP00000417573	9606.ENSP00000325369	0	0	0	0	0.064	0	0.9	0	0.902
COG2	COPE	1854425	1845219	9606.ENSP00000355629	9606.ENSP00000262812	0	0	0	0	0.062	0	0.9	0	0.902
SEC24D	AP2S1	1846673	1845323	9606.ENSP00000280551	9606.ENSP00000263270	0	0	0	0	0.061	0	0.9	0	0.902
YIPF6	IGF2R	1861304	1853441	9606.ENSP00000417573	9606.ENSP00000349437	0	0	0	0	0.068	0	0.9	0	0.902
EIF4A3	CNOT2	1846086	1843182	9606.ENSP00000269349	9606.ENSP00000229195	0	0	0	0	0.054	0	0.9	0.044	0.901
TPD52	VAMP7	1856977	1845189	9606.ENSP00000368391	9606.ENSP00000262640	0	0	0	0	0.053	0	0.9	0	0.901
CLTA	M6PR	1843621	1842110	9606.ENSP00000242285	9606.ENSP00000000412	0	0	0	0	0.054	0	0.9	0	0.901
IGF2R	CLTA	1853441	1843621	9606.ENSP00000349437	9606.ENSP00000242285	0	0	0	0	0	0.054	0.9	0	0.901
EXOSC9	RPS14	1857072	1849373	9606.ENSP00000368984	9606.ENSP00000311028	0	0	0	0	0.053	0	0.9	0	0.901
KIF1B	ARFGAP3	1845440	1845314	9606.ENSP00000263934	9606.ENSP00000263245	0	0	0	0	0.055	0	0.9	0	0.901
NAPG	SEC24D	1850661	1846673	9606.ENSP00000324628	9606.ENSP00000280551	0	0	0	0	0	0	0.9	0.055	0.901
TMED10	NAPA	1848494	1845337	9606.ENSP00000303145	9606.ENSP00000263354	0	0	0	0	0.053	0	0.9	0	0.901
DCTN1	COPB1	1854219	1843983	9606.ENSP00000354791	9606.ENSP00000249923	0	0	0	0	0.054	0	0.9	0.041	0.901
USO1	CLTA	1862154	1843621	9606.ENSP00000440586	9606.ENSP00000242285	0	0	0	0	0	0	0.9	0.056	0.901
IGF2R	CLTC	1853441	1846064	9606.ENSP00000349437	9606.ENSP00000269122	0	0	0	0	0	0	0.9	0.058	0.901
AP1G1	NAPA	1858105	1845337	9606.ENSP00000377148	9606.ENSP00000263354	0	0	0	0	0.055	0	0.9	0	0.901
SEC24D	CTSC	1846673	1843108	9606.ENSP00000280551	9606.ENSP00000227266	0	0	0	0	0.046	0	0.9	0.046	0.901
TPD52	NAPA	1856977	1845337	9606.ENSP00000368391	9606.ENSP00000263354	0	0	0	0	0.055	0	0.9	0	0.901
USO1	CTSC	1862154	1843108	9606.ENSP00000440586	9606.ENSP00000227266	0	0	0	0	0	0	0.9	0.057	0.901
KDELR3	ZW10	1859221	1842389	9606.ENSP00000386918	9606.ENSP00000200135	0	0	0	0	0	0	0.9	0.051	0.901
NAPA	ZW10	1845337	1842389	9606.ENSP00000263354	9606.ENSP00000200135	0	0	0	0	0.051	0	0.9	0	0.901
RPS6KA3	AP2S1	1857057	1845323	9606.ENSP00000368884	9606.ENSP00000263270	0	0	0	0	0.053	0	0.9	0	0.901
CTSC	YKT6	1843108	1842981	9606.ENSP00000227266	9606.ENSP00000223369	0	0	0	0	0.051	0	0.9	0	0.901
YIPF6	NAPA	1861304	1845337	9606.ENSP00000417573	9606.ENSP00000263354	0	0	0	0	0.057	0	0.9	0	0.901
CLTC	M6PR	1846064	1842110	9606.ENSP00000269122	9606.ENSP00000000412	0	0	0	0	0	0	0.9	0.058	0.901
ATF6	WFS1	1854690	1843097	9606.ENSP00000356919	9606.ENSP00000226760	0	0	0	0	0	0.285	0.8	0.364	0.901
DCTN1	ARFGAP3	1854219	1845314	9606.ENSP00000354791	9606.ENSP00000263245	0	0	0	0	0.054	0	0.9	0.042	0.901
RPS14	SSR1	1849373	1843739	9606.ENSP00000311028	9606.ENSP00000244763	0	0	0	0	0.058	0	0.9	0	0.901
AP1G1	SH3GL2	1858105	1857254	9606.ENSP00000377148	9606.ENSP00000369981	0	0	0	0	0.056	0	0.9	0	0.901
COG2	TMED10	1854425	1848494	9606.ENSP00000355629	9606.ENSP00000303145	0	0	0	0	0.054	0	0.9	0	0.901
KDELR3	CLTA	1859221	1843621	9606.ENSP00000386918	9606.ENSP00000242285	0	0	0	0	0.054	0	0.9	0	0.901
SH3GL2	AP3B1	1857254	1844363	9606.ENSP00000369981	9606.ENSP00000255194	0	0	0	0	0.057	0	0.9	0	0.901
FUS	SKIV2L2	1844273	1843261	9606.ENSP00000254108	9606.ENSP00000230640	0	0	0	0	0	0	0.9	0.059	0.901
TPD52	ARF1	1856977	1846224	9606.ENSP00000368391	9606.ENSP00000272102	0	0	0	0	0.052	0	0.9	0	0.901
DCTN1	ARCN1	1854219	1845462	9606.ENSP00000354791	9606.ENSP00000264028	0	0	0	0	0.042	0	0.9	0	0.9
EXOSC10	RPS14	1856580	1849373	9606.ENSP00000366135	9606.ENSP00000311028	0	0	0	0	0	0	0.9	0	0.9
USO1	NAPG	1862154	1850661	9606.ENSP00000440586	9606.ENSP00000324628	0	0	0	0	0	0	0.9	0	0.9
KDELR3	KIF1B	1859221	1845440	9606.ENSP00000386918	9606.ENSP00000263934	0	0	0	0	0	0	0.9	0	0.9
IGF2R	NAPA	1853441	1845337	9606.ENSP00000349437	9606.ENSP00000263354	0	0	0	0	0	0	0.9	0	0.9
DCTN1	TMED10	1854219	1848494	9606.ENSP00000354791	9606.ENSP00000303145	0	0	0	0	0	0	0.9	0	0.9
KDELR3	NAPG	1859221	1850661	9606.ENSP00000386918	9606.ENSP00000324628	0	0	0	0	0	0	0.9	0	0.9
STX12	STX7	1856019	1854689	9606.ENSP00000363054	9606.ENSP00000356918	0	0	0	0.942	0.074	0.774	0.54	0.861	0.9
NAPG	ARCN1	1850661	1845462	9606.ENSP00000324628	9606.ENSP00000264028	0	0	0	0	0	0	0.9	0	0.9
TMED10	ZW10	1848494	1842389	9606.ENSP00000303145	9606.ENSP00000200135	0	0	0	0	0	0	0.9	0	0.9
TPD52	AP3B1	1856977	1844363	9606.ENSP00000368391	9606.ENSP00000255194	0	0	0	0	0	0	0.9	0	0.9
NAPA	M6PR	1845337	1842110	9606.ENSP00000263354	9606.ENSP00000000412	0	0	0	0	0	0	0.9	0	0.9
TMED2	CTSC	1845096	1843108	9606.ENSP00000262225	9606.ENSP00000227266	0	0	0	0	0	0	0.9	0.047	0.9
SH3GL2	AP3S1	1857254	1850729	9606.ENSP00000369981	9606.ENSP00000325369	0	0	0	0	0	0	0.9	0.049	0.9
KDELR3	COG2	1859221	1854425	9606.ENSP00000386918	9606.ENSP00000355629	0	0	0	0	0	0	0.9	0	0.9
SNX2	TPD52	1857048	1856977	9606.ENSP00000368831	9606.ENSP00000368391	0	0	0	0	0	0	0.9	0	0.9
SHC1	SGMS1	1860266	1854226	9606.ENSP00000401303	9606.ENSP00000354829	0	0	0	0	0	0	0.9	0.046	0.9
ARFGAP1	KIF1B	1849722	1845440	9606.ENSP00000314615	9606.ENSP00000263934	0	0	0	0	0.047	0	0.9	0	0.9
YIPF6	SH3GL2	1861304	1857254	9606.ENSP00000417573	9606.ENSP00000369981	0	0	0	0	0	0	0.9	0	0.9
SH3GL2	IGF2R	1857254	1853441	9606.ENSP00000369981	9606.ENSP00000349437	0	0	0	0	0	0	0.9	0.046	0.9
RPS6KA3	CLTA	1857057	1843621	9606.ENSP00000368884	9606.ENSP00000242285	0	0	0	0	0	0	0.9	0	0.9
COG2	DCTN1	1854425	1854219	9606.ENSP00000355629	9606.ENSP00000354791	0	0	0	0	0	0	0.9	0	0.9
KDELR3	DCTN1	1859221	1854219	9606.ENSP00000386918	9606.ENSP00000354791	0	0	0	0	0	0	0.9	0	0.9
GOLGA4	NAPA	1853422	1845337	9606.ENSP00000349305	9606.ENSP00000263354	0	0	0	0	0	0	0.9	0	0.9
NAPG	COPE	1850661	1845219	9606.ENSP00000324628	9606.ENSP00000262812	0	0	0	0	0	0	0.9	0	0.9
NAPG	TMED2	1850661	1845096	9606.ENSP00000324628	9606.ENSP00000262225	0	0	0	0	0	0	0.9	0	0.9
TMED10	KIF1B	1848494	1845440	9606.ENSP00000303145	9606.ENSP00000263934	0	0	0	0	0	0	0.9	0	0.9
COPB2	NAPG	1851147	1850661	9606.ENSP00000329419	9606.ENSP00000324628	0	0	0	0	0	0	0.9	0.043	0.9
NAPG	ZW10	1850661	1842389	9606.ENSP00000324628	9606.ENSP00000200135	0	0	0	0	0	0	0.9	0	0.9
AP1G1	TPD52	1858105	1856977	9606.ENSP00000377148	9606.ENSP00000368391	0	0	0	0	0	0	0.9	0	0.9
TPD52	OCRL	1856977	1855405	9606.ENSP00000368391	9606.ENSP00000360154	0	0	0	0	0	0	0.9	0	0.9
SH3GL2	M6PR	1857254	1842110	9606.ENSP00000369981	9606.ENSP00000000412	0	0	0	0	0	0	0.9	0	0.9
NAPG	M6PR	1850661	1842110	9606.ENSP00000324628	9606.ENSP00000000412	0	0	0	0	0	0	0.9	0	0.9
NAPG	LMAN1	1850661	1844034	9606.ENSP00000324628	9606.ENSP00000251047	0	0	0	0	0	0	0.9	0	0.9
SEC31A	CTSC	1853159	1843108	9606.ENSP00000347329	9606.ENSP00000227266	0	0	0	0	0.049	0	0.9	0	0.9
NAPG	COPB1	1850661	1843983	9606.ENSP00000324628	9606.ENSP00000249923	0	0	0	0	0	0	0.9	0	0.9
NAPG	CTSC	1850661	1843108	9606.ENSP00000324628	9606.ENSP00000227266	0	0	0	0	0	0	0.9	0	0.9
CTSC	BET1	1843108	1842924	9606.ENSP00000227266	9606.ENSP00000222547	0	0	0	0	0	0	0.9	0	0.9
DCTN1	COPE	1854219	1845219	9606.ENSP00000354791	9606.ENSP00000262812	0	0	0	0	0.049	0	0.9	0	0.9
DCTN1	TMED2	1854219	1845096	9606.ENSP00000354791	9606.ENSP00000262225	0	0	0	0	0	0	0.9	0	0.9
RAB9A	NAPG	1861457	1850661	9606.ENSP00000420127	9606.ENSP00000324628	0	0	0	0	0	0	0.9	0	0.9
KDELR3	KIF5B	1859221	1848911	9606.ENSP00000386918	9606.ENSP00000307078	0	0	0	0	0	0	0.9	0	0.9
SRPRB	RPS14	1861356	1849373	9606.ENSP00000418401	9606.ENSP00000311028	0	0	0	0	0	0	0.9	0	0.9
KIF1B	TMED2	1845440	1845096	9606.ENSP00000263934	9606.ENSP00000262225	0	0	0	0	0	0	0.9	0	0.9
SH3GL2	TPD52	1857254	1856977	9606.ENSP00000369981	9606.ENSP00000368391	0	0	0	0	0	0	0.9	0	0.9
NAPG	TMED10	1850661	1848494	9606.ENSP00000324628	9606.ENSP00000303145	0	0	0	0	0	0	0.9	0	0.9
YIPF6	TPD52	1861304	1856977	9606.ENSP00000417573	9606.ENSP00000368391	0	0	0	0	0	0	0.9	0	0.9
CTSC	GOSR2	1843108	1843032	9606.ENSP00000227266	9606.ENSP00000225567	0	0	0	0	0	0	0.9	0	0.9
ARCN1	NAPA	1845462	1845337	9606.ENSP00000264028	9606.ENSP00000263354	0	0	0	0	0.044	0	0.9	0.042	0.9
COPB2	KIF1B	1851147	1845440	9606.ENSP00000329419	9606.ENSP00000263934	0	0	0	0	0.047	0	0.9	0	0.9
COG2	NAPA	1854425	1845337	9606.ENSP00000355629	9606.ENSP00000263354	0	0	0	0	0.047	0	0.9	0	0.9
TPD52	AP3S1	1856977	1850729	9606.ENSP00000368391	9606.ENSP00000325369	0	0	0	0	0	0	0.9	0	0.9
NAPA	LMAN1	1845337	1844034	9606.ENSP00000263354	9606.ENSP00000251047	0	0	0	0	0	0	0.9	0.044	0.9
COG2	TMED2	1854425	1845096	9606.ENSP00000355629	9606.ENSP00000262225	0	0	0	0	0.049	0	0.9	0	0.9
IGF2R	AP3S1	1853441	1850729	9606.ENSP00000349437	9606.ENSP00000325369	0	0	0	0	0	0	0.9	0.044	0.9
COPE	ZW10	1845219	1842389	9606.ENSP00000262812	9606.ENSP00000200135	0	0	0	0	0.05	0	0.9	0	0.9
TMED2	ZW10	1845096	1842389	9606.ENSP00000262225	9606.ENSP00000200135	0	0	0	0	0	0	0.9	0	0.9
SPCS3	RPS14	1861710	1849373	9606.ENSP00000427463	9606.ENSP00000311028	0	0	0	0	0	0	0.9	0	0.9
NAPA	CTSC	1845337	1843108	9606.ENSP00000263354	9606.ENSP00000227266	0	0	0	0	0.047	0	0.9	0	0.9
H2AFX	CEBPB	1856252	1848728	9606.ENSP00000364310	9606.ENSP00000305422	0	0	0	0	0	0	0.9	0.048	0.9
SEC31A	CLTC	1853159	1846064	9606.ENSP00000347329	9606.ENSP00000269122	0	0	0	0	0.778	0	0.36	0.356	0.9
TPD52	IGF2R	1856977	1853441	9606.ENSP00000368391	9606.ENSP00000349437	0	0	0	0	0	0	0.9	0	0.9
