# Supplementary material for: Meta-Analysis of Polymyositis and Dermatomyositis Microarray Data Reveals Novel Genetic Biomarkers
Source: Genes (Basel). 2019 Oct 30;10(11):864. doi: 10.3390/genes10110864 (PMC6895911; doi:10.3390/genes10110864)
Supplement: Supplementary file 1 [file genes-10-00864-s001.zip › Supplementary_files/Supplementary Table 3.pdf]

Supplementary Table 3. Detected SNPs and eGenes in muscle tissue.

gene	SNP	GTEX_p_value	p_value	rs_id_dbSNP142_GRCh37p13	external_gene_name	description
ENSG00000178252	3_49878264_C_G_b37	1.81E-05	2.00E-06	rs1996663	WDR6	WD repeat domain 6 [Source:HGNC Symbol;Acc:12758]
ENSG00000145020	3_49878264_C_G_b37	5.07E-10	2.00E-06	rs1996663	AMT	aminomethyltransferase [Source:HGNC Symbol;Acc:473]
ENSG00000185614	3_49878264_C_G_b37	4.22E-06	2.00E-06	rs1996663	FAM212A	family with sequence similarity 212, member A [Source:HGNC Symbol;Acc:32480]
ENSG00000178252	3_49868876_A_T_b37	1.79E-05	3.00E-06	rs7649428	WDR6	WD repeat domain 6 [Source:HGNC Symbol;Acc:12758]
ENSG00000145020	3_49868876_A_T_b37	6.64E-10	3.00E-06	rs7649428	AMT	aminomethyltransferase [Source:HGNC Symbol;Acc:473]
ENSG00000185614	3_49868876_A_T_b37	4.98E-06	3.00E-06	rs7649428	FAM212A	family with sequence similarity 212, member A [Source:HGNC Symbol;Acc:32480]
ENSG00000178252	3_49853796_T_A_b37	1.78E-05	5.00E-06	rs62260720	WDR6	WD repeat domain 6 [Source:HGNC Symbol;Acc:12758]
ENSG00000178252	3_49871201_C_T_b37	1.81E-05	5.00E-06	rs61557789	WDR6	WD repeat domain 6 [Source:HGNC Symbol;Acc:12758]
ENSG00000145020	3_49853796_T_A_b37	4.80E-10	5.00E-06	rs62260720	AMT	aminomethyltransferase [Source:HGNC Symbol;Acc:473]
ENSG00000145020	3_49871201_C_T_b37	5.07E-10	5.00E-06	rs61557789	AMT	aminomethyltransferase [Source:HGNC Symbol;Acc:473]
ENSG00000185614	3_49853796_T_A_b37	4.27E-06	5.00E-06	rs62260720	FAM212A	family with sequence similarity 212, member A [Source:HGNC Symbol;Acc:32480]
ENSG00000185614	3_49871201_C_T_b37	4.22E-06	5.00E-06	rs61557789	FAM212A	family with sequence similarity 212, member A [Source:HGNC Symbol;Acc:32480]
ENSG00000178252	3_49848414_C_T_b37	3.42E-05	6.00E-06	rs28535523	WDR6	WD repeat domain 6 [Source:HGNC Symbol;Acc:12758]
ENSG00000178252	3_49872124_T_C_b37	1.81E-05	6.00E-06	rs62260723	WDR6	WD repeat domain 6 [Source:HGNC Symbol;Acc:12758]
ENSG00000145020	3_49848414_C_T_b37	1.85E-10	6.00E-06	rs28535523	AMT	aminomethyltransferase [Source:HGNC Symbol;Acc:473]
ENSG00000145020	3_49872124_T_C_b37	5.07E-10	6.00E-06	rs62260723	AMT	aminomethyltransferase [Source:HGNC Symbol;Acc:473]
ENSG00000235261	3_49848414_C_T_b37	6.22E-05	6.00E-06	rs28535523	NICN1-AS1	NICN1 antisense RNA 1 [Source:HGNC Symbol;Acc:40838]
ENSG00000185614	3_49848414_C_T_b37	2.49E-06	6.00E-06	rs28535523	FAM212A	family with sequence similarity 212, member A [Source:HGNC Symbol;Acc:32480]
ENSG00000185614	3_49872124_T_C_b37	4.22E-06	6.00E-06	rs62260723	FAM212A	family with sequence similarity 212, member A [Source:HGNC Symbol;Acc:32480]
ENSG00000178252	3_49878395_A_G_b37	1.81E-05	7.00E-06	rs1996664	WDR6	WD repeat domain 6 [Source:HGNC Symbol;Acc:12758]
ENSG00000178252	3_49886319_G_T_b37	3.91E-05	7.00E-06	rs77941694	WDR6	WD repeat domain 6 [Source:HGNC Symbol;Acc:12758]
ENSG00000145020	3_49878395_A_G_b37	5.07E-10	7.00E-06	rs1996664	AMT	aminomethyltransferase [Source:HGNC Symbol;Acc:473]
ENSG00000145020	3_49886319_G_T_b37	2.49E-10	7.00E-06	rs77941694	AMT	aminomethyltransferase [Source:HGNC Symbol;Acc:473]
ENSG00000185614	3_49878395_A_G_b37	4.22E-06	7.00E-06	rs1996664	FAM212A	family with sequence similarity 212, member A [Source:HGNC Symbol;Acc:32480]
ENSG00000185614	3_49886319_G_T_b37	9.78E-06	7.00E-06	rs77941694	FAM212A	family with sequence similarity 212, member A [Source:HGNC Symbol;Acc:32480]
ENSG00000004534	3_49886319_G_T_b37	5.79E-06	7.00E-06	rs77941694	RBM6	RNA binding motif protein 6 [Source:HGNC Symbol;Acc:9903]
ENSG00000178252	3_49874246_C_T_b37	1.81E-05	8.00E-06	rs59684465	WDR6	WD repeat domain 6 [Source:HGNC Symbol;Acc:12758]
ENSG00000145020	3_49874246_C_T_b37	5.07E-10	8.00E-06	rs59684465	AMT	aminomethyltransferase [Source:HGNC Symbol;Acc:473]
ENSG00000185614	3_49874246_C_T_b37	4.22E-06	8.00E-06	rs59684465	FAM212A	family with sequence similarity 212, member A [Source:HGNC Symbol;Acc:32480]
ENSG00000178252	3_49866584_T_G_b37	2.10E-05	1.10E-05	rs35129566	WDR6	WD repeat domain 6 [Source:HGNC Symbol;Acc:12758]
ENSG00000145020	3_49866584_T_G_b37	9.25E-10	1.10E-05	rs35129566	AMT	aminomethyltransferase [Source:HGNC Symbol;Acc:473]
ENSG00000185614	3_49866584_T_G_b37	4.20E-06	1.10E-05	rs35129566	FAM212A	family with sequence similarity 212, member A [Source:HGNC Symbol;Acc:32480]
ENSG00000232112	3_49210732_A_C_b37	1.23E-06	1.40E-05	rs7617480	TMA7	translation machinery associated 7 homolog (S. cerevisiae) [Source:HGNC Symbol;Acc:26932]
ENSG00000178252	3_49210732_A_C_b37	8.32E-12	1.40E-05	rs7617480	WDR6	WD repeat domain 6 [Source:HGNC Symbol;Acc:12758]
ENSG00000067560	3_49210732_A_C_b37	2.21E-06	1.40E-05	rs7617480	RHOA	ras homolog family member A [Source:HGNC Symbol;Acc:667]
ENSG00000145020	3_49210732_A_C_b37	1.43E-18	1.40E-05	rs7617480	AMT	aminomethyltransferase [Source:HGNC Symbol;Acc:473]
ENSG00000145020	3_49655927_T_A_b37	2.16E-09	1.40E-05	rs62262672	AMT	aminomethyltransferase [Source:HGNC Symbol;Acc:473]
ENSG00000235261	3_49210732_A_C_b37	9.28E-09	1.40E-05	rs7617480	NICN1-AS1	NICN1 antisense RNA 1 [Source:HGNC Symbol;Acc:40838]
ENSG00000145029	3_49210732_A_C_b37	2.48E-08	1.40E-05	rs7617480	NICN1	nicolin 1 [Source:HGNC Symbol;Acc:18317]
ENSG00000185614	3_49210732_A_C_b37	6.75E-07	1.40E-05	rs7617480	FAM212A	family with sequence similarity 212, member A [Source:HGNC Symbol;Acc:32480]
ENSG00000185614	3_49655927_T_A_b37	5.23E-06	1.40E-05	rs62262672	FAM212A	family with sequence similarity 212, member A [Source:HGNC Symbol;Acc:32480]
ENSG00000145020	3_49715354_G_T_b37	1.24E-09	1.50E-05	rs4855882	AMT	aminomethyltransferase [Source:HGNC Symbol;Acc:473]
ENSG00000185614	3_49715354_G_T_b37	2.42E-06	1.50E-05	rs4855882	FAM212A	family with sequence similarity 212, member A [Source:HGNC Symbol;Acc:32480]
ENSG00000004534	3_49715354_G_T_b37	8.76E-05	1.50E-05	rs4855882	RBM6	RNA binding motif protein 6 [Source:HGNC Symbol;Acc:9903]
ENSG00000145020	3_49656530_CTGAA_C_b37	1.32E-09	2.00E-05	rs72410852	AMT	aminomethyltransferase [Source:HGNC Symbol;Acc:473]
ENSG00000185614	3_49656530_CTGAA_C_b37	2.92E-06	2.00E-05	rs72410852	FAM212A	family with sequence similarity 212, member A [Source:HGNC Symbol;Acc:32480]
ENSG00000004534	3_49656530_CTGAA_C_b37	9.11E-05	2.00E-05	rs72410852	RBM6	RNA binding motif protein 6 [Source:HGNC Symbol;Acc:9903]
ENSG00000145020	3_49808274_T_G_b37	1.44E-09	2.20E-05	rs62262730	AMT	aminomethyltransferase [Source:HGNC Symbol;Acc:473]
ENSG00000185614	3_49808274_T_G_b37	2.62E-06	2.20E-05	rs62262730	FAM212A	family with sequence similarity 212, member A [Source:HGNC Symbol;Acc:32480]
ENSG00000004534	3_49808274_T_G_b37	8.64E-05	2.20E-05	rs62262730	RBM6	RNA binding motif protein 6 [Source:HGNC Symbol;Acc:9903]
ENSG00000178977	17_8183681_A_C_b37	7.76E-05	2.60E-05	rs11078739	LINC00324	long intergenic non-protein coding RNA 324 [Source:HGNC Symbol;Acc:26628]
ENSG00000178252	3_49829653_A_G_b37	3.18E-05	3.00E-05	rs7637711	WDR6	WD repeat domain 6 [Source:HGNC Symbol;Acc:12758]
ENSG00000145020	3_49811047_G_A_b37	1.74E-09	3.00E-05	rs148383796	AMT	aminomethyltransferase [Source:HGNC Symbol;Acc:473]
ENSG00000145020	3_49829653_A_G_b37	2.09E-10	3.00E-05	rs7637711	AMT	aminomethyltransferase [Source:HGNC Symbol;Acc:473]
ENSG00000235261	3_49829653_A_G_b37	3.08E-05	3.00E-05	rs7637711	NICN1-AS1	NICN1 antisense RNA 1 [Source:HGNC Symbol;Acc:40838]
ENSG00000145029	3_49829653_A_G_b37	6.63E-05	3.00E-05	rs7637711	NICN1	nicolin 1 [Source:HGNC Symbol;Acc:18317]
ENSG00000185614	3_49811047_G_A_b37	5.36E-06	3.00E-05	rs148383796	FAM212A	family with sequence similarity 212, member A [Source:HGNC Symbol;Acc:32480]
ENSG00000185614	3_49829653_A_G_b37	2.00E-06	3.00E-05	rs7637711	FAM212A	family with sequence similarity 212, member A [Source:HGNC Symbol;Acc:32480]
ENSG00000178252	3_49887277_T_TC_b37	7.18E-05	3.30E-05	rs200837337	WDR6	WD repeat domain 6 [Source:HGNC Symbol;Acc:12758]
ENSG00000145020	3_49884852_AAAGAG_A_b37	3.72E-07	3.30E-05	rs566869627	AMT	aminomethyltransferase [Source:HGNC Symbol;Acc:473]
ENSG00000145020	3_49887277_T_TC_b37	1.71E-07	3.30E-05	rs200837337	AMT	aminomethyltransferase [Source:HGNC Symbol;Acc:473]
ENSG00000185614	3_49884852_AAAGAG_A_b37	2.01E-05	3.30E-05	rs566869627	FAM212A	family with sequence similarity 212, member A [Source:HGNC Symbol;Acc:32480]
ENSG00000185614	3_49887277_T_TC_b37	7.99E-06	3.30E-05	rs200837337	FAM212A	family with sequence similarity 212, member A [Source:HGNC Symbol;Acc:32480]
ENSG00000004534	3_49887277_T_TC_b37	3.33E-05	3.30E-05	rs200837337	RBM6	RNA binding motif protein 6 [Source:HGNC Symbol;Acc:9903]
ENSG00000178252	3_49835450_C_T_b37	3.15E-05	3.80E-05	rs6790568	WDR6	WD repeat domain 6 [Source:HGNC Symbol;Acc:12758]
ENSG00000145020	3_49789607_T_TA_b37	5.42E-10	3.80E-05	rs201319899	AMT	aminomethyltransferase [Source:HGNC Symbol;Acc:473]
ENSG00000145020	3_49835450_C_T_b37	2.12E-10	3.80E-05	rs6790568	AMT	aminomethyltransferase [Source:HGNC Symbol;Acc:473]
ENSG00000235261	3_49835450_C_T_b37	3.07E-05	3.80E-05	rs6790568	NICN1-AS1	NICN1 antisense RNA 1 [Source:HGNC Symbol;Acc:40838]
ENSG00000145029	3_49835450_C_T_b37	6.83E-05	3.80E-05	rs6790568	NICN1	nicolin 1 [Source:HGNC Symbol;Acc:18317]
ENSG00000185614	3_49789607_T_TA_b37	3.73E-05	3.80E-05	rs201319899	FAM212A	family with sequence similarity 212, member A [Source:HGNC Symbol;Acc:32480]
ENSG00000185614	3_49835450_C_T_b37	2.01E-06	3.80E-05	rs6790568	FAM212A	family with sequence similarity 212, member A [Source:HGNC Symbol;Acc:32480]
ENSG00000145020	3_49777660_T_C_b37	2.63E-09	3.90E-05	rs77473999	AMT	aminomethyltransferase [Source:HGNC Symbol;Acc:473]
ENSG00000185614	3_49777660_T_C_b37	5.59E-06	3.90E-05	rs77473999	FAM212A	family with sequence similarity 212, member A [Source:HGNC Symbol;Acc:32480]
ENSG00000178252	3_49834571_A_G_b37	1.67E-05	4.40E-05	rs6809879	WDR6	WD repeat domain 6 [Source:HGNC Symbol;Acc:12758]
ENSG00000145020	3_49834571_A_G_b37	1.99E-10	4.40E-05	rs6809879	AMT	aminomethyltransferase [Source:HGNC Symbol;Acc:473]
ENSG00000235261	3_49834571_A_G_b37	4.16E-05	4.40E-05	rs6809879	NICN1-AS1	NICN1 antisense RNA 1 [Source:HGNC Symbol;Acc:40838]
ENSG00000185614	3_49834571_A_G_b37	8.28E-06	4.40E-05	rs6809879	FAM212A	family with sequence similarity 212, member A [Source:HGNC Symbol;Acc:32480]
ENSG00000145020	3_49827605_CCT_C_b37	3.01E-09	4.50E-05	rs199640415	AMT	aminomethyltransferase [Source:HGNC Symbol;Acc:473]
ENSG00000185614	3_49827605_CCT_C_b37	1.14E-05	4.50E-05	rs199640415	FAM212A	family with sequence similarity 212, member A [Source:HGNC Symbol;Acc:32480]
ENSG00000213672	3_49476263_T_C_b37	1.30E-05	6.00E-05	rs7619789	NCKIPSD	NCK interacting protein with SH3 domain [Source:HGNC Symbol;Acc:15486]
ENSG00000178252	3_49476263_T_C_b37	5.78E-09	6.00E-05	rs7619789	WDR6	WD repeat domain 6 [Source:HGNC Symbol;Acc:12758]
ENSG00000067560	3_49476263_T_C_b37	4.66E-09	6.00E-05	rs7619789	RHOA	ras homolog family member A [Source:HGNC Symbol;Acc:667]
ENSG00000145020	3_49476263_T_C_b37	3.45E-27	6.00E-05	rs7619789	AMT	aminomethyltransferase [Source:HGNC Symbol;Acc:473]
ENSG00000235261	3_49476263_T_C_b37	4.60E-09	6.00E-05	rs7619789	NICN1-AS1	NICN1 antisense RNA 1 [Source:HGNC Symbol;Acc:40838]
ENSG00000145029	3_49476263_T_C_b37	2.13E-09	6.00E-05	rs7619789	NICN1	nicolin 1 [Source:HGNC Symbol;Acc:18317]
ENSG00000185614	3_49476263_T_C_b37	5.78E-05	6.00E-05	rs7619789	FAM212A	family with sequence similarity 212, member A [Source:HGNC Symbol;Acc:32480]
ENSG00000232112	3_49284513_A_G_b37	8.54E-05	6.50E-05	rs12637346	TMA7	translation machinery associated 7 homolog (S. cerevisiae) [Source:HGNC Symbol;Acc:26932]
ENSG00000213672	3_49284513_A_G_b37	2.51E-05	6.50E-05	rs12637346	NCKIPSD	NCK interacting protein with SH3 domain [Source:HGNC Symbol;Acc:15486]
ENSG00000178252	3_49284513_A_G_b37	3.67E-09	6.50E-05	rs12637346	WDR6	WD repeat domain 6 [Source:HGNC Symbol;Acc:12758]
ENSG00000067560	3_49284513_A_G_b37	2.50E-08	6.50E-05	rs12637346	RHOA	ras homolog family member A [Source:HGNC Symbol;Acc:667]
ENSG00000145020	3_49284513_A_G_b37	1.76E-25	6.50E-05	rs12637346	AMT	aminomethyltransferase [Source:HGNC Symbol;Acc:473]
ENSG00000235261	3_49284513_A_G_b37	1.14E-10	6.50E-05	rs12637346	NICN1-AS1	NICN1 antisense RNA 1 [Source:HGNC Symbol;Acc:40838]
ENSG00000145029	3_49284513_A_G_b37	1.82E-10	6.50E-05	rs12637346	NICN1	nicolin 1 [Source:HGNC Symbol;Acc:18317]
ENSG00000213672	3_49582994_T_C_b37	2.15E-05	7.00E-05	rs3866330	NCKIPSD	NCK interacting protein with SH3 domain [Source:HGNC Symbol;Acc:15486]
ENSG00000213672	3_49604904_G_A_b37	2.59E-05	7.00E-05	rs11928090	NCKIPSD	NCK interacting protein with SH3 domain [Source:HGNC Symbol;Acc:15486]
ENSG00000178252	3_49582994_T_C_b37	3.37E-09	7.00E-05	rs3866330	WDR6	WD repeat domain 6 [Source:HGNC Symbol;Acc:12758]
ENSG00000178252	3_49604904_G_A_b37	3.99E-09	7.00E-05	rs11928090	WDR6	WD repeat domain 6 [Source:HGNC Symbol;Acc:12758]
ENSG00000178252	3_49804290_C_T_b37	3.41E-05	7.00E-05	rs6804970	WDR6	WD repeat domain 6 [Source:HGNC Symbol;Acc:12758]
ENSG00000067560	3_49582994_T_C_b37	2.81E-09	7.00E-05	rs3866330	RHOA	ras homolog family member A [Source:HGNC Symbol;Acc:667]
ENSG00000067560	3_49604904_G_A_b37	3.18E-09	7.00E-05	rs11928090	RHOA	ras homolog family member A [Source:HGNC Symbol;Acc:667]
ENSG00000145020	3_49582994_T_C_b37	4.28E-27	7.00E-05	rs3866330	AMT	aminomethyltransferase [Source:HGNC Symbol;Acc:473]
ENSG00000145020	3_49604904_G_A_b37	7.05E-27	7.00E-05	rs11928090	AMT	aminomethyltransferase [Source:HGNC Symbol;Acc:473]
ENSG00000145020	3_49646263_G_A_b37	1.11E-07	7.00E-05	rs7646288	AMT	aminomethyltransferase [Source:HGNC Symbol;Acc:473]
ENSG00000145020	3_49804290_C_T_b37	4.47E-10	7.00E-05	rs6804970	AMT	aminomethyltransferase [Source:HGNC Symbol;Acc:473]
ENSG00000235261	3_49582994_T_C_b37	5.98E-09	7.00E-05	rs3866330	NICN1-AS1	NICN1 antisense RNA 1 [Source:HGNC Symbol;Acc:40838]
ENSG00000235261	3_49604904_G_A_b37	9.33E-09	7.00E-05	rs11928090	NICN1-AS1	NICN1 antisense RNA 1 [Source:HGNC Symbol;Acc:40838]
ENSG00000235261	3_49804290_C_T_b37	2.84E-05	7.00E-05	rs6804970	NICN1-AS1	NICN1 antisense RNA 1 [Source:HGNC Symbol;Acc:40838]
ENSG00000145029	3_49582994_T_C_b37	2.23E-09	7.00E-05	rs3866330	NICN1	nicolin 1 [Source:HGNC Symbol;Acc:18317]
ENSG00000145029	3_49604904_G_A_b37	3.60E-09	7.00E-05	rs11928090	NICN1	nicolin 1 [Source:HGNC Symbol;Acc:18317]
ENSG00000145029	3_49804290_C_T_b37	7.71E-05	7.00E-05	rs6804970	NICN1	nicolin 1 [Source:HGNC Symbol;Acc:18317]
ENSG00000185614	3_49582994_T_C_b37	5.85E-05	7.00E-05	rs3866330	FAM212A	family with sequence similarity 212, member A [Source:HGNC Symbol;Acc:32480]

ENSG00000185614	3_49604904_G_A_b37	5.99E-05	7.00E-05	rs11928090	FAM212A	family with sequence similarity 212, member A [Source:HGNC Symbol;Acc:32480]
ENSG00000185614	3_49804290_C_T_b37	1.20E-06	7.00E-05	rs6804970	FAM212A	family with sequence similarity 212, member A [Source:HGNC Symbol;Acc:32480]
ENSG00000153406	16_4603161_C_A_b37	6.14E-11	7.00E-05	rs859219	NMRAL1	NmrA-like family domain containing 1 [Source:HGNC Symbol;Acc:24987]
ENSG00000089486	16_4603161_C_A_b37	4.38E-10	7.00E-05	rs859219	CDIP1	cell death-inducing p53 target 1 [Source:HGNC Symbol;Acc:13234]
ENSG00000213672	3_49550774_A_G_b37	1.39E-05	8.00E-05	rs3905330	NCKIPSD	NCK interacting protein with SH3 domain [Source:HGNC Symbol;Acc:15486]
ENSG00000178252	3_49550774_A_G_b37	5.54E-09	8.00E-05	rs3905330	WDR6	WD repeat domain 6 [Source:HGNC Symbol;Acc:12758]
ENSG00000178252	3_49798864_G_A_b37	2.35E-05	8.00E-05	rs7621797	WDR6	WD repeat domain 6 [Source:HGNC Symbol;Acc:12758]
ENSG00000067560	3_49550774_A_G_b37	4.69E-09	8.00E-05	rs3905330	RHOA	ras homolog family member A [Source:HGNC Symbol;Acc:667]
ENSG00000145020	3_49550774_A_G_b37	3.79E-27	8.00E-05	rs3905330	AMT	aminomethyltransferase [Source:HGNC Symbol;Acc:473]
ENSG00000145020	3_49789615_T_A_b37	7.68E-08	8.00E-05	rs7429338	AMT	aminomethyltransferase [Source:HGNC Symbol;Acc:473]
ENSG00000145020	3_49798864_G_A_b37	1.90E-11	8.00E-05	rs7621797	AMT	aminomethyltransferase [Source:HGNC Symbol;Acc:473]
ENSG00000235261	3_49550774_A_G_b37	4.95E-09	8.00E-05	rs3905330	NICN1-AS1	NICN1 antisense RNA 1 [Source:HGNC Symbol;Acc:40838]
ENSG00000235261	3_49798864_G_A_b37	1.73E-05	8.00E-05	rs7621797	NICN1-AS1	NICN1 antisense RNA 1 [Source:HGNC Symbol;Acc:40838]
ENSG00000145029	3_49550774_A_G_b37	2.24E-09	8.00E-05	rs3905330	NICN1	nicolin 1 [Source:HGNC Symbol;Acc:18317]
ENSG00000145029	3_49798864_G_A_b37	4.85E-05	8.00E-05	rs7621797	NICN1	nicolin 1 [Source:HGNC Symbol;Acc:18317]
ENSG00000185614	3_49550774_A_G_b37	6.02E-05	8.00E-05	rs3905330	FAM212A	family with sequence similarity 212, member A [Source:HGNC Symbol;Acc:32480]
ENSG00000185614	3_49798864_G_A_b37	1.16E-05	8.00E-05	rs7621797	FAM212A	family with sequence similarity 212, member A [Source:HGNC Symbol;Acc:32480]
ENSG00000178977	17_8182267_G_A_b37	6.49E-05	8.00E-05	rs6503096	LINC00324	long intergenic non-protein coding RNA 324 [Source:HGNC Symbol;Acc:26628]
ENSG00000232112	3_49236439_T_G_b37	8.87E-05	9.00E-05	rs6808036	TMA7	translation machinery associated 7 homolog (S. cerevisiae) [Source:HGNC Symbol;Acc:26932]
ENSG00000213672	3_49236439_T_G_b37	2.59E-05	9.00E-05	rs6808036	NCKIPSD	NCK interacting protein with SH3 domain [Source:HGNC Symbol;Acc:15486]
ENSG00000213672	3_49286618_T_G_b37	1.83E-05	9.00E-05	rs4955443	NCKIPSD	NCK interacting protein with SH3 domain [Source:HGNC Symbol;Acc:15486]
ENSG00000213672	3_49443081_T_C_b37	1.32E-05	9.00E-05	rs13096474	NCKIPSD	NCK interacting protein with SH3 domain [Source:HGNC Symbol;Acc:15486]
ENSG00000213672	3_49481438_AGGACT_A_b37	1.60E-05	9.00E-05	rs60691649	NCKIPSD	NCK interacting protein with SH3 domain [Source:HGNC Symbol;Acc:15486]
ENSG00000213672	3_49516350_G_A_b37	1.28E-05	9.00E-05	rs9809237	NCKIPSD	NCK interacting protein with SH3 domain [Source:HGNC Symbol;Acc:15486]
ENSG00000213672	3_49596462_C_T_b37	2.15E-05	9.00E-05	rs7625857	NCKIPSD	NCK interacting protein with SH3 domain [Source:HGNC Symbol;Acc:15486]
ENSG00000178252	3_49236439_T_G_b37	8.06E-10	9.00E-05	rs6808036	WDR6	WD repeat domain 6 [Source:HGNC Symbol;Acc:12758]
ENSG00000178252	3_49286618_T_G_b37	7.00E-10	9.00E-05	rs4955443	WDR6	WD repeat domain 6 [Source:HGNC Symbol;Acc:12758]
ENSG00000178252	3_49443081_T_C_b37	6.06E-09	9.00E-05	rs13096474	WDR6	WD repeat domain 6 [Source:HGNC Symbol;Acc:12758]
ENSG00000178252	3_49481438_AGGACT_A_b37	6.90E-09	9.00E-05	rs60691649	WDR6	WD repeat domain 6 [Source:HGNC Symbol;Acc:12758]
ENSG00000178252	3_49516350_G_A_b37	6.86E-09	9.00E-05	rs9809237	WDR6	WD repeat domain 6 [Source:HGNC Symbol;Acc:12758]
ENSG00000178252	3_49596462_C_T_b37	3.36E-09	9.00E-05	rs7625857	WDR6	WD repeat domain 6 [Source:HGNC Symbol;Acc:12758]
ENSG00000178252	3_49609477_C_G_b37	3.24E-05	9.00E-05	rs1532204	WDR6	WD repeat domain 6 [Source:HGNC Symbol;Acc:12758]
ENSG00000178252	3_49609794_G_T_b37	3.23E-05	9.00E-05	rs6797299	WDR6	WD repeat domain 6 [Source:HGNC Symbol;Acc:12758]
ENSG00000178252	3_49615624_G_T_b37	3.25E-05	9.00E-05	rs11130208	WDR6	WD repeat domain 6 [Source:HGNC Symbol;Acc:12758]
ENSG00000067560	3_49236439_T_G_b37	3.39E-08	9.00E-05	rs6808036	RHOA	ras homolog family member A [Source:HGNC Symbol;Acc:667]
ENSG00000067560	3_49286618_T_G_b37	3.80E-08	9.00E-05	rs4955443	RHOA	ras homolog family member A [Source:HGNC Symbol;Acc:667]
ENSG00000067560	3_49443081_T_C_b37	4.63E-09	9.00E-05	rs13096474	RHOA	ras homolog family member A [Source:HGNC Symbol;Acc:667]
ENSG00000067560	3_49481438_AGGACT_A_b37	5.32E-09	9.00E-05	rs60691649	RHOA	ras homolog family member A [Source:HGNC Symbol;Acc:667]
ENSG00000067560	3_49516350_G_A_b37	4.03E-09	9.00E-05	rs9809237	RHOA	ras homolog family member A [Source:HGNC Symbol;Acc:667]
ENSG00000067560	3_49596462_C_T_b37	2.81E-09	9.00E-05	rs7625857	RHOA	ras homolog family member A [Source:HGNC Symbol;Acc:667]
ENSG00000145020	3_49236439_T_G_b37	4.15E-26	9.00E-05	rs6808036	AMT	aminomethyltransferase [Source:HGNC Symbol;Acc:473]
ENSG00000145020	3_49286618_T_G_b37	2.51E-26	9.00E-05	rs4955443	AMT	aminomethyltransferase [Source:HGNC Symbol;Acc:473]
ENSG00000145020	3_49443081_T_C_b37	3.73E-27	9.00E-05	rs13096474	AMT	aminomethyltransferase [Source:HGNC Symbol;Acc:473]
ENSG00000145020	3_49481438_AGGACT_A_b37	8.07E-27	9.00E-05	rs60691649	AMT	aminomethyltransferase [Source:HGNC Symbol;Acc:473]
ENSG00000145020	3_49516350_G_A_b37	2.51E-27	9.00E-05	rs9809237	AMT	aminomethyltransferase [Source:HGNC Symbol;Acc:473]
ENSG00000145020	3_49596462_C_T_b37	4.28E-27	9.00E-05	rs7625857	AMT	aminomethyltransferase [Source:HGNC Symbol;Acc:473]
ENSG00000145020	3_49609477_C_G_b37	2.06E-14	9.00E-05	rs1532204	AMT	aminomethyltransferase [Source:HGNC Symbol;Acc:473]
ENSG00000145020	3_49609794_G_T_b37	2.07E-14	9.00E-05	rs6797299	AMT	aminomethyltransferase [Source:HGNC Symbol;Acc:473]
ENSG00000145020	3_49615624_G_T_b37	2.10E-14	9.00E-05	rs11130208	AMT	aminomethyltransferase [Source:HGNC Symbol;Acc:473]
ENSG00000235261	3_49236439_T_G_b37	1.06E-10	9.00E-05	rs6808036	NICN1-AS1	NICN1 antisense RNA 1 [Source:HGNC Symbol;Acc:40838]
ENSG00000235261	3_49286618_T_G_b37	1.30E-10	9.00E-05	rs4955443	NICN1-AS1	NICN1 antisense RNA 1 [Source:HGNC Symbol;Acc:40838]
ENSG00000235261	3_49443081_T_C_b37	4.57E-09	9.00E-05	rs13096474	NICN1-AS1	NICN1 antisense RNA 1 [Source:HGNC Symbol;Acc:40838]
ENSG00000235261	3_49481438_AGGACT_A_b37	7.98E-09	9.00E-05	rs60691649	NICN1-AS1	NICN1 antisense RNA 1 [Source:HGNC Symbol;Acc:40838]
ENSG00000235261	3_49516350_G_A_b37	5.76E-09	9.00E-05	rs9809237	NICN1-AS1	NICN1 antisense RNA 1 [Source:HGNC Symbol;Acc:40838]
ENSG00000235261	3_49596462_C_T_b37	5.98E-09	9.00E-05	rs7625857	NICN1-AS1	NICN1 antisense RNA 1 [Source:HGNC Symbol;Acc:40838]
ENSG00000235261	3_49609477_C_G_b37	4.49E-07	9.00E-05	rs1532204	NICN1-AS1	NICN1 antisense RNA 1 [Source:HGNC Symbol;Acc:40838]
ENSG00000235261	3_49609794_G_T_b37	4.49E-07	9.00E-05	rs6797299	NICN1-AS1	NICN1 antisense RNA 1 [Source:HGNC Symbol;Acc:40838]
ENSG00000235261	3_49615624_G_T_b37	4.52E-07	9.00E-05	rs11130208	NICN1-AS1	NICN1 antisense RNA 1 [Source:HGNC Symbol;Acc:40838]
ENSG00000145029	3_49236439_T_G_b37	8.05E-11	9.00E-05	rs6808036	NICN1	nicolin 1 [Source:HGNC Symbol;Acc:18317]
ENSG00000145029	3_49286618_T_G_b37	8.64E-11	9.00E-05	rs4955443	NICN1	nicolin 1 [Source:HGNC Symbol;Acc:18317]
ENSG00000145029	3_49443081_T_C_b37	2.19E-09	9.00E-05	rs13096474	NICN1	nicolin 1 [Source:HGNC Symbol;Acc:18317]
ENSG00000145029	3_49481438_AGGACT_A_b37	2.24E-09	9.00E-05	rs60691649	NICN1	nicolin 1 [Source:HGNC Symbol;Acc:18317]
ENSG00000145029	3_49516350_G_A_b37	2.30E-09	9.00E-05	rs9809237	NICN1	nicolin 1 [Source:HGNC Symbol;Acc:18317]
ENSG00000145029	3_49596462_C_T_b37	2.23E-09	9.00E-05	rs7625857	NICN1	nicolin 1 [Source:HGNC Symbol;Acc:18317]
ENSG00000145029	3_49609477_C_G_b37	3.63E-06	9.00E-05	rs1532204	NICN1	nicolin 1 [Source:HGNC Symbol;Acc:18317]
ENSG00000145029	3_49609794_G_T_b37	3.63E-06	9.00E-05	rs6797299	NICN1	nicolin 1 [Source:HGNC Symbol;Acc:18317]
ENSG00000145029	3_49615624_G_T_b37	3.67E-06	9.00E-05	rs11130208	NICN1	nicolin 1 [Source:HGNC Symbol;Acc:18317]
ENSG00000185614	3_49236439_T_G_b37	2.42E-05	9.00E-05	rs6808036	FAM212A	family with sequence similarity 212, member A [Source:HGNC Symbol;Acc:32480]
ENSG00000185614	3_49286618_T_G_b37	3.77E-05	9.00E-05	rs4955443	FAM212A	family with sequence similarity 212, member A [Source:HGNC Symbol;Acc:32480]
ENSG00000185614	3_49443081_T_C_b37	5.99E-05	9.00E-05	rs13096474	FAM212A	family with sequence similarity 212, member A [Source:HGNC Symbol;Acc:32480]
ENSG00000185614	3_49481438_AGGACT_A_b37	4.93E-05	9.00E-05	rs60691649	FAM212A	family with sequence similarity 212, member A [Source:HGNC Symbol;Acc:32480]
ENSG00000185614	3_49516350_G_A_b37	5.41E-05	9.00E-05	rs9809237	FAM212A	family with sequence similarity 212, member A [Source:HGNC Symbol;Acc:32480]
ENSG00000185614	3_49596462_C_T_b37	5.85E-05	9.00E-05	rs7625857	FAM212A	family with sequence similarity 212, member A [Source:HGNC Symbol;Acc:32480]
ENSG00000185614	3_49609477_C_G_b37	7.70E-07	9.00E-05	rs1532204	FAM212A	family with sequence similarity 212, member A [Source:HGNC Symbol;Acc:32480]
ENSG00000185614	3_49609794_G_T_b37	7.70E-07	9.00E-05	rs6797299	FAM212A	family with sequence similarity 212, member A [Source:HGNC Symbol;Acc:32480]
ENSG00000185614	3_49615624_G_T_b37	7.74E-07	9.00E-05	rs11130208	FAM212A	family with sequence similarity 212, member A [Source:HGNC Symbol;Acc:32480]
ENSG00000004534	3_50152491_C_T_b37	3.16E-09	9.00E-05	rs62263602	RBM6	RNA binding motif protein 6 [Source:HGNC Symbol;Acc:9903]
ENSG00000004534	3_50224562_G_A_b37	3.04E-07	9.00E-05	rs12637671	RBM6	RNA binding motif protein 6 [Source:HGNC Symbol;Acc:9903]
ENSG00000170322	11_129707902_C_A_b37	2.06E-06	9.00E-05	rs61916118	NFRKB	nuclear factor related to kappaB binding protein [Source:HGNC Symbol;Acc:7802]
ENSG00000170322	11_129710515_C_T_b37	3.60E-06	9.00E-05	rs59992343	NFRKB	nuclear factor related to kappaB binding protein [Source:HGNC Symbol;Acc:7802]
ENSG00000178977	17_8169006_T_C_b37	4.90E-05	9.00E-05	rs11655563	LINC00324	long intergenic non-protein coding RNA 324 [Source:HGNC Symbol;Acc:26628]
ENSG00000213672	3_49289878_T_G_b37	1.79E-05	1.00E-04	rs4384984	NCKIPSD	NCK interacting protein with SH3 domain [Source:HGNC Symbol;Acc:15486]
ENSG00000213672	3_49400482_T_C_b37	1.03E-05	1.00E-04	rs974495	NCKIPSD	NCK interacting protein with SH3 domain [Source:HGNC Symbol;Acc:15486]
ENSG00000213672	3_49584745_A_G_b37	2.15E-05	1.00E-04	rs3870341	NCKIPSD	NCK interacting protein with SH3 domain [Source:HGNC Symbol;Acc:15486]
ENSG00000178252	3_49289878_T_G_b37	6.83E-10	1.00E-04	rs4384984	WDR6	WD repeat domain 6 [Source:HGNC Symbol;Acc:12758]
ENSG00000178252	3_49400482_T_C_b37	2.97E-09	1.00E-04	rs974495	WDR6	WD repeat domain 6 [Source:HGNC Symbol;Acc:12758]
ENSG00000178252	3_49584745_A_G_b37	3.37E-09	1.00E-04	rs3870341	WDR6	WD repeat domain 6 [Source:HGNC Symbol;Acc:12758]
ENSG00000067560	3_49289878_T_G_b37	3.90E-08	1.00E-04	rs4384984	RHOA	ras homolog family member A [Source:HGNC Symbol;Acc:667]
ENSG00000067560	3_49400482_T_C_b37	2.49E-09	1.00E-04	rs974495	RHOA	ras homolog family member A [Source:HGNC Symbol;Acc:667]
ENSG00000067560	3_49584745_A_G_b37	2.81E-09	1.00E-04	rs3870341	RHOA	ras homolog family member A [Source:HGNC Symbol;Acc:667]
ENSG00000145020	3_49289878_T_G_b37	2.47E-26	1.00E-04	rs4384984	AMT	aminomethyltransferase [Source:HGNC Symbol;Acc:473]
ENSG00000145020	3_49400482_T_C_b37	8.88E-27	1.00E-04	rs974495	AMT	aminomethyltransferase [Source:HGNC Symbol;Acc:473]
ENSG00000145020	3_49584745_A_G_b37	4.28E-27	1.00E-04	rs3870341	AMT	aminomethyltransferase [Source:HGNC Symbol;Acc:473]
ENSG00000145020	3_49624095_T_G_b37	1.56E-13	1.00E-04	rs9869256	AMT	aminomethyltransferase [Source:HGNC Symbol;Acc:473]
ENSG00000145020	3_49632628_T_C_b37	2.48E-11	1.00E-04	rs2329022	AMT	aminomethyltransferase [Source:HGNC Symbol;Acc:473]
ENSG00000145020	3_49917686_G_A_b37	1.35E-05	1.00E-04	rs62262061	AMT	aminomethyltransferase [Source:HGNC Symbol;Acc:473]
ENSG00000235261	3_49289878_T_G_b37	1.33E-10	1.00E-04	rs4384984	NICN1-AS1	NICN1 antisense RNA 1 [Source:HGNC Symbol;Acc:40838]
ENSG00000235261	3_49400482_T_C_b37	2.54E-09	1.00E-04	rs974495	NICN1-AS1	NICN1 antisense RNA 1 [Source:HGNC Symbol;Acc:40838]
ENSG00000235261	3_49584745_A_G_b37	5.98E-09	1.00E-04	rs3870341	NICN1-AS1	NICN1 antisense RNA 1 [Source:HGNC Symbol;Acc:40838]
ENSG00000235261	3_49624095_T_G_b37	1.78E-05	1.00E-04	rs9869256	NICN1-AS1	NICN1 antisense RNA 1 [Source:HGNC Symbol;Acc:40838]
ENSG00000145029	3_49289878_T_G_b37	8.79E-11	1.00E-04	rs4384984	NICN1	nicolin 1 [Source:HGNC Symbol;Acc:18317]
ENSG00000145029	3_49400482_T_C_b37	1.14E-09	1.00E-04	rs974495	NICN1	nicolin 1 [Source:HGNC Symbol;Acc:18317]
ENSG00000145029	3_49584745_A_G_b37	2.23E-09	1.00E-04	rs3870341	NICN1	nicolin 1 [Source:HGNC Symbol;Acc:18317]
ENSG00000145029	3_49624095_T_G_b37	3.83E-05	1.00E-04	rs9869256	NICN1	nicolin 1 [Source:HGNC Symbol;Acc:18317]
ENSG00000145029	3_49632628_T_C_b37	2.67E-05	1.00E-04	rs2329022	NICN1	nicolin 1 [Source:HGNC Symbol;Acc:18317]
ENSG00000185614	3_49289878_T_G_b37	3.92E-05	1.00E-04	rs4384984	FAM212A	family with sequence similarity 212, member A [Source:HGNC Symbol;Acc:32480]
ENSG00000185614	3_49400482_T_C_b37	3.36E-05	1.00E-04	rs974495	FAM212A	family with sequence similarity 212, member A [Source:HGNC Symbol;Acc:32480]
ENSG00000185614	3_49584745_A_G_b37	5.85E-05	1.00E-04	rs3870341	FAM212A	family with sequence similarity 212, member A [Source:HGNC Symbol;Acc:32480]
ENSG00000185614	3_49624095_T_G_b37	3.01E-06	1.00E-04	rs9869256	FAM212A	family with sequence similarity 212, member A [Source:HGNC Symbol;Acc:32480]
ENSG00000004534	3_49917686_G_A_b37	1.93E-07	1.00E-04	rs62262061	RBM6	RNA binding motif protein 6 [Source:HGNC Symbol;Acc:9903]
ENSG00000273035	2_39689282_AT_A_b37	4.93E-05	1.00E-04	rs200333711	RP11-449G16.1	
ENSG00000178977	17_8190253_C_T_b37	7.16E-05	1.00E-04	rs6503097	LINC00324	long intergenic non-protein coding RNA 324 [Source:HGNC Symbol;Acc:26628]
ENSG00000170322	11_129708766_C_G_b37	1.42E-06	0.000101	rs11221871	NFRKB	nuclear factor related to kappaB binding protein [Source:HGNC Symbol;Acc:7802]

ENSG00000170322	11_129686195_G_A_b37	3.88E-06	0.000109	rs11221861	NFRKB	nuclear factor related to kappaB binding protein [Source:HGNC Symbol;Acc:7802]
ENSG00000232112	3_49276077_C_T_b37	7.58E-05	0.000109999	rs10154992	TMA7	translation machinery associated 7 homolog (S. cerevisiae) [Source:HGNC Symbol;Acc:26932]
ENSG00000213672	3_49276077_C_T_b37	2.77E-05	0.000109999	rs10154992	NCKIPSD	NCK interacting protein with SH3 domain [Source:HGNC Symbol;Acc:15486]
ENSG00000213672	3_49442020_A_G_b37	1.48E-05	0.000109999	rs11715060	NCKIPSD	NCK interacting protein with SH3 domain [Source:HGNC Symbol;Acc:15486]
ENSG00000178252	3_49276077_C_T_b37	1.21E-09	0.000109999	rs10154992	WDR6	WD repeat domain 6 [Source:HGNC Symbol;Acc:12758]
ENSG00000178252	3_49442020_A_G_b37	7.17E-09	0.000109999	rs11715060	WDR6	WD repeat domain 6 [Source:HGNC Symbol;Acc:12758]
ENSG00000178252	3_49753788_A_G_b37	3.36E-05	0.000109999	rs11720705	WDR6	WD repeat domain 6 [Source:HGNC Symbol;Acc:12758]
ENSG00000114316	3_49753788_A_G_b37	7.17E-05	0.000109999	rs11720705	USP4	ubiquitin specific peptidase 4 (proto-oncogene) [Source:HGNC Symbol;Acc:12627]
ENSG00000067560	3_49276077_C_T_b37	2.65E-08	0.000109999	rs10154992	RHOA	ras homolog family member A [Source:HGNC Symbol;Acc:667]
ENSG00000067560	3_49442020_A_G_b37	4.69E-09	0.000109999	rs11715060	RHOA	ras homolog family member A [Source:HGNC Symbol;Acc:667]
ENSG00000145020	3_49276077_C_T_b37	5.57E-26	0.000109999	rs10154992	AMT	aminomethyltransferase [Source:HGNC Symbol;Acc:473]
ENSG00000145020	3_49442020_A_G_b37	5.21E-27	0.000109999	rs11715060	AMT	aminomethyltransferase [Source:HGNC Symbol;Acc:473]
ENSG00000145020	3_49753788_A_G_b37	1.40E-10	0.000109999	rs11720705	AMT	aminomethyltransferase [Source:HGNC Symbol;Acc:473]
ENSG00000235261	3_49276077_C_T_b37	1.13E-10	0.000109999	rs10154992	NICN1-AS1	NICN1 antisense RNA 1 [Source:HGNC Symbol;Acc:40838]
ENSG00000235261	3_49442020_A_G_b37	5.00E-09	0.000109999	rs11715060	NICN1-AS1	NICN1 antisense RNA 1 [Source:HGNC Symbol;Acc:40838]
ENSG00000235261	3_49753788_A_G_b37	3.52E-05	0.000109999	rs11720705	NICN1-AS1	NICN1 antisense RNA 1 [Source:HGNC Symbol;Acc:40838]
ENSG00000145029	3_49276077_C_T_b37	7.62E-11	0.000109999	rs10154992	NICN1	nicolin 1 [Source:HGNC Symbol;Acc:18317]
ENSG00000145029	3_49442020_A_G_b37	2.42E-09	0.000109999	rs11715060	NICN1	nicolin 1 [Source:HGNC Symbol;Acc:18317]
ENSG00000145029	3_49753788_A_G_b37	5.45E-05	0.000109999	rs11720705	NICN1	nicolin 1 [Source:HGNC Symbol;Acc:18317]
ENSG00000185614	3_49276077_C_T_b37	2.45E-05	0.000109999	rs10154992	FAM212A	family with sequence similarity 212, member A [Source:HGNC Symbol;Acc:32480]
ENSG00000185614	3_49442020_A_G_b37	4.40E-05	0.000109999	rs11715060	FAM212A	family with sequence similarity 212, member A [Source:HGNC Symbol;Acc:32480]
ENSG00000185614	3_49753788_A_G_b37	1.42E-06	0.000109999	rs11720705	FAM212A	family with sequence similarity 212, member A [Source:HGNC Symbol;Acc:32480]
ENSG00000004534	3_50044006_T_A_b37	4.90E-10	0.000109999	rs7635601	RBM6	RNA binding motif protein 6 [Source:HGNC Symbol;Acc:9903]
ENSG00000004534	3_50049958_G_T_b37	1.48E-11	0.000109999	rs7645466	RBM6	RNA binding motif protein 6 [Source:HGNC Symbol;Acc:9903]
ENSG00000120256	6_150003180_G_A_b37	1.22E-05	0.000109999	rs147884962	LRP11	low density lipoprotein receptor-related protein 11 [Source:HGNC Symbol;Acc:16936]
ENSG00000147894	9_27585721_G_C_b37	1.33E-06	0.000109999	rs700823	C9orf72	chromosome 9 open reading frame 72 [Source:HGNC Symbol;Acc:28337]
ENSG00000225213	10_16749526_G_T_b37	2.06E-05	0.000109999	rs3951063	RP11-197M22.2	
ENSG00000264247	18_72385570_A_G_b37	1.22E-10	0.000109999	rs7244762	LINC00909	long intergenic non-protein coding RNA 909 [Source:HGNC Symbol;Acc:44331]
ENSG00000232112	3_49275828_A_G_b37	9.72E-05	0.000119999	rs11130186	TMA7	translation machinery associated 7 homolog (S. cerevisiae) [Source:HGNC Symbol;Acc:26932]
ENSG00000213672	3_49275828_A_G_b37	2.86E-05	0.000119999	rs11130186	NCKIPSD	NCK interacting protein with SH3 domain [Source:HGNC Symbol;Acc:15486]
ENSG00000213672	3_49483074_A_G_b37	1.25E-05	0.000119999	rs57273516	NCKIPSD	NCK interacting protein with SH3 domain [Source:HGNC Symbol;Acc:15486]
ENSG00000178252	3_49275828_A_G_b37	1.65E-09	0.000119999	rs11130186	WDR6	WD repeat domain 6 [Source:HGNC Symbol;Acc:12758]
ENSG00000178252	3_49483074_A_G_b37	6.10E-09	0.000119999	rs57273516	WDR6	WD repeat domain 6 [Source:HGNC Symbol;Acc:12758]
ENSG00000067560	3_49275828_A_G_b37	2.29E-08	0.000119999	rs11130186	RHOA	ras homolog family member A [Source:HGNC Symbol;Acc:667]
ENSG00000067560	3_49483074_A_G_b37	4.66E-09	0.000119999	rs57273516	RHOA	ras homolog family member A [Source:HGNC Symbol;Acc:667]
ENSG00000067560	3_49626306_G_A_b37	4.56E-05	0.000119999	rs6446285	RHOA	ras homolog family member A [Source:HGNC Symbol;Acc:667]
ENSG00000145020	3_49275828_A_G_b37	2.70E-25	0.000119999	rs11130186	AMT	aminomethyltransferase [Source:HGNC Symbol;Acc:473]
ENSG00000145020	3_49483074_A_G_b37	3.97E-27	0.000119999	rs57273516	AMT	aminomethyltransferase [Source:HGNC Symbol;Acc:473]
ENSG00000145020	3_49626306_G_A_b37	3.65E-13	0.000119999	rs6446285	AMT	aminomethyltransferase [Source:HGNC Symbol;Acc:473]
ENSG00000145020	3_50101468_G_A_b37	9.61E-05	0.000119999	rs13097720	AMT	aminomethyltransferase [Source:HGNC Symbol;Acc:473]
ENSG00000235261	3_49275828_A_G_b37	2.01E-10	0.000119999	rs11130186	NICN1-AS1	NICN1 antisense RNA 1 [Source:HGNC Symbol;Acc:40838]
ENSG00000235261	3_49483074_A_G_b37	4.82E-09	0.000119999	rs57273516	NICN1-AS1	NICN1 antisense RNA 1 [Source:HGNC Symbol;Acc:40838]
ENSG00000235261	3_49626306_G_A_b37	1.16E-05	0.000119999	rs6446285	NICN1-AS1	NICN1 antisense RNA 1 [Source:HGNC Symbol;Acc:40838]
ENSG00000145029	3_49275828_A_G_b37	8.59E-11	0.000119999	rs11130186	NICN1	nicolin 1 [Source:HGNC Symbol;Acc:18317]
ENSG00000145029	3_49483074_A_G_b37	2.30E-09	0.000119999	rs57273516	NICN1	nicolin 1 [Source:HGNC Symbol;Acc:18317]
ENSG00000145029	3_49626306_G_A_b37	4.45E-05	0.000119999	rs6446285	NICN1	nicolin 1 [Source:HGNC Symbol;Acc:18317]
ENSG00000185614	3_49275828_A_G_b37	1.51E-05	0.000119999	rs11130186	FAM212A	family with sequence similarity 212, member A [Source:HGNC Symbol;Acc:32480]
ENSG00000185614	3_49483074_A_G_b37	5.39E-05	0.000119999	rs57273516	FAM212A	family with sequence similarity 212, member A [Source:HGNC Symbol;Acc:32480]
ENSG00000185614	3_49626306_G_A_b37	2.38E-06	0.000119999	rs6446285	FAM212A	family with sequence similarity 212, member A [Source:HGNC Symbol;Acc:32480]
ENSG00000004534	3_50101468_G_A_b37	3.37E-09	0.000119999	rs13097720	RBM6	RNA binding motif protein 6 [Source:HGNC Symbol;Acc:9903]
ENSG00000225213	10_16756849_T_C_b37	7.60E-05	0.000119999	rs4747283	RP11-197M22.2	
ENSG00000178977	17_8174893_G_A_b37	5.57E-05	0.000119999	rs7225429	LINC00324	long intergenic non-protein coding RNA 324 [Source:HGNC Symbol;Acc:26628]
ENSG00000264247	18_72381941_C_T_b37	2.78E-10	0.000119999	rs10221304	LINC00909	long intergenic non-protein coding RNA 909 [Source:HGNC Symbol;Acc:44331]
ENSG00000232112	3_49255172_T_C_b37	8.27E-05	0.000129999	rs2082923	TMA7	translation machinery associated 7 homolog (S. cerevisiae) [Source:HGNC Symbol;Acc:26932]
ENSG00000213672	3_49255172_T_C_b37	2.56E-05	0.000129999	rs2082923	NCKIPSD	NCK interacting protein with SH3 domain [Source:HGNC Symbol;Acc:15486]
ENSG00000213672	3_49432321_C_T_b37	1.32E-05	0.000129999	rs10155014	NCKIPSD	NCK interacting protein with SH3 domain [Source:HGNC Symbol;Acc:15486]
ENSG00000178252	3_49255172_T_C_b37	1.26E-09	0.000129999	rs2082923	WDR6	WD repeat domain 6 [Source:HGNC Symbol;Acc:12758]
ENSG00000178252	3_49432321_C_T_b37	6.05E-09	0.000129999	rs10155014	WDR6	WD repeat domain 6 [Source:HGNC Symbol;Acc:12758]
ENSG00000178252	3_49739507_G_C_b37	3.83E-05	0.000129999	rs1491985	WDR6	WD repeat domain 6 [Source:HGNC Symbol;Acc:12758]
ENSG00000178252	3_49745235_G_A_b37	3.83E-05	0.000129999	rs11709734	WDR6	WD repeat domain 6 [Source:HGNC Symbol;Acc:12758]
ENSG00000114316	3_49739507_G_C_b37	7.13E-05	0.000129999	rs1491985	USP4	ubiquitin specific peptidase 4 (proto-oncogene) [Source:HGNC Symbol;Acc:12627]
ENSG00000114316	3_49745235_G_A_b37	7.13E-05	0.000129999	rs11709734	USP4	ubiquitin specific peptidase 4 (proto-oncogene) [Source:HGNC Symbol;Acc:12627]
ENSG00000067560	3_49255172_T_C_b37	3.32E-08	0.000129999	rs2082923	RHOA	ras homolog family member A [Source:HGNC Symbol;Acc:667]
ENSG00000067560	3_49432321_C_T_b37	4.63E-09	0.000129999	rs10155014	RHOA	ras homolog family member A [Source:HGNC Symbol;Acc:667]
ENSG00000145020	3_49255172_T_C_b37	4.80E-26	0.000129999	rs2082923	AMT	aminomethyltransferase [Source:HGNC Symbol;Acc:473]
ENSG00000145020	3_49432321_C_T_b37	3.71E-27	0.000129999	rs10155014	AMT	aminomethyltransferase [Source:HGNC Symbol;Acc:473]
ENSG00000145020	3_49739507_G_C_b37	1.53E-10	0.000129999	rs1491985	AMT	aminomethyltransferase [Source:HGNC Symbol;Acc:473]
ENSG00000145020	3_49745235_G_A_b37	1.53E-10	0.000129999	rs11709734	AMT	aminomethyltransferase [Source:HGNC Symbol;Acc:473]
ENSG00000235261	3_49255172_T_C_b37	9.62E-11	0.000129999	rs2082923	NICN1-AS1	NICN1 antisense RNA 1 [Source:HGNC Symbol;Acc:40838]
ENSG00000235261	3_49432321_C_T_b37	4.57E-09	0.000129999	rs10155014	NICN1-AS1	NICN1 antisense RNA 1 [Source:HGNC Symbol;Acc:40838]
ENSG00000235261	3_49739507_G_C_b37	2.02E-05	0.000129999	rs1491985	NICN1-AS1	NICN1 antisense RNA 1 [Source:HGNC Symbol;Acc:40838]
ENSG00000235261	3_49745235_G_A_b37	2.02E-05	0.000129999	rs11709734	NICN1-AS1	NICN1 antisense RNA 1 [Source:HGNC Symbol;Acc:40838]
ENSG00000145029	3_49255172_T_C_b37	6.49E-11	0.000129999	rs2082923	NICN1	nicolin 1 [Source:HGNC Symbol;Acc:18317]
ENSG00000145029	3_49432321_C_T_b37	2.19E-09	0.000129999	rs10155014	NICN1	nicolin 1 [Source:HGNC Symbol;Acc:18317]
ENSG00000145029	3_49739507_G_C_b37	3.72E-05	0.000129999	rs1491985	NICN1	nicolin 1 [Source:HGNC Symbol;Acc:18317]
ENSG00000145029	3_49745235_G_A_b37	3.72E-05	0.000129999	rs11709734	NICN1	nicolin 1 [Source:HGNC Symbol;Acc:18317]
ENSG00000185614	3_49255172_T_C_b37	2.40E-05	0.000129999	rs2082923	FAM212A	family with sequence similarity 212, member A [Source:HGNC Symbol;Acc:32480]
ENSG00000185614	3_49432321_C_T_b37	5.97E-05	0.000129999	rs10155014	FAM212A	family with sequence similarity 212, member A [Source:HGNC Symbol;Acc:32480]
ENSG00000185614	3_49739507_G_C_b37	1.42E-06	0.000129999	rs1491985	FAM212A	family with sequence similarity 212, member A [Source:HGNC Symbol;Acc:32480]
ENSG00000185614	3_49745235_G_A_b37	1.42E-06	0.000129999	rs11709734	FAM212A	family with sequence similarity 212, member A [Source:HGNC Symbol;Acc:32480]
ENSG00000178977	17_8183852_C_G_b37	6.52E-05	0.000129999	rs11652212	LINC00324	long intergenic non-protein coding RNA 324 [Source:HGNC Symbol;Acc:26628]
ENSG00000232112	3_48412264_CG_C_b37	1.52E-08	0.000139999	rs35404483	TMA7	translation machinery associated 7 homolog (S. cerevisiae) [Source:HGNC Symbol;Acc:26932]
ENSG00000244380	3_48412264_CG_C_b37	8.84E-07	0.000139999	rs35404483	RP11-24C3.2	
ENSG00000213672	3_48412264_CG_C_b37	1.88E-05	0.000139999	rs35404483	NCKIPSD	NCK interacting protein with SH3 domain [Source:HGNC Symbol;Acc:15486]
ENSG00000213672	3_49409002_G_C_b37	1.30E-05	0.000139999	rs11719762	NCKIPSD	NCK interacting protein with SH3 domain [Source:HGNC Symbol;Acc:15486]
ENSG00000213672	3_49536863_T_A_b37	1.30E-05	0.000139999	rs3870339	NCKIPSD	NCK interacting protein with SH3 domain [Source:HGNC Symbol;Acc:15486]
ENSG00000178252	3_49409002_G_C_b37	5.85E-09	0.000139999	rs11719762	WDR6	WD repeat domain 6 [Source:HGNC Symbol;Acc:12758]
ENSG00000178252	3_49536863_T_A_b37	5.78E-09	0.000139999	rs3870339	WDR6	WD repeat domain 6 [Source:HGNC Symbol;Acc:12758]
ENSG00000067560	3_49409002_G_C_b37	4.65E-09	0.000139999	rs11719762	RHOA	ras homolog family member A [Source:HGNC Symbol;Acc:667]
ENSG00000067560	3_49536863_T_A_b37	4.66E-09	0.000139999	rs3870339	RHOA	ras homolog family member A [Source:HGNC Symbol;Acc:667]
ENSG00000145020	3_49409002_G_C_b37	3.52E-27	0.000139999	rs11719762	AMT	aminomethyltransferase [Source:HGNC Symbol;Acc:473]
ENSG00000145020	3_49536863_T_A_b37	3.45E-27	0.000139999	rs3870339	AMT	aminomethyltransferase [Source:HGNC Symbol;Acc:473]
ENSG00000235261	3_49409002_G_C_b37	4.59E-09	0.000139999	rs11719762	NICN1-AS1	NICN1 antisense RNA 1 [Source:HGNC Symbol;Acc:40838]
ENSG00000235261	3_49536863_T_A_b37	4.60E-09	0.000139999	rs3870339	NICN1-AS1	NICN1 antisense RNA 1 [Source:HGNC Symbol;Acc:40838]
ENSG00000145029	3_49409002_G_C_b37	2.14E-09	0.000139999	rs11719762	NICN1	nicolin 1 [Source:HGNC Symbol;Acc:18317]
ENSG00000145029	3_49536863_T_A_b37	2.13E-09	0.000139999	rs3870339	NICN1	nicolin 1 [Source:HGNC Symbol;Acc:18317]
ENSG00000185614	3_49409002_G_C_b37	5.83E-05	0.000139999	rs11719762	FAM212A	family with sequence similarity 212, member A [Source:HGNC Symbol;Acc:32480]
ENSG00000185614	3_49536863_T_A_b37	5.78E-05	0.000139999	rs3870339	FAM212A	family with sequence similarity 212, member A [Source:HGNC Symbol;Acc:32480]
ENSG00000232112	3_49218888_G_A_b37	2.87E-05	0.000149999	rs4955421	TMA7	translation machinery associated 7 homolog (S. cerevisiae) [Source:HGNC Symbol;Acc:26932]
ENSG00000232112	3_49222904_T_C_b37	1.91E-05	0.000149999	rs7432077	TMA7	translation machinery associated 7 homolog (S. cerevisiae) [Source:HGNC Symbol;Acc:26932]
ENSG00000213672	3_49218888_G_A_b37	1.60E-05	0.000149999	rs4955421	NCKIPSD	NCK interacting protein with SH3 domain [Source:HGNC Symbol;Acc:15486]
ENSG00000213672	3_49222904_T_C_b37	7.41E-05	0.000149999	rs7432077	NCKIPSD	NCK interacting protein with SH3 domain [Source:HGNC Symbol;Acc:15486]
ENSG00000178252	3_49218888_G_A_b37	7.89E-10	0.000149999	rs4955421	WDR6	WD repeat domain 6 [Source:HGNC Symbol;Acc:12758]
ENSG00000178252	3_49222904_T_C_b37	1.31E-09	0.000149999	rs7432077	WDR6	WD repeat domain 6 [Source:HGNC Symbol;Acc:12758]
ENSG00000067560	3_49218888_G_A_b37	4.35E-08	0.000149999	rs4955421	RHOA	ras homolog family member A [Source:HGNC Symbol;Acc:667]
ENSG00000067560	3_49222904_T_C_b37	6.00E-08	0.000149999	rs7432077	RHOA	ras homolog family member A [Source:HGNC Symbol;Acc:667]
ENSG00000145020	3_49218888_G_A_b37	2.83E-26	0.000149999	rs4955421	AMT	aminomethyltransferase [Source:HGNC Symbol;Acc:473]
ENSG00000145020	3_49222904_T_C_b37	5.97E-26	0.000149999	rs7432077	AMT	aminomethyltransferase [Source:HGNC Symbol;Acc:473]
ENSG00000235261	3_49218888_G_A_b37	1.91E-10	0.000149999	rs4955421	NICN1-AS1	NICN1 antisense RNA 1 [Source:HGNC Symbol;Acc:40838]
ENSG00000235261	3_49222904_T_C_b37	1.67E-10	0.000149999	rs7432077	NICN1-AS1	NICN1 antisense RNA 1 [Source:HGNC Symbol;Acc:40838]
ENSG00000145029	3_49218888_G_A_b37	1.13E-10	0.000149999	rs4955421	NICN1	nicolin 1 [Source:HGNC Symbol;Acc:18317]
ENSG00000145029	3_49222904_T_C_b37	4.46E-11	0.000149999	rs7432077	NICN1	nicolin 1 [Source:HGNC Symbol;Acc:18317]
ENSG00000185614	3_49218888_G_A_b37	1.17E-05	0.000149999	rs4955421	FAM212A	family with sequence similarity 212, member A [Source:HGNC Symbol;Acc:32480]

ENSG00000185614	3_49222904_T_C_b37	9.92E-06	0.000149999	rs7432077	FAM212A	family with sequence similarity 212, member A [Source:HGNC Symbol;Acc:32480]
ENSG00000004534	3_50031840_C_T_b37	4.52E-10	0.000149999	rs35012435	RBM6	RNA binding motif protein 6 [Source:HGNC Symbol;Acc:9903]
ENSG00000273035	2_39647221_T_C_b37	1.73E-05	0.000149999	rs7598583	RP11-449G16.1	
ENSG00000213672	3_49460350_A_G_b37	1.35E-05	0.000159998	rs1464569	NCKIPSD	NCK interacting protein with SH3 domain [Source:HGNC Symbol;Acc:15486]
ENSG00000213672	3_49521549_G_A_b37	1.30E-05	0.000159998	rs1009051	NCKIPSD	NCK interacting protein with SH3 domain [Source:HGNC Symbol;Acc:15486]
ENSG00000178252	3_49460350_A_G_b37	3.72E-09	0.000159998	rs1464569	WDR6	WD repeat domain 6 [Source:HGNC Symbol;Acc:12758]
ENSG00000178252	3_49521549_G_A_b37	5.78E-09	0.000159998	rs1009051	WDR6	WD repeat domain 6 [Source:HGNC Symbol;Acc:12758]
ENSG00000178252	3_49657441_A_G_b37	5.28E-05	0.000159998	rs4855833	WDR6	WD repeat domain 6 [Source:HGNC Symbol;Acc:12758]
ENSG00000178252	3_49769071_C_T_b37	3.83E-05	0.000159998	rs9870858	WDR6	WD repeat domain 6 [Source:HGNC Symbol;Acc:12758]
ENSG00000114316	3_49769071_C_T_b37	7.13E-05	0.000159998	rs9870858	USP4	ubiquitin specific peptidase 4 (proto-oncogene) [Source:HGNC Symbol;Acc:12627]
ENSG00000067560	3_49460350_A_G_b37	1.73E-09	0.000159998	rs1464569	RHOA	ras homolog family member A [Source:HGNC Symbol;Acc:667]
ENSG00000067560	3_49521549_G_A_b37	4.66E-09	0.000159998	rs1009051	RHOA	ras homolog family member A [Source:HGNC Symbol;Acc:667]
ENSG00000145020	3_49460350_A_G_b37	2.13E-26	0.000159998	rs1464569	AMT	aminomethyltransferase [Source:HGNC Symbol;Acc:473]
ENSG00000145020	3_49521549_G_A_b37	3.45E-27	0.000159998	rs1009051	AMT	aminomethyltransferase [Source:HGNC Symbol;Acc:473]
ENSG00000145020	3_49657441_A_G_b37	1.60E-09	0.000159998	rs4855833	AMT	aminomethyltransferase [Source:HGNC Symbol;Acc:473]
ENSG00000145020	3_49769071_C_T_b37	1.53E-10	0.000159998	rs9870858	AMT	aminomethyltransferase [Source:HGNC Symbol;Acc:473]
ENSG00000235261	3_49460350_A_G_b37	3.47E-09	0.000159998	rs1464569	NICN1-AS1	NICN1 antisense RNA 1 [Source:HGNC Symbol;Acc:40838]
ENSG00000235261	3_49521549_G_A_b37	4.60E-09	0.000159998	rs1009051	NICN1-AS1	NICN1 antisense RNA 1 [Source:HGNC Symbol;Acc:40838]
ENSG00000235261	3_49769071_C_T_b37	2.02E-05	0.000159998	rs9870858	NICN1-AS1	NICN1 antisense RNA 1 [Source:HGNC Symbol;Acc:40838]
ENSG00000145029	3_49460350_A_G_b37	3.00E-09	0.000159998	rs1464569	NICN1	nicolin 1 [Source:HGNC Symbol;Acc:18317]
ENSG00000145029	3_49521549_G_A_b37	2.13E-09	0.000159998	rs1009051	NICN1	nicolin 1 [Source:HGNC Symbol;Acc:18317]
ENSG00000145029	3_49769071_C_T_b37	3.72E-05	0.000159998	rs9870858	NICN1	nicolin 1 [Source:HGNC Symbol;Acc:18317]
ENSG00000185614	3_49460350_A_G_b37	5.00E-05	0.000159998	rs1464569	FAM212A	family with sequence similarity 212, member A [Source:HGNC Symbol;Acc:32480]
ENSG00000185614	3_49521549_G_A_b37	5.78E-05	0.000159998	rs1009051	FAM212A	family with sequence similarity 212, member A [Source:HGNC Symbol;Acc:32480]
ENSG00000185614	3_49657441_A_G_b37	9.44E-07	0.000159998	rs4855833	FAM212A	family with sequence similarity 212, member A [Source:HGNC Symbol;Acc:32480]
ENSG00000185614	3_49769071_C_T_b37	1.42E-06	0.000159998	rs9870858	FAM212A	family with sequence similarity 212, member A [Source:HGNC Symbol;Acc:32480]
ENSG00000004534	3_49657441_A_G_b37	4.37E-05	0.000159998	rs4855833	RBM6	RNA binding motif protein 6 [Source:HGNC Symbol;Acc:9903]
ENSG00000004534	3_50222926_T_A_b37	3.31E-07	0.000159998	rs1046956	RBM6	RNA binding motif protein 6 [Source:HGNC Symbol;Acc:9903]
ENSG00000232112	3_49228946_A_G_b37	5.91E-05	0.000169998	rs11130183	TMA7	translation machinery associated 7 homolog (S. cerevisiae) [Source:HGNC Symbol;Acc:26932]
ENSG00000232112	3_49269758_G_T_b37	7.80E-05	0.000169998	rs2099526	TMA7	translation machinery associated 7 homolog (S. cerevisiae) [Source:HGNC Symbol;Acc:26932]
ENSG00000213672	3_49228946_A_G_b37	2.34E-05	0.000169998	rs11130183	NCKIPSD	NCK interacting protein with SH3 domain [Source:HGNC Symbol;Acc:15486]
ENSG00000213672	3_49269758_G_T_b37	3.43E-05	0.000169998	rs2099526	NCKIPSD	NCK interacting protein with SH3 domain [Source:HGNC Symbol;Acc:15486]
ENSG00000213672	3_49412496_A_G_b37	7.92E-06	0.000169998	rs6775069	NCKIPSD	NCK interacting protein with SH3 domain [Source:HGNC Symbol;Acc:15486]
ENSG00000213672	3_49484992_T_C_b37	2.08E-05	0.000169998	rs11923032	NCKIPSD	NCK interacting protein with SH3 domain [Source:HGNC Symbol;Acc:15486]
ENSG00000213672	3_49496263_G_A_b37	1.28E-05	0.000169998	rs2329025	NCKIPSD	NCK interacting protein with SH3 domain [Source:HGNC Symbol;Acc:15486]
ENSG00000213672	3_49518551_A_G_b37	8.02E-06	0.000169998	rs7622786	NCKIPSD	NCK interacting protein with SH3 domain [Source:HGNC Symbol;Acc:15486]
ENSG00000213672	3_49529601_G_T_b37	1.30E-05	0.000169998	rs4855858	NCKIPSD	NCK interacting protein with SH3 domain [Source:HGNC Symbol;Acc:15486]
ENSG00000213672	3_49598064_T_C_b37	1.80E-05	0.000169998	rs9862534	NCKIPSD	NCK interacting protein with SH3 domain [Source:HGNC Symbol;Acc:15486]
ENSG00000178252	3_49228946_A_G_b37	6.96E-10	0.000169998	rs11130183	WDR6	WD repeat domain 6 [Source:HGNC Symbol;Acc:12758]
ENSG00000178252	3_49269758_G_T_b37	9.66E-10	0.000169998	rs2099526	WDR6	WD repeat domain 6 [Source:HGNC Symbol;Acc:12758]
ENSG00000178252	3_49412496_A_G_b37	2.93E-09	0.000169998	rs6775069	WDR6	WD repeat domain 6 [Source:HGNC Symbol;Acc:12758]
ENSG00000178252	3_49484992_T_C_b37	1.35E-08	0.000169998	rs11923032	WDR6	WD repeat domain 6 [Source:HGNC Symbol;Acc:12758]
ENSG00000178252	3_49496263_G_A_b37	5.85E-09	0.000169998	rs2329025	WDR6	WD repeat domain 6 [Source:HGNC Symbol;Acc:12758]
ENSG00000178252	3_49518551_A_G_b37	7.64E-09	0.000169998	rs7622786	WDR6	WD repeat domain 6 [Source:HGNC Symbol;Acc:12758]
ENSG00000178252	3_49529601_G_T_b37	5.78E-09	0.000169998	rs4855858	WDR6	WD repeat domain 6 [Source:HGNC Symbol;Acc:12758]
ENSG00000178252	3_49598064_T_C_b37	1.89E-09	0.000169998	rs9862534	WDR6	WD repeat domain 6 [Source:HGNC Symbol;Acc:12758]
ENSG00000178252	3_49642027_C_T_b37	4.87E-05	0.000169998	rs4241405	WDR6	WD repeat domain 6 [Source:HGNC Symbol;Acc:12758]
ENSG00000067560	3_49228946_A_G_b37	3.41E-08	0.000169998	rs11130183	RHOA	ras homolog family member A [Source:HGNC Symbol;Acc:667]
ENSG00000067560	3_49269758_G_T_b37	2.43E-08	0.000169998	rs2099526	RHOA	ras homolog family member A [Source:HGNC Symbol;Acc:667]
ENSG00000067560	3_49412496_A_G_b37	1.22E-08	0.000169998	rs6775069	RHOA	ras homolog family member A [Source:HGNC Symbol;Acc:667]
ENSG00000067560	3_49484992_T_C_b37	7.95E-09	0.000169998	rs11923032	RHOA	ras homolog family member A [Source:HGNC Symbol;Acc:667]
ENSG00000067560	3_49496263_G_A_b37	4.68E-09	0.000169998	rs2329025	RHOA	ras homolog family member A [Source:HGNC Symbol;Acc:667]
ENSG00000067560	3_49518551_A_G_b37	1.22E-08	0.000169998	rs7622786	RHOA	ras homolog family member A [Source:HGNC Symbol;Acc:667]
ENSG00000067560	3_49529601_G_T_b37	4.66E-09	0.000169998	rs4855858	RHOA	ras homolog family member A [Source:HGNC Symbol;Acc:667]
ENSG00000067560	3_49598064_T_C_b37	3.61E-09	0.000169998	rs9862534	RHOA	ras homolog family member A [Source:HGNC Symbol;Acc:667]
ENSG00000145020	3_49228946_A_G_b37	3.47E-26	0.000169998	rs11130183	AMT	aminomethyltransferase [Source:HGNC Symbol;Acc:473]
ENSG00000145020	3_49269758_G_T_b37	3.26E-26	0.000169998	rs2099526	AMT	aminomethyltransferase [Source:HGNC Symbol;Acc:473]
ENSG00000145020	3_49412496_A_G_b37	5.30E-27	0.000169998	rs6775069	AMT	aminomethyltransferase [Source:HGNC Symbol;Acc:473]
ENSG00000145020	3_49484992_T_C_b37	2.57E-27	0.000169998	rs11923032	AMT	aminomethyltransferase [Source:HGNC Symbol;Acc:473]
ENSG00000145020	3_49496263_G_A_b37	3.38E-27	0.000169998	rs2329025	AMT	aminomethyltransferase [Source:HGNC Symbol;Acc:473]
ENSG00000145020	3_49518551_A_G_b37	8.84E-25	0.000169998	rs7622786	AMT	aminomethyltransferase [Source:HGNC Symbol;Acc:473]
ENSG00000145020	3_49529601_G_T_b37	3.45E-27	0.000169998	rs4855858	AMT	aminomethyltransferase [Source:HGNC Symbol;Acc:473]
ENSG00000145020	3_49598064_T_C_b37	2.37E-27	0.000169998	rs9862534	AMT	aminomethyltransferase [Source:HGNC Symbol;Acc:473]
ENSG00000145020	3_49642027_C_T_b37	6.67E-14	0.000169998	rs4241405	AMT	aminomethyltransferase [Source:HGNC Symbol;Acc:473]
ENSG00000235261	3_49228946_A_G_b37	1.54E-10	0.000169998	rs11130183	NICN1-AS1	NICN1 antisense RNA 1 [Source:HGNC Symbol;Acc:40838]
ENSG00000235261	3_49269758_G_T_b37	1.75E-10	0.000169998	rs2099526	NICN1-AS1	NICN1 antisense RNA 1 [Source:HGNC Symbol;Acc:40838]
ENSG00000235261	3_49412496_A_G_b37	6.34E-09	0.000169998	rs6775069	NICN1-AS1	NICN1 antisense RNA 1 [Source:HGNC Symbol;Acc:40838]
ENSG00000235261	3_49484992_T_C_b37	3.72E-09	0.000169998	rs11923032	NICN1-AS1	NICN1 antisense RNA 1 [Source:HGNC Symbol;Acc:40838]
ENSG00000235261	3_49496263_G_A_b37	4.57E-09	0.000169998	rs2329025	NICN1-AS1	NICN1 antisense RNA 1 [Source:HGNC Symbol;Acc:40838]
ENSG00000235261	3_49518551_A_G_b37	2.24E-08	0.000169998	rs7622786	NICN1-AS1	NICN1 antisense RNA 1 [Source:HGNC Symbol;Acc:40838]
ENSG00000235261	3_49529601_G_T_b37	4.60E-09	0.000169998	rs4855858	NICN1-AS1	NICN1 antisense RNA 1 [Source:HGNC Symbol;Acc:40838]
ENSG00000235261	3_49598064_T_C_b37	7.44E-09	0.000169998	rs9862534	NICN1-AS1	NICN1 antisense RNA 1 [Source:HGNC Symbol;Acc:40838]
ENSG00000235261	3_49642027_C_T_b37	1.29E-06	0.000169998	rs4241405	NICN1-AS1	NICN1 antisense RNA 1 [Source:HGNC Symbol;Acc:40838]
ENSG00000145029	3_49228946_A_G_b37	5.98E-11	0.000169998	rs11130183	NICN1	nicolin 1 [Source:HGNC Symbol;Acc:18317]
ENSG00000145029	3_49269758_G_T_b37	1.22E-10	0.000169998	rs2099526	NICN1	nicolin 1 [Source:HGNC Symbol;Acc:18317]
ENSG00000145029	3_49412496_A_G_b37	4.89E-09	0.000169998	rs6775069	NICN1	nicolin 1 [Source:HGNC Symbol;Acc:18317]
ENSG00000145029	3_49484992_T_C_b37	1.93E-09	0.000169998	rs11923032	NICN1	nicolin 1 [Source:HGNC Symbol;Acc:18317]
ENSG00000145029	3_49496263_G_A_b37	2.10E-09	0.000169998	rs2329025	NICN1	nicolin 1 [Source:HGNC Symbol;Acc:18317]
ENSG00000145029	3_49518551_A_G_b37	2.53E-08	0.000169998	rs7622786	NICN1	nicolin 1 [Source:HGNC Symbol;Acc:18317]
ENSG00000145029	3_49529601_G_T_b37	2.13E-09	0.000169998	rs4855858	NICN1	nicolin 1 [Source:HGNC Symbol;Acc:18317]
ENSG00000145029	3_49598064_T_C_b37	1.69E-09	0.000169998	rs9862534	NICN1	nicolin 1 [Source:HGNC Symbol;Acc:18317]
ENSG00000145029	3_49642027_C_T_b37	1.06E-05	0.000169998	rs4241405	NICN1	nicolin 1 [Source:HGNC Symbol;Acc:18317]
ENSG00000185614	3_49228946_A_G_b37	1.55E-05	0.000169998	rs11130183	FAM212A	family with sequence similarity 212, member A [Source:HGNC Symbol;Acc:32480]
ENSG00000185614	3_49269758_G_T_b37	3.63E-05	0.000169998	rs2099526	FAM212A	family with sequence similarity 212, member A [Source:HGNC Symbol;Acc:32480]
ENSG00000185614	3_49412496_A_G_b37	8.34E-05	0.000169998	rs6775069	FAM212A	family with sequence similarity 212, member A [Source:HGNC Symbol;Acc:32480]
ENSG00000185614	3_49484992_T_C_b37	6.24E-05	0.000169998	rs11923032	FAM212A	family with sequence similarity 212, member A [Source:HGNC Symbol;Acc:32480]
ENSG00000185614	3_49496263_G_A_b37	5.78E-05	0.000169998	rs2329025	FAM212A	family with sequence similarity 212, member A [Source:HGNC Symbol;Acc:32480]
ENSG00000185614	3_49518551_A_G_b37	3.84E-05	0.000169998	rs7622786	FAM212A	family with sequence similarity 212, member A [Source:HGNC Symbol;Acc:32480]
ENSG00000185614	3_49529601_G_T_b37	5.78E-05	0.000169998	rs4855858	FAM212A	family with sequence similarity 212, member A [Source:HGNC Symbol;Acc:32480]
ENSG00000185614	3_49598064_T_C_b37	3.71E-05	0.000169998	rs9862534	FAM212A	family with sequence similarity 212, member A [Source:HGNC Symbol;Acc:32480]
ENSG00000185614	3_49642027_C_T_b37	1.99E-06	0.000169998	rs4241405	FAM212A	family with sequence similarity 212, member A [Source:HGNC Symbol;Acc:32480]
ENSG00000004534	3_50047134_GT_G_b37	4.51E-10	0.000169998	rs11294686	RBM6	RNA binding motif protein 6 [Source:HGNC Symbol;Acc:9903]
ENSG00000004534	3_50065274_G_A_b37	9.34E-10	0.000169998	rs35375092	RBM6	RNA binding motif protein 6 [Source:HGNC Symbol;Acc:9903]
ENSG00000186792	3_50352458_T_G_b37	2.81E-06	0.000169998	rs9877046	HYAL3	hyaluronoglucosaminidase 3 [Source:HGNC Symbol;Acc:5322]
ENSG00000213672	3_49412015_C_T_b37	1.30E-05	0.000179998	rs6769744	NCKIPSD	NCK interacting protein with SH3 domain [Source:HGNC Symbol;Acc:15486]
ENSG00000178252	3_49412015_C_T_b37	5.87E-09	0.000179998	rs6769744	WDR6	WD repeat domain 6 [Source:HGNC Symbol;Acc:12758]
ENSG00000178252	3_49624065_T_C_b37	3.28E-05	0.000179998	rs9869120	WDR6	WD repeat domain 6 [Source:HGNC Symbol;Acc:12758]
ENSG00000067560	3_49412015_C_T_b37	4.65E-09	0.000179998	rs6769744	RHOA	ras homolog family member A [Source:HGNC Symbol;Acc:667]
ENSG00000145020	3_49412015_C_T_b37	3.54E-27	0.000179998	rs6769744	AMT	aminomethyltransferase [Source:HGNC Symbol;Acc:473]
ENSG00000145020	3_49624065_T_C_b37	2.12E-14	0.000179998	rs9869120	AMT	aminomethyltransferase [Source:HGNC Symbol;Acc:473]
ENSG00000235261	3_49412015_C_T_b37	4.59E-09	0.000179998	rs6769744	NICN1-AS1	NICN1 antisense RNA 1 [Source:HGNC Symbol;Acc:40838]
ENSG00000235261	3_49624065_T_C_b37	4.55E-07	0.000179998	rs9869120	NICN1-AS1	NICN1 antisense RNA 1 [Source:HGNC Symbol;Acc:40838]
ENSG00000145029	3_49412015_C_T_b37	2.15E-09	0.000179998	rs6769744	NICN1	nicolin 1 [Source:HGNC Symbol;Acc:18317]
ENSG00000145029	3_49624065_T_C_b37	3.71E-06	0.000179998	rs9869120	NICN1	nicolin 1 [Source:HGNC Symbol;Acc:18317]
ENSG00000185614	3_49412015_C_T_b37	5.84E-05	0.000179998	rs6769744	FAM212A	family with sequence similarity 212, member A [Source:HGNC Symbol;Acc:32480]
ENSG00000185614	3_49624065_T_C_b37	7.80E-07	0.000179998	rs9869120	FAM212A	family with sequence similarity 212, member A [Source:HGNC Symbol;Acc:32480]
ENSG00000064115	12_27114015_A_G_b37	2.48E-05	0.000179998	rs17407618	TM7SF3	transmembrane 7 superfamily member 3 [Source:HGNC Symbol;Acc:23049]
ENSG00000102606	13_111966803_G_A_b37	5.27E-06	0.000179998	rs9515402	ARHGEF7	Rho guanine nucleotide exchange factor (GEF) 7 [Source:HGNC Symbol;Acc:15607]
ENSG00000145020	3_49710444_C_G_b37	1.78E-08	0.000189998	rs13093525	AMT	aminomethyltransferase [Source:HGNC Symbol;Acc:473]
ENSG00000235261	3_49710444_C_G_b37	2.65E-05	0.000189998	rs13093525	NICN1-AS1	NICN1 antisense RNA 1 [Source:HGNC Symbol;Acc:40838]
ENSG00000004534	3_50003586_G_A_b37	6.09E-10	0.000189998	rs62262118	RBM6	RNA binding motif protein 6 [Source:HGNC Symbol;Acc:9903]
ENSG00000004534	3_50046112_G_A_b37	4.51E-10	0.000189998	rs6787892	RBM6	RNA binding motif protein 6 [Source:HGNC Symbol;Acc:9903]
ENSG00000204147	10_52486028_A_T_b37	8.69E-05	0.000189998	rs117694379	ASAH2B	N-acylsphingosine amidohydrolase (non-lysosomal ceramidase) 2B [Source:HGNC Symbol;Acc:23456]

ENSG00000232112	3_49224210_A_G_b37	4.15E-05	0.000199998	rs6446256	TMA7	translation machinery associated 7 homolog (S. cerevisiae) [Source:HGNC Symbol;Acc:26932]
ENSG00000232112	3_49307872_T_C_b37	6.55E-05	0.000199998	rs11130187	TMA7	translation machinery associated 7 homolog (S. cerevisiae) [Source:HGNC Symbol;Acc:26932]
ENSG00000213672	3_49224210_A_G_b37	1.93E-05	0.000199998	rs6446256	NCKIPSD	NCK interacting protein with SH3 domain [Source:HGNC Symbol;Acc:15486]
ENSG00000213672	3_49307872_T_C_b37	8.29E-05	0.000199998	rs11130187	NCKIPSD	NCK interacting protein with SH3 domain [Source:HGNC Symbol;Acc:15486]
ENSG00000178252	3_49224210_A_G_b37	7.21E-10	0.000199998	rs6446256	WDR6	WD repeat domain 6 [Source:HGNC Symbol;Acc:12758]
ENSG00000178252	3_49307872_T_C_b37	1.92E-09	0.000199998	rs11130187	WDR6	WD repeat domain 6 [Source:HGNC Symbol;Acc:12758]
ENSG00000067560	3_49224210_A_G_b37	3.76E-08	0.000199998	rs6446256	RHOA	ras homolog family member A [Source:HGNC Symbol;Acc:667]
ENSG00000067560	3_49307872_T_C_b37	5.86E-08	0.000199998	rs11130187	RHOA	ras homolog family member A [Source:HGNC Symbol;Acc:667]
ENSG00000145020	3_49224210_A_G_b37	2.90E-26	0.000199998	rs6446256	AMT	aminomethyltransferase [Source:HGNC Symbol;Acc:473]
ENSG00000145020	3_49307872_T_C_b37	2.75E-25	0.000199998	rs11130187	AMT	aminomethyltransferase [Source:HGNC Symbol;Acc:473]
ENSG00000235261	3_49224210_A_G_b37	1.66E-10	0.000199998	rs6446256	NICN1-AS1	NICN1 antisense RNA 1 [Source:HGNC Symbol;Acc:40838]
ENSG00000235261	3_49307872_T_C_b37	1.31E-10	0.000199998	rs11130187	NICN1-AS1	NICN1 antisense RNA 1 [Source:HGNC Symbol;Acc:40838]
ENSG00000145029	3_49224210_A_G_b37	7.92E-11	0.000199998	rs6446256	NICN1	nicolin 1 [Source:HGNC Symbol;Acc:18317]
ENSG00000145029	3_49307872_T_C_b37	5.51E-11	0.000199998	rs11130187	NICN1	nicolin 1 [Source:HGNC Symbol;Acc:18317]
ENSG00000185614	3_49224210_A_G_b37	1.33E-05	0.000199998	rs6446256	FAM212A	family with sequence similarity 212, member A [Source:HGNC Symbol;Acc:32480]
ENSG00000185614	3_49307872_T_C_b37	3.09E-05	0.000199998	rs11130187	FAM212A	family with sequence similarity 212, member A [Source:HGNC Symbol;Acc:32480]
ENSG00000004534	3_49976144_TC_T_b37	6.09E-10	0.000199998	rs200537286	RBM6	RNA binding motif protein 6 [Source:HGNC Symbol;Acc:9903]
ENSG00000147894	9_27586162_A_G_b37	4.63E-07	0.000199998	rs2453556	C9orf72	chromosome 9 open reading frame 72 [Source:HGNC Symbol;Acc:28337]
ENSG00000232112	3_49216472_T_C_b37	2.44E-05	0.000209998	rs9834003	TMA7	translation machinery associated 7 homolog (S. cerevisiae) [Source:HGNC Symbol;Acc:26932]
ENSG00000213672	3_49216472_T_C_b37	1.48E-05	0.000209998	rs9834003	NCKIPSD	NCK interacting protein with SH3 domain [Source:HGNC Symbol;Acc:15486]
ENSG00000178252	3_49216472_T_C_b37	8.37E-10	0.000209998	rs9834003	WDR6	WD repeat domain 6 [Source:HGNC Symbol;Acc:12758]
ENSG00000067560	3_49216472_T_C_b37	4.72E-08	0.000209998	rs9834003	RHOA	ras homolog family member A [Source:HGNC Symbol;Acc:667]
ENSG00000145020	3_49216472_T_C_b37	2.97E-26	0.000209998	rs9834003	AMT	aminomethyltransferase [Source:HGNC Symbol;Acc:473]
ENSG00000145020	3_49628155_CATA_C_b37	4.42E-13	0.000209998	rs74693643	AMT	aminomethyltransferase [Source:HGNC Symbol;Acc:473]
ENSG00000235261	3_49216472_T_C_b37	2.07E-10	0.000209998	rs9834003	NICN1-AS1	NICN1 antisense RNA 1 [Source:HGNC Symbol;Acc:40838]
ENSG00000235261	3_49628155_CATA_C_b37	1.67E-05	0.000209998	rs74693643	NICN1-AS1	NICN1 antisense RNA 1 [Source:HGNC Symbol;Acc:40838]
ENSG00000145029	3_49216472_T_C_b37	1.36E-10	0.000209998	rs9834003	NICN1	nicolin 1 [Source:HGNC Symbol;Acc:18317]
ENSG00000145029	3_49628155_CATA_C_b37	6.10E-05	0.000209998	rs74693643	NICN1	nicolin 1 [Source:HGNC Symbol;Acc:18317]
ENSG00000185614	3_49216472_T_C_b37	1.11E-05	0.000209998	rs9834003	FAM212A	family with sequence similarity 212, member A [Source:HGNC Symbol;Acc:32480]
ENSG00000185614	3_49628155_CATA_C_b37	3.14E-06	0.000209998	rs74693643	FAM212A	family with sequence similarity 212, member A [Source:HGNC Symbol;Acc:32480]
ENSG00000004534	3_49987475_C_T_b37	6.09E-10	0.000209998	rs7615318	RBM6	RNA binding motif protein 6 [Source:HGNC Symbol;Acc:9903]
ENSG00000004534	3_50088943_A_G_b37	1.80E-14	0.000209998	rs6446197	RBM6	RNA binding motif protein 6 [Source:HGNC Symbol;Acc:9903]
ENSG00000204644	6_30436560_A_G_b37	5.00E-08	0.000209998	rs115256213	ZFP57	ZFP57 zinc finger protein [Source:HGNC Symbol;Acc:18791]
ENSG00000204632	6_30436560_A_G_b37	7.17E-08	0.000209998	rs115256213	HLA-G	major histocompatibility complex, class I, G [Source:HGNC Symbol;Acc:4964]
ENSG00000230795	6_30436560_A_G_b37	4.02E-06	0.000209998	rs115256213	HLA-K	major histocompatibility complex, class I, K (pseudogene) [Source:HGNC Symbol;Acc:4969]
ENSG00000204625	6_30436560_A_G_b37	1.43E-07	0.000209998	rs115256213	HCG9	HLA complex group 9 (non-protein coding) [Source:HGNC Symbol;Acc:21243]
ENSG00000232112	3_49220448_G_A_b37	3.19E-05	0.000219998	rs4955423	TMA7	translation machinery associated 7 homolog (S. cerevisiae) [Source:HGNC Symbol;Acc:26932]
ENSG00000213672	3_49220448_G_A_b37	1.69E-05	0.000219998	rs4955423	NCKIPSD	NCK interacting protein with SH3 domain [Source:HGNC Symbol;Acc:15486]
ENSG00000213672	3_49449638_G_A_b37	1.13E-05	0.000219998	rs940045	NCKIPSD	NCK interacting protein with SH3 domain [Source:HGNC Symbol;Acc:15486]
ENSG00000178252	3_49220448_G_A_b37	7.65E-10	0.000219998	rs4955423	WDR6	WD repeat domain 6 [Source:HGNC Symbol;Acc:12758]
ENSG00000178252	3_49449638_G_A_b37	6.94E-09	0.000219998	rs940045	WDR6	WD repeat domain 6 [Source:HGNC Symbol;Acc:12758]
ENSG00000067560	3_49220448_G_A_b37	4.15E-08	0.000219998	rs4955423	RHOA	ras homolog family member A [Source:HGNC Symbol;Acc:667]
ENSG00000067560	3_49449638_G_A_b37	3.96E-09	0.000219998	rs940045	RHOA	ras homolog family member A [Source:HGNC Symbol;Acc:667]
ENSG00000145020	3_49220448_G_A_b37	2.80E-26	0.000219998	rs4955423	AMT	aminomethyltransferase [Source:HGNC Symbol;Acc:473]
ENSG00000145020	3_49449638_G_A_b37	4.67E-27	0.000219998	rs940045	AMT	aminomethyltransferase [Source:HGNC Symbol;Acc:473]
ENSG00000235261	3_49220448_G_A_b37	1.82E-10	0.000219998	rs4955423	NICN1-AS1	NICN1 antisense RNA 1 [Source:HGNC Symbol;Acc:40838]
ENSG00000235261	3_49449638_G_A_b37	8.84E-09	0.000219998	rs940045	NICN1-AS1	NICN1 antisense RNA 1 [Source:HGNC Symbol;Acc:40838]
ENSG00000145029	3_49220448_G_A_b37	1.01E-10	0.000219998	rs4955423	NICN1	nicolin 1 [Source:HGNC Symbol;Acc:18317]
ENSG00000145029	3_49449638_G_A_b37	3.02E-09	0.000219998	rs940045	NICN1	nicolin 1 [Source:HGNC Symbol;Acc:18317]
ENSG00000185614	3_49220448_G_A_b37	1.21E-05	0.000219998	rs4955423	FAM212A	family with sequence similarity 212, member A [Source:HGNC Symbol;Acc:32480]
ENSG00000185614	3_49449638_G_A_b37	6.55E-05	0.000219998	rs940045	FAM212A	family with sequence similarity 212, member A [Source:HGNC Symbol;Acc:32480]
ENSG00000004534	3_49997193_C_T_b37	6.51E-10	0.000219998	rs13083396	RBM6	RNA binding motif protein 6 [Source:HGNC Symbol;Acc:9903]
ENSG00000227354	13_80207603_A_C_b37	4.16E-18	0.000219998	rs58739959	RBM26-AS1	RBM26 antisense RNA 1 [Source:HGNC Symbol;Acc:39805]
ENSG00000227354	13_80216147_A_G_b37	1.78E-19	0.000219998	rs6563127	RBM26-AS1	RBM26 antisense RNA 1 [Source:HGNC Symbol;Acc:39805]
ENSG00000004534	3_49899795_G_A_b37	3.13E-15	0.000229998	rs3796386	RBM6	RNA binding motif protein 6 [Source:HGNC Symbol;Acc:9903]
ENSG00000004534	3_50120257_A_G_b37	6.33E-10	0.000229998	rs55924524	RBM6	RNA binding motif protein 6 [Source:HGNC Symbol;Acc:9903]
ENSG00000004534	3_50126215_C_A_b37	1.38E-14	0.000229998	rs2247510	RBM6	RNA binding motif protein 6 [Source:HGNC Symbol;Acc:9903]
ENSG00000004534	3_50136463_G_A_b37	6.01E-10	0.000229998	rs13095697	RBM6	RNA binding motif protein 6 [Source:HGNC Symbol;Acc:9903]
ENSG00000186792	3_50357869_A_C_b37	1.25E-06	0.000229998	rs709210	HYAL3	hyaluronoglucosaminidase 3 [Source:HGNC Symbol;Acc:5322]
ENSG00000260787	15_77312826_T_C_b37	7.58E-06	0.000229998	rs16968627	RP11-797A18.4	
ENSG00000213672	3_49533094_C_G_b37	1.04E-05	0.000239998	rs7374731	NCKIPSD	NCK interacting protein with SH3 domain [Source:HGNC Symbol;Acc:15486]
ENSG00000178252	3_49533094_C_G_b37	4.04E-09	0.000239998	rs7374731	WDR6	WD repeat domain 6 [Source:HGNC Symbol;Acc:12758]
ENSG00000067560	3_49533094_C_G_b37	5.83E-09	0.000239998	rs7374731	RHOA	ras homolog family member A [Source:HGNC Symbol;Acc:667]
ENSG00000145020	3_49533094_C_G_b37	2.07E-27	0.000239998	rs7374731	AMT	aminomethyltransferase [Source:HGNC Symbol;Acc:473]
ENSG00000235261	3_49533094_C_G_b37	7.96E-09	0.000239998	rs7374731	NICN1-AS1	NICN1 antisense RNA 1 [Source:HGNC Symbol;Acc:40838]
ENSG00000145029	3_49533094_C_G_b37	2.03E-09	0.000239998	rs7374731	NICN1	nicolin 1 [Source:HGNC Symbol;Acc:18317]
ENSG00000185614	3_49533094_C_G_b37	3.19E-05	0.000239998	rs7374731	FAM212A	family with sequence similarity 212, member A [Source:HGNC Symbol;Acc:32480]
ENSG00000166669	16_10494325_T_G_b37	6.10E-05	0.000239998	rs12925855	ATF7IP2	activating transcription factor 7 interacting protein 2 [Source:HGNC Symbol;Acc:20397]
ENSG00000223745	1_93810436_T_A_b37	1.85E-31	0.000249998	rs3067470	RP4-717I23.3	
ENSG00000145020	3_49724808_T_C_b37	3.48E-09	0.000259997	rs3020779	AMT	aminomethyltransferase [Source:HGNC Symbol;Acc:473]
ENSG00000235261	3_49724808_T_C_b37	3.05E-05	0.000259997	rs3020779	NICN1-AS1	NICN1 antisense RNA 1 [Source:HGNC Symbol;Acc:40838]
ENSG00000145029	3_49724808_T_C_b37	4.97E-05	0.000259997	rs3020779	NICN1	nicolin 1 [Source:HGNC Symbol;Acc:18317]
ENSG00000185614	3_49724808_T_C_b37	2.90E-06	0.000259997	rs3020779	FAM212A	family with sequence similarity 212, member A [Source:HGNC Symbol;Acc:32480]
ENSG00000004534	3_49902160_T_C_b37	3.13E-15	0.000279997	rs2883059	RBM6	RNA binding motif protein 6 [Source:HGNC Symbol;Acc:9903]
ENSG00000164078	3_50067350_T_C_b37	7.46E-05	0.000289997	rs35849525	MST1R	macrophage stimulating 1 receptor (c-met-related tyrosine kinase) [Source:HGNC Symbol;Acc:7381]
ENSG00000004534	3_49991060_C_T_b37	5.83E-10	0.000289997	rs71326904	RBM6	RNA binding motif protein 6 [Source:HGNC Symbol;Acc:9903]
ENSG00000004534	3_50067350_T_C_b37	1.25E-10	0.000289997	rs35849525	RBM6	RNA binding motif protein 6 [Source:HGNC Symbol;Acc:9903]
ENSG00000204147	10_52488890_G_T_b37	5.85E-07	0.000289997	rs12780210	ASAH2B	N-acylsphingosine amidohydrolase (non-lysosomal ceramidase) 2B [Source:HGNC Symbol;Acc:23456]
ENSG00000178252	3_49708574_TGTGC_T_b37	3.29E-05	0.000309997	rs200763649	WDR6	WD repeat domain 6 [Source:HGNC Symbol;Acc:12758]
ENSG00000145020	3_49708574_TGTGC_T_b37	2.24E-08	0.000309997	rs200763649	AMT	aminomethyltransferase [Source:HGNC Symbol;Acc:473]
ENSG00000145020	3_49887278_T_C_b37	1.43E-05	0.000309997	rs55901550	AMT	aminomethyltransferase [Source:HGNC Symbol;Acc:473]
ENSG00000185614	3_49708574_TGTGC_T_b37	7.35E-05	0.000309997	rs200763649	FAM212A	family with sequence similarity 212, member A [Source:HGNC Symbol;Acc:32480]
ENSG00000185614	3_49887278_T_C_b37	1.92E-05	0.000309997	rs55901550	FAM212A	family with sequence similarity 212, member A [Source:HGNC Symbol;Acc:32480]
ENSG00000143067	1_120148215_GC_G_b37	9.95E-05	0.000309997	rs587638658	ZNF697	zinc finger protein 697 [Source:HGNC Symbol;Acc:32034]
ENSG00000227354	13_80204753_C_A_b37	1.78E-19	0.000309997	rs6563125	RBM26-AS1	RBM26 antisense RNA 1 [Source:HGNC Symbol;Acc:39805]
ENSG00000004534	3_50085153_A_G_b37	1.05E-10	0.000329997	rs17304079	RBM6	RNA binding motif protein 6 [Source:HGNC Symbol;Acc:9903]
ENSG00000213672	3_49488984_T_C_b37	4.55E-05	0.000339997	rs4855869	NCKIPSD	NCK interacting protein with SH3 domain [Source:HGNC Symbol;Acc:15486]
ENSG00000178252	3_49488984_T_C_b37	3.10E-08	0.000339997	rs4855869	WDR6	WD repeat domain 6 [Source:HGNC Symbol;Acc:12758]
ENSG00000067560	3_49488984_T_C_b37	1.30E-08	0.000339997	rs4855869	RHOA	ras homolog family member A [Source:HGNC Symbol;Acc:667]
ENSG00000145020	3_49488984_T_C_b37	1.17E-25	0.000339997	rs4855869	AMT	aminomethyltransferase [Source:HGNC Symbol;Acc:473]
ENSG00000235261	3_49488984_T_C_b37	1.78E-08	0.000339997	rs4855869	NICN1-AS1	NICN1 antisense RNA 1 [Source:HGNC Symbol;Acc:40838]
ENSG00000145029	3_49488984_T_C_b37	5.10E-09	0.000339997	rs4855869	NICN1	nicolin 1 [Source:HGNC Symbol;Acc:18317]
ENSG00000004534	3_50165101_T_C_b37	2.16E-14	0.000339997	rs2624848	RBM6	RNA binding motif protein 6 [Source:HGNC Symbol;Acc:9903]
ENSG00000004534	3_49908023_G_A_b37	3.20E-15	0.000349997	rs952594	RBM6	RNA binding motif protein 6 [Source:HGNC Symbol;Acc:9903]
ENSG00000225213	10_16748793_G_C_b37	8.14E-05	0.000349997	rs4523590	RP11-197M22.2	
ENSG00000213672	3_49328833_A_C_b37	4.99E-05	0.000359996	rs9811047	NCKIPSD	NCK interacting protein with SH3 domain [Source:HGNC Symbol;Acc:15486]
ENSG00000178252	3_49328833_A_C_b37	3.44E-07	0.000359996	rs9811047	WDR6	WD repeat domain 6 [Source:HGNC Symbol;Acc:12758]
ENSG00000067560	3_49328833_A_C_b37	1.76E-08	0.000359996	rs9811047	RHOA	ras homolog family member A [Source:HGNC Symbol;Acc:667]
ENSG00000145020	3_49328833_A_C_b37	5.54E-25	0.000359996	rs9811047	AMT	aminomethyltransferase [Source:HGNC Symbol;Acc:473]
ENSG00000235261	3_49328833_A_C_b37	2.18E-11	0.000359996	rs9811047	NICN1-AS1	NICN1 antisense RNA 1 [Source:HGNC Symbol;Acc:40838]
ENSG00000145029	3_49328833_A_C_b37	3.13E-11	0.000359996	rs9811047	NICN1	nicolin 1 [Source:HGNC Symbol;Acc:18317]
ENSG00000004534	3_50108948_G_GAGTC_b37	1.00E-10	0.000359996	rs55914752	RBM6	RNA binding motif protein 6 [Source:HGNC Symbol;Acc:9903]
ENSG00000213672	3_49408816_G_C_b37	1.31E-05	0.000369996	rs6796147	NCKIPSD	NCK interacting protein with SH3 domain [Source:HGNC Symbol;Acc:15486]
ENSG00000178252	3_49408816_G_C_b37	1.48E-09	0.000369996	rs6796147	WDR6	WD repeat domain 6 [Source:HGNC Symbol;Acc:12758]
ENSG00000067560	3_49408816_G_C_b37	1.49E-09	0.000369996	rs6796147	RHOA	ras homolog family member A [Source:HGNC Symbol;Acc:667]
ENSG00000145020	3_49408816_G_C_b37	6.58E-26	0.000369996	rs6796147	AMT	aminomethyltransferase [Source:HGNC Symbol;Acc:473]
ENSG00000235261	3_49408816_G_C_b37	5.84E-09	0.000369996	rs6796147	NICN1-AS1	NICN1 antisense RNA 1 [Source:HGNC Symbol;Acc:40838]
ENSG00000145029	3_49408816_G_C_b37	8.80E-09	0.000369996	rs6796147	NICN1	nicolin 1 [Source:HGNC Symbol;Acc:18317]
ENSG00000185614	3_49408816_G_C_b37	3.17E-05	0.000369996	rs6796147	FAM212A	family with sequence similarity 212, member A [Source:HGNC Symbol;Acc:32480]
ENSG00000264247	18_72388641_T_C_b37	1.13E-09	0.000369996	rs12959304	LINC00909	long intergenic non-protein coding RNA 909 [Source:HGNC Symbol;Acc:44331]
ENSG00000215790	1_1666809_G_A_b37	8.37E-05	0.000379996	rs2076326	SLC35E2	solute carrier family 35, member E2 [Source:HGNC Symbol;Acc:20863]
ENSG00000169885	1_1666809_G_A_b37	4.42E-11	0.000379996	rs2076326	CALML6	calmodulin-like 6 [Source:HGNC Symbol;Acc:24193]
ENSG00000178821	1_1666809_G_A_b37	2.52E-07	0.000379996	rs2076326	TMEM52	transmembrane protein 52 [Source:HGNC Symbol;Acc:27916]

ENSG00000004534	3_50135412_A_C_b37	9.43E-11	0.000389996	rs7648652	RBM6	RNA binding motif protein 6 [Source:HGNC Symbol;Acc:9903]
ENSG00000240045	3_155005811_A_G_b37	1.12E-05	0.000399996	rs62279098	RP11-451G4.2	
ENSG00000102606	13_111968025_T_A_b37	1.04E-05	0.000409996	rs9515403	ARHGEF7	Rho guanine nucleotide exchange factor (GEF) 7 [Source:HGNC Symbol;Acc:15607]
ENSG00000004534	3_49993453_G_GA_b37	5.81E-10	0.000419996	rs199936500	RBM6	RNA binding motif protein 6 [Source:HGNC Symbol;Acc:9903]
ENSG00000232112	3_48412260_AC_A_b37	4.28E-07	0.000429996	rs200936725	TMA7	translation machinery associated 7 homolog (S. cerevisiae) [Source:HGNC Symbol;Acc:26932]
ENSG00000244380	3_48412260_AC_A_b37	1.18E-05	0.000429996	rs200936725	RP11-24C3.2	
ENSG00000213672	3_48412260_AC_A_b37	3.27E-05	0.000429996	rs200936725	NCKIPSD	NCK interacting protein with SH3 domain [Source:HGNC Symbol;Acc:15486]
ENSG00000229980	17_48984310_G_A_b37	4.04E-05	0.000439996	rs11079939	TOB1-AS1	TOB1 antisense RNA 1 [Source:HGNC Symbol;Acc:44340]
ENSG00000240399	12_48403837_GC_G_b37	6.03E-05	0.000449996	rs68168422	RP1-228P16.1	
ENSG00000004534	3_50078221_T_C_b37	2.30E-15	0.000459995	rs7430334	RBM6	RNA binding motif protein 6 [Source:HGNC Symbol;Acc:9903]
ENSG00000215790	1_1667323_C_T_b37	9.43E-05	0.000459995	rs78368772	SLC35E2	solute carrier family 35, member E2 [Source:HGNC Symbol;Acc:20863]
ENSG00000169885	1_1667323_C_T_b37	4.26E-11	0.000459995	rs78368772	CALML6	calmodulin-like 6 [Source:HGNC Symbol;Acc:24193]
ENSG00000178821	1_1667323_C_T_b37	2.57E-07	0.000459995	rs78368772	TMEM52	transmembrane protein 52 [Source:HGNC Symbol;Acc:27916]
ENSG00000178462	10_5492027_TGTCA_T_b37	4.30E-08	0.000459995	rs66980083	TUBAL3	tubulin, alpha-like 3 [Source:HGNC Symbol;Acc:23534]
ENSG00000225213	10_16763882_A_G_b37	4.10E-06	0.000469995	rs10732931	RP11-197M22.2	
ENSG00000159461	16_57107653_A_G_b37	2.16E-05	0.000469995	rs7201800	AMFR	autocrine motility factor receptor, E3 ubiquitin protein ligase [Source:HGNC Symbol;Acc:463]
ENSG00000213672	3_49378797_A_T_b37	2.02E-05	0.000499995	rs9834535	NCKIPSD	NCK interacting protein with SH3 domain [Source:HGNC Symbol;Acc:15486]
ENSG00000178252	3_49378797_A_T_b37	6.70E-09	0.000499995	rs9834535	WDR6	WD repeat domain 6 [Source:HGNC Symbol;Acc:12758]
ENSG00000067560	3_49378797_A_T_b37	3.74E-08	0.000499995	rs9834535	RHOA	ras homolog family member A [Source:HGNC Symbol;Acc:667]
ENSG00000145020	3_49378797_A_T_b37	5.14E-26	0.000499995	rs9834535	AMT	aminomethyltransferase [Source:HGNC Symbol;Acc:473]
ENSG00000235261	3_49378797_A_T_b37	1.83E-10	0.000499995	rs9834535	NICN1-AS1	NICN1 antisense RNA 1 [Source:HGNC Symbol;Acc:40838]
ENSG00000145029	3_49378797_A_T_b37	1.62E-10	0.000499995	rs9834535	NICN1	nicolin 1 [Source:HGNC Symbol;Acc:18317]
ENSG00000185614	3_49378797_A_T_b37	2.91E-05	0.000499995	rs9834535	FAM212A	family with sequence similarity 212, member A [Source:HGNC Symbol;Acc:32480]
ENSG00000004534	3_50043341_A_T_b37	1.30E-14	0.000499995	rs12634780	RBM6	RNA binding motif protein 6 [Source:HGNC Symbol;Acc:9903]
ENSG00000159461	16_57109161_C_T_b37	2.14E-05	0.000499995	rs113689453	AMFR	autocrine motility factor receptor, E3 ubiquitin protein ligase [Source:HGNC Symbol;Acc:463]
