# Supplementary material for: Meta-Analysis of Polymyositis and Dermatomyositis Microarray Data Reveals Novel Genetic Biomarkers
Source: Genes (Basel). 2019 Oct 30;10(11):864. doi: 10.3390/genes10110864 (PMC6895911; doi:10.3390/genes10110864)
Supplement: Supplementary file 1 [file genes-10-00864-s001.zip › Supplementary_files/Supplementary Table 4.pdf]

Supplementary Table 4. Detected SNPs and eGenes in sun-exposed skin tissue.

gene	SNP	GTEX_p_value	p_value	rs_id_dbSNP142_GRCh37p13	external_gene_name	description
ENSG00000235725	2_65836286_A_G_b37	6.86E-09	9.00E-06	rs17030469	AC007389.3	
ENSG00000235725	2_65835876_A_G_b37	2.01E-09	1.20E-05	rs17030462	AC007389.3	
ENSG00000087076	19_49381273_TCC_T_b37	9.41E-10	1.20E-05	rs201219796	HSD17B14	hydroxysteroid (17-beta) dehydrogenase 14 [Source:HGNC Symbol;Acc:23238]
ENSG00000105559	19_49381273_TCC_T_b37	5.19E-05	1.20E-05	rs201219796	PLEKHA4	pleckstrin homology domain containing, family A (phosphoinositide binding specific) member 4 [Source:HGNC Symbol;Acc:14339]
ENSG00000235725	2_65844734_T_C_b37	2.98E-10	1.60E-05	rs6760617	AC007389.3	
ENSG00000120539	10_27275804_T_C_b37	9.44E-05	3.50E-05	rs149490734	MASTL	microtubule associated serine/threonine kinase-like [Source:HGNC Symbol;Acc:19042]
ENSG00000120539	10_27271427_T_C_b37	9.44E-05	5.10E-05	rs78379439	MASTL	microtubule associated serine/threonine kinase-like [Source:HGNC Symbol;Acc:19042]
ENSG00000131042	19_54791788_A_G_b37	5.47E-07	6.20E-05	rs397600	LILRB2	leukocyte immunoglobulin-like receptor, subfamily B (with TM and ITIM domains), member 2 [Source:HGNC Symbol;Acc:6606]
ENSG00000111554	12_68736461_C_A_b37	6.31E-06	7.00E-05	rs117565525	MDM1	Mdm1 nuclear protein homolog (mouse) [Source:HGNC Symbol;Acc:29917]
ENSG00000155542	5_56215203_G_A_b37	3.87E-14	8.00E-05	rs80310300	SETD9	SET domain containing 9 [Source:HGNC Symbol;Acc:28508]
ENSG00000155542	5_56256606_G_A_b37	1.53E-14	1.00E-04	rs79683734	SETD9	SET domain containing 9 [Source:HGNC Symbol;Acc:28508]
ENSG00000108264	17_35766475_A_G_b37	1.54E-25	1.00E-04	rs72828246	TADA2A	transcriptional adaptor 2A [Source:HGNC Symbol;Acc:11531]
ENSG00000006114	17_35766475_A_G_b37	4.48E-21	1.00E-04	rs72828246	SYNRG	synergin, gamma [Source:HGNC Symbol;Acc:557]
ENSG00000155542	5_56258685_G_GAT_b37	1.52E-14	0.000109999	rs77039815	SETD9	SET domain containing 9 [Source:HGNC Symbol;Acc:28508]
ENSG00000182853	17_4659152_C_T_b37	1.21E-06	0.000119999	rs12946532	VMO1	vitelline membrane outer layer 1 homolog (chicken) [Source:HGNC Symbol;Acc:30387]
ENSG00000108264	17_35760056_C_T_b37	1.41E-24	0.000119999	rs1112173	TADA2A	transcriptional adaptor 2A [Source:HGNC Symbol;Acc:11531]
ENSG00000108264	17_35762107_G_C_b37	6.13E-26	0.000119999	rs878520	TADA2A	transcriptional adaptor 2A [Source:HGNC Symbol;Acc:11531]
ENSG00000006114	17_35760056_C_T_b37	5.17E-21	0.000119999	rs1112173	SYNRG	synergin, gamma [Source:HGNC Symbol;Acc:557]
ENSG00000006114	17_35762107_G_C_b37	5.76E-20	0.000119999	rs878520	SYNRG	synergin, gamma [Source:HGNC Symbol;Acc:557]
ENSG00000087076	19_49380865_G_A_b37	2.20E-09	0.000119999	rs516983	HSD17B14	hydroxysteroid (17-beta) dehydrogenase 14 [Source:HGNC Symbol;Acc:23238]
ENSG00000105559	19_49380865_G_A_b37	3.08E-06	0.000119999	rs516983	PLEKHA4	pleckstrin homology domain containing, family A (phosphoinositide binding specific) member 4 [Source:HGNC Symbol;Acc:14339]
ENSG00000108264	17_35755546_A_G_b37	2.16E-25	0.000129999	rs113593735	TADA2A	transcriptional adaptor 2A [Source:HGNC Symbol;Acc:11531]
ENSG00000006114	17_35755546_A_G_b37	4.25E-21	0.000129999	rs113593735	SYNRG	synergin, gamma [Source:HGNC Symbol;Acc:557]
ENSG00000111554	12_68649851_C_T_b37	5.91E-05	0.000139999	rs140421991	MDM1	Mdm1 nuclear protein homolog (mouse) [Source:HGNC Symbol;Acc:29917]
ENSG00000108264	17_35759965_A_T_b37	1.84E-25	0.000139999	rs1112174	TADA2A	transcriptional adaptor 2A [Source:HGNC Symbol;Acc:11531]
ENSG00000006114	17_35759965_A_T_b37	4.26E-21	0.000139999	rs1112174	SYNRG	synergin, gamma [Source:HGNC Symbol;Acc:557]
ENSG00000182853	17_4659688_C_T_b37	1.82E-06	0.000149999	rs4572467	VMO1	vitelline membrane outer layer 1 homolog (chicken) [Source:HGNC Symbol;Acc:30387]
ENSG00000092036	14_23322613_C_T_b37	1.16E-07	0.000159998	rs4982695	HAUS4	HAUS augmin-like complex, subunit 4 [Source:HGNC Symbol;Acc:20163]
ENSG00000108264	17_35766563_CA_C_b37	4.53E-25	0.000159998	rs564400766	TADA2A	transcriptional adaptor 2A [Source:HGNC Symbol;Acc:11531]
ENSG00000006114	17_35766563_CA_C_b37	3.19E-20	0.000159998	rs564400766	SYNRG	synergin, gamma [Source:HGNC Symbol;Acc:557]
ENSG00000269973	2_9960648_C_T_b37	6.75E-06	0.000169998	rs78272618	RP11-95D17.1	
ENSG00000235725	2_65825974_TA_T_b37	4.70E-07	0.000169998	rs5831762	AC007389.3	
ENSG00000093183	3_42580706_G_A_b37	2.45E-05	0.000169998	rs342508	SEC22C	SEC22 vesicle trafficking protein homolog C (S. cerevisiae) [Source:HGNC Symbol;Acc:16828]
ENSG00000114857	3_42580706_G_A_b37	2.15E-05	0.000169998	rs342508	NKTR	natural killer-tumor recognition sequence [Source:HGNC Symbol;Acc:7833]
ENSG00000093183	3_42586704_C_A_b37	3.41E-05	0.000179998	rs6799021	SEC22C	SEC22 vesicle trafficking protein homolog C (S. cerevisiae) [Source:HGNC Symbol;Acc:16828]
ENSG00000108264	17_35791505_A_C_b37	5.42E-25	0.000179998	rs853225	TADA2A	transcriptional adaptor 2A [Source:HGNC Symbol;Acc:11531]
ENSG00000108264	17_35800919_A_G_b37	5.23E-25	0.000179998	rs853229	TADA2A	transcriptional adaptor 2A [Source:HGNC Symbol;Acc:11531]
ENSG00000006114	17_35791505_A_C_b37	1.07E-22	0.000179998	rs853225	SYNRG	synergin, gamma [Source:HGNC Symbol;Acc:557]
ENSG00000006114	17_35800919_A_G_b37	1.05E-22	0.000179998	rs853229	SYNRG	synergin, gamma [Source:HGNC Symbol;Acc:557]
ENSG00000235725	2_65823020_A_G_b37	2.02E-07	0.000189998	rs11695927	AC007389.3	
ENSG00000182853	17_4670972_G_C_b37	3.78E-07	0.000189998	rs8069974	VMO1	vitelline membrane outer layer 1 homolog (chicken) [Source:HGNC Symbol;Acc:30387]
ENSG00000108264	17_35783812_A_G_b37	5.35E-25	0.000189998	rs7501983	TADA2A	transcriptional adaptor 2A [Source:HGNC Symbol;Acc:11531]
ENSG00000108264	17_35793286_G_C_b37	5.75E-25	0.000189998	rs860468	TADA2A	transcriptional adaptor 2A [Source:HGNC Symbol;Acc:11531]
ENSG00000006114	17_35783812_A_G_b37	1.09E-22	0.000189998	rs7501983	SYNRG	synergin, gamma [Source:HGNC Symbol;Acc:557]
ENSG00000006114	17_35793286_G_C_b37	1.46E-22	0.000189998	rs860468	SYNRG	synergin, gamma [Source:HGNC Symbol;Acc:557]
ENSG00000235725	2_65813030_G_C_b37	3.64E-11	0.000199998	rs7607271	AC007389.3	
ENSG00000235725	2_65819883_C_A_b37	1.31E-13	0.000199998	rs1115848	AC007389.3	
ENSG00000108264	17_35768827_T_A_b37	5.19E-25	0.000199998	rs56186148	TADA2A	transcriptional adaptor 2A [Source:HGNC Symbol;Acc:11531]
ENSG00000006114	17_35768827_T_A_b37	1.07E-22	0.000199998	rs56186148	SYNRG	synergin, gamma [Source:HGNC Symbol;Acc:557]
ENSG00000087076	19_49396623_C_T_b37	2.15E-06	0.000199998	rs112370206	HSD17B14	hydroxysteroid (17-beta) dehydrogenase 14 [Source:HGNC Symbol;Acc:23238]
ENSG00000235725	2_65824380_C_G_b37	1.31E-13	0.000209998	rs12614575	AC007389.3	
ENSG00000182853	17_4668491_G_A_b37	1.85E-07	0.000209998	rs35473600	VMO1	vitelline membrane outer layer 1 homolog (chicken) [Source:HGNC Symbol;Acc:30387]
ENSG00000182853	17_4673609_T_C_b37	3.63E-08	0.000209998	rs9890797	VMO1	vitelline membrane outer layer 1 homolog (chicken) [Source:HGNC Symbol;Acc:30387]
ENSG00000182853	17_4676420_G_A_b37	3.37E-08	0.000209998	rs2089483	VMO1	vitelline membrane outer layer 1 homolog (chicken) [Source:HGNC Symbol;Acc:30387]
ENSG00000108264	17_35842517_A_G_b37	6.40E-23	0.000209998	rs853233	TADA2A	transcriptional adaptor 2A [Source:HGNC Symbol;Acc:11531]
ENSG00000006114	17_35842517_A_G_b37	4.87E-23	0.000209998	rs853233	SYNRG	synergin, gamma [Source:HGNC Symbol;Acc:557]
ENSG00000261349	1_25535823_C_T_b37	4.54E-07	0.000219998	rs4649079	RP3-465N24.5	
ENSG00000187010	1_25535823_C_T_b37	9.62E-06	0.000219998	rs4649079	RHD	Rh blood group, D antigen [Source:HGNC Symbol;Acc:10009]
ENSG00000235725	2_65824325_C_G_b37	1.31E-13	0.000219998	rs12614551	AC007389.3	
ENSG00000182853	17_4685228_G_A_b37	4.20E-09	0.000219998	rs34460487	VMO1	vitelline membrane outer layer 1 homolog (chicken) [Source:HGNC Symbol;Acc:30387]
ENSG00000108264	17_35809215_T_C_b37	5.05E-25	0.000219998	rs2522964	TADA2A	transcriptional adaptor 2A [Source:HGNC Symbol;Acc:11531]
ENSG00000108264	17_35844422_A_C_b37	2.31E-24	0.000219998	rs2680721	TADA2A	transcriptional adaptor 2A [Source:HGNC Symbol;Acc:11531]
ENSG00000006114	17_35809215_T_C_b37	1.16E-22	0.000219998	rs2522964	SYNRG	synergin, gamma [Source:HGNC Symbol;Acc:557]
ENSG00000006114	17_35844422_A_C_b37	4.41E-23	0.000219998	rs2680721	SYNRG	synergin, gamma [Source:HGNC Symbol;Acc:557]
ENSG00000087076	19_49380836_G_C_b37	1.85E-10	0.000219998	rs661186	HSD17B14	hydroxysteroid (17-beta) dehydrogenase 14 [Source:HGNC Symbol;Acc:23238]
ENSG00000105559	19_49380836_G_C_b37	2.45E-05	0.000219998	rs661186	PLEKHA4	pleckstrin homology domain containing, family A (phosphoinositide binding specific) member 4 [Source:HGNC Symbol;Acc:14339]
ENSG00000108264	17_35799889_G_T_b37	5.54E-25	0.000229998	rs853228	TADA2A	transcriptional adaptor 2A [Source:HGNC Symbol;Acc:11531]
ENSG00000108264	17_35823995_C_T_b37	2.92E-24	0.000229998	rs864083	TADA2A	transcriptional adaptor 2A [Source:HGNC Symbol;Acc:11531]
ENSG00000108264	17_35828164_T_C_b37	2.10E-23	0.000229998	rs853211	TADA2A	transcriptional adaptor 2A [Source:HGNC Symbol;Acc:11531]
ENSG00000006114	17_35799889_G_T_b37	1.06E-22	0.000229998	rs853228	SYNRG	synergin, gamma [Source:HGNC Symbol;Acc:557]
ENSG00000006114	17_35823995_C_T_b37	2.87E-22	0.000229998	rs864083	SYNRG	synergin, gamma [Source:HGNC Symbol;Acc:557]
ENSG00000006114	17_35828164_T_C_b37	2.50E-22	0.000229998	rs853211	SYNRG	synergin, gamma [Source:HGNC Symbol;Acc:557]
ENSG00000165914	14_91230469_G_GTA_b37	1.43E-07	0.000239998	rs201745532	TTC7B	tetratricopeptide repeat domain 7B [Source:HGNC Symbol;Acc:19858]
ENSG00000108264	17_35787666_C_T_b37	5.38E-25	0.000239998	rs853220	TADA2A	transcriptional adaptor 2A [Source:HGNC Symbol;Acc:11531]
ENSG00000108264	17_35807445_G_C_b37	1.49E-24	0.000239998	rs853203	TADA2A	transcriptional adaptor 2A [Source:HGNC Symbol;Acc:11531]
ENSG00000006114	17_35787666_C_T_b37	1.08E-22	0.000239998	rs853220	SYNRG	synergin, gamma [Source:HGNC Symbol;Acc:557]
ENSG00000006114	17_35807445_G_C_b37	6.62E-23	0.000239998	rs853203	SYNRG	synergin, gamma [Source:HGNC Symbol;Acc:557]
ENSG00000204305	6_32539473_C_A_b37	3.77E-06	0.000249998	rs9269294	AGER	advanced glycosylation end product-specific receptor [Source:HGNC Symbol;Acc:320]
ENSG00000204301	6_32539473_C_A_b37	4.12E-06	0.000249998	rs9269294	NOTCH4	notch 4 [Source:HGNC Symbol;Acc:7884]
ENSG00000198502	6_32539473_C_A_b37	2.57E-16	0.000249998	rs9269294	HLA-DRB5	major histocompatibility complex, class II, DR beta 5 [Source:HGNC Symbol;Acc:4953]
ENSG00000196126	6_32539473_C_A_b37	1.54E-22	0.000249998	rs9269294	HLA-DRB1	major histocompatibility complex, class II, DR beta 1 [Source:HGNC Symbol;Acc:4948]
ENSG00000196735	6_32539473_C_A_b37	4.13E-14	0.000249998	rs9269294	HLA-DQA1	major histocompatibility complex, class II, DQ alpha 1 [Source:HGNC Symbol;Acc:4942]
ENSG00000223534	6_32539473_C_A_b37	3.53E-11	0.000249998	rs9269294	HLA-DQB1-AS1	HLA-DQB1 antisense RNA 1 [Source:HGNC Symbol;Acc:39762]
ENSG00000241287	6_32539473_C_A_b37	4.83E-06	0.000249998	rs9269294	XXbac-BPG254F23.6	
ENSG00000179344	6_32539473_C_A_b37	1.35E-12	0.000249998	rs9269294	HLA-DQB1	major histocompatibility complex, class II, DQ beta 1 [Source:HGNC Symbol;Acc:4944]
ENSG00000108264	17_35783416_A_G_b37	5.35E-25	0.000249998	rs72828253	TADA2A	transcriptional adaptor 2A [Source:HGNC Symbol;Acc:11531]
ENSG00000108264	17_35833481_A_T_b37	3.25E-24	0.000249998	rs1859191	TADA2A	transcriptional adaptor 2A [Source:HGNC Symbol;Acc:11531]
ENSG00000006114	17_35783416_A_G_b37	1.09E-22	0.000249998	rs72828253	SYNRG	synergin, gamma [Source:HGNC Symbol;Acc:557]
ENSG00000006114	17_35833481_A_T_b37	2.10E-22	0.000249998	rs1859191	SYNRG	synergin, gamma [Source:HGNC Symbol;Acc:557]
ENSG00000108264	17_35817105_A_G_b37	3.34E-24	0.000269997	rs4795200	TADA2A	transcriptional adaptor 2A [Source:HGNC Symbol;Acc:11531]
ENSG00000108264	17_35843050_C_T_b37	2.65E-24	0.000269997	rs853234	TADA2A	transcriptional adaptor 2A [Source:HGNC Symbol;Acc:11531]
ENSG00000108264	17_35874936_A_G_b37	5.82E-24	0.000269997	rs1063215	TADA2A	transcriptional adaptor 2A [Source:HGNC Symbol;Acc:11531]
ENSG00000006114	17_35817105_A_G_b37	2.24E-22	0.000269997	rs4795200	SYNRG	synergin, gamma [Source:HGNC Symbol;Acc:557]
ENSG00000006114	17_35843050_C_T_b37	9.41E-23	0.000269997	rs853234	SYNRG	synergin, gamma [Source:HGNC Symbol;Acc:557]
ENSG00000006114	17_35874936_A_G_b37	2.33E-25	0.000269997	rs1063215	SYNRG	synergin, gamma [Source:HGNC Symbol;Acc:557]
ENSG00000155542	5_56270111_C_T_b37	5.74E-13	0.000279997	rs76813406	SETD9	SET domain containing 9 [Source:HGNC Symbol;Acc:28508]
ENSG00000108264	17_35828972_T_C_b37	3.27E-24	0.000279997	rs4795203	TADA2A	transcriptional adaptor 2A [Source:HGNC Symbol;Acc:11531]
ENSG00000108264	17_35849342_A_C_b37	4.56E-24	0.000279997	rs853198	TADA2A	transcriptional adaptor 2A [Source:HGNC Symbol;Acc:11531]
ENSG00000006114	17_35828972_T_C_b37	2.13E-22	0.000279997	rs4795203	SYNRG	synergin, gamma [Source:HGNC Symbol;Acc:557]
ENSG00000006114	17_35849342_A_C_b37	1.02E-23	0.000279997	rs853198	SYNRG	synergin, gamma [Source:HGNC Symbol;Acc:557]
ENSG00000087076	19_49381268_TCTC_T_b37	1.48E-10	0.000279997	rs66808476	HSD17B14	hydroxysteroid (17-beta) dehydrogenase 14 [Source:HGNC Symbol;Acc:23238]
ENSG00000087076	19_49382418_G_A_b37	1.85E-10	0.000279997	rs7251410	HSD17B14	hydroxysteroid (17-beta) dehydrogenase 14 [Source:HGNC Symbol;Acc:23238]
ENSG00000105559	19_49381268_TCTC_T_b37	3.74E-05	0.000279997	rs66808476	PLEKHA4	pleckstrin homology domain containing, family A (phosphoinositide binding specific) member 4 [Source:HGNC Symbol;Acc:14339]
ENSG00000105559	19_49382418_G_A_b37	8.13E-05	0.000279997	rs7251410	PLEKHA4	pleckstrin homology domain containing, family A (phosphoinositide binding specific) member 4 [Source:HGNC Symbol;Acc:14339]
ENSG00000140254	15_45495229_G_A_b37	1.79E-06	0.000289997	rs12902212	DUOXA1	dual oxidase maturation factor 1 [Source:HGNC Symbol;Acc:26507]
ENSG00000137857	15_45495229_G_A_b37	2.67E-07	0.000289997	rs12902212	DUOX1	dual oxidase 1 [Source:HGNC Symbol;Acc:3062]
ENSG00000182853	17_4672333_G_A_b37	2.16E-08	0.000289997	rs34009049	VMO1	vitelline membrane outer layer 1 homolog (chicken) [Source:HGNC Symbol;Acc:30387]
ENSG00000108264	17_35821931_G_C_b37	3.31E-24	0.000289997	rs853192	TADA2A	transcriptional adaptor 2A [Source:HGNC Symbol;Acc:11531]
ENSG00000108264	17_35830300_G_A_b37	3.26E-24	0.000289997	rs2522968	TADA2A	transcriptional adaptor 2A [Source:HGNC Symbol;Acc:11531]
ENSG00000006114	17_35821931_G_C_b37	2.20E-22	0.000289997	rs853192	SYNRG	synergin, gamma [Source:HGNC Symbol;Acc:557]
ENSG00000006114	17_35830300_G_A_b37	2.04E-22	0.000289997	rs2522968	SYNRG	synergin, gamma [Source:HGNC Symbol;Acc:557]
ENSG00000155542	5_56275972_C_G_b37	5.43E-13	0.000299997	rs75326369	SETD9	SET domain containing 9 [Source:HGNC Symbol;Acc:28508]
ENSG00000235725	2_65822041_T_C_b37	1.38E-07	0.000309997	rs6546164	AC007389.3	
ENSG00000272209	6_13773149_C_A_b37	8.52E-05	0.000309997	rs9464277	RP3-500L14.2	
ENSG00000108264	17_35816616_C_T_b37	3.33E-24	0.000309997	rs2522966	TADA2A	transcriptional adaptor 2A [Source:HGNC Symbol;Acc:11531]
ENSG00000006114	17_35816616_C_T_b37	2.25E-22	0.000309997	rs2522966	SYNRG	synergin, gamma [Source:HGNC Symbol;Acc:557]
ENSG00000087076	19_49378994_A_G_b37	6.06E-11	0.000319997	rs500079	HSD17B14	hydroxysteroid (17-beta) dehydrogenase 14 [Source:HGNC Symbol;Acc:23238]
ENSG00000105559	19_49378994_A_G_b37	1.89E-05	0.000319997	rs500079	PLEKHA4	pleckstrin homology domain containing, family A (phosphoinositide binding specific) member 4 [Source:HGNC Symbol;Acc:14339]
ENSG00000196301	6_32481655_C_T_b37	6.45E-05	0.000329997	rs75364579	HLA-DRB9	major histocompatibility complex, class II, DR beta 9 (pseudogene) [Source:HGNC Symbol;Acc:4957]
ENSG00000229391	6_32481655_C_T_b37	1.65E-15	0.000329997	rs75364579	HLA-DRB6	major histocompatibility complex, class II, DR beta 6 (pseudogene) [Source:HGNC Symbol;Acc:4954]
ENSG00000179344	6_32481655_C_T_b37	2.82E-05	0.000329997	rs75364579	HLA-DQB1	major histocompatibility complex, class II, DQ beta 1 [Source:HGNC Symbol;Acc:4944]
ENSG00000108264	17_35829668_T_C_b37	4.14E-24	0.000329997	rs7217375	TADA2A	transcriptional adaptor 2A [Source:HGNC Symbol;Acc:11531]
ENSG00000006114	17_35829668_T_C_b37	2.15E-22	0.000329997	rs7217375	SYNRG	synergin, gamma [Source:HGNC Symbol;Acc:557]
ENSG00000087076	19_49380353_C_A_b37	8.34E-10	0.000329997	rs576530	HSD17B14	hydroxysteroid (17-beta) dehydrogenase 14 [Source:HGNC Symbol;Acc:23238]
ENSG00000105559	19_49380353_C_A_b37	1.57E-05	0.000329997	rs576530	PLEKHA4	pleckstrin homology domain containing, family A (phosphoinositide binding specific) member 4 [Source:HGNC Symbol;Acc:14339]
ENSG00000235725	2_65837042_T_C_b37	4.74E-07	0.000339997	rs6749987	AC007389.3	
ENSG00000140254	15_45490773_C_T_b37	1.60E-06	0.000339997	rs12323957	DUOXA1	dual oxidase maturation factor 1 [Source:HGNC Symbol;Acc:26507]
ENSG00000137857	15_45490773_C_T_b37	2.53E-07	0.000339997	rs12323957	DUOX1	dual oxidase 1 [Source:HGNC Symbol;Acc:3062]
ENSG00000127586	16_830953_G_A_b37	4.00E-09	0.000339997	rs3765333	CHTF18	CTF18, chromosome transmission fidelity factor 18 homolog (S. cerevisiae) [Source:HGNC Symbol;Acc:18435]
ENSG00000182853	17_4676020_T_G_b37	4.54E-05	0.000339997	rs7217326	VMO1	vitelline membrane outer layer 1 homolog (chicken) [Source:HGNC Symbol;Acc:30387]

ENSG00000108264	17_35826456_C_A_b37	3.28E-24	0.000339997	rs853208	TADA2A	transcriptional adaptor 2A [Source:HGNC Symbol;Acc:11531]
ENSG00000108264	17_35839021_C_T_b37	3.23E-24	0.000339997	rs2522972	TADA2A	transcriptional adaptor 2A [Source:HGNC Symbol;Acc:11531]
ENSG00000108264	17_35860964_T_C_b37	3.12E-23	0.000339997	rs853214	TADA2A	transcriptional adaptor 2A [Source:HGNC Symbol;Acc:11531]
ENSG00000108264	17_35866325_A_C_b37	2.49E-23	0.000339997	rs11653332	TADA2A	transcriptional adaptor 2A [Source:HGNC Symbol;Acc:11531]
ENSG00000006114	17_35826456_C_A_b37	2.16E-22	0.000339997	rs853208	SYNRG	synergin, gamma [Source:HGNC Symbol;Acc:557]
ENSG00000006114	17_35839021_C_T_b37	2.00E-22	0.000339997	rs2522972	SYNRG	synergin, gamma [Source:HGNC Symbol;Acc:557]
ENSG00000006114	17_35860964_T_C_b37	5.24E-24	0.000339997	rs853214	SYNRG	synergin, gamma [Source:HGNC Symbol;Acc:557]
ENSG00000006114	17_35866325_A_C_b37	2.17E-23	0.000339997	rs11653332	SYNRG	synergin, gamma [Source:HGNC Symbol;Acc:557]
ENSG00000087076	19_49379866_A_G_b37	3.69E-11	0.000339997	rs580907	HSD17B14	hydroxysteroid (17-beta) dehydrogenase 14 [Source:HGNC Symbol;Acc:23238]
ENSG00000087076	19_49396737_G_A_b37	2.54E-06	0.000339997	rs9941490	HSD17B14	hydroxysteroid (17-beta) dehydrogenase 14 [Source:HGNC Symbol;Acc:23238]
ENSG00000235725	2_65820608_T_C_b37	1.31E-13	0.000349997	rs11687213	AC007389.3	
ENSG00000093183	3_42581680_A_G_b37	1.51E-05	0.000349997	rs342507	SEC22C	SEC22 vesicle trafficking protein homolog C (S. cerevisiae) [Source:HGNC Symbol;Acc:16828]
ENSG00000114857	3_42581680_A_G_b37	1.79E-05	0.000349997	rs342507	NKTR	natural killer-tumor recognition sequence [Source:HGNC Symbol;Acc:7833]
ENSG00000108264	17_35835031_G_A_b37	9.96E-25	0.000349997	rs2107104	TADA2A	transcriptional adaptor 2A [Source:HGNC Symbol;Acc:11531]
ENSG00000108264	17_35848255_C_A_b37	5.35E-24	0.000349997	rs853195	TADA2A	transcriptional adaptor 2A [Source:HGNC Symbol;Acc:11531]
ENSG00000006114	17_35835031_G_A_b37	1.13E-21	0.000349997	rs2107104	SYNRG	synergin, gamma [Source:HGNC Symbol;Acc:557]
ENSG00000006114	17_35848255_C_A_b37	2.83E-23	0.000349997	rs853195	SYNRG	synergin, gamma [Source:HGNC Symbol;Acc:557]
ENSG00000087076	19_49378514_A_G_b37	3.93E-11	0.000349997	rs594600	HSD17B14	hydroxysteroid (17-beta) dehydrogenase 14 [Source:HGNC Symbol;Acc:23238]
ENSG00000105559	19_49378514_A_G_b37	3.68E-05	0.000349997	rs594600	PLEKHA4	pleckstrin homology domain containing, family A (phosphoinositide binding specific) member 4 [Source:HGNC Symbol;Acc:14339]
ENSG00000104804	19_49378514_A_G_b37	7.38E-05	0.000349997	rs594600	TULP2	tubby like protein 2 [Source:HGNC Symbol;Acc:12424]
ENSG00000108264	17_35833208_T_C_b37	1.18E-24	0.000359996	rs6607275	TADA2A	transcriptional adaptor 2A [Source:HGNC Symbol;Acc:11531]
ENSG00000006114	17_35833208_T_C_b37	1.53E-21	0.000359996	rs6607275	SYNRG	synergin, gamma [Source:HGNC Symbol;Acc:557]
ENSG00000148426	10_11865304_C_T_b37	6.49E-06	0.000369996	rs4750123	PROSER2	proline and serine-rich protein 2 [Source:HGNC Symbol;Acc:23728]
ENSG00000108264	17_35816318_C_G_b37	3.33E-24	0.000369996	rs2522965	TADA2A	transcriptional adaptor 2A [Source:HGNC Symbol;Acc:11531]
ENSG00000006114	17_35816318_C_G_b37	2.25E-22	0.000369996	rs2522965	SYNRG	synergin, gamma [Source:HGNC Symbol;Acc:557]
ENSG00000108264	17_35856593_A_G_b37	5.13E-24	0.000379996	rs11870754	TADA2A	transcriptional adaptor 2A [Source:HGNC Symbol;Acc:11531]
ENSG00000006114	17_35856593_A_G_b37	4.75E-24	0.000379996	rs11870754	SYNRG	synergin, gamma [Source:HGNC Symbol;Acc:557]
ENSG00000087076	19_49395673_A_C_b37	1.86E-06	0.000379996	rs7254122	HSD17B14	hydroxysteroid (17-beta) dehydrogenase 14 [Source:HGNC Symbol;Acc:23238]
ENSG00000235725	2_65843579_G_T_b37	5.90E-07	0.000399996	rs7557512	AC007389.3	
ENSG00000173376	4_121994727_C_T_b37	2.90E-05	0.000399996	rs28567741	NDNF	neuron-derived neurotrophic factor [Source:HGNC Symbol;Acc:26256]
ENSG00000108264	17_35821758_A_T_b37	3.30E-24	0.000399996	rs853191	TADA2A	transcriptional adaptor 2A [Source:HGNC Symbol;Acc:11531]
ENSG00000006114	17_35821758_A_T_b37	2.20E-22	0.000399996	rs853191	SYNRG	synergin, gamma [Source:HGNC Symbol;Acc:557]
ENSG00000116127	2_73816285_A_G_b37	7.09E-06	0.000409996	rs6722867	ALMS1	Alstrom syndrome 1 [Source:HGNC Symbol;Acc:428]
ENSG00000163016	2_73816285_A_G_b37	1.18E-05	0.000409996	rs6722867	ALMS1P	Alstrom syndrome 1 pseudogene [Source:HGNC Symbol;Acc:29586]
ENSG00000087076	19_49378699_CAGAT_C_b37	2.71E-09	0.000409996	rs5828365	HSD17B14	hydroxysteroid (17-beta) dehydrogenase 14 [Source:HGNC Symbol;Acc:23238]
ENSG00000105559	19_49378699_CAGAT_C_b37	2.89E-05	0.000409996	rs5828365	PLEKHA4	pleckstrin homology domain containing, family A (phosphoinositide binding specific) member 4 [Source:HGNC Symbol;Acc:14339]
ENSG00000104804	19_49378699_CAGAT_C_b37	2.27E-05	0.000409996	rs5828365	TULP2	tubby like protein 2 [Source:HGNC Symbol;Acc:12424]
ENSG00000116127	2_73828538_G_A_b37	4.96E-06	0.000419996	rs1052161	ALMS1	Alstrom syndrome 1 [Source:HGNC Symbol;Acc:428]
ENSG00000163016	2_73828538_G_A_b37	5.88E-06	0.000419996	rs1052161	ALMS1P	Alstrom syndrome 1 pseudogene [Source:HGNC Symbol;Acc:29586]
ENSG00000250321	4_701500_A_G_b37	5.22E-06	0.000419996	rs28507182	RP11-478C6.4	
ENSG00000108264	17_35851177_A_C_b37	4.30E-23	0.000429996	rs865483	TADA2A	transcriptional adaptor 2A [Source:HGNC Symbol;Acc:11531]
ENSG00000108264	17_35857151_G_A_b37	5.13E-24	0.000429996	rs9906140	TADA2A	transcriptional adaptor 2A [Source:HGNC Symbol;Acc:11531]
ENSG00000006114	17_35851177_A_C_b37	2.06E-22	0.000429996	rs865483	SYNRG	synergin, gamma [Source:HGNC Symbol;Acc:557]
ENSG00000006114	17_35857151_G_A_b37	4.75E-24	0.000429996	rs9906140	SYNRG	synergin, gamma [Source:HGNC Symbol;Acc:557]
ENSG00000155542	5_56261691_G_A_b37	3.73E-43	0.000439996	rs2662021	SETD9	SET domain containing 9 [Source:HGNC Symbol;Acc:28508]
ENSG00000108264	17_35868304_T_C_b37	1.62E-23	0.000439996	rs12603185	TADA2A	transcriptional adaptor 2A [Source:HGNC Symbol;Acc:11531]
ENSG00000108264	17_35875511_C_CAA_b37	1.94E-22	0.000439996	rs10648808	TADA2A	transcriptional adaptor 2A [Source:HGNC Symbol;Acc:11531]
ENSG00000006114	17_35868304_T_C_b37	3.59E-25	0.000439996	rs12603185	SYNRG	synergin, gamma [Source:HGNC Symbol;Acc:557]
ENSG00000006114	17_35875511_C_CAA_b37	1.64E-22	0.000439996	rs10648808	SYNRG	synergin, gamma [Source:HGNC Symbol;Acc:557]
ENSG00000217702	2_74363344_C_T_b37	1.03E-05	0.000449996	rs9917179	MGC10955	
ENSG00000108264	17_35848045_A_C_b37	2.56E-24	0.000469995	rs853194	TADA2A	transcriptional adaptor 2A [Source:HGNC Symbol;Acc:11531]
ENSG00000006114	17_35848045_A_C_b37	2.70E-23	0.000469995	rs853194	SYNRG	synergin, gamma [Source:HGNC Symbol;Acc:557]
ENSG00000132185	1_161679644_T_C_b37	3.49E-05	0.000479995	rs7523762	FCRLA	Fc receptor-like A [Source:HGNC Symbol;Acc:18504]
ENSG00000140577	15_91014946_G_A_b37	3.39E-05	0.000479995	rs62021772	CRTC3	CREB regulated transcription coactivator 3 [Source:HGNC Symbol;Acc:26148]
