# Supplementary material for: Meta-Analysis of Polymyositis and Dermatomyositis Microarray Data Reveals Novel Genetic Biomarkers
Source: Genes (Basel). 2019 Oct 30;10(11):864. doi: 10.3390/genes10110864 (PMC6895911; doi:10.3390/genes10110864)
Supplement: Supplementary file 1 [file genes-10-00864-s001.zip › Supplementary_files/Supplementary Table 5.pdf]

Supplementary Table 5. Detected SNPs and eGenes in not sun-exposed skin tissue.

gene	SNP	GTEX_p_value	p_value	rs_id_dbSNP142_GRCh37p13	external_gene_name	description
ENSG00000146833	7_99478804_T_C_b37	1.00E-10	2.00E-06	rs2572019	TRIM4	tripartite motif containing 4 [Source:HGNC Symbol;Acc:16275]
ENSG00000146221	6_44285376_G_A_b37	5.26E-05	4.00E-06	rs2396242	TCTE1	t-complex-associated-testis-expressed 1 [Source:HGNC Symbol;Acc:11693]
ENSG00000146833	7_99477524_G_A_b37	8.05E-11	4.00E-06	rs2527926	TRIM4	tripartite motif containing 4 [Source:HGNC Symbol;Acc:16275]
ENSG00000146833	7_99479638_G_A_b37	1.53E-11	4.00E-06	rs2527925	TRIM4	tripartite motif containing 4 [Source:HGNC Symbol;Acc:16275]
ENSG00000136169	13_50002977_C_CAATA_b37	6.45E-05	4.00E-06	rs66884685	SETDB2	SET domain, bifurcated 2 [Source:HGNC Symbol;Acc:20263]
ENSG00000146833	7_99473229_A_G_b37	1.02E-08	5.00E-06	rs2572024	TRIM4	tripartite motif containing 4 [Source:HGNC Symbol;Acc:16275]
ENSG00000146833	7_99520082_C_T_b37	7.36E-11	7.00E-06	rs2571991	TRIM4	tripartite motif containing 4 [Source:HGNC Symbol;Acc:16275]
ENSG00000146833	7_99499872_A_T_b37	1.50E-11	8.00E-06	rs2527913	TRIM4	tripartite motif containing 4 [Source:HGNC Symbol;Acc:16275]
ENSG00000146833	7_99501313_A_T_b37	1.50E-11	8.00E-06	rs2572003	TRIM4	tripartite motif containing 4 [Source:HGNC Symbol;Acc:16275]
ENSG00000146833	7_99511372_A_G_b37	1.50E-11	8.00E-06	rs2571998	TRIM4	tripartite motif containing 4 [Source:HGNC Symbol;Acc:16275]
ENSG00000146833	7_99519916_A_G_b37	1.50E-11	8.00E-06	rs2571992	TRIM4	tripartite motif containing 4 [Source:HGNC Symbol;Acc:16275]
ENSG00000146833	7_99483731_CTT_C_b37	2.56E-11	9.00E-06	rs58306841	TRIM4	tripartite motif containing 4 [Source:HGNC Symbol;Acc:16275]
ENSG00000146833	7_99484295_G_A_b37	1.54E-11	9.00E-06	rs2572013	TRIM4	tripartite motif containing 4 [Source:HGNC Symbol;Acc:16275]
ENSG00000146833	7_99490956_A_T_b37	1.50E-11	9.00E-06	rs2572008	TRIM4	tripartite motif containing 4 [Source:HGNC Symbol;Acc:16275]
ENSG00000146833	7_99494030_G_A_b37	1.50E-11	9.00E-06	rs2572005	TRIM4	tripartite motif containing 4 [Source:HGNC Symbol;Acc:16275]
ENSG00000146833	7_99494513_C_T_b37	1.75E-11	9.00E-06	rs2527919	TRIM4	tripartite motif containing 4 [Source:HGNC Symbol;Acc:16275]
ENSG00000146833	7_99500911_A_G_b37	1.50E-11	9.00E-06	rs2247761	TRIM4	tripartite motif containing 4 [Source:HGNC Symbol;Acc:16275]
ENSG00000146833	7_99518271_C_A_b37	2.47E-11	9.00E-06	rs2571994	TRIM4	tripartite motif containing 4 [Source:HGNC Symbol;Acc:16275]
ENSG00000146833	7_99519575_T_C_b37	1.50E-11	9.00E-06	rs2571993	TRIM4	tripartite motif containing 4 [Source:HGNC Symbol;Acc:16275]
ENSG00000136169	13_50044981_A_G_b37	5.42E-05	9.00E-06	rs7994566	SETDB2	SET domain, bifurcated 2 [Source:HGNC Symbol;Acc:20263]
ENSG00000146833	7_99481492_A_C_b37	1.53E-11	1.00E-05	rs2572015	TRIM4	tripartite motif containing 4 [Source:HGNC Symbol;Acc:16275]
ENSG00000146833	7_99521448_C_T_b37	1.50E-11	1.00E-05	rs2527907	TRIM4	tripartite motif containing 4 [Source:HGNC Symbol;Acc:16275]
ENSG00000146833	7_99525112_A_T_b37	1.45E-10	1.00E-05	rs2527901	TRIM4	tripartite motif containing 4 [Source:HGNC Symbol;Acc:16275]
ENSG00000146833	7_99480193_T_C_b37	1.53E-11	1.10E-05	rs2572018	TRIM4	tripartite motif containing 4 [Source:HGNC Symbol;Acc:16275]
ENSG00000146833	7_99484473_G_A_b37	1.53E-11	1.10E-05	rs2572012	TRIM4	tripartite motif containing 4 [Source:HGNC Symbol;Acc:16275]
ENSG00000146833	7_99493811_G_A_b37	1.50E-11	1.10E-05	rs2572006	TRIM4	tripartite motif containing 4 [Source:HGNC Symbol;Acc:16275]
ENSG00000146833	7_99512224_G_T_b37	1.50E-11	1.10E-05	rs2527909	TRIM4	tripartite motif containing 4 [Source:HGNC Symbol;Acc:16275]
ENSG00000146833	7_99514417_A_C_b37	1.50E-11	1.10E-05	rs2571997	TRIM4	tripartite motif containing 4 [Source:HGNC Symbol;Acc:16275]
ENSG00000146833	7_99498499_A_T_b37	1.50E-11	1.20E-05	rs10808114	TRIM4	tripartite motif containing 4 [Source:HGNC Symbol;Acc:16275]
ENSG00000146833	7_99521698_C_T_b37	1.50E-11	1.20E-05	rs2527906	TRIM4	tripartite motif containing 4 [Source:HGNC Symbol;Acc:16275]
ENSG00000146833	7_99529181_A_G_b37	2.07E-11	1.20E-05	rs2527899	TRIM4	tripartite motif containing 4 [Source:HGNC Symbol;Acc:16275]
ENSG00000146833	7_99537733_G_A_b37	1.90E-05	1.20E-05	rs11979058	TRIM4	tripartite motif containing 4 [Source:HGNC Symbol;Acc:16275]
ENSG00000136169	13_50020280_C_G_b37	7.48E-05	1.20E-05	rs61959992	SETDB2	SET domain, bifurcated 2 [Source:HGNC Symbol;Acc:20263]
ENSG00000146833	7_99488940_A_T_b37	1.48E-11	1.30E-05	rs1048705	TRIM4	tripartite motif containing 4 [Source:HGNC Symbol;Acc:16275]
ENSG00000146833	7_99498018_A_C_b37	1.50E-11	1.30E-05	rs2527915	TRIM4	tripartite motif containing 4 [Source:HGNC Symbol;Acc:16275]
ENSG00000146833	7_99451283_T_G_b37	5.28E-05	1.40E-05	rs523265	TRIM4	tripartite motif containing 4 [Source:HGNC Symbol;Acc:16275]
ENSG00000146833	7_99492756_A_G_b37	1.50E-11	1.40E-05	rs2527922	TRIM4	tripartite motif containing 4 [Source:HGNC Symbol;Acc:16275]
ENSG00000146833	7_99510850_T_A_b37	1.54E-11	1.50E-05	rs2571999	TRIM4	tripartite motif containing 4 [Source:HGNC Symbol;Acc:16275]
ENSG00000120805	12_101690273_C_T_b37	1.72E-05	1.50E-05	rs7296602	ARL1	ADP-ribosylation factor-like 1 [Source:HGNC Symbol;Acc:692]
ENSG00000136169	13_50057996_C_T_b37	2.94E-05	1.50E-05	rs9568219	SETDB2	SET domain, bifurcated 2 [Source:HGNC Symbol;Acc:20263]
ENSG00000146833	7_99488543_C_T_b37	1.50E-11	1.60E-05	rs2572010	TRIM4	tripartite motif containing 4 [Source:HGNC Symbol;Acc:16275]
ENSG00000146833	7_99493270_G_C_b37	1.49E-11	1.70E-05	rs2527921	TRIM4	tripartite motif containing 4 [Source:HGNC Symbol;Acc:16275]
ENSG00000124733	6_43350753_C_T_b37	7.60E-05	2.00E-05	rs9394951	MEA1	male-enhanced antigen 1 [Source:HGNC Symbol;Acc:6986]
ENSG00000124733	6_43355851_A_G_b37	6.79E-05	2.00E-05	rs2396004	MEA1	male-enhanced antigen 1 [Source:HGNC Symbol;Acc:6986]
ENSG00000146833	7_99459144_T_C_b37	2.89E-11	2.30E-05	rs503115	TRIM4	tripartite motif containing 4 [Source:HGNC Symbol;Acc:16275]
ENSG00000124733	6_43352898_G_A_b37	7.60E-05	2.40E-05	rs6919440	MEA1	male-enhanced antigen 1 [Source:HGNC Symbol;Acc:6986]
ENSG00000146833	7_99474729_T_C_b37	6.38E-11	2.50E-05	rs2572022	TRIM4	tripartite motif containing 4 [Source:HGNC Symbol;Acc:16275]
ENSG00000166924	7_99474729_T_C_b37	6.59E-05	2.50E-05	rs2572022	NYAP1	neuronal tyrosine-phosphorylated phosphoinositide-3-kinase adaptor 1 [Source:HGNC Symbol;Acc:22009]
ENSG00000146833	7_99462904_T_TG_b37	2.80E-11	2.60E-05	rs202184595	TRIM4	tripartite motif containing 4 [Source:HGNC Symbol;Acc:16275]
ENSG00000146833	7_99515982_C_G_b37	4.29E-11	2.60E-05	rs7809747	TRIM4	tripartite motif containing 4 [Source:HGNC Symbol;Acc:16275]
ENSG00000146833	7_99466558_G_T_b37	1.72E-11	2.80E-05	rs474229	TRIM4	tripartite motif containing 4 [Source:HGNC Symbol;Acc:16275]
ENSG00000124733	6_43364494_G_A_b37	3.38E-05	3.00E-05	rs6912283	MEA1	male-enhanced antigen 1 [Source:HGNC Symbol;Acc:6986]
ENSG00000140009	14_64694195_C_T_b37	3.71E-05	3.00E-05	rs928554	ESR2	estrogen receptor 2 (ER beta) [Source:HGNC Symbol;Acc:3468]
ENSG00000146833	7_99574738_T_A_b37	7.27E-05	3.20E-05	rs2247607	TRIM4	tripartite motif containing 4 [Source:HGNC Symbol;Acc:16275]
ENSG00000146833	7_99473624_T_G_b37	2.70E-11	3.30E-05	rs1025576	TRIM4	tripartite motif containing 4 [Source:HGNC Symbol;Acc:16275]
ENSG00000120805	12_101682211_A_G_b37	2.11E-05	3.40E-05	rs703713	ARL1	ADP-ribosylation factor-like 1 [Source:HGNC Symbol;Acc:692]
ENSG00000146833	7_99498145_G_A_b37	1.05E-11	4.10E-05	rs2527914	TRIM4	tripartite motif containing 4 [Source:HGNC Symbol;Acc:16275]
ENSG00000146833	7_99503625_A_G_b37	1.28E-10	4.40E-05	rs2527911	TRIM4	tripartite motif containing 4 [Source:HGNC Symbol;Acc:16275]
ENSG00000120805	12_101707067_A_G_b37	3.26E-05	4.40E-05	rs7980431	ARL1	ADP-ribosylation factor-like 1 [Source:HGNC Symbol;Acc:692]
ENSG00000233621	1_37930294_C_A_b37	1.22E-10	4.50E-05	rs2811642	LINC01137	long intergenic non-protein coding RNA 1137 [Source:HGNC Symbol;Acc:49453]
ENSG00000136169	13_50062327_C_T_b37	2.44E-05	4.60E-05	rs959421	SETDB2	SET domain, bifurcated 2 [Source:HGNC Symbol;Acc:20263]
ENSG00000146833	7_99489005_A_G_b37	1.04E-11	4.90E-05	rs2572009	TRIM4	tripartite motif containing 4 [Source:HGNC Symbol;Acc:16275]
ENSG00000146833	7_99515100_T_C_b37	8.55E-12	5.00E-05	rs2571996	TRIM4	tripartite motif containing 4 [Source:HGNC Symbol;Acc:16275]
ENSG00000136169	13_50053063_A_G_b37	2.79E-05	5.40E-05	rs9526564	SETDB2	SET domain, bifurcated 2 [Source:HGNC Symbol;Acc:20263]
ENSG00000272668	1_159822219_C_T_b37	2.63E-14	6.00E-05	rs2494498	RP11-190A12.8	
ENSG00000146833	7_99510166_AT_A_b37	1.75E-10	6.00E-05	rs67670034	TRIM4	tripartite motif containing 4 [Source:HGNC Symbol;Acc:16275]
ENSG00000136169	13_50027270_C_A_b37	1.33E-05	6.00E-05	rs7982297	SETDB2	SET domain, bifurcated 2 [Source:HGNC Symbol;Acc:20263]
ENSG00000136169	13_50044675_T_C_b37	1.33E-05	6.00E-05	rs6561525	SETDB2	SET domain, bifurcated 2 [Source:HGNC Symbol;Acc:20263]
ENSG00000136169	13_50057097_G_A_b37	2.79E-05	6.00E-05	rs2057413	SETDB2	SET domain, bifurcated 2 [Source:HGNC Symbol;Acc:20263]
ENSG00000140009	14_64695087_A_G_b37	3.91E-05	6.00E-05	rs1152579	ESR2	estrogen receptor 2 (ER beta) [Source:HGNC Symbol;Acc:3468]
ENSG00000140009	14_64697485_T_G_b37	9.16E-05	6.00E-05	rs1152577	ESR2	estrogen receptor 2 (ER beta) [Source:HGNC Symbol;Acc:3468]
ENSG00000146833	7_99487152_T_TA_b37	9.75E-12	6.20E-05	rs59413551	TRIM4	tripartite motif containing 4 [Source:HGNC Symbol;Acc:16275]
ENSG00000136169	13_50013622_A_C_b37	1.33E-05	7.00E-05	rs9316453	SETDB2	SET domain, bifurcated 2 [Source:HGNC Symbol;Acc:20263]
ENSG00000136169	13_50031536_A_AG_b37	2.87E-05	7.00E-05	rs35097626	SETDB2	SET domain, bifurcated 2 [Source:HGNC Symbol;Acc:20263]
ENSG00000136169	13_50054308_G_A_b37	2.79E-05	7.00E-05	rs4142285	SETDB2	SET domain, bifurcated 2 [Source:HGNC Symbol;Acc:20263]
ENSG00000140009	14_64697037_T_C_b37	9.51E-05	7.00E-05	rs1152578	ESR2	estrogen receptor 2 (ER beta) [Source:HGNC Symbol;Acc:3468]
ENSG00000136169	13_50012370_A_G_b37	1.33E-05	7.00E-05	rs7321670	SETDB2	SET domain, bifurcated 2 [Source:HGNC Symbol;Acc:20263]
ENSG00000120805	12_101713141_C_T_b37	2.16E-05	7.30E-05	rs2290719	ARL1	ADP-ribosylation factor-like 1 [Source:HGNC Symbol;Acc:692]
ENSG00000136169	13_50007393_C_T_b37	1.33E-05	7.40E-05	rs7998660	SETDB2	SET domain, bifurcated 2 [Source:HGNC Symbol;Acc:20263]
ENSG00000124733	6_43349308_A_C_b37	3.40E-05	8.00E-05	rs7763350	MEA1	male-enhanced antigen 1 [Source:HGNC Symbol;Acc:6986]
ENSG00000146833	7_99481344_T_G_b37	1.04E-11	8.00E-05	rs2082745	TRIM4	tripartite motif containing 4 [Source:HGNC Symbol;Acc:16275]
ENSG00000136169	13_50008301_A_G_b37	1.33E-05	8.00E-05	rs3764090	SETDB2	SET domain, bifurcated 2 [Source:HGNC Symbol;Acc:20263]
ENSG00000136169	13_50010199_T_C_b37	2.07E-05	8.00E-05	rs1322787	SETDB2	SET domain, bifurcated 2 [Source:HGNC Symbol;Acc:20263]
ENSG00000136169	13_50015428_C_A_b37	1.33E-05	8.00E-05	rs7982598	SETDB2	SET domain, bifurcated 2 [Source:HGNC Symbol;Acc:20263]
ENSG00000136169	13_50054346_G_T_b37	2.79E-05	8.00E-05	rs7996852	SETDB2	SET domain, bifurcated 2 [Source:HGNC Symbol;Acc:20263]
ENSG00000136169	13_50060676_T_G_b37	2.57E-05	8.00E-05	rs7321228	SETDB2	SET domain, bifurcated 2 [Source:HGNC Symbol;Acc:20263]
ENSG00000146833	7_99481416_C_T_b37	1.04E-11	9.00E-05	rs2082744	TRIM4	tripartite motif containing 4 [Source:HGNC Symbol;Acc:16275]
ENSG00000146833	7_99524694_A_G_b37	1.10E-11	9.00E-05	rs2527903	TRIM4	tripartite motif containing 4 [Source:HGNC Symbol;Acc:16275]
ENSG00000120805	12_101722005_A_C_b37	2.45E-05	9.00E-05	rs7313828	ARL1	ADP-ribosylation factor-like 1 [Source:HGNC Symbol;Acc:692]
ENSG00000136169	13_50008646_G_C_b37	2.16E-05	9.00E-05	rs7987940	SETDB2	SET domain, bifurcated 2 [Source:HGNC Symbol;Acc:20263]
ENSG00000172273	11_119029849_T_C_b37	4.57E-06	1.00E-04	rs485030	HINFP	histone H4 transcription factor [Source:HGNC Symbol;Acc:17850]
ENSG00000136169	13_50008112_G_A_b37	1.35E-05	1.00E-04	rs3764091	SETDB2	SET domain, bifurcated 2 [Source:HGNC Symbol;Acc:20263]
ENSG00000136169	13_50043820_A_G_b37	1.33E-05	1.00E-04	rs2407692	SETDB2	SET domain, bifurcated 2 [Source:HGNC Symbol;Acc:20263]
ENSG00000136169	13_50073563_C_G_b37	9.48E-05	1.00E-04	rs3794381	SETDB2	SET domain, bifurcated 2 [Source:HGNC Symbol;Acc:20263]
ENSG00000120805	12_101706987_A_G_b37	4.38E-05	0.000109999	rs1471130	ARL1	ADP-ribosylation factor-like 1 [Source:HGNC Symbol;Acc:692]
ENSG00000136169	13_50006884_G_A_b37	1.34E-05	0.000109999	rs7998022	SETDB2	SET domain, bifurcated 2 [Source:HGNC Symbol;Acc:20263]
ENSG00000136169	13_50009798_A_G_b37	1.34E-05	0.000109999	rs7330512	SETDB2	SET domain, bifurcated 2 [Source:HGNC Symbol;Acc:20263]
ENSG00000233621	1_37922609_A_T_b37	7.44E-13	0.000119999	rs2811617	LINC01137	long intergenic non-protein coding RNA 1137 [Source:HGNC Symbol;Acc:49453]
ENSG00000136169	13_50025495_T_A_b37	4.21E-05	0.000119999	rs1543514	SETDB2	SET domain, bifurcated 2 [Source:HGNC Symbol;Acc:20263]
ENSG00000136169	13_50031535_C_CA_b37	5.49E-05	0.000119999	rs201518296	SETDB2	SET domain, bifurcated 2 [Source:HGNC Symbol;Acc:20263]
ENSG00000140009	14_64700292_C_T_b37	8.49E-05	0.000119999	rs944047	ESR2	estrogen receptor 2 (ER beta) [Source:HGNC Symbol;Acc:3468]
ENSG00000140009	14_64700299_G_A_b37	8.49E-05	0.000119999	rs944046	ESR2	estrogen receptor 2 (ER beta) [Source:HGNC Symbol;Acc:3468]
ENSG00000272668	1_159827011_C_T_b37	1.22E-09	0.000129999	rs2494501	RP11-190A12.8	
ENSG00000115841	2_38173070_C_T_b37	7.29E-06	0.000129999	rs4670798	RMDN2	regulator of microtubule dynamics 2 [Source:HGNC Symbol;Acc:26567]
ENSG00000146833	7_99510167_T_A_b37	3.26E-10	0.000129999	rs34277597	TRIM4	tripartite motif containing 4 [Source:HGNC Symbol;Acc:16275]
ENSG00000146833	7_99568753_T_G_b37	3.63E-05	0.000129999	rs2527910	TRIM4	tripartite motif containing 4 [Source:HGNC Symbol;Acc:16275]
ENSG00000166997	7_99568753_T_G_b37	7.30E-05	0.000129999	rs2527910	CNPY4	canopy FGF signaling regulator 4 [Source:HGNC Symbol;Acc:28631]
ENSG00000233621	1_37930245_C_T_b37	1.90E-10	0.000139999	rs2660563	LINC01137	long intergenic non-protein coding RNA 1137 [Source:HGNC Symbol;Acc:49453]
ENSG00000272668	1_159822670_A_T_b37	2.01E-10	0.000139999	rs2501338	RP11-190A12.8	
ENSG00000124733	6_43354431_A_G_b37	3.40E-05	0.000139999	rs1214761	MEA1	male-enhanced antigen 1 [Source:HGNC Symbol;Acc:6986]
ENSG00000136169	13_50066721_C_T_b37	4.53E-05	0.000139999	rs7324622	SETDB2	SET domain, bifurcated 2 [Source:HGNC Symbol;Acc:20263]
ENSG00000204632	6_29817106_T_A_b37	4.73E-05	0.000149999	rs2523765	HLA-G	major histocompatibility complex, class I, G [Source:HGNC Symbol;Acc:4964]
ENSG00000206341	6_29817106_T_A_b37	1.52E-12	0.000149999	rs2523765	HLA-H	major histocompatibility complex, class I, H (pseudogene) [Source:HGNC Symbol;Acc:4965]

ENSG00000230795	6_29817106_T_A_b37	9.91E-05	0.000149999	rs2523765	HLA-K	major histocompatibility complex, class I, K (pseudogene) [Source:HGNC Symbol;Acc:4969]
ENSG00000227766	6_29817106_T_A_b37	5.59E-15	0.000149999	rs2523765	HCG4P5	HLA complex group 4 pseudogene 5 [Source:HGNC Symbol;Acc:22925]
ENSG00000204622	6_29817106_T_A_b37	2.94E-13	0.000149999	rs2523765	HLA-J	major histocompatibility complex, class I, J (pseudogene) [Source:HGNC Symbol;Acc:4967]
ENSG00000124733	6_43349795_G_A_b37	2.21E-05	0.000149999	rs7764523	MEA1	male-enhanced antigen 1 [Source:HGNC Symbol;Acc:6986]
ENSG00000124733	6_43352980_A_G_b37	3.40E-05	0.000149999	rs1214759	MEA1	male-enhanced antigen 1 [Source:HGNC Symbol;Acc:6986]
ENSG00000136169	13_50022089_G_C_b37	4.83E-05	0.000149999	rs7992603	SETDB2	SET domain, bifurcated 2 [Source:HGNC Symbol;Acc:20263]
ENSG00000233621	1_37930629_T_C_b37	1.33E-12	0.000159998	rs2783724	LINC01137	long intergenic non-protein coding RNA 1137 [Source:HGNC Symbol;Acc:49453]
ENSG00000136169	13_50012949_C_T_b37	3.24E-05	0.000159998	rs7327520	SETDB2	SET domain, bifurcated 2 [Source:HGNC Symbol;Acc:20263]
ENSG00000124733	6_43349215_G_A_b37	2.59E-05	0.000169998	rs7763558	MEA1	male-enhanced antigen 1 [Source:HGNC Symbol;Acc:6986]
ENSG00000136169	13_50040593_T_G_b37	8.59E-06	0.000169998	rs7328782	SETDB2	SET domain, bifurcated 2 [Source:HGNC Symbol;Acc:20263]
ENSG00000136169	13_50066643_T_A_b37	4.55E-05	0.000169998	rs6561527	SETDB2	SET domain, bifurcated 2 [Source:HGNC Symbol;Acc:20263]
ENSG00000138821	4_103319643_A_G_b37	7.00E-05	0.000179998	rs80271034	SLC39A8	solute carrier family 39 (zinc transporter), member 8 [Source:HGNC Symbol;Acc:20862]
ENSG00000140009	14_64686207_A_G_b37	9.87E-05	0.000179998	rs915057	ESR2	estrogen receptor 2 (ER beta) [Source:HGNC Symbol;Acc:3468]
ENSG00000233621	1_37927023_A_G_b37	2.63E-12	0.000189998	rs2811638	LINC01137	long intergenic non-protein coding RNA 1137 [Source:HGNC Symbol;Acc:49453]
ENSG00000233621	1_37932018_C_T_b37	1.37E-12	0.000189998	rs2783727	LINC01137	long intergenic non-protein coding RNA 1137 [Source:HGNC Symbol;Acc:49453]
ENSG00000138821	4_103322304_T_C_b37	7.00E-05	0.000189998	rs61325025	SLC39A8	solute carrier family 39 (zinc transporter), member 8 [Source:HGNC Symbol;Acc:20862]
ENSG00000175643	16_11461071_C_G_b37	5.28E-09	0.000199998	rs9938869	RMI2	RecQ mediated genome instability 2 [Source:HGNC Symbol;Acc:28349]
ENSG00000233621	1_37922859_C_T_b37	6.51E-14	0.000209998	rs2660569	LINC01137	long intergenic non-protein coding RNA 1137 [Source:HGNC Symbol;Acc:49453]
ENSG00000124762	6_37593165_T_C_b37	4.81E-05	0.000209998	rs115736884	CDKN1A	cyclin-dependent kinase inhibitor 1A (p21, Cip1) [Source:HGNC Symbol;Acc:1784]
ENSG00000136169	13_50059531_T_C_b37	4.15E-05	0.000219998	rs1409017	SETDB2	SET domain, bifurcated 2 [Source:HGNC Symbol;Acc:20263]
ENSG00000153406	16_4560627_T_A_b37	5.49E-09	0.000219998	rs1477123	NMRAL1	NmrA-like family domain containing 1 [Source:HGNC Symbol;Acc:24987]
ENSG00000089486	16_4560627_T_A_b37	2.72E-05	0.000219998	rs1477123	CDIP1	cell death-inducing p53 target 1 [Source:HGNC Symbol;Acc:13234]
ENSG00000272668	1_159827475_G_A_b37	9.96E-10	0.000229998	rs11584200	RP11-190A12.8	
ENSG00000136169	13_50005444_T_C_b37	1.32E-05	0.000229998	rs1358991	SETDB2	SET domain, bifurcated 2 [Source:HGNC Symbol;Acc:20263]
ENSG00000136169	13_50005780_C_T_b37	5.95E-06	0.000239998	rs1853324	SETDB2	SET domain, bifurcated 2 [Source:HGNC Symbol;Acc:20263]
ENSG00000233621	1_37930414_A_G_b37	9.04E-13	0.000249998	rs2660564	LINC01137	long intergenic non-protein coding RNA 1137 [Source:HGNC Symbol;Acc:49453]
ENSG00000272668	1_159824205_C_T_b37	9.13E-08	0.000249998	rs11807946	RP11-190A12.8	
ENSG00000146833	7_99475473_T_C_b37	2.45E-11	0.000249998	rs2060452	TRIM4	tripartite motif containing 4 [Source:HGNC Symbol;Acc:16275]
ENSG00000166924	7_99475473_T_C_b37	4.79E-05	0.000249998	rs2060452	NYAP1	neuronal tyrosine-phosphorylated phosphoinositide-3-kinase adaptor 1 [Source:HGNC Symbol;Acc:22009]
ENSG00000136169	13_50009874_C_T_b37	5.15E-05	0.000249998	rs9535243	SETDB2	SET domain, bifurcated 2 [Source:HGNC Symbol;Acc:20263]
ENSG00000136169	13_50068628_A_G_b37	4.14E-05	0.000249998	rs7337488	SETDB2	SET domain, bifurcated 2 [Source:HGNC Symbol;Acc:20263]
ENSG00000187990	6_25315540_T_C_b37	8.38E-05	0.000269997	rs6936496	HIST1H2BG	histone cluster 1, H2bg [Source:HGNC Symbol;Acc:4746]
ENSG00000154548	6_89772333_T_C_b37	4.87E-07	0.000269997	rs7760084	SRSF12	serine/arginine-rich splicing factor 12 [Source:HGNC Symbol;Acc:21220]
ENSG00000233621	1_37929005_G_A_b37	1.13E-12	0.000289997	rs2811641	LINC01137	long intergenic non-protein coding RNA 1137 [Source:HGNC Symbol;Acc:49453]
ENSG00000233621	1_37921596_G_A_b37	4.94E-13	0.000299997	rs2492298	LINC01137	long intergenic non-protein coding RNA 1137 [Source:HGNC Symbol;Acc:49453]
ENSG00000272668	1_159827416_C_A_b37	9.75E-10	0.000299997	rs4477268	RP11-190A12.8	
ENSG00000136169	13_50038080_G_A_b37	3.92E-05	0.000299997	rs12429813	SETDB2	SET domain, bifurcated 2 [Source:HGNC Symbol;Acc:20263]
ENSG00000136169	13_50057287_A_G_b37	4.15E-05	0.000299997	rs2057414	SETDB2	SET domain, bifurcated 2 [Source:HGNC Symbol;Acc:20263]
ENSG00000136169	13_50058686_G_A_b37	5.02E-05	0.000299997	rs2057416	SETDB2	SET domain, bifurcated 2 [Source:HGNC Symbol;Acc:20263]
ENSG00000254419	20_57299586_G_A_b37	7.74E-05	0.000319997	rs6128410	RP11-261P9.4	
ENSG00000146833	7_99574758_A_G_b37	2.64E-08	0.000369996	rs2283017	TRIM4	tripartite motif containing 4 [Source:HGNC Symbol;Acc:16275]
ENSG00000085514	7_99574758_A_G_b37	8.40E-05	0.000369996	rs2283017	PILRA	paired immunoglobin-like type 2 receptor alpha [Source:HGNC Symbol;Acc:20396]
ENSG00000233621	1_37919466_G_A_b37	2.06E-12	0.000379996	rs12401987	LINC01137	long intergenic non-protein coding RNA 1137 [Source:HGNC Symbol;Acc:49453]
ENSG00000233621	1_37930001_C_T_b37	8.88E-13	0.000379996	rs2660562	LINC01137	long intergenic non-protein coding RNA 1137 [Source:HGNC Symbol;Acc:49453]
ENSG00000233621	1_37930917_T_C_b37	9.25E-13	0.000379996	rs2783725	LINC01137	long intergenic non-protein coding RNA 1137 [Source:HGNC Symbol;Acc:49453]
ENSG00000233621	1_37927981_A_C_b37	9.50E-13	0.000389996	rs3122179	LINC01137	long intergenic non-protein coding RNA 1137 [Source:HGNC Symbol;Acc:49453]
ENSG00000233621	1_37932566_A_G_b37	4.88E-12	0.000389996	rs2484555	LINC01137	long intergenic non-protein coding RNA 1137 [Source:HGNC Symbol;Acc:49453]
ENSG00000233621	1_37931752_T_C_b37	9.25E-13	0.000399996	rs2245873	LINC01137	long intergenic non-protein coding RNA 1137 [Source:HGNC Symbol;Acc:49453]
ENSG00000136169	13_50018840_CA_C_b37	3.71E-05	0.000459995	rs71307668	SETDB2	SET domain, bifurcated 2 [Source:HGNC Symbol;Acc:20263]
ENSG00000148803	10_134254005_G_A_b37	1.59E-06	0.000469995	rs189735161	FUOM	fucose mutarotase [Source:HGNC Symbol;Acc:24733]
